# Transcriptome profiling of *Paraburkholderia aromaticivorans* AR20-38 during ferulic acid bioconversion

**DOI:** 10.1186/s13568-022-01487-7

**Published:** 2022-11-26

**Authors:** Caroline Poyntner, Thomas Marek Ludwikowski, Andreas Otto Wagner, Rosa Margesin

**Affiliations:** Department of Microbiology, University of Innsbruck, Technikerstraße 25, 6020 Innsbruck, Austria

**Keywords:** Ferulic acid, Vanillic acid, Transcriptome, Bioconversion, *Paraburkholderia*

## Abstract

The importance and need of renewable-based, sustainable feedstocks increased in recent years. Lignin-derived monomers have high potential, energetic and economic value in the microbial bioconversion to valuable biomolecules. The bacterium *Paraburkholderia aromaticivorans* AR20-38 produces a remarkable yield of vanillic acid from ferulic acid at moderate and low temperatures and is therefore a good candidate for biotechnological applications. To understand this bioconversion process on a molecular level, a transcriptomic study during the bioconversion process was conducted to elucidate gene expression patterns. Differentially expressed genes, cellular transporters as well as transcriptional factors involved in the bioconversion process could be described. Additional enzymes known for xenobiotic degradation were differentially expressed and a potential membrane vesicle mechanism was detected. The bioconversion mechanism on a transcriptional level of *P. aromaticivorans* could be elucidated and results can be used for strain optimization. Additionally, the transcriptome study showed the high potential of the strain for other degradation applications.

**Supplementary Information:**

The online version contains supplementary material available at 10.1186/s13568-022-01487-7.

## Keypoints

- *P. aromaticivorans* AR20-38 differentially regulates three main enzymes for the bioconversion of ferulic acid to vanillic acid.
- ATP binding cassette transporters, major facilitator superfamily transporters and outer membrane vesicles are putative transport routes for catabolic enzymes.
- Regulated degradation enzymes indicate xenobiotic degradation potential

**Supplementary Information:**

The online version contains supplementary material available at 10.1186/s13568-022-01487-7.

## Introduction

Lignin is the second most abundant component of plants. It is a three-dimensional complex molecule consisting of various aromatic compounds. To enter the carbon cycle, lignin is degraded to lignin monomers by extracellular enzymes of bacteria and fungi (Bugg et al. 2011). Subsequently, less complex aromatic compounds can be used as carbon source by other microbes. For example, lignin-derived bi- and monoaryls can be degraded by bacteria (Kamimura et al. 2017). These processes are crucial for ecosystem functioning of the natural environment and may be utilized in the valorization of renewable feedstocks for industrial applications. The substitution of fossil fuel-based to renewable-based, nonfood feedstocks has been suggested as sustainable solution for environmental problems (Anastas and Eghbali 2010). This leads to an increased interest in lignin valorization as well as biocatalytic bioconversion of e.g. ferulic acid (FA) as feedstock to produce valuable biomolecules (Cao et al. 2018; Civolani et al. 2000; Graf and Altenbuchner 2014; Mori et al. 2021). Moreover, microorganisms being good degraders of aromatic compounds could harbor applicable enzymes for pollutant or synthetic polymer degradation (Fuchs et al. 2011; Knott et al. 2020).

Within this group of promising microbial candidates to be utilized for such applications are members of the genus *Paraburkholderia*. *Paraburkholderia aromaticivorans* BN5^T^ is a hydrocarbon degrading bacterium, which was previously isolated from gasoline-contaminated soil (Lee and Jeon 2018). Berger et al. (2021) described the isolation of the strain *Paraburkholderia aromaticivorans* AR20-from Alpine forest soil. The microorganism was able to utilize a high amount of lignin sulfonic acid and phenol as sole carbon source (Berger et al. 2021). The cold adapted, Gram-negative bacterium belongs to the group of *Betaproteobacteria* and exhibits its degradation capability over a wide range of temperatures (0–30 °C). A rich genomic toolkit enables the strain to express various enzymes capable of degrading additional lignin monomers, including *p*-coumaric acid, 4-hydroxybenzoic acid, FA, vanillic acid (VA) and benzoic acid (Margesin et al. 2021; Poyntner et al. 2020). The high bioconversion capacity (88–89%) of FA to vanillin and further to VA at low (10 °C) and moderate (20 °C, 30 °C) temperatures is particularly interesting for biotechnological applications. A comparable high yield from pure FA was previously only reported from an engineered *Pseudomonas putida* strain KT2440 (Upadhyay et al. 2020).

The degradation of FA to vanillin has been intensively studied in different microbes due to the utilization of vanillin as a flavoring agent (Graf and Altenbuchner 2014). Additionally, there is a growing interest in the microbial production of medium chain-length polyhydroxyyalkanoates using FA as a non-fatty acid feedstock. Therefore, the improvement was studied in *P. putida* using CRISPR/Cas9 (Zhou et al. 2020). To date, however, studies on cold-adapted strains are scarce even though these strains may be of particular interest for cost-efficient applications in a number of circumstances where maintaining high process temperatures is not a viable option.

To elucidate the gene expression patterns during the bioconversion of FA to VA in *P. aromaticivorans* we here investigated the transcriptomic response in the exponential growth phase with FA as sole carbon source. Therein, the differentially expressed genes responsible for the individual bioconversion stages of FA to VA in *P. aromaticivorans* were identified and putative transcription factors and transporters were linked to the FA bioconversion.

## Materials and methods

### Experimental setup

The experimental setup based on two different culture conditions: the bacterial strain *P. aromaticivorans* cultured with glucose or FA as sole carbon source. The transcriptome during the bioconversion was sequenced and the differential expression during cultivation with FA in comparison to glucose was determined. The bioconversion was monitored with high pressure liquid chromatography (HPLC) measurements.

### Culture conditions and RNA extraction

The experimental setup was based on the bacterial strain *P. aromaticivorans* cultured with three cultures with glucose and three cultures with FA as sole carbon source. Subsequentially, from each culture RNA was extracted resulting in six RNA extracts which were further sequenced. The bioconversion to VA was monitored with HPLC. The strain *P. aromaticivorans* AR20-38 (deposited at China General Microbiological culture collection center under the number CGMCC 1.18749) was precultured in 20 mL pH-neutral mineral medium (MM: 3.5 g L^−1^ Na_2_HPO_4_ × 2H_2_O, 2 g L^−1^ KH_2_PO_4_, 1 g L^−1^ (NH_4_)_2_SO_4_, 0.2 g L^−1^ MgSO_4_ × 7 H_2_O, 0.05 g L^−1^ Ca(NO_3_)_2_ × 4 H_2_O, 10 mg L^−1^ ammonium iron(III) citrate, a trace element and vitamin solution) (Margesin and Schinner 1997) containing glucose (2 g L^−1^). After 3 days at 20 °C and 150 rpm the biomass was centrifuged (10 000 × g, 10 min), washed twice with sterile MM and resuspended in MM. 20 mL MM containing (i) 2 g L^−1^ glucose (G1-3) or (ii) 10 mM trans-FA (Sigma-Aldrich 128708, FA1-3) were prepared. These were inoculated with the washed biomass at an initial (t0) optical density at 600 nm (OD600) of 0.05 in triplicates. The cultures were incubated at 20 °C and 150 rpm.

To determine the exponential phase, growth curves at the above described conditions were studied in pre-tests. Cells were harvested on ice in their exponential phase: G1-3 after 18 h and FA1-3 after 96 h. Approximately 5 × 10^8^ cells were centrifuged (4 °C, 10 000 × *g*) and washed twice with sterile MM. The supernatant was discarded, and 1 mL Nucleo Protect (Macherey–Nagel, Düren, Germany) was added before storing in liquid nitrogen. Lysozyme (400 µL, 10 mg mL^−1^, Sigma-Aldrich, USA) was added to each sample followed by beat beating with a FastPrep instrument (6 m s^−1^, 30 s, MP Biomedicals, Irvine, CA, USA) in 2 mL tubes containing glass beads (0.5 mm diameter, BioSpec Products, Inc., Bartlesville, OK, USA). RNA was further extracted using the RneasyMini Kit (Qiagen, Hilden, Germany) following the manufacturer’s recommendations. Quality and quantity of the extracted RNA were measured with a Nanodrop instrument (Thermo Fisher, Waltham, MA, USA). DNA was further digested with DNase I (Thermo Fisher, Waltham, MA, USA) and after the enzyme reaction cleaned using Monarch RNA Cleanup Kit (New England Biolabs, Ipswich, MA, USA). The RNA quality was determined using the Agilent Bioanalyzer 2100 Pico kit (Agilent Technologies, Santa Clara, CA, USA) and the QuantiFluor RNA kit (Quantus, Promega, Madison, WI, USA).

### Sequencing

Libraries were prepared and sequencing was done at Novogene, Cambridge. Library was prepared using 1 µg RNA input and the Illumina Ribo-Zero Plus rRNA Depletion Kit (Illumina, San Diego, CA, USA) followed by NEBNext® UltraTM RNA Library Prep Kit for Illumina (New England Biolabs, Ipswich, MA, USA) following the manufacturer’s recommendation. Quality assessment was done with Agilent Bioanalyzer 2100 (Agilent Technologies, Santa Clara, CA, USA). Libraries were clustered using the PE Cluster Kit cBot-HS (Illumina, San Diego, CA, USA) and were further sequenced on an Illumina NovaSeq 6000.

### Sequencing data analysis

Raw data processing was done at Novogene Cambridge. Raw reads were processed using fastp (Chen et al. 2018) and cleaned reads were mapped against the reference genomes using Bowtie2 (Langemead and Salzberg 2012, reference genomes: previously published genome of *P. aromaticivorans* AR20-38 (Poyntner et al. 2020; NCBI, PRJNA624061) and genome of *P. phytofirmans* (NCBI, PRJNA17463)). FeatureCounts (Liao et al. 2014) was used for counting the reads followed by calculation of fragments per kilobase of transcript per million mapped reads (FPKM). The differential gene expression analysis was performed using DESeq2 R package (Love et al. 2014). Genes with resulting *p*-values < 0.05 were assigned as differentially expressed. Gene length biases were corrected using ClusterProfilerR (Yu et al. 2012) and the gene Ontology (GO) enrichment (Ashburner et al. 2000) and Kyoto encyclopedia of genes and genomes (KEGG, Kanehisa and Goto 2000) pathway analysis were performed. Data was sorted and duplicated reads were marked using Picard (http://broadinstitute.github.io/picard accessed 01.09.2021) and Samtools (Li et al. 2009). Further analysis was done using R studio version 4.1.3(R Core Team 2017) using the package ggplot (Wickham 2016). Inkscape (Inkscape Project 2020) was used for graph creation.

### HPLC analysis

FA and VA were quantified with HPLC analysis as described previously (Margesin et al. 2021) in reducing intervals (28–8 h intervals, Additional file 1: Table S1). In short: after centrifugation (10 min, 20 000 × *g*) to remove larger particles the supernatants were frozen (− 20 °C) and at least 0.7 mL of the supernatant was filtered (0.2 µm RC filter) for HPLC measurement. The measurement was performed at 70 °C using a Shimadzu Prominence system equipped with a RFQ Fast Acid column (50 × 7.8 mm, Phenomenex, Torrance, CA, Germany) and a mobile phase of 5 mM sulfuric acid as described previously (Wagner et al. 2017). A UV detection at 220 nm combined with a crosscheck at 270 nm was applied. The calibration was performed via injection of 1, 5 and 10 mM FA and VA external standards.

The rate constant of decline (k), DT50 was calculated using Computer Assisted Kinetic Evaluation (CAKE) software (available online with public free access, https://cake-kinetics.org/, accessed: 21.04.2022) using a convergence tolerance of 1 × 10^–5^, 100 max. iterations and iteratively reweighted least squares (max. reweightings: 200, error variance tolerance: 1 × 10^10^) and a simple first order fit.

## Results

**Fig. 1:**
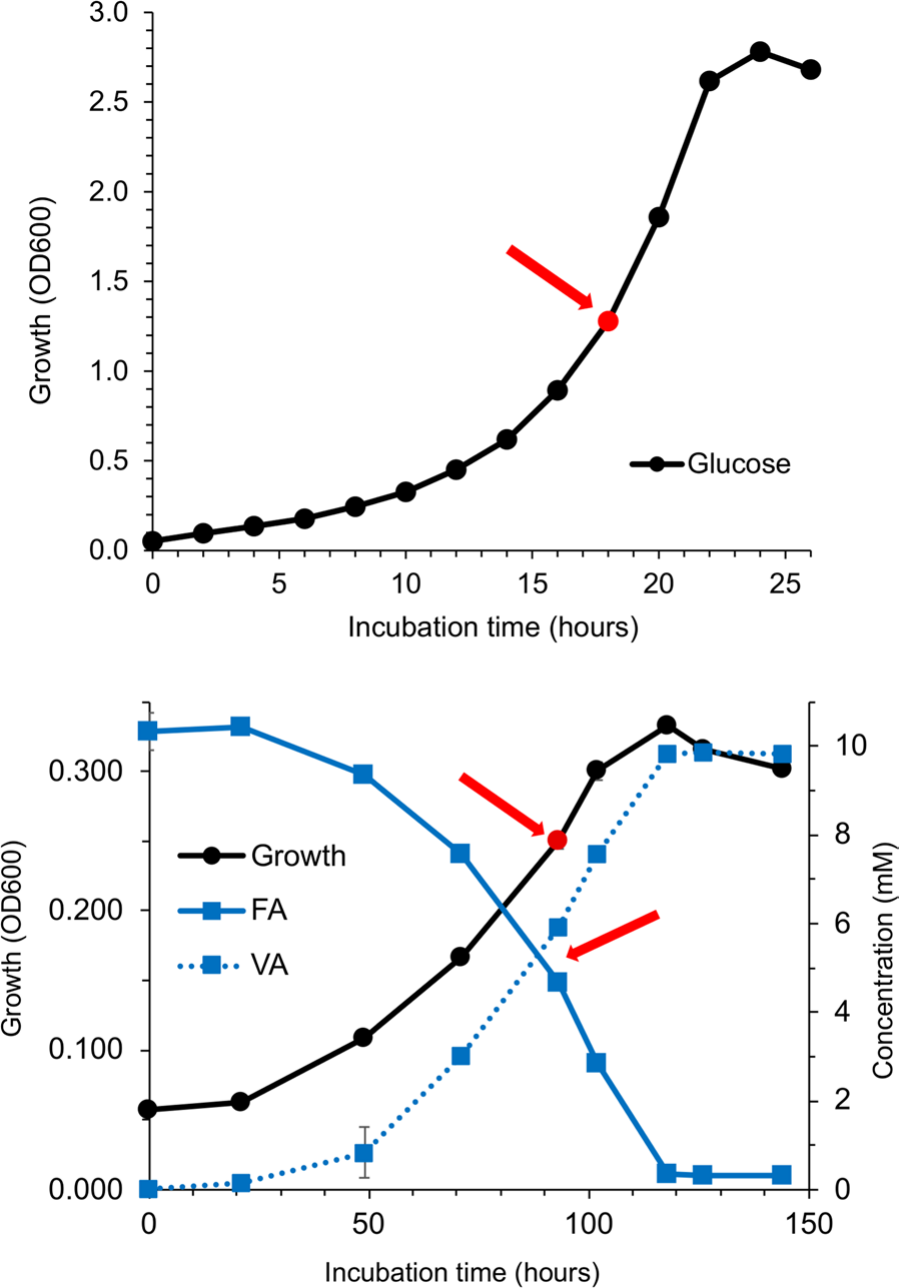
Growth curves of *P. aromaticivorans* AR20-38 based on OD600 values and HPLC values. Upper panel: cultivation with glucose as sole carbon source; Lower panel: cultivation with ferulic acid as sole carbon source. HPLC results of ferulic acid depletion and vanillic acid production are marked in blue. Red points and arrows mark the timepoints of biomass harvesting. Values plotted are the mean of triplicates from independent cultures, ± standard deviation

The strain *P. aromaticivorans* AR20-38 exhibited very good bioconversion capability of FA to VA at low and moderate temperatures (10–30 °C) (Berger et al. 2021; Margesin et al. 2021) but the gene expression patterns during the bioconversion of FA to VA remained unclear. To close this knowledge gap, two experiments were set up in the present study: the strain was cultivated with (i) glucose (G1-3) and (ii) FA (FA1-3) as sole carbon source in triplicates at 20 °C. In the exponential growth phase (after 18 h of cultivation for glucose and after 96 h for FA, Fig. 1) the cells were harvested, and RNA was extracted and sequenced. The strain exhibited a rate constant of decline for FA (k) of 0.63 (σ: 0.0031; r^2^: 0.8) and a DT50 of 55.5 h.

### Transcriptome sequencing

The cDNA sequencing of the samples yielded in 15.3–10.8 million raw reads (Additional file 1: Table S2). After removing reads with adaptor sequence contamination, uncertain nucleotides and low-quality nucleotides 15.2–10.8 million clean reads were obtained. The base error rate of all samples was 0.02%. The mapping resulted in of 12 to 8 million uniquely mapped reads. In the principal component analysis (PCA) analysis (Additional file 1: Fig. S1) the three biological replicates from three independent cultures of the two conditions (G, FA) clustered well together, and the sequenced samples G1-3 and FA1-3 were clearly separated. Therein, G1-3 replicates were very similar and FA1-2 differed slightly from FA3.

In total 5049 genes were expressed, 306 were only expressed in the samples with FA and 59 solely with glucose as sole carbon source. 1653 genes were upregulated in the FA samples compared to 1682 downregulated genes.

### Transcriptional response to ferulic acid

**Fig. 2:**
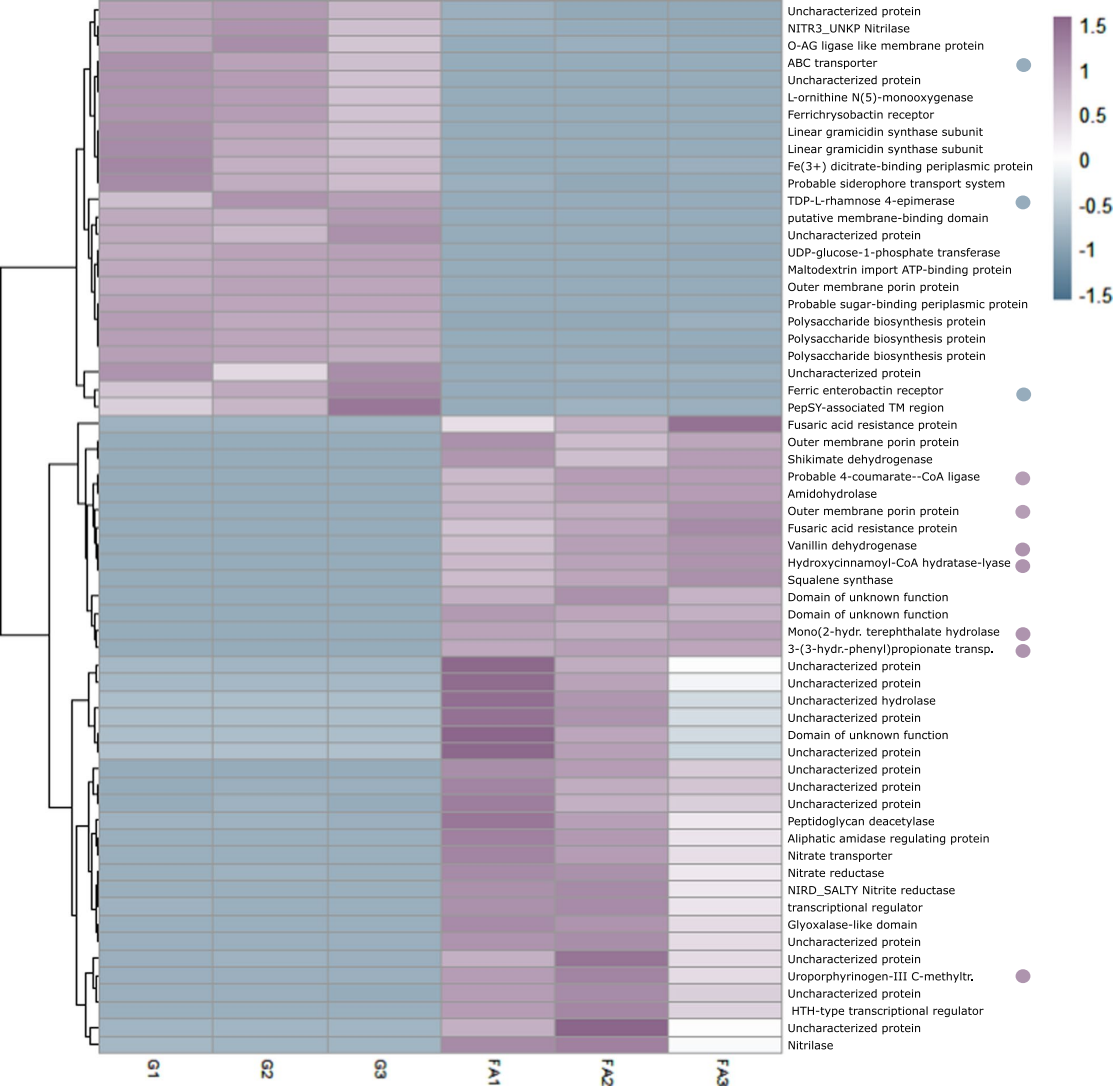
Heatmap of expressed transcripts of the biological triplicates. Biological replicates cultivated with glucose as sole carbon source are marked with G1-3 (left columns) and biological replicates cultivated with ferulic acid as sole carbon source with FA1-3 (right columns). Values presented are fold change > 50. Upregulation is marked in violet (values 0 − 1.5) and downregulation is marked with grey (values 0 − (− 1.5)). Gene names are listed on the right, dots mark genes involved in FA bioconversion

The highest differentially expressed genes clustered well within the triplicates and transcripts reflect the activities associated with FA as sole carbon source (Fig. 2). Genes coding for enzymes involved in bioconversion of FA were within the ten most highly upregulated genes (*p* < 0.05, Additional file 1: Table S3) when *P. aromaticivorans* was cultivated with FA as sole carbon source*.* Three genes directly involved in the bioconversion were determined: 4-coumarate-CoA ligase (RS20035, log_2_FC:89), hydroxycinnamoyl-CoA hydratase lyase (RS20025, log_2_FC: 9.6) and vanillin dehydrogenase (RS20030, log_2_FC:9.1).

Mono(2-hydroxyethyl) terephthalate hydrolase (RS20005, log_2_FC:11.36) was within the ten most upregulated genes. This enzyme was previously described in *Ideonella sakaiensis* (Yoshida et al. 2016) and hydrolyzes polyethylene terephthalate (PET)-derived mono(2-hydroxyethyl) terephthalate to terephthalic acid and ethylene glycol (Knott et al. 2020). The second enzyme responsible for PET degradation in *I. sakaiensis,* the PETase, was not detected in *P. aromaticivorans*.

The 3-(3-hydroxy-phenyl)propionate transporter was highly upregulated (RS20015, log_2_FC:11.7) as well as the outer membrane porin protein BP0840 (RS20010, log_2_FC: 11.5), important for transports.

Uroporphyrinogen-III *C*-methyltransferase (RS09835, log_2_FC:11.5) was upregulated and is involved in the cobalamin biosynthesis. Similarly, in a transcriptome study on lignin valorisation (Zhu et al. 2021) cobalt-precorrin-3B C(17)-methyltransferase, which is involved in the adenosyl cobalamin biosynthesis, was upregulated.

The NIRD_SALTY nitrite reductase (NADH) small subunit (RS09845/ RS09840, log2FC: 11.3/9.6) and MFS_1 nitrate transporter (RS09855, log_2_FC: 7.17) were highly upregulated in *P. aromaticivorans* cultivated with FA as sole carbon source.

In other bacterial strains the bioconversion of VA to protocatechuic acid is catalyzed by the enzyme vanillate *O*-demethylase consisting of two subunits: VanA oxygenase and VanB oxidoreductase. Here, the gene for VanA was upregulated (RS34940, log_2_FC: 1.9) whereas VanB was downregulated (RS10660, log_2_FC: − 1.1).

In *P. aromaticivorans* cultivated with FA, the highest downregulated gene was dTDP-L-rhamnose 4-epimerase (RS31050, log_2_FC: − 9.4), involved in the interconversion of dTDP-rhamnose to dTDP-6d talose. Addtionally, the ABC transporter for syringomycin transport (RS20255, log_2_FC: − 8.7) was downregulated in *P. aromaticivorans* in presence of FA.

*P. aromaticivorans* downregulated the ferric enterobactin receptor (RS22965, log_2_FC: − 8.3) in presence of FA compared to glucose as sole carbon source. The cultivation medium for both conditions contained 0.1 mg l^−1^ iron respectively.

Additional iron related genes were differently expressed, three down and three slightly upregulated: the ferric iron reductase (RS20240, log_2_FC: − 4.7), ferrous iron permease (RS06920, log_2_FC: − 4.7), iron sulfur cluster assembly protein CyaY (RS17920, log_2_FC: − 0.8) were downregulated and the probable iron transporter (RS09245, log2FC:0.6), iron-sulfur cluster carrier protein (RS04965, log_2_FC:0.4), iron-sulfur cluster assembly scaffold protein (RS12775, log_2_FC: 0.03) were slightly upregulated.

### Transporters and transcriptional factors

The data was screened based on transcriptional factors reported by Tropel and van der Meer (2004) and their importance for the degradation pathways of aromatic compounds. *P. aromaticivorans* differentially expressed 134 genes in the LysR group, 47 up and 87 down. Within the group, CatR was upregulated (RS10680, log_2_FC: 0.1), a transcriptional factor involved in the catechol pathway. Further, BenM (RS07845, log_2_FC: 1.0), involved in the benzoate (catechol) pathway and two genes of NahR, both involved in the naphthalene and salicylate pathway (RS30310/ RS21410, log2FC: − 0.7/− 0.1), were downregulated. Twenty-one IclR genes were differentially expressed, eight down and 13 up. Within the IclR group, PcaU (RS18385, log_2_FC: 1.9) and two genes coding for PCAR (RS13595/RS29425, log_2_FC: − 1.3/0.2) were differentially expressed.

Twenty-three MarR genes (9 up, 14 down) and 26 TetR genes (14 down, 12 up) were differentially expressed. These transcriptional factors regulate aromatic compounds transporters.

In the transcriptome of *P. aromaticivorans* 336 transporters were differentially expressed. Of these transporters, 161 are categorized as ATP-binding cassette (ABC) transporters and as 96 major facilitator superfamily (MFS) transporters. The ABC transporter spermidine/putrescine was highly upregulated (RS26120, log_2_FC: 5.6). This transporter is responsible for transporting polyamines which are important for cell proliferation and for ion homeostasis. The upregulated sugar transporter (RS26045, log_2_FC: 5.4) belongs to the ABC group which was reported in *Thermobifida fusca* (Adav et al. 2012) cultivated on different lignocellulosic materials e.g. hay, saw dust, wood chip, un-dried plant biomass. The shikimate transporter (RS20435, log_2_FC: 5.2) was upregulated, transporting shikimate, an intermediate product in the aromatic compound biosynthetic pathway (Kubota et al. 2015). The second most important group of transporters are the MFS group. These transporters are reported to be involved in FA transport in other bacteria (Winkler and Kao 2011). Two MFS transporters were within the ten highest differentially expressed genes: 3-(3-hydroxy-phenyl) propionate transporter and MFS_1 nitrate transporter (see Sect. Transcriptional response to ferulic acid).

*P. aromaticivorans* upregulated the maltodextrin import in the experiment with glucose compared to FA, used to import glucose (RS05040, log_2_FC: − 5.9). Further, a probable siderophore transport system (RS20230, − log_2_FC: − 5.7) was downregulated in the FA experiment.

### Enrichment KEGG Terms

**Fig. 3:**
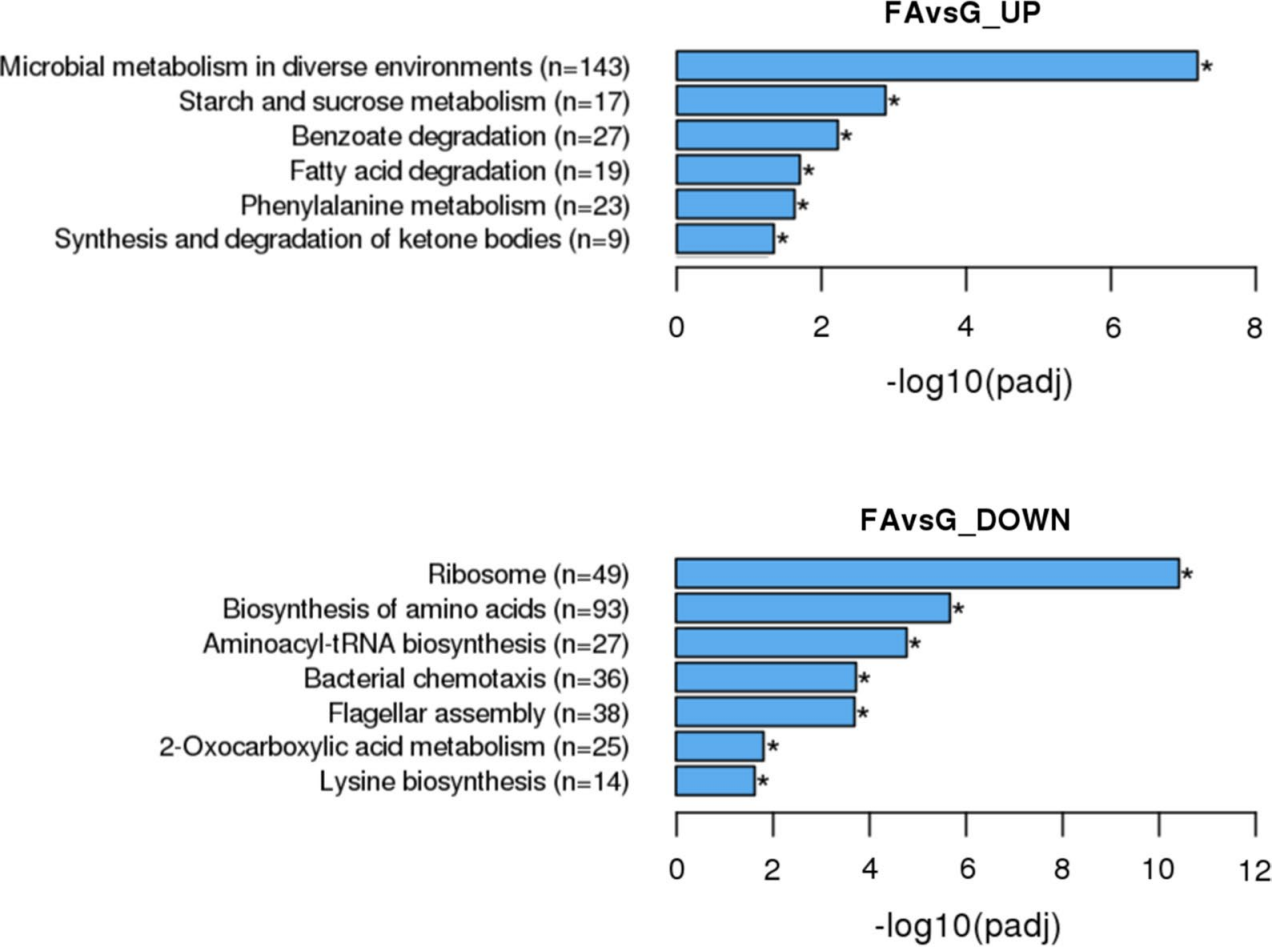
Significantly up- and downregulated KEGG terms. The upper panel represents the upregulated terms and the lower panel the downregulated terms of *P. aromaticivorans* cultivated with ferulic acid in comparison to glucose as sole carbon source. Bars represent the -log10 adjusted *p*-value

In the cultivation with FA as sole carbon source, *P. aromaticivorans* upregulated genes of the KEGG term (Fig. 3, Additional file 1: Table S5) microbial metabolism in diverse environments (bpy01120) and benzoate degradation (bpy00362,  Fig. 3, Additional file 1: Table S5). Both terms contain pathways involving ring cleaving enzymes. In presence of FA* P. aromaticivorans* upregulated the term starch and sucrose metabolism (bpy00500) to increase the carbohydrate metabolism. The upregulated term fatty acid degradation (bpy00071) contains fatty acid oxidation enzymes such as enoyl-SCoA hydratase. The term phenylalanine metabolism (bpy00360) was upregulated comprising the modules phenylacetate degradation and trans-cinnamate degradation. In both modules, enzymes involved lignin and FA biodegradation are found. Nine genes fit in the term synthesis and degradation of ketone bodies (bpy00072).

KEGG terms related to growth were upregulated in the cultivation of *P. aromaticivorans* with glucose as sole carbon source whereas in the cultivation with FA as sole carbon source these terms were downregulated: Ribosome (bpy03010), biosynthesis of amino acids (bpy01230) and aminoacyl-tRNA biosynthesis (bpy00970). Further bacterial chemotaxis (bpy02030), flagellar assembly (bpy02040), 2-oxocarboxylic acid metabolism (bpy01210) and lysine biosynthesis (bpy00300) were downregulated in presence of FA﻿.

## Discussion

**Fig. 4:**
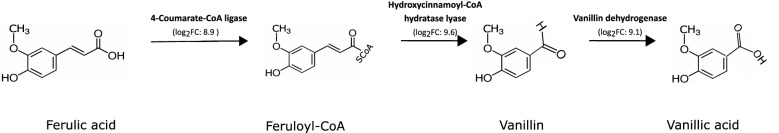
In this study proposed bioconversion pathway of ferulic acid to vanillic acid for *P. aromaticivorans* AR20-38 based on the differentially expressed genes. The chemical structures of ferulic acid, feruloyl CoA, vanillin and vanillic acid are depicted. Putative involved enzymes and their log_2_FC values are shown above the arrows

Based on the strongly differentially expressed genes in the transcriptome of *P. aromaticivorans* during bioconversion of FA to VA in this study, the pathway depicted in Fig. 4 is proposed. 4-coumarate-CoA ligase is a known enzyme in FA metabolism responsible for the step FA to feruloyl-CoA and was previously reported in *Streptomyces coelicolor* (Kaneko et al. 2003) and in plants (Wohl and Petersen 2020). Subsequently, hydroxycinnamoyl-CoA hydratase lyase converts ferulolyl-CoA to vanillin, a coenzyme A dependent deacetylase, which was previously reported in *Pseudomonas fluorescens* (Bennett et al. 2008). Vanillin dehydrogenase is a known enzyme for Vanillin metabolization to VA. *P. aromaticivorans* was not able to further degrade VA through the protocatechuate pathway and bioaccumulated VA (Margesin et al. 2021). In other bacteria, this step is catalyzed by the enzyme vanillate *O-*demethylase encoded by the genes VanA and VanB, which were only slightly regulated in *P. aromaticivorans, VanB* was downregulated. In contrast, PcaU was slightly upregulated, a transcriptional factor reported to play a role in the protocatechuate pathway. *P. aromaticivorans* harbors the gene for protocatechuate-3,4-dioxygenase (Poyntner et al. 2021) but the gene was not upregulated during FA bioconversion (Additional file 1: Table S3). This indicates that PcaU is involved in the previous steps of FA to VA bioconversion.

The upregulated KEGG terms can be related to FA bioconversion. The main upregulated KEEG terms involve pathways with ring cleaving enzymes, which are important for the bioconversion of FA to VA. For example, the KEGG term microbial metabolism in diverse environments comprises various pathways including carbohydrate metabolism, energy metabolism, sulfur- and amino acid metabolism, as well as xenobiotic degradation. Further,  the upregulated KEGG term benzoate degradation can be related to the frequently reported ability to degrade benzoate in *Paraburkholderia* strains (Donoso et al. 2016; Herpell et al. 2021; Vanwijnsberghe et al. 2021). The upregulated term fatty acid degradation contains fatty acid oxidation enzymes such as enoyl-SCoA hydratase. These are reported to be involved in the degradation of FA (Gasson et al. 1998). The upregulated KEGG term phenylalanine metabolism could be linked to osmotic stress response as previously reported in *Burkholderia cenocepacia* (Behrends et al. 2011) but also comprises enzymes involved lignin and FA biodegradation.

The highly regulated mono(2-hydroxyethyl) terephthalate hydrolase was previously reported in *I. sakaiensis* (Yoshida et al. 2016) during PET degradation. *I. sakaiensis* did not show any activity against ethyl ferulate (Yoshida et al. 2016). Although the gene coding for PETase is missing the genome and transcriptome of *P. aromaticivorans*, the strain showed growth in presence of bisphenol A, a chemical plasticizer, in previous tests (data not shown). A closely related species, *B. xenovorans*, was reported to be an effective polychlorinated biphenyl degrader (Chain et al. 2006). Additionally, the KEGG term lysine biosynthesis was regulated, which was detected in *B. xenovorans* (Parnell et al. 2006) in presence of polychlorinated biphenyls. Therefore, *P. aromaticivorans* might be a good candidate for plastic but also xenobiotic degradation. In future studies, the involvement of mono(2-hydroxyethyl) terephthalate hydrolase in FA bioconversion needs to be studied in detail which could be also important for other FA bioconverting organisms.

Uroporphyrinogen-III *C*-methyltransferase was highly regulated, which is involved in cobalamin biosynthesis. Similary, cobalt-precorrin-3B *C*(17)-methyltransferase was upregulated in a transcriptome study on lignin valorisation (Zhu et al. 2021). This enzyme is involved in the adenosyl cobalamin biosynthesis. Cobalamin produced by *Rhizobium* isolates from forest soils were shown to enhance methane oxidation of a methanotroph (Iguchi et al. 2011). A similar mechanism might play a role in *P. aromaticivorans*.

The most regulated transporters in the transcriptome of *P. aromaticivorans* during bioconversion of FA were ABC transporters and MFS transporters. ABC transporters are reported in *Enterobacter lignolyticus* (DeAngelis et al. 2013)*, Clostridium beijerinckii* (Lee et al. 2015; Zhang and Ezeji 2013) and *Lactobacillus brevis* (Winkler and Kao 2011) to be involved in the transport of lignin-derived aromatic substances such as FA. The regulated ABC sugar transporter and spermidine/putrescine might be used for nutrient acquisition in *P. aromaticivorans* during FA bioconversion.

Within the MFS group, the MFS_1 nitrate transporter was highly regulated, indicating utilization of nitrate. This is in contrast to previously reported results detecting no nitrate reduction capability in the strain *P. aromaticivorans* BNT5^T^ (Lee and Jeon 2018). Additionally, the NIRD_SALTY nitrite reductase small subunit was highly upregulated. It was previously shown in denitrifying bacteria that the addition of nitrate improved the biodegradation processes of toluene as nitrate can be used as an alternative electron acceptor (Leahy and Olsen 1997).

Another putative transport mechanisms during FA bioconversion is the upregulated 3-(3-hydroxy-phenyl)propionate transporter, which also belongs to the MFS group. Phenylpropionate and its hydroxylated derivatives are degradation products of lignin and were shown to be degraded by various bacteria (Arai et al. 1999; Barnes et al. 1997; Burlingame and Chapman 1983; Dagley et al. 1965; Pérez-Pantoja et al. 2008; Xu et al. 2013). The regulated transcription factors MarR and TetR are known regulators of aromatic compounds transporters for degradation pathways including HpcR involved in the hydroxycinnamate pathway (Tropel and van der Meer 2004). Thus, this indicates an involvement of these transcription factors for transporting mechanisms in *P. aromaticivorans* during bioconversion of FA. Although many transcriptional factors are annotated in the genome *of P. aromaticivorans*, only a few of the highly differentially expressed transcriptional factors could be related to previously reported involvement in aromatic compound degradation. The targets of the differentially expressed transcription factors in this study still need to be studied.

The enzymes for catabolizing FA might be transported through the highly regulated outer membrane porin protein. This is in line with the upregulation of the GO term transmembrane transport (Additional file 1: Table S4). *P. aromaticivorans* might use a mechanism based on outer membrane vesicles (OMVs) as reported by Salvachúa et al. (2020) in *P. putida*. OMVs in *P. putida* are used to catabolize lignin-derived substances which are transported via porin proteins. The utilization of OMVs has also been suggested as a synthetic biology tool and the OMV model harbors several potential biotechnological applications (Salvachúa et al. 2020).

Several downregulated genes during bioconversion of FA indicate plant interaction capabilities of *P. aromaticivorans*. *Burkholderia* species were previously reported to act as plant promoting (Herpell et al. 2021; Donoso et al. 2016). The ABC transporter for syringomycin was downregulated, reported as a potential plant virulence factor in *B. thailandesis* (Kovacs-Simon et al. 2019). The downregulation of the KEGG term chemotaxis indicates potential plant interaction capabilities. Sheibani-Tezerji et al. (2015) reported chemotaxis in the transcriptome of the endophyte *B. phytophormans* and Balsanelli et al. (2016) showed that chemotaxis plays an important role in initial plant contact. Further, due to the higher growth rate with glucose compared to FA, *P. aromaticivorans* needed more cell wall components and therefore might upregulated dTDP-L-rhamnose 4-epimerase in presence of glucose. This epimerase is involved in the production of L-rhammnose, a polysaccharide component of pathogenic or plant associated bacteria (Graninger et al. 1999). Additionally, the epimerase is involved in the interconversion to dTDP-6-deoxy-D-talose, which was shown in *B. thailandensis* (Yoo et al. 2011) to serve as building block for O-antigenic polysaccharide biosynthesis. The O-antigenic polysaccharide is a reported virulence factor in *Burkholderia pseudomallei* (DeShazer et al. 1998)*.* In presence of glucose, enterobactin, an iron siderophore, ferric iron reductase, ferrous iron permease and probable siderophore transport system were upregulated. This indicates the importance of iron during high growth rates but not during bioconversion of FA. Catechol-like siderophore production was previously reported from rhizospheric bacteria (Joshi et al. 2006) which gives them advantage to sequester iron from soil.

The downregulated KEGG terms related to growth fit well with the higher growth rate with glucose as sole carbon source in comparison to FA. The regulated term 2-oxocarboxylic acid metabolism might be involved in the glucose metabolism in the cultivation of *P. aromaticivorans* with glucose as sole carbon source. The upregulated KEGG term synthesis and degradation of ketone bodies cannot be related to any *P. aromaticivorans* metabolism.

In conclusion, the transcriptional profile of *P. aromaticivorans* during bioconversion of FA to VA elucidated the expressed genes involved in the bioconversion process. These genes were clustered within the ten most highly differentially expressed genes. These enzymes and the transcripts known from aromatic compound degradation and synthetic polymer degradation offer new gene targets for bioconversion optimization and metabolic engineering. In addition, the strong expression of an outer membrane porin was detected, indicating towards the occurrence of a recently proposed outer membrane vesicle mechanisms for bacterial lignin catabolism (Salvachúa et al. 2020). To confirm this hypothesis, further studies on exosomes using e.g. exoproteome experiments are needed. These could offer a number of new applications for synthetic biology that may even lead to novel biotechnological applications.

Overall, the strain AR20-38 is a promising candidate not only for FA bioconversion applications but also for xenobiotic and synthetic polymer degradation, especially in moderate and cold temperature environments.

## Supplementary Information

**Additional file 1: Figure S1.** PCA analysis. The RNA-sequencing results of the 3 biological replicates cultivated with glucose (G1-3) or ferulic acid (FA1-3) are depicted. **Table S1.** OD600 and HPLC values. **Table S2.** Sequencing statistics. **Table S3.** Gene expression values. **Table S4.** Regulated GO terms. **Table S5.** Regulated KEGG terms.

## Data Availability

Rawdata (Fastq files) of the sequenced samples were deposited in the NCBI Sequencing Read Archive under the numbers SRR18913740, SRR18913739, SRR18913740, SRR18913578, SRR18913579 and SRR18913580 within the Bioproject PRJNA624061.
