# Supplementary material for: Transcriptome profiling of Paraburkholderia aromaticivorans AR20-38 during ferulic acid bioconversion
Source: AMB Express. 2022 Nov 26;12:148. doi: 10.1186/s13568-022-01487-7 (PMC9701309; doi:10.1186/s13568-022-01487-7)
Supplement: Supplementary file 1 — Additional file 1: Figure S1. PCA analysis. The RNA-sequencing results of the 3 biological replicates cultivated with glucose (G1-3) or ferulic acid (FA1-3) are depicted. Table S1. OD600 and HPLC values. Table S2. Sequencing statistics. Table S3. Gene expression values. Table S4. Regulated GO terms. Table S5. Regulated KEGG terms. [file 13568_2022_1487_MOESM1_ESM.docx]

Additional file 1

AMB Express

Transcriptome profiling of *Paraburkholderia aromaticivorans* AR20-38 during ferulic acid bioconversion

Caroline Poyntner^a,*^, Thomas Marek Ludwikowski^a^, Andreas Otto Wagner^a^ , Rosa Margesin^a^

^a^Department of Microbiology, University of Innsbruck, Technikerstraße 25, 6020 Innsbruck, Austria

*Corresponding author: [caroline.poyntner@uibk.ac.at](mailto:caroline.poyntner@uibk.ac.at)


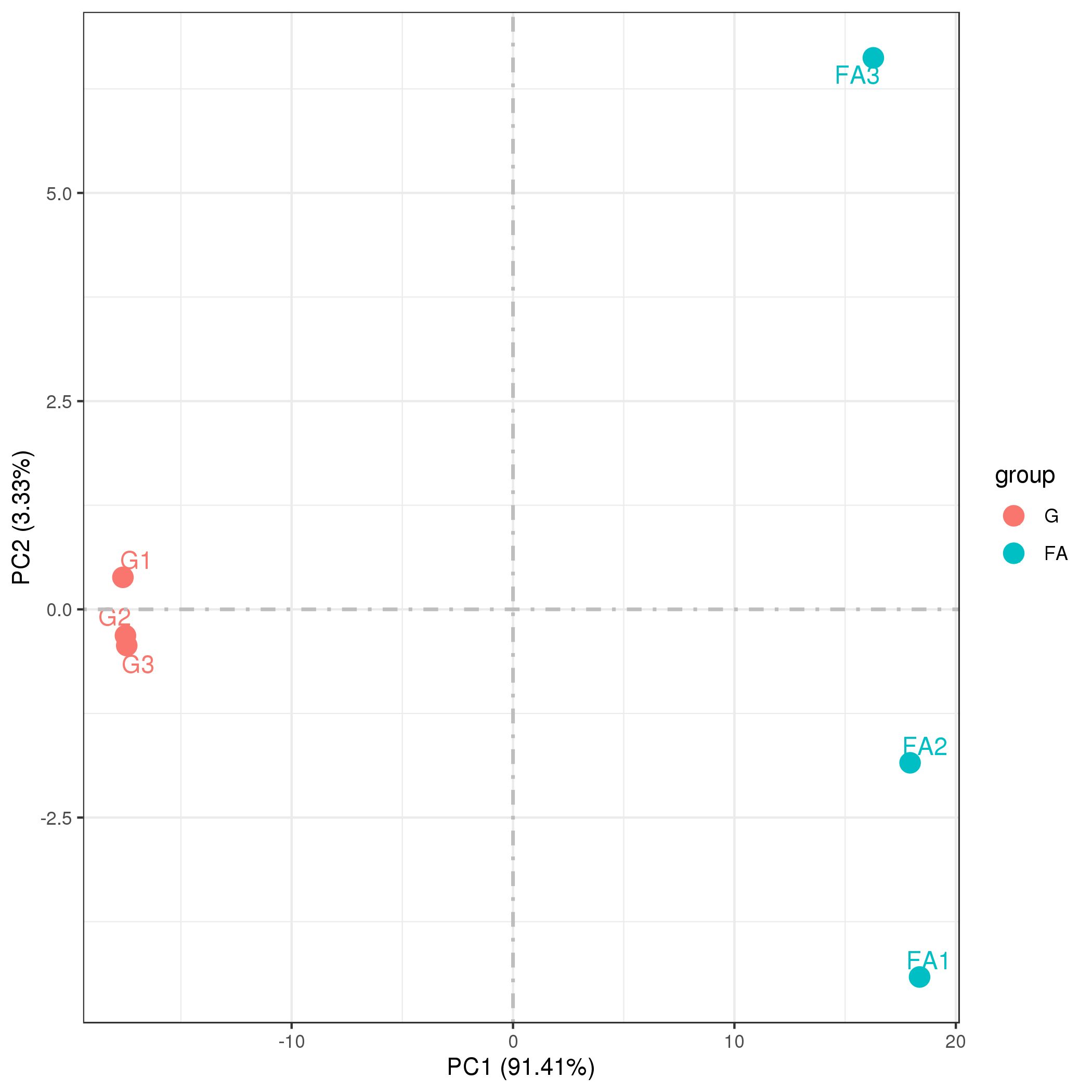


Figure S1: PCA analysis. The RNA-sequencing results of the 3 biological replicates cultivated with glucose (G1-3) or ferulic acid (FA1-3) are depicted.

Table S1: OD600 and HPLC values

|  |  | **OD600** |  | **mM FA Rest** |  | **mM VA Konz** |  |
| --- | --- | --- | --- | --- | --- | --- | --- |
| **sample names** | **h** | **MW** | **SD** | **MW** | **SD** | **MW** | **SD** |
| G 1-3 | 0 | 0.052 | 0.000 | na | na | na | na |
| G 1-3 | 2 | 0.096 | 0.001 | na | na | na | na |
| G 1-3 | 4 | 0.135 | 0.002 | na | na | na | na |
| G 1-3 | 6 | 0.177 | 0.002 | na | na | na | na |
| G 1-3 | 8 | 0.245 | 0.002 | na | na | na | na |
| G 1-3 | 10 | 0.326 | 0.001 | na | na | na | na |
| G 1-3 | 12 | 0.451 | 0.002 | na | na | na | na |
| G 1-3 | 14 | 0.619 | 0.004 | na | na | na | na |
| G 1-3 | 16 | 0.894 | 0.001 | na | na | na | na |
| G 1-3 | 18 | 1.278 | 0.018 | na | na | na | na |
| G 1-3 | 20 | 1.858 | 0.018 | na | na | na | na |
| G 1-3 | 22 | 2.617 | 0.039 | na | na | na | na |
| G 1-3 | 24 | 2.780 | 0.008 | na | na | na | na |
| G 1-3 | 26 | 2.680 | 0.014 | na | na | na | na |
| FA1-3 | 0 | 0.058 | 0.000 | 10.3 | 0.4 | 0.0 | 0.0 |
| FA1-3 | 21 | 0.063 | 0.000 | 10.4 | 0.1 | 0.1 | 0.0 |
| FA1-3 | 49 | 0.10833333 | 0.002 | 9.4 | 0.0 | 0.8 | 0.6 |
| FA1-3 | 71 | 0.16666667 | 0.004 | 7.6 | 0.1 | 3.0 | 0.1 |
| FA1-3 | 93 | 0.25033333 | 0.005 | 4.7 | 0.1 | 5.9 | 0.1 |
| FA1-3 | 102 | 0.300 | 0.006 | 2.8 | 0.1 | 7.6 | 0.1 |
| FA1-3 | 118 | 0.333 | 0.001 | 0.4 | 0.0 | 9.8 | 0.0 |
| FA1-3 | 126 | 0.315 | 0.002 | 0.3 | 0.0 | 9.8 | 0.0 |
| FA1-3 | 144 | 0.302 | 0.004 | 0.3 | 0.0 | 9.8 | 0.0 |

Table S2: Sequencing statistics

| **Sample names** | **Raw reads** | **Clean reads** | **Mapped reads** | **Uniquely mapped reads** |
| --- | --- | --- | --- | --- |
| G1 | 13572037 | 13463962 | 22580426 | 11602710 |
| G2 | 10875685 | 10799524 | 18116184 | 9322677 |
| G3 | 14791408 | 14666093 | 24656993 | 12579110 |
| FA1 | 15357819 | 15237718 | 23289756 | 11452348 |
| FA2 | 11796770 | 11668468 | 17838542 | 8613141 |
| FA3 | 12146316 | 11813105 | 17968555 | 8444773 |

| Table S3: Gene expression values | | |  |  |  |  |  |  |  |
| --- | --- | --- | --- | --- | --- | --- | --- | --- | --- |
| **Gene_ID** | **FA1** | **FA2** | **FA3** | **G1** | **G2** | **G3** | **log2FoldChange** | ***p*-value** | ***p-*adj** |
| gene-BPHYT_RS03900 | 808.480095 | 911.047841 | 874.378219 | 72237.8716 | 72189.7861 | 78812.9706 | -6.42847122 | 0 | 0 |
| gene-BPHYT_RS05025 | 5297.81922 | 5479.08865 | 5841.04985 | 543943.305 | 549057.536 | 538777.418 | -6.6176796 | 0 | 0 |
| gene-BPHYT_RS05030 | 758.997601 | 802.234164 | 720.853671 | 33370.0593 | 32698.3423 | 30995.5434 | -5.41074314 | 0 | 0 |
| gene-BPHYT_RS05035 | 567.452465 | 580.339608 | 555.128497 | 28965.7439 | 29163.4874 | 27708.1235 | -5.65562484 | 0 | 0 |
| gene-BPHYT_RS05040 | 986.45745 | 1155.34521 | 1439.67391 | 70246.2179 | 73069.4504 | 72294.2643 | -5.91280943 | 0 | 0 |
| gene-BPHYT_RS05490 | 5059.1859 | 4284.27181 | 4666.73956 | 574020.234 | 615522.029 | 588706.182 | -6.98754136 | 0 | 0 |
| gene-BPHYT_RS06910 | 1898.69116 | 1653.54116 | 1762.9905 | 27910.759 | 28913.0448 | 26484.0553 | -3.96956809 | 0 | 0 |
| gene-BPHYT_RS15280 | 130217.172 | 109469.759 | 121315.911 | 351.004317 | 336.415518 | 299.679417 | 8.51619858 | 0 | 0 |
| gene-BPHYT_RS17360 | 14709.0702 | 16233.507 | 16152.6125 | 879.482727 | 991.802785 | 909.902762 | 4.08289768 | 0 | 0 |
| gene-BPHYT_RS19735 | 14219.832 | 10995.5153 | 10481.3547 | 272.126942 | 235.490862 | 255.315999 | 5.5461481 | 0 | 0 |
| gene-BPHYT_RS20005 | 59596.0766 | 60448.1309 | 63409.7051 | 25.6351467 | 22.4277012 | 21.7290212 | 11.3592397 | 0 | 0 |
| gene-BPHYT_RS20010 | 68245.9357 | 74713.3905 | 84064.3487 | 17.7474093 | 39.8714688 | 22.6343971 | 11.5124758 | 0 | 0 |
| gene-BPHYT_RS20015 | 29616.0704 | 33374.8615 | 31290.5396 | 10.845639 | 9.96786719 | 8.14838295 | 11.678146 | 0 | 0 |
| gene-BPHYT_RS20025 | 219535.467 | 266189.19 | 283236.523 | 355.934153 | 343.891418 | 300.584793 | 9.58780444 | 0 | 0 |
| gene-BPHYT_RS20030 | 192012.825 | 248004.504 | 258247.607 | 391.428971 | 434.848206 | 440.918055 | 9.10628417 | 0 | 0 |
| gene-BPHYT_RS20035 | 91444.446 | 116432.768 | 114015.87 | 218.884715 | 223.031028 | 219.100964 | 8.9281712 | 0 | 0 |
| gene-BPHYT_RS20040 | 53855.3093 | 65015.1049 | 63968.9005 | 136.063471 | 89.7108047 | 101.402099 | 9.11879925 | 0 | 0 |
| gene-BPHYT_RS20045 | 14203.8699 | 17587.2772 | 19046.1944 | 235.646157 | 223.031028 | 268.896637 | 6.12311149 | 0 | 0 |
| gene-BPHYT_RS20055 | 17278.9675 | 17052.81 | 12531.0599 | 44.3685232 | 52.3313027 | 59.7548083 | 8.22336684 | 0 | 0 |
| gene-BPHYT_RS20255 | 13.5677805 | 24.5364173 | 23.3845338 | 9125.12627 | 8539.97021 | 7017.56847 | -8.68431482 | 0 | 0 |
| gene-BPHYT_RS20265 | 69.4351118 | 35.2044248 | 68.1201636 | 10754.93 | 9722.40846 | 8276.04095 | -7.36259658 | 0 | 0 |
| gene-BPHYT_RS20270 | 35.914713 | 35.2044248 | 42.7021921 | 8431.00538 | 8135.02561 | 6347.59032 | -7.656228 | 0 | 0 |
| gene-BPHYT_RS20275 | 79.8104733 | 50.1396353 | 102.688605 | 16190.5671 | 15706.8667 | 12204.4669 | -7.56200429 | 0 | 0 |
| gene-BPHYT_RS20435 | 5058.3878 | 4993.69431 | 5255.41978 | 132.119602 | 148.272024 | 140.333262 | 5.18835828 | 0 | 0 |
| gene-BPHYT_RS27155 | 27698.2248 | 25697.0965 | 24424.6372 | 879.482727 | 898.35403 | 877.309231 | 4.87361753 | 0 | 0 |
| gene-BPHYT_RS27160 | 41765.6188 | 41685.2393 | 38471.6249 | 77.8914074 | 99.6786719 | 100.496723 | 8.77912452 | 0 | 0 |
| gene-BPHYT_RS29620 | 20200.0308 | 20563.6513 | 17325.9061 | 716.798142 | 722.670371 | 669.072778 | 4.78474065 | 0 | 0 |
| gene-BPHYT_RS33515 | 74936.4477 | 68778.778 | 70775.8332 | 2878.03821 | 3445.1441 | 3144.37044 | 4.50222714 | 0 | 0 |
| gene-BPHYT_RS30065 | 4109.44127 | 3450.03363 | 4091.27669 | 70.0036699 | 56.0692529 | 47.9849218 | 6.06699336 | 9.46E-278 | 1.76E-275 |
| gene-BPHYT_RS17290 | 11126.3781 | 11367.8288 | 10588.1102 | 114331.769 | 121863.406 | 111468.068 | -3.39354512 | 7.06E-277 | 1.27E-274 |
| gene-BPHYT_RS20280 | 37.5109225 | 28.8036203 | 30.5015658 | 4411.21717 | 4312.34854 | 3428.65847 | -6.95662778 | 5.50E-275 | 9.58E-273 |
| gene-BPHYT_RS07370 | 177.977356 | 164.287316 | 191.143146 | 3566.2433 | 3817.69313 | 3947.43885 | -4.4082207 | 4.19E-269 | 7.07E-267 |
| gene-BPHYT_RS05015 | 642.47431 | 592.074416 | 560.212092 | 7805.90218 | 8070.23447 | 8472.50751 | -3.76054752 | 1.36E-261 | 2.23E-259 |
| gene-BPHYT_RS11060 | 8400.05232 | 9350.50858 | 9239.94099 | 479.180051 | 579.38228 | 471.700835 | 4.14387694 | 3.84E-259 | 6.10E-257 |
| gene-BPHYT_RS22965 | 19.9526183 | 12.801609 | 16.2675018 | 4203.1781 | 5073.6444 | 5960.08944 | -8.25563731 | 2.42E-244 | 3.74E-242 |
| gene-BPHYT_RS06960 | 431.774661 | 268.833789 | 331.450348 | 12081.0559 | 11277.3957 | 10859.0783 | -5.04593647 | 2.39E-241 | 3.58E-239 |
| gene-BPHYT_RS18250 | 8329.8191 | 9108.34481 | 11648.548 | 369.737693 | 372.549036 | 378.447119 | 4.69759433 | 8.34E-241 | 1.22E-238 |
| gene-BPHYT_RS06955 | 58.2616455 | 49.0728345 | 52.8693807 | 2025.17659 | 1957.43992 | 1949.27428 | -5.20235955 | 6.15E-231 | 8.73E-229 |
| gene-BPHYT_RS05020 | 2091.83251 | 2119.73309 | 2051.73866 | 17232.7344 | 17112.336 | 16567.4733 | -3.02301792 | 6.34E-231 | 8.78E-229 |
| gene-BPHYT_RS13770 | 7368.901 | 11113.9302 | 14812.5771 | 100.568653 | 85.9728545 | 74.2408224 | 6.99755582 | 3.44E-226 | 4.64E-224 |
| gene-BPHYT_RS19085 | 2221.12547 | 2241.34838 | 2233.73133 | 31039.2329 | 37605.025 | 28864.2885 | -3.86406708 | 4.61E-226 | 6.07E-224 |
| gene-BPHYT_RS20225 | 142.860747 | 96.0120675 | 140.307203 | 6152.43522 | 5002.62334 | 4503.33964 | -5.36150806 | 2.78E-222 | 3.58E-220 |
| gene-BPHYT_RS11375 | 513.979448 | 529.133172 | 489.041771 | 5902.98552 | 6590.00619 | 7050.162 | -3.67308903 | 1.16E-218 | 1.45E-216 |
| gene-BPHYT_RS35140 | 5834.9437 | 5849.26851 | 5431.31215 | 559.043392 | 558.200563 | 580.345941 | 3.33325894 | 2.21E-217 | 2.71E-215 |
| gene-BPHYT_RS07375 | 106.946034 | 112.014079 | 122.006263 | 2185.88924 | 2153.05931 | 2125.82257 | -4.24807438 | 2.13E-216 | 2.55E-214 |
| gene-BPHYT_RS05010 | 920.214757 | 753.16133 | 726.953984 | 10355.6133 | 10248.2135 | 10301.3668 | -3.6845169 | 9.81E-213 | 1.15E-210 |
| gene-BPHYT_RS24490 | 14300.4406 | 13052.3072 | 11404.5354 | 1086.53584 | 1087.74351 | 1081.92418 | 3.57333218 | 2.88E-208 | 3.31E-206 |
| gene-BPHYT_RS26220 | 8055.27107 | 7751.37425 | 6727.62869 | 539.324049 | 605.547932 | 612.034097 | 3.68146801 | 4.23E-207 | 4.76E-205 |
| gene-BPHYT_RS26335 | 9791.14887 | 8051.14526 | 5935.6047 | 151.838946 | 170.699726 | 198.277318 | 5.51114652 | 6.83E-207 | 7.52E-205 |
| gene-BPHYT_RS03390 | 6483.00475 | 6635.50067 | 4084.15966 | 71.9756043 | 102.170639 | 72.4300706 | 6.13622424 | 2.52E-203 | 2.72E-201 |
| gene-BPHYT_RS04640 | 11226.1412 | 10610.4003 | 6178.60051 | 130.147668 | 158.239892 | 139.427886 | 6.03625909 | 2.15E-202 | 2.27E-200 |
| gene-BPHYT_RS06915 | 449.332965 | 371.246661 | 450.406455 | 5655.50776 | 5040.00285 | 5474.80796 | -3.66760211 | 4.17E-200 | 4.33E-198 |
| gene-BPHYT_RS13775 | 2648.90961 | 3377.49118 | 3802.52853 | 65.073834 | 51.0853193 | 77.8623259 | 5.6471147 | 6.15E-200 | 6.26E-198 |
| gene-BPHYT_RS17340 | 6139.02161 | 6956.60769 | 7563.37159 | 472.27828 | 553.216629 | 522.401885 | 3.73955465 | 1.00E-197 | 1.00E-195 |
| gene-BPHYT_RS18365 | 10239.6837 | 10149.5423 | 10448.8197 | 1136.82016 | 1113.90916 | 1286.53913 | 3.12294738 | 8.27E-194 | 8.11E-192 |
| gene-BPHYT_RS30075 | 8805.48952 | 8510.93639 | 8014.79477 | 913.991578 | 1022.95237 | 928.915656 | 3.14513315 | 2.33E-192 | 2.25E-190 |
| gene-BPHYT_RS35165 | 44.6938651 | 61.8744435 | 46.7690675 | 2279.55613 | 2323.75904 | 1776.34748 | -5.38900535 | 3.43E-186 | 3.24E-184 |
| gene-BPHYT_RS05225 | 2525.20338 | 3140.66141 | 2960.68532 | 27744.1305 | 27420.3567 | 29703.572 | -3.29894381 | 1.31E-185 | 1.22E-183 |
| gene-BPHYT_RS35130 | 18899.9182 | 19890.5 | 19154.9833 | 2210.53842 | 2592.89145 | 2402.86759 | 3.00785965 | 6.88E-185 | 6.29E-183 |
| gene-BPHYT_RS35155 | 149.245585 | 80.0100563 | 156.574704 | 6291.45659 | 5869.82779 | 4910.75879 | -5.46056344 | 9.70E-185 | 8.72E-183 |
| gene-BPHYT_RS18270 | 6016.91158 | 6166.10834 | 8130.70072 | 254.379533 | 210.571194 | 316.881559 | 4.69369871 | 8.39E-178 | 7.42E-176 |
| gene-BPHYT_RS22335 | 1658.46164 | 1912.77375 | 1788.40847 | 62.1159325 | 74.7590039 | 47.0795459 | 4.88389892 | 8.31E-175 | 7.23E-173 |
| gene-BPHYT_RS29525 | 4512.48416 | 5115.3096 | 5685.49186 | 44313.309 | 46972.3281 | 46609.6558 | -3.17097173 | 1.28E-173 | 1.09E-171 |
| gene-BPHYT_RS12830 | 5509.31697 | 5616.70595 | 5756.66218 | 33640.2143 | 35042.0371 | 32935.7639 | -2.58957577 | 6.71E-173 | 5.66E-171 |
| gene-BPHYT_RS01885 | 29609.6856 | 29323.1522 | 29008.0058 | 161632.558 | 169180.872 | 164209.835 | -2.49288124 | 1.25E-172 | 1.04E-170 |
| gene-BPHYT_RS26205 | 4687.2691 | 4649.11767 | 3775.07713 | 341.144645 | 309.003883 | 354.907346 | 3.70311252 | 1.12E-171 | 9.18E-170 |
| gene-BPHYT_RS27465 | 2790.97225 | 3045.71614 | 2643.46903 | 157.754749 | 137.058174 | 195.561191 | 4.1031108 | 1.44E-171 | 1.16E-169 |
| gene-BPHYT_RS22095 | 4629.00745 | 4473.09555 | 4502.03111 | 26341.0992 | 28551.7096 | 27216.5044 | -2.59338177 | 6.16E-168 | 4.89E-166 |
| gene-BPHYT_RS16735 | 4390.37414 | 4325.87704 | 4530.49924 | 26836.0548 | 25398.1256 | 27269.0162 | -2.58541663 | 4.17E-167 | 3.26E-165 |
| gene-BPHYT_RS35135 | 12869.4388 | 12588.2489 | 11231.6932 | 1285.70121 | 989.310818 | 1149.82737 | 3.42001783 | 6.53E-167 | 5.03E-165 |
| gene-BPHYT_RS24635 | 4453.42441 | 4370.68267 | 3843.19729 | 451.57297 | 403.698621 | 451.782566 | 3.27475985 | 2.42E-164 | 1.84E-162 |
| gene-BPHYT_RS29975 | 2813.31919 | 3437.23202 | 3054.22345 | 277.056778 | 270.378397 | 262.559006 | 3.52194459 | 3.32E-164 | 2.49E-162 |
| gene-BPHYT_RS07360 | 94.9744633 | 146.151703 | 170.808768 | 3721.04015 | 3320.54576 | 3415.07783 | -4.67640945 | 9.56E-164 | 7.06E-162 |
| gene-BPHYT_RS07060 | 15497.5977 | 14540.4942 | 11070.0349 | 950.472364 | 956.91525 | 1072.87042 | 3.78544556 | 7.65E-163 | 5.58E-161 |
| gene-BPHYT_RS18265 | 2692.80537 | 2751.27914 | 3887.93292 | 85.7791449 | 118.368423 | 108.645106 | 4.90355846 | 1.10E-160 | 7.90E-159 |
| gene-BPHYT_RS31000 | 55.0692266 | 36.2712255 | 63.0365693 | 1404.01727 | 1617.28645 | 1633.29809 | -4.90559597 | 1.62E-160 | 1.15E-158 |
| gene-BPHYT_RS09840 | 6340.144 | 6988.61172 | 4110.59435 | 10.845639 | 4.98393359 | 6.33763118 | 9.59552652 | 5.31E-159 | 3.72E-157 |
| gene-BPHYT_RS23970 | 176.381146 | 161.086913 | 172.842206 | 2096.16623 | 2020.98507 | 1841.53455 | -3.54326938 | 3.33E-158 | 2.31E-156 |
| gene-BPHYT_RS35145 | 2393.5161 | 2282.95361 | 2026.32069 | 181.417962 | 195.619394 | 188.318184 | 3.56940317 | 4.25E-157 | 2.90E-155 |
| gene-BPHYT_RS12840 | 15040.2837 | 15277.6535 | 15573.0828 | 104046.159 | 130521.745 | 119368.378 | -2.94720638 | 7.27E-155 | 4.90E-153 |
| gene-BPHYT_RS19380 | 6163.76285 | 6487.21536 | 7007.22638 | 38793.8648 | 40660.1762 | 40018.5194 | -2.60359728 | 5.04E-154 | 3.36E-152 |
| gene-BPHYT_RS11450 | 1890.71011 | 1583.13231 | 1275.98217 | 19711.4559 | 19750.0829 | 19729.0459 | -3.63849262 | 7.33E-154 | 4.82E-152 |
| gene-BPHYT_RS10100 | 1044.7191 | 1078.53556 | 1231.24654 | 8297.89981 | 8344.35082 | 9118.04052 | -2.94164567 | 6.38E-153 | 4.15E-151 |
| gene-BPHYT_RS20245 | 20.7507231 | 24.5364173 | 32.5350035 | 1628.81779 | 1360.61387 | 1224.97357 | -5.77471113 | 9.62E-151 | 6.18E-149 |
| gene-BPHYT_RS10310 | 11280.4123 | 11733.7415 | 8875.95564 | 860.74935 | 996.786719 | 1004.96723 | 3.47805707 | 1.07E-148 | 6.80E-147 |
| gene-BPHYT_RS02265 | 2889.93724 | 2805.68597 | 2846.81281 | 15584.1973 | 17290.5116 | 17195.8042 | -2.55111773 | 3.61E-148 | 2.26E-146 |
| gene-BPHYT_RS11185 | 18477.7208 | 17792.1029 | 14784.1089 | 1243.30462 | 1658.4039 | 1293.78214 | 3.60644735 | 3.64E-148 | 2.26E-146 |
| gene-BPHYT_RS31030 | 35.914713 | 37.3380263 | 38.6353167 | 1122.03065 | 1140.07481 | 1001.34573 | -4.86862603 | 4.94E-148 | 3.01E-146 |
| gene-BPHYT_RS11380 | 213.892069 | 204.825744 | 186.059551 | 2150.39442 | 2049.64269 | 2402.86759 | -3.44705814 | 4.97E-148 | 3.01E-146 |
| gene-BPHYT_RS07390 | 130.889176 | 145.084902 | 175.892363 | 2026.16256 | 1845.30141 | 1951.08503 | -3.69305415 | 4.97E-147 | 2.98E-145 |
| gene-BPHYT_RS12835 | 2528.3958 | 2400.30169 | 2454.35933 | 13569.8663 | 14914.4213 | 13824.1844 | -2.51837385 | 2.94E-145 | 1.75E-143 |
| gene-BPHYT_RS20050 | 2229.10652 | 2317.09123 | 2442.1587 | 261.281303 | 213.063161 | 229.060098 | 3.30931693 | 1.16E-144 | 6.81E-143 |
| gene-BPHYT_RS24640 | 3782.21833 | 3469.23604 | 3948.93605 | 411.148315 | 479.703608 | 405.608396 | 3.11410255 | 8.13E-144 | 4.71E-142 |
| gene-BPHYT_RS19390 | 7881.28424 | 9396.38101 | 9391.4321 | 57956.1369 | 60263.233 | 60785.126 | -2.74693202 | 2.10E-143 | 1.21E-141 |
| gene-BPHYT_RS35160 | 31.1260846 | 45.8724323 | 45.7523487 | 1479.93674 | 1497.67205 | 1142.58436 | -5.08619407 | 3.43E-141 | 1.95E-139 |
| gene-BPHYT_RS19385 | 26231.3083 | 29374.3587 | 29210.3328 | 158092.936 | 171077.259 | 158694.285 | -2.52410765 | 7.76E-141 | 4.36E-139 |
| gene-BPHYT_RS17455 | 2035.16707 | 2147.46991 | 1623.70002 | 16019.9948 | 16703.6534 | 16523.1099 | -3.08418502 | 5.75E-140 | 3.20E-138 |
| gene-BPHYT_RS33895 | 3685.64766 | 3849.01711 | 3839.13041 | 18917.7523 | 20531.3144 | 20108.3984 | -2.38867045 | 1.04E-138 | 5.71E-137 |
| gene-BPHYT_RS16725 | 1826.06363 | 1785.82446 | 1470.17547 | 12033.7295 | 12746.4102 | 12487.8496 | -2.87400441 | 8.05E-138 | 4.39E-136 |
| gene-BPHYT_RS23980 | 119.71571 | 87.4776615 | 150.474391 | 2158.28216 | 1986.09754 | 1997.2592 | -4.09968338 | 4.02E-137 | 2.17E-135 |
| gene-BPHYT_RS26245 | 2178.02782 | 2213.61156 | 1891.09708 | 212.968911 | 215.555128 | 224.533219 | 3.26547151 | 6.07E-136 | 3.24E-134 |
| gene-BPHYT_RS15600 | 1769.39819 | 1985.3162 | 2288.63415 | 14965.0099 | 14970.4905 | 15276.4073 | -2.90390756 | 1.29E-135 | 6.82E-134 |
| gene-BPHYT_RS19395 | 22668.5687 | 27590.6678 | 27267.3831 | 165244.156 | 169583.324 | 169458.299 | -2.7015426 | 8.58E-135 | 4.48E-133 |
| gene-BPHYT_RS07365 | 55.0692266 | 68.275248 | 127.089857 | 2855.36096 | 2869.49977 | 3079.18338 | -5.14651912 | 8.63E-135 | 4.48E-133 |
| gene-BPHYT_RS19090 | 440.553813 | 459.791123 | 474.807707 | 3620.47149 | 4583.97292 | 3908.50769 | -3.13920497 | 1.06E-134 | 5.44E-133 |
| gene-BPHYT_RS18255 | 1979.29974 | 1979.98219 | 2730.90686 | 93.6668823 | 78.4969541 | 116.793489 | 4.52212738 | 1.59E-134 | 8.07E-133 |
| gene-BPHYT_RS11180 | 27828.3158 | 27287.6964 | 23290.9956 | 3076.21761 | 3644.50144 | 3607.92289 | 2.9245075 | 2.23E-134 | 1.12E-132 |
| gene-BPHYT_RS19400 | 5047.21433 | 6056.22786 | 6634.09056 | 43771.0271 | 43907.209 | 44472.9688 | -2.89757875 | 1.44E-131 | 7.20E-130 |
| gene-BPHYT_RS29535 | 484.449573 | 389.382274 | 501.242398 | 3753.57706 | 3690.60283 | 3755.49916 | -3.02417672 | 5.08E-131 | 2.51E-129 |
| gene-BPHYT_RS07410 | 62.2521692 | 88.5444623 | 72.187039 | 1277.81347 | 1227.29365 | 1131.71985 | -4.0390857 | 1.40E-130 | 6.85E-129 |
| gene-BPHYT_RS34855 | 1755.03231 | 1945.84457 | 1741.63941 | 148.881045 | 189.389477 | 146.670893 | 3.49659375 | 3.12E-130 | 1.51E-128 |
| gene-BPHYT_RS02145 | 1064.67171 | 955.853472 | 1066.53808 | 5841.85556 | 6268.54248 | 6381.08922 | -2.58193319 | 4.58E-129 | 2.20E-127 |
| gene-BPHYT_RS24485 | 1525.17815 | 1391.10818 | 1415.27265 | 99.5826854 | 135.81219 | 105.023602 | 3.68091471 | 2.05E-127 | 9.79E-126 |
| gene-BPHYT_RS31025 | 174.784937 | 181.356128 | 174.875644 | 1546.98251 | 1482.72024 | 1477.57344 | -3.08625515 | 7.62E-127 | 3.61E-125 |
| gene-BPHYT_RS17355 | 1780.57166 | 1882.90332 | 2059.87241 | 133.10557 | 186.89751 | 138.52251 | 3.65159621 | 1.89E-126 | 8.85E-125 |
| gene-BPHYT_RS21640 | 117.321396 | 148.285304 | 115.90595 | 1582.47733 | 1559.97122 | 1378.88747 | -3.5714591 | 3.83E-125 | 1.78E-123 |
| gene-BPHYT_RS06145 | 20629.4111 | 21480.0331 | 18237.9029 | 3311.86377 | 3613.35186 | 3457.6305 | 2.53928505 | 1.64E-124 | 7.56E-123 |
| gene-BPHYT_RS29625 | 34394.3235 | 34962.261 | 30520.8834 | 6177.0844 | 6536.42891 | 6222.64844 | 2.39912936 | 5.52E-123 | 2.53E-121 |
| gene-BPHYT_RS30995 | 39.9052367 | 30.9372218 | 55.9195373 | 990.897018 | 936.979516 | 955.171557 | -4.50787102 | 7.29E-122 | 3.31E-120 |
| gene-BPHYT_RS02075 | 696.745432 | 733.958916 | 647.649913 | 3986.26532 | 4321.07043 | 4037.97644 | -2.57036394 | 4.33E-121 | 1.95E-119 |
| gene-BPHYT_RS18260 | 1834.04468 | 1600.20113 | 2580.43247 | 78.8773746 | 73.5130205 | 94.1590918 | 4.60187917 | 4.57E-120 | 2.04E-118 |
| gene-BPHYT_RS19405 | 6169.34959 | 7484.67406 | 8083.93165 | 48810.3054 | 49494.1985 | 48864.0418 | -2.75940134 | 8.88E-120 | 3.93E-118 |
| gene-BPHYT_RS20235 | 55.0692266 | 29.870421 | 66.0867259 | 2010.38709 | 1542.52745 | 1393.37348 | -5.02467769 | 3.06E-118 | 1.34E-116 |
| gene-BPHYT_RS06920 | 720.688574 | 470.459131 | 643.583038 | 6710.49264 | 6125.25439 | 6440.84403 | -3.39083032 | 2.00E-117 | 8.71E-116 |
| gene-BPHYT_RS22070 | 12618.8339 | 11171.5375 | 12186.3923 | 56524.5126 | 55043.8086 | 57454.2482 | -2.23200127 | 2.22E-116 | 9.57E-115 |
| gene-BPHYT_RS11445 | 317.645684 | 289.103003 | 326.366754 | 2010.38709 | 2128.13964 | 2114.05269 | -2.74313098 | 3.09E-116 | 1.32E-114 |
| gene-BPHYT_RS34155 | 2086.24577 | 2317.09123 | 2436.05839 | 310.579662 | 313.987816 | 326.840694 | 2.84469468 | 3.75E-116 | 1.59E-114 |
| gene-BPHYT_RS13785 | 926.599595 | 803.300965 | 849.976966 | 17.7474093 | 31.149585 | 21.7290212 | 5.220697 | 6.77E-115 | 2.85E-113 |
| gene-BPHYT_RS18080 | 6559.6228 | 7874.05634 | 7304.10829 | 37757.6132 | 40420.9474 | 38225.8752 | -2.42105291 | 2.40E-114 | 1.01E-112 |
| gene-BPHYT_RS06925 | 676.792814 | 632.612845 | 665.950853 | 3819.63686 | 3842.6128 | 3468.49501 | -2.49349406 | 2.77E-114 | 1.15E-112 |
| gene-BPHYT_RS16475 | 5316.17563 | 4020.77203 | 3174.19628 | 252.407599 | 241.720779 | 185.602056 | 4.20495712 | 3.69E-114 | 1.52E-112 |
| gene-BPHYT_RS18075 | 1635.3166 | 1972.51459 | 1783.32488 | 9833.05071 | 10894.8788 | 10438.0786 | -2.53196046 | 4.77E-114 | 1.95E-112 |
| gene-BPHYT_RS03025 | 134.081595 | 94.9452668 | 170.808768 | 1908.83247 | 2229.0643 | 1975.53018 | -3.93200433 | 4.43E-113 | 1.80E-111 |
| gene-BPHYT_RS16720 | 587.405084 | 593.141217 | 621.215223 | 3127.4879 | 3158.56792 | 3301.00047 | -2.41218028 | 7.52E-113 | 3.03E-111 |
| gene-BPHYT_RS22625 | 165.20768 | 153.619308 | 168.775331 | 1575.57556 | 1419.17509 | 1803.50876 | -3.29862511 | 1.21E-112 | 4.85E-111 |
| gene-BPHYT_RS05935 | 1216.31161 | 1285.4949 | 1234.2967 | 5781.71156 | 6077.90702 | 5784.44652 | -2.23980843 | 1.67E-112 | 6.64E-111 |
| gene-BPHYT_RS28080 | 7253.17582 | 6224.78238 | 4731.80957 | 541.295983 | 571.90638 | 491.619105 | 3.50562151 | 2.00E-112 | 7.87E-111 |
| gene-BPHYT_RS26585 | 2587.45555 | 2526.18418 | 2473.67699 | 311.56563 | 309.003883 | 389.31163 | 2.90590266 | 7.46E-112 | 2.92E-110 |
| gene-BPHYT_RS10780 | 12.7696757 | 19.2024135 | 22.3678149 | 1226.54318 | 827.332977 | 1275.67462 | -5.9639381 | 1.31E-111 | 5.09E-110 |
| gene-BPHYT_RS07405 | 72.6275307 | 46.939233 | 49.8192241 | 956.388167 | 946.947383 | 952.455429 | -4.05589556 | 1.97E-111 | 7.57E-110 |
| gene-BPHYT_RS18380 | 18429.8345 | 17783.5685 | 17068.6762 | 77652.8033 | 72673.2277 | 76899.9114 | -2.09238239 | 7.52E-110 | 2.88E-108 |
| gene-BPHYT_RS26200 | 1133.30872 | 1063.60035 | 1193.62794 | 38.4527201 | 73.5130205 | 66.9978153 | 4.25450482 | 1.36E-109 | 5.15E-108 |
| gene-BPHYT_RS16730 | 1903.47979 | 1731.41762 | 1560.66345 | 10048.9775 | 9280.08435 | 10118.4809 | -2.50224031 | 1.76E-109 | 6.64E-108 |
| gene-BPHYT_RS17350 | 901.858349 | 867.30901 | 989.26745 | 64.0878669 | 41.1174522 | 51.6064253 | 4.12341473 | 1.83E-109 | 6.86E-108 |
| gene-BPHYT_RS19080 | 4752.71369 | 4766.46575 | 3675.43868 | 539.324049 | 578.136297 | 497.05136 | 3.03247503 | 4.99E-109 | 1.86E-107 |
| gene-BPHYT_RS13310 | 1986.48268 | 1885.03693 | 1258.69795 | 73.9475387 | 83.4808877 | 94.1590918 | 4.348291 | 1.71E-108 | 6.32E-107 |
| gene-BPHYT_RS35670 | 1160.44428 | 1106.27238 | 1034.00308 | 5711.70789 | 6606.20398 | 5884.94324 | -2.46252849 | 2.11E-107 | 7.74E-106 |
| gene-BPHYT_RS15595 | 691.158699 | 935.584258 | 950.632133 | 6435.4078 | 7042.29817 | 6836.49329 | -2.98040248 | 3.93E-107 | 1.43E-105 |
| gene-BPHYT_RS18085 | 11430.456 | 13763.8633 | 13799.9251 | 66847.589 | 71151.882 | 69790.8999 | -2.41391453 | 1.24E-106 | 4.48E-105 |
| gene-BPHYT_RS19410 | 5741.56545 | 6587.49463 | 7213.62031 | 40795.3781 | 37014.4288 | 36402.4481 | -2.54722194 | 2.15E-106 | 7.74E-105 |
| gene-BPHYT_RS32415 | 1593.01705 | 1414.5778 | 1533.21204 | 212.968911 | 204.341277 | 201.898822 | 2.87481403 | 2.63E-106 | 9.39E-105 |
| gene-BPHYT_RS26860 | 2180.42213 | 2575.25701 | 2700.40529 | 346.074481 | 300.281999 | 333.178325 | 2.92550803 | 5.16E-106 | 1.83E-104 |
| gene-BPHYT_RS17335 | 4771.8682 | 5136.64561 | 4919.90256 | 950.472364 | 1017.96844 | 876.403855 | 2.38323453 | 1.33E-105 | 4.69E-104 |
| gene-BPHYT_RS03315 | 694.351118 | 601.675623 | 567.329124 | 3720.05418 | 4309.85658 | 4007.19366 | -2.68944502 | 1.98E-105 | 6.94E-104 |
| gene-BPHYT_RS18070 | 7052.85153 | 7680.9654 | 6904.53777 | 31715.6064 | 32011.8055 | 30272.148 | -2.11912276 | 5.50E-105 | 1.91E-103 |
| gene-BPHYT_RS10470 | 1810.10154 | 1531.92588 | 1619.63314 | 225.786485 | 221.785045 | 213.668708 | 2.90884099 | 5.52E-105 | 1.91E-103 |
| gene-BPHYT_RS12285 | 9364.96094 | 14254.5916 | 14782.0755 | 1059.91472 | 1221.06373 | 1087.35644 | 3.51148822 | 1.50E-104 | 5.17E-103 |
| gene-BPHYT_RS11190 | 84259.1091 | 81014.9826 | 58756.1829 | 7765.47753 | 9348.61344 | 8559.4236 | 3.1254409 | 2.01E-104 | 6.88E-103 |
| gene-BPHYT_RS05930 | 1234.66802 | 1463.65063 | 1228.19638 | 6940.223 | 6825.49706 | 7169.67162 | -2.41541151 | 1.11E-103 | 3.76E-102 |
| gene-BPHYT_RS11250 | 2933.833 | 3091.58857 | 2074.10647 | 195.221502 | 230.506929 | 235.39773 | 3.61553149 | 1.76E-103 | 5.92E-102 |
| gene-BPHYT_RS26215 | 3157.30233 | 3157.73022 | 2495.02808 | 298.748056 | 307.757899 | 378.447119 | 3.15869341 | 2.49E-103 | 8.34E-102 |
| gene-BPHYT_RS01995 | 498.017354 | 554.73639 | 564.278967 | 2867.19257 | 2830.87428 | 2907.16196 | -2.41364402 | 2.83E-103 | 9.43E-102 |
| gene-BPHYT_RS02840 | 11693.8306 | 13377.6814 | 13482.7088 | 57680.0661 | 60425.2109 | 57371.859 | -2.18641916 | 1.25E-101 | 4.14E-100 |
| gene-BPHYT_RS03850 | 5102.28356 | 5292.39852 | 4987.00601 | 1099.35341 | 1186.1762 | 1135.34136 | 2.16931845 | 4.62E-101 | 1.52E-99 |
| gene-BPHYT_RS26630 | 1414.24159 | 1440.18101 | 1271.91529 | 133.10557 | 147.026041 | 174.737545 | 3.17802509 | 5.28E-101 | 1.73E-99 |
| gene-BPHYT_RS18160 | 13116.8513 | 14808.2612 | 15165.3785 | 66219.5279 | 72414.0631 | 65347.3151 | -2.24306074 | 1.02E-100 | 3.33E-99 |
| gene-BPHYT_RS09700 | 4950.64366 | 4923.28546 | 4409.50969 | 892.3003 | 970.621067 | 971.468322 | 2.33357959 | 2.26E-100 | 7.29E-99 |
| gene-BPHYT_RS15605 | 2234.69325 | 2442.97372 | 2931.20047 | 14663.3039 | 14980.4584 | 15586.9512 | -2.57199136 | 5.42E-100 | 1.74E-98 |
| gene-BPHYT_RS06150 | 10894.1296 | 10366.1029 | 8973.56065 | 1699.80742 | 1939.99615 | 1843.3453 | 2.46356771 | 7.76E-100 | 2.48E-98 |
| gene-BPHYT_RS30010 | 1047.91152 | 1504.18906 | 1692.8369 | 74.9335059 | 59.8072031 | 77.8623259 | 4.31004382 | 1.60E-99 | 5.08E-98 |
| gene-BPHYT_RS31010 | 169.198203 | 158.953312 | 145.390797 | 1187.10449 | 1172.47038 | 1173.36714 | -2.89630898 | 2.42E-99 | 7.63E-98 |
| gene-BPHYT_RS30070 | 937.773062 | 892.912228 | 1022.81917 | 89.7230136 | 100.924655 | 92.3483401 | 3.33806762 | 3.97E-99 | 1.25E-97 |
| gene-BPHYT_RS02715 | 1105.37506 | 1173.48083 | 1246.49732 | 5467.18803 | 5356.48263 | 5229.4511 | -2.1876508 | 1.25E-98 | 3.90E-97 |
| gene-BPHYT_RS11370 | 426.187928 | 310.439018 | 373.135821 | 2827.75388 | 3402.78066 | 3706.60887 | -3.15950447 | 3.78E-98 | 1.17E-96 |
| gene-BPHYT_RS24480 | 853.972065 | 1017.72792 | 893.695877 | 70.9896371 | 62.2991699 | 83.2945812 | 3.66295728 | 3.99E-98 | 1.23E-96 |
| gene-BPHYT_RS04195 | 8890.08862 | 10474.9166 | 8233.38932 | 50163.0523 | 51389.3393 | 47450.75 | -2.43275057 | 1.44E-97 | 4.40E-96 |
| gene-BPHYT_RS04200 | 46314.0177 | 53571.5333 | 43384.4104 | 237129.051 | 259072.344 | 237052.757 | -2.35558738 | 1.91E-97 | 5.81E-96 |
| gene-BPHYT_RS18560 | 3050.35629 | 3126.793 | 2991.18688 | 11608.7776 | 11803.2007 | 12094.9164 | -1.95342222 | 2.76E-97 | 8.36E-96 |
| gene-BPHYT_RS03385 | 3420.67689 | 3185.46704 | 1921.59864 | 102.540587 | 137.058174 | 161.156907 | 4.41052725 | 7.49E-96 | 2.26E-94 |
| gene-BPHYT_RS03365 | 2822.89644 | 2800.35197 | 2968.81907 | 577.776769 | 480.949592 | 504.294367 | 2.45721585 | 1.63E-95 | 4.90E-94 |
| gene-BPHYT_RS18845 | 916.224234 | 1030.52953 | 999.434639 | 5586.49006 | 7494.59014 | 6197.29792 | -2.71043039 | 5.52E-94 | 1.65E-92 |
| gene-BPHYT_RS30990 | 31.9241893 | 32.0040225 | 20.3343772 | 590.594342 | 610.531865 | 621.993232 | -4.4265564 | 1.76E-93 | 5.21E-92 |
| gene-BPHYT_RS01700 | 2938.62163 | 2370.43127 | 1902.28099 | 234.660189 | 237.982829 | 224.533219 | 3.37208843 | 4.79E-93 | 1.41E-91 |
| gene-BPHYT_RS03430 | 243.421944 | 214.426951 | 275.530811 | 1592.337 | 1807.92191 | 1669.51313 | -2.78819421 | 4.00E-92 | 1.17E-90 |
| gene-BPHYT_RS08025 | 964.908622 | 1093.47077 | 1003.50151 | 119.302029 | 94.6947383 | 85.105333 | 3.35530699 | 4.89E-92 | 1.43E-90 |
| gene-BPHYT_RS07380 | 63.8483787 | 98.145669 | 115.90595 | 1175.27288 | 1203.61996 | 1193.28541 | -3.69762107 | 6.67E-92 | 1.93E-90 |
| gene-BPHYT_RS32440 | 9383.31735 | 8513.06999 | 6916.7384 | 979.065412 | 1113.90916 | 1259.37785 | 2.88765143 | 1.09E-91 | 3.14E-90 |
| gene-BPHYT_RS32420 | 2217.93305 | 2124.00029 | 2022.25381 | 386.499135 | 304.019949 | 344.948211 | 2.61677623 | 3.55E-91 | 1.02E-89 |
| gene-BPHYT_RS22340 | 626.512216 | 648.614856 | 688.318668 | 35.4948186 | 48.5933525 | 32.5935318 | 4.09429543 | 1.90E-90 | 5.44E-89 |
| gene-BPHYT_RS35710 | 1972.9149 | 1420.9786 | 1249.54748 | 102.540587 | 80.9889209 | 114.982737 | 3.9514927 | 8.89E-90 | 2.52E-88 |
| gene-BPHYT_RS34990 | 1019.97785 | 1026.26232 | 1109.24028 | 152.824913 | 137.058174 | 139.427886 | 2.87602641 | 1.03E-89 | 2.91E-88 |
| gene-BPHYT_RS21330 | 1383.1155 | 1629.00475 | 1656.23502 | 147.895077 | 193.127427 | 124.036496 | 3.33762065 | 1.40E-89 | 3.93E-88 |
| gene-BPHYT_RS18990 | 248.210572 | 268.833789 | 266.380341 | 1486.83851 | 1567.44712 | 1426.87239 | -2.51727292 | 3.77E-89 | 1.05E-87 |
| gene-BPHYT_RS15590 | 203.516707 | 193.090936 | 209.444085 | 1214.71157 | 1424.15902 | 1374.36059 | -2.72621397 | 6.07E-89 | 1.69E-87 |
| gene-BPHYT_RS23975 | 95.772568 | 97.0788683 | 73.2037579 | 1115.12888 | 912.059848 | 898.132876 | -3.45626789 | 8.78E-89 | 2.43E-87 |
| gene-BPHYT_RS16695 | 1188.37795 | 1178.81483 | 1113.30715 | 4571.92982 | 4880.51697 | 4887.21902 | -2.04235251 | 1.36E-88 | 3.73E-87 |
| gene-BPHYT_RS04340 | 803.691466 | 965.454679 | 890.645721 | 4276.13967 | 4453.14467 | 4097.73125 | -2.27101237 | 3.52E-88 | 9.63E-87 |
| gene-BPHYT_RS29385 | 2763.83669 | 2696.8723 | 2637.36872 | 633.976898 | 615.515799 | 610.223345 | 2.12257848 | 2.15E-87 | 5.87E-86 |
| gene-BPHYT_RS01430 | 13957.2556 | 15385.4004 | 14551.2803 | 3878.7949 | 3975.93302 | 3845.13138 | 1.90757945 | 3.28E-87 | 8.88E-86 |
| gene-BPHYT_RS31040 | 11.1734663 | 9.60120675 | 6.10031316 | 637.920767 | 635.451533 | 598.453459 | -6.09725538 | 4.70E-87 | 1.27E-85 |
| gene-BPHYT_RS12850 | 3032.79799 | 2999.84371 | 2958.65188 | 10523.2277 | 10651.9121 | 10985.831 | -1.83867214 | 5.82E-87 | 1.56E-85 |
| gene-BPHYT_RS05210 | 1808.50533 | 1867.96811 | 1925.66552 | 7131.50063 | 6973.76908 | 7508.2822 | -1.94824965 | 1.10E-86 | 2.94E-85 |
| gene-BPHYT_RS14970 | 295.298751 | 293.370206 | 206.393928 | 1987.70984 | 1942.48812 | 2093.22904 | -2.91952002 | 2.40E-86 | 6.38E-85 |
| gene-BPHYT_RS18690 | 9582.04543 | 10803.4912 | 10843.3066 | 39424.8838 | 40893.1751 | 40416.8848 | -1.95101115 | 2.82E-86 | 7.45E-85 |
| gene-BPHYT_RS06555 | 14440.907 | 16570.6161 | 17382.8423 | 67556.4994 | 67978.3623 | 67799.073 | -2.07102989 | 3.32E-86 | 8.73E-85 |
| gene-BPHYT_RS31020 | 107.744139 | 91.7448645 | 78.2873522 | 924.837217 | 827.332977 | 872.782351 | -3.23270956 | 9.67E-86 | 2.53E-84 |
| gene-BPHYT_RS27460 | 1377.52877 | 1322.83293 | 1254.63107 | 218.884715 | 230.506929 | 197.371943 | 2.61633669 | 6.83E-85 | 1.78E-83 |
| gene-BPHYT_RS07385 | 91.7820443 | 82.1436578 | 124.039701 | 914.977545 | 1004.26262 | 1062.00591 | -3.32454001 | 2.62E-84 | 6.79E-83 |
| gene-BPHYT_RS23855 | 910.637501 | 815.035773 | 699.502575 | 62.1159325 | 33.6415518 | 38.931163 | 4.15940703 | 9.56E-84 | 2.47E-82 |
| gene-BPHYT_RS29715 | 1102.98074 | 998.525502 | 1025.86933 | 137.049438 | 97.1867051 | 132.184879 | 3.08368197 | 9.93E-84 | 2.55E-82 |
| gene-BPHYT_RS28200 | 1573.06443 | 1814.62808 | 1697.9205 | 218.884715 | 176.929643 | 259.842878 | 2.94761096 | 1.18E-83 | 3.02E-82 |
| gene-BPHYT_RS22400 | 2844.44527 | 2467.51014 | 1393.92156 | 119.302029 | 98.4326885 | 121.320368 | 4.30211422 | 2.33E-83 | 5.93E-82 |
| gene-BPHYT_RS19350 | 5705.65074 | 7753.50785 | 6942.15637 | 34923.9436 | 38581.8759 | 37853.7657 | -2.44873884 | 3.79E-83 | 9.59E-82 |
| gene-BPHYT_RS24710 | 5014.49204 | 4878.47983 | 3838.11369 | 833.142269 | 776.247657 | 806.689912 | 2.50642483 | 1.98E-82 | 4.98E-81 |
| gene-BPHYT_RS05200 | 910.637501 | 1010.26031 | 969.949792 | 4631.08786 | 3979.67097 | 4578.48584 | -2.19087831 | 5.06E-82 | 1.27E-80 |
| gene-BPHYT_RS26125 | 857.164484 | 888.645025 | 542.927871 | 25.6351467 | 26.1656514 | 24.4451488 | 4.91112741 | 1.33E-81 | 3.32E-80 |
| gene-BPHYT_RS28050 | 728.669621 | 651.815258 | 592.747095 | 58.1720638 | 57.3152363 | 53.4171771 | 3.54958184 | 5.18E-80 | 1.29E-78 |
| gene-BPHYT_RS24630 | 986.45745 | 897.179431 | 722.887109 | 74.9335059 | 73.5130205 | 87.8214607 | 3.45920518 | 2.36E-79 | 5.85E-78 |
| gene-BPHYT_RS34135 | 1541.93835 | 1655.67476 | 887.595564 | 68.0317356 | 72.2670371 | 79.6730777 | 4.21355832 | 5.28E-79 | 1.30E-77 |
| gene-BPHYT_RS34970 | 1134.90493 | 1206.55165 | 1202.77841 | 168.600388 | 213.063161 | 186.507432 | 2.6457138 | 5.91E-79 | 1.45E-77 |
| gene-BPHYT_RS18840 | 761.391916 | 584.606811 | 623.248661 | 4031.61981 | 5084.85825 | 3936.57434 | -2.7263898 | 6.36E-79 | 1.55E-77 |
| gene-BPHYT_RS15055 | 1038.33426 | 1286.56171 | 1166.17653 | 176.488126 | 144.534074 | 145.765517 | 2.90012175 | 1.60E-78 | 3.89E-77 |
| gene-BPHYT_RS03420 | 3091.05963 | 3567.38171 | 3572.75007 | 12927.0157 | 13146.3708 | 13414.0491 | -1.94883577 | 3.27E-78 | 7.91E-77 |
| gene-BPHYT_RS10465 | 3664.89694 | 3663.39378 | 3565.63304 | 535.38018 | 674.077019 | 738.786721 | 2.48320432 | 3.53E-78 | 8.50E-77 |
| gene-BPHYT_RS35705 | 9594.8151 | 6667.50469 | 5230.00181 | 556.085491 | 631.713583 | 717.057699 | 3.49593525 | 4.36E-77 | 1.05E-75 |
| gene-BPHYT_RS13320 | 3968.17673 | 4468.82834 | 2765.4753 | 246.491796 | 397.468704 | 272.518141 | 3.61625531 | 2.43E-76 | 5.80E-75 |
| gene-BPHYT_RS16700 | 33451.7618 | 32872.3983 | 30275.8542 | 105399.892 | 111039.549 | 107463.59 | -1.74544441 | 5.54E-75 | 1.32E-73 |
| gene-BPHYT_RS05545 | 1399.0776 | 1424.179 | 1531.1786 | 5269.99459 | 5094.82612 | 5387.89188 | -1.85547198 | 1.43E-74 | 3.39E-73 |
| gene-BPHYT_RS28240 | 1564.28528 | 1275.8937 | 1140.75856 | 142.965241 | 77.2509707 | 108.645106 | 3.58835764 | 2.05E-74 | 4.82E-73 |
| gene-BPHYT_RS16690 | 1427.01126 | 1594.86712 | 1698.93721 | 6082.43155 | 6684.70093 | 6448.08704 | -2.02575917 | 2.39E-74 | 5.61E-73 |
| gene-BPHYT_RS11345 | 1223.49456 | 1315.36533 | 1215.99576 | 254.379533 | 244.212746 | 231.776226 | 2.36247671 | 4.83E-74 | 1.13E-72 |
| gene-BPHYT_RS18090 | 4452.62631 | 5918.61056 | 6207.06864 | 28382.0513 | 31969.442 | 30204.2448 | -2.44979637 | 5.09E-74 | 1.18E-72 |
| gene-BPHYT_RS02630 | 4575.53444 | 4686.4557 | 5077.49398 | 1216.6835 | 1313.2665 | 1277.48537 | 1.91337446 | 7.08E-74 | 1.64E-72 |
| gene-BPHYT_RS28250 | 636.887577 | 732.892115 | 466.673956 | 27.6070811 | 22.4277012 | 27.1612765 | 4.56207523 | 1.21E-73 | 2.80E-72 |
| gene-BPHYT_RS15510 | 3984.93693 | 3808.47868 | 2761.40842 | 470.306346 | 546.986712 | 535.077147 | 2.76619301 | 2.26E-73 | 5.19E-72 |
| gene-BPHYT_RS10385 | 2811.72298 | 2376.83207 | 1753.84003 | 284.944516 | 279.100281 | 252.599871 | 3.08911537 | 2.51E-73 | 5.74E-72 |
| gene-BPHYT_RS12515 | 1808.50533 | 1744.21923 | 1799.59238 | 6467.94472 | 6163.87987 | 5934.73891 | -1.79429279 | 2.80E-73 | 6.36E-72 |
| gene-BPHYT_RS20240 | 21.5488278 | 20.2692143 | 21.351096 | 625.103194 | 596.826048 | 414.662154 | -4.691575 | 3.69E-73 | 8.36E-72 |
| gene-BPHYT_RS18165 | 18803.3475 | 21883.2838 | 23748.5191 | 85887.6013 | 94399.4402 | 90033.2939 | -2.06880966 | 1.16E-72 | 2.63E-71 |
| gene-BPHYT_RS18310 | 6826.18978 | 7248.9111 | 7833.81881 | 25724.8698 | 24665.4874 | 25662.8794 | -1.79563983 | 1.91E-72 | 4.30E-71 |
| gene-BPHYT_RS06885 | 22031.6812 | 21674.1908 | 18488.0157 | 5213.79446 | 5504.75465 | 5511.023 | 1.93821114 | 9.79E-72 | 2.19E-70 |
| gene-BPHYT_RS10475 | 503.604087 | 478.993537 | 569.362561 | 34.5088514 | 41.1174522 | 41.6472906 | 3.72786249 | 1.01E-71 | 2.24E-70 |
| gene-BPHYT_RS20430 | 455.717803 | 419.252695 | 463.6238 | 11.8316062 | 14.9518008 | 12.6752624 | 5.09791318 | 3.73E-71 | 8.28E-70 |
| gene-BPHYT_RS12510 | 3113.40656 | 3100.12298 | 2931.20047 | 10187.0129 | 9635.18962 | 10062.3476 | -1.70836605 | 4.01E-71 | 8.88E-70 |
| gene-BPHYT_RS16020 | 686.370071 | 609.143228 | 623.248661 | 2633.51834 | 2489.47483 | 2771.35558 | -2.03963063 | 1.55E-70 | 3.42E-69 |
| gene-BPHYT_RS02000 | 1751.04179 | 1601.26793 | 1473.22563 | 337.200776 | 338.907484 | 320.503063 | 2.27702468 | 3.05E-70 | 6.69E-69 |
| gene-BPHYT_RS16685 | 3368.80008 | 3232.40627 | 3483.27881 | 10553.7927 | 10899.8628 | 10768.5408 | -1.67583995 | 5.83E-70 | 1.27E-68 |
| gene-BPHYT_RS16705 | 11860.6344 | 13619.8452 | 14525.8623 | 3459.75884 | 3396.55074 | 3148.89732 | 1.999521 | 7.43E-70 | 1.62E-68 |
| gene-BPHYT_RS25830 | 2352.01465 | 2261.61759 | 2273.38337 | 574.818867 | 618.007766 | 605.696466 | 1.9378251 | 7.54E-70 | 1.63E-68 |
| gene-BPHYT_RS12790 | 1472.50323 | 1564.9967 | 1334.95186 | 274.098877 | 256.67258 | 304.206297 | 2.38538282 | 8.72E-70 | 1.88E-68 |
| gene-BPHYT_RS17460 | 134.8797 | 128.01609 | 141.323921 | 818.352761 | 863.466495 | 779.528635 | -2.60493809 | 8.73E-70 | 1.88E-68 |
| gene-BPHYT_RS02005 | 3592.2694 | 3428.69761 | 2126.97585 | 304.663859 | 357.597235 | 338.61058 | 3.19339856 | 6.09E-69 | 1.30E-67 |
| gene-BPHYT_RS09860 | 781.344534 | 1025.19552 | 479.891302 | 12.8175734 | 22.4277012 | 11.7698865 | 5.64046282 | 1.16E-68 | 2.48E-67 |
| gene-BPHYT_RS23640 | 628.90653 | 1033.72993 | 682.218355 | 50.2843263 | 52.3313027 | 47.0795459 | 3.97153852 | 1.35E-68 | 2.88E-67 |
| gene-BPHYT_RS34190 | 64.6464834 | 78.9432555 | 66.0867259 | 580.73467 | 803.659292 | 717.963075 | -3.32951411 | 1.80E-68 | 3.81E-67 |
| gene-BPHYT_RS14190 | 6135.82919 | 7602.02215 | 7779.93271 | 29614.5103 | 29086.2365 | 29205.6152 | -2.03067866 | 1.92E-68 | 4.04E-67 |
| gene-BPHYT_RS32435 | 585.808874 | 619.811236 | 832.692746 | 60.1439981 | 52.3313027 | 57.9440565 | 3.57481152 | 3.22E-68 | 6.77E-67 |
| gene-BPHYT_RS17060 | 12269.2641 | 13101.38 | 11791.9053 | 39222.7605 | 43616.8948 | 41360.2865 | -1.74074722 | 4.44E-68 | 9.27E-67 |
| gene-BPHYT_RS07355 | 271.355609 | 167.487718 | 133.190171 | 2107.99784 | 2956.7186 | 2327.7214 | -3.68427016 | 6.12E-68 | 1.27E-66 |
| gene-BPHYT_RS35480 | 3249.88247 | 3089.45497 | 4048.5745 | 698.064765 | 721.424388 | 645.533005 | 2.33148409 | 7.99E-68 | 1.66E-66 |
| gene-BPHYT_RS23985 | 131.687281 | 77.8764548 | 101.671886 | 994.840887 | 936.979516 | 851.958706 | -3.14874405 | 2.88E-67 | 5.95E-66 |
| gene-BPHYT_RS04315 | 1079.8357 | 1138.2764 | 1146.85887 | 3825.55267 | 3932.32361 | 3713.85187 | -1.76974165 | 2.92E-67 | 6.01E-66 |
| gene-BPHYT_RS15410 | 12767.2814 | 15351.2628 | 17978.6396 | 3271.43911 | 2997.83606 | 2909.87809 | 2.32814069 | 4.33E-67 | 8.88E-66 |
| gene-BPHYT_RS28165 | 552.288475 | 600.608822 | 384.319729 | 14.7895077 | 19.9357344 | 16.2967659 | 4.92768236 | 9.18E-67 | 1.88E-65 |
| gene-BPHYT_RS09465 | 543.509323 | 567.537999 | 491.075209 | 2087.29253 | 2113.18784 | 2179.23975 | -1.99352137 | 1.14E-66 | 2.32E-65 |
| gene-BPHYT_RS29970 | 760.593811 | 747.827326 | 819.475401 | 104.512521 | 105.908589 | 69.713943 | 3.06500231 | 2.70E-66 | 5.47E-65 |
| gene-BPHYT_RS35700 | 28693.4614 | 25859.2502 | 16457.6282 | 2653.23769 | 2981.63827 | 3231.28653 | 3.00164745 | 2.74E-66 | 5.54E-65 |
| gene-BPHYT_RS19415 | 3060.73165 | 3548.1793 | 3778.12728 | 14179.1941 | 13672.1758 | 12737.7333 | -1.96666848 | 3.27E-66 | 6.58E-65 |
| gene-BPHYT_RS17345 | 483.651468 | 464.058326 | 535.810839 | 45.3544904 | 49.8393359 | 46.17417 | 3.39557848 | 4.15E-66 | 8.33E-65 |
| gene-BPHYT_RS05165 | 814.864933 | 1051.86554 | 855.060561 | 3988.23725 | 3853.82665 | 4011.72054 | -2.12420492 | 1.46E-65 | 2.91E-64 |
| gene-BPHYT_RS32425 | 1081.43191 | 1020.92832 | 838.793059 | 150.852979 | 145.780058 | 119.509617 | 2.82555357 | 2.16E-65 | 4.29E-64 |
| gene-BPHYT_RS37480 | 2119.76617 | 2752.34594 | 2646.51919 | 411.148315 | 487.179509 | 356.718098 | 2.58579371 | 3.53E-65 | 6.99E-64 |
| gene-BPHYT_RS09080 | 954.533261 | 1073.20156 | 899.796191 | 3590.89248 | 3659.45324 | 3760.93142 | -1.91177692 | 3.97E-65 | 7.86E-64 |
| gene-BPHYT_RS02705 | 105.349825 | 139.750898 | 144.374078 | 839.058072 | 970.621067 | 861.917841 | -2.78494658 | 7.07E-65 | 1.39E-63 |
| gene-BPHYT_RS17465 | 288.115809 | 304.038214 | 287.731437 | 1267.9538 | 1236.01553 | 1240.36496 | -2.09017638 | 7.92E-65 | 1.55E-63 |
| gene-BPHYT_RS18965 | 398.254262 | 412.85189 | 367.035508 | 1646.56519 | 1811.65986 | 1598.89381 | -2.10107076 | 9.34E-65 | 1.83E-63 |
| gene-BPHYT_RS18030 | 11649.9348 | 14359.1381 | 15624.9354 | 56902.138 | 60896.1926 | 59671.5137 | -2.09185058 | 1.83E-64 | 3.57E-63 |
| gene-BPHYT_RS06880 | 4680.08616 | 5076.90477 | 4848.73224 | 1510.50172 | 1469.01443 | 1516.5046 | 1.69940379 | 1.92E-64 | 3.72E-63 |
| gene-BPHYT_RS03425 | 1059.88309 | 1344.16895 | 1393.92156 | 5888.19601 | 5351.4987 | 5743.7046 | -2.16216884 | 1.98E-64 | 3.83E-63 |
| gene-BPHYT_RS18025 | 1600.19999 | 1854.0997 | 2100.54116 | 7653.07727 | 7493.34416 | 7975.45615 | -2.05821733 | 2.92E-64 | 5.63E-63 |
| gene-BPHYT_RS01850 | 2350.41844 | 2521.91697 | 1730.4555 | 376.639464 | 352.613302 | 344.948211 | 2.61985478 | 6.73E-64 | 1.29E-62 |
| gene-BPHYT_RS16265 | 7361.71806 | 8373.31909 | 8496.71951 | 25911.2176 | 27468.95 | 27815.8633 | -1.74466961 | 7.56E-64 | 1.45E-62 |
| gene-BPHYT_RS28170 | 792.518 | 725.42451 | 461.590362 | 11.8316062 | 19.9357344 | 32.5935318 | 4.92714391 | 1.12E-63 | 2.13E-62 |
| gene-BPHYT_RS29595 | 739.843088 | 802.234164 | 949.615415 | 92.6809151 | 99.6786719 | 124.941872 | 2.96743469 | 1.50E-63 | 2.85E-62 |
| gene-BPHYT_RS18040 | 10105.6021 | 12601.0505 | 14111.0411 | 53423.6458 | 55993.2479 | 56098.9005 | -2.16862618 | 2.19E-63 | 4.14E-62 |
| gene-BPHYT_RS18960 | 319.241893 | 268.833789 | 344.667693 | 1515.43156 | 1576.169 | 1411.481 | -2.26954638 | 5.78E-63 | 1.09E-61 |
| gene-BPHYT_RS04360 | 498.815458 | 429.920702 | 641.5496 | 2861.27676 | 2774.80503 | 2854.65016 | -2.43502365 | 6.22E-63 | 1.17E-61 |
| gene-BPHYT_RS23195 | 624.117901 | 794.766559 | 692.385543 | 95.6388167 | 74.7590039 | 65.1870636 | 3.16329201 | 6.41E-63 | 1.20E-61 |
| gene-BPHYT_RS18000 | 9419.23206 | 10416.2425 | 10371.5491 | 30338.2102 | 33332.5479 | 32631.5576 | -1.67277307 | 8.29E-63 | 1.55E-61 |
| gene-BPHYT_RS34250 | 237.835211 | 266.700188 | 242.995807 | 1085.54987 | 1145.05874 | 1196.00154 | -2.19858559 | 1.17E-62 | 2.18E-61 |
| gene-BPHYT_RS17985 | 15200.7028 | 17585.1436 | 17605.5038 | 55224.0219 | 56145.2579 | 53879.8242 | -1.71347376 | 1.39E-62 | 2.58E-61 |
| gene-BPHYT_RS13300 | 3584.28836 | 3865.01912 | 2078.17335 | 243.533894 | 307.757899 | 339.515956 | 3.41887452 | 1.41E-62 | 2.60E-61 |
| gene-BPHYT_RS23720 | 3213.96776 | 3279.34551 | 2539.76371 | 559.043392 | 669.093085 | 613.844849 | 2.2953451 | 1.48E-62 | 2.72E-61 |
| gene-BPHYT_RS27090 | 5305.00216 | 4969.1579 | 3786.26103 | 581.720638 | 796.183392 | 805.784536 | 2.68747414 | 1.81E-62 | 3.32E-61 |
| gene-BPHYT_RS24525 | 1591.42084 | 1416.7114 | 1118.39075 | 226.772452 | 188.143493 | 212.763333 | 2.71462023 | 2.89E-62 | 5.29E-61 |
| gene-BPHYT_RS09850 | 6842.94998 | 7101.69259 | 3811.679 | 26.6211139 | 42.3634355 | 15.39139 | 7.73382217 | 4.04E-62 | 7.36E-61 |
| gene-BPHYT_RS18035 | 4530.04247 | 5488.68986 | 6266.03833 | 23137.6919 | 22965.966 | 24553.794 | -2.11760935 | 2.60E-61 | 4.73E-60 |
| gene-BPHYT_RS19675 | 1122.93336 | 1136.1428 | 1188.54435 | 175.502158 | 190.63546 | 243.546113 | 2.49523831 | 2.75E-61 | 4.97E-60 |
| gene-BPHYT_RS20260 | 69.4351118 | 72.542451 | 164.708455 | 20707.2828 | 18091.6789 | 15468.347 | -7.46890902 | 5.11E-61 | 9.21E-60 |
| gene-BPHYT_RS18020 | 1162.8386 | 1264.15889 | 1449.84109 | 4720.81087 | 5270.50978 | 5254.80163 | -1.97615874 | 7.69E-61 | 1.38E-59 |
| gene-BPHYT_RS15580 | 1298.5164 | 1622.60394 | 1513.89438 | 5517.47235 | 5418.7818 | 5792.5949 | -1.91644987 | 8.68E-61 | 1.56E-59 |
| gene-BPHYT_RS03240 | 1654.47111 | 1643.93996 | 1463.05844 | 388.47107 | 402.452638 | 363.055729 | 2.04668265 | 2.35E-60 | 4.19E-59 |
| gene-BPHYT_RS07555 | 3186.8322 | 3168.39823 | 3663.23805 | 922.865283 | 934.487549 | 935.253287 | 1.84288697 | 2.51E-60 | 4.46E-59 |
| gene-BPHYT_RS14790 | 1246.63959 | 1607.66873 | 1353.2528 | 6143.56151 | 5473.60507 | 5592.50683 | -2.03319504 | 4.07E-60 | 7.23E-59 |
| gene-BPHYT_RS23240 | 9749.64742 | 9205.42367 | 9362.96398 | 27111.1396 | 26389.9284 | 26197.9566 | -1.49277185 | 7.07E-60 | 1.25E-58 |
| gene-BPHYT_RS31015 | 67.0407976 | 88.5444623 | 53.8860995 | 568.903064 | 590.596131 | 607.507218 | -3.08126785 | 7.51E-60 | 1.32E-58 |
| gene-BPHYT_RS34985 | 639.281891 | 720.090506 | 815.408525 | 99.5826854 | 100.924655 | 114.982737 | 2.78092978 | 9.04E-60 | 1.59E-58 |
| gene-BPHYT_RS14750 | 10886.1486 | 11974.8384 | 11641.4309 | 33471.6139 | 32662.2088 | 33488.0432 | -1.52986601 | 2.17E-59 | 3.80E-58 |
| gene-BPHYT_RS17995 | 14238.9865 | 14894.6721 | 14254.3984 | 40705.6551 | 43527.184 | 40307.3343 | -1.52123945 | 2.22E-59 | 3.87E-58 |
| gene-BPHYT_RS07650 | 591.395607 | 667.81727 | 539.877714 | 82.8212433 | 67.2831035 | 82.3892054 | 2.94377743 | 3.06E-59 | 5.33E-58 |
| gene-BPHYT_RS34115 | 1013.59301 | 945.185465 | 948.598696 | 3356.23229 | 3519.9031 | 4080.52911 | -1.91373513 | 9.82E-59 | 1.70E-57 |
| gene-BPHYT_RS18095 | 2083.85146 | 2867.56042 | 2866.13046 | 11457.9246 | 12584.4323 | 11668.4844 | -2.19219629 | 1.61E-58 | 2.78E-57 |
| gene-BPHYT_RS24065 | 588.203188 | 539.80118 | 467.690675 | 36.4807857 | 47.3473691 | 61.56556 | 3.45024984 | 1.83E-58 | 3.15E-57 |
| gene-BPHYT_RS11150 | 1573.86253 | 1667.40957 | 1462.04172 | 402.27461 | 357.597235 | 334.083701 | 2.10427116 | 2.12E-58 | 3.65E-57 |
| gene-BPHYT_RS16570 | 964.110518 | 884.377822 | 937.414788 | 3456.80094 | 3043.93744 | 3603.39601 | -1.8586404 | 2.49E-58 | 4.27E-57 |
| gene-BPHYT_RS34525 | 57.4635408 | 81.076857 | 57.952975 | 717.784109 | 1711.98119 | 1165.21876 | -4.19748498 | 4.09E-58 | 6.99E-57 |
| gene-BPHYT_RS18970 | 494.02683 | 410.718289 | 493.108647 | 1858.54814 | 2270.18175 | 2070.59464 | -2.14696231 | 4.54E-58 | 7.73E-57 |
| gene-BPHYT_RS11400 | 4593.89084 | 5227.32368 | 4398.32579 | 15007.4065 | 15511.2473 | 15958.1553 | -1.70879046 | 6.88E-58 | 1.17E-56 |
| gene-BPHYT_RS06900 | 4369.62341 | 4853.94341 | 4549.8169 | 13480.1433 | 13510.198 | 14436.2185 | -1.58891619 | 7.30E-58 | 1.23E-56 |
| gene-BPHYT_RS05495 | 176.381146 | 234.696165 | 249.096121 | 1344.85924 | 1156.27259 | 1213.20368 | -2.4980088 | 7.39E-58 | 1.25E-56 |
| gene-BPHYT_RS01435 | 5772.69154 | 5990.08621 | 5172.04884 | 1554.87025 | 1746.86872 | 1537.32825 | 1.80800915 | 8.99E-58 | 1.51E-56 |
| gene-BPHYT_RS31080 | 0 | 4.267203 | 6.10031316 | 660.598012 | 619.253749 | 763.231869 | -7.74824183 | 9.22E-58 | 1.55E-56 |
| gene-BPHYT_RS35170 | 22.3469325 | 16.0020113 | 38.6353167 | 606.369817 | 470.981725 | 406.513772 | -4.27206782 | 1.73E-57 | 2.89E-56 |
| gene-BPHYT_RS18150 | 66496.4902 | 67516.7527 | 69724.5459 | 183885.837 | 195589.49 | 183444.545 | -1.4662156 | 1.89E-57 | 3.15E-56 |
| gene-BPHYT_RS24705 | 401.446681 | 504.596755 | 405.670825 | 4.92983591 | 8.72188379 | 5.4322553 | 6.14277913 | 2.73E-57 | 4.53E-56 |
| gene-BPHYT_RS14935 | 89562.5151 | 81998.5729 | 87243.6286 | 237799.509 | 248979.879 | 238653.461 | -1.48696586 | 4.68E-57 | 7.74E-56 |
| gene-BPHYT_RS34140 | 788.527476 | 820.369777 | 498.192241 | 64.0878669 | 56.0692529 | 42.5526665 | 3.70031303 | 6.54E-57 | 1.08E-55 |
| gene-BPHYT_RS19010 | 178.77546 | 202.692143 | 208.427366 | 1079.63407 | 1336.94019 | 984.143585 | -2.52847083 | 6.64E-57 | 1.09E-55 |
| gene-BPHYT_RS35175 | 3229.13175 | 3055.31735 | 2149.34367 | 518.618738 | 458.521891 | 496.145984 | 2.51650465 | 7.74E-57 | 1.27E-55 |
| gene-BPHYT_RS23165 | 487.641992 | 518.465165 | 514.459743 | 59.1580309 | 72.2670371 | 74.2408224 | 2.88704782 | 1.26E-56 | 2.06E-55 |
| gene-BPHYT_RS30830 | 1443.77146 | 1312.16492 | 1517.96126 | 365.793825 | 331.431584 | 359.434226 | 2.01450795 | 5.36E-56 | 8.74E-55 |
| gene-BPHYT_RS20505 | 1412.64538 | 1250.29048 | 1133.64153 | 262.267271 | 287.822165 | 277.950396 | 2.19885831 | 7.54E-56 | 1.22E-54 |
| gene-BPHYT_RS06030 | 4022.44786 | 3838.3491 | 5127.31321 | 16759.4702 | 17111.09 | 17221.1547 | -1.97599271 | 1.09E-55 | 1.76E-54 |
| gene-BPHYT_RS12135 | 6581.17163 | 7271.31391 | 8846.4708 | 28928.2771 | 28587.8431 | 29481.7549 | -1.938515 | 1.37E-55 | 2.21E-54 |
| gene-BPHYT_RS17990 | 21873.6564 | 25434.6635 | 24915.7124 | 73057.2103 | 72974.7557 | 71282.9594 | -1.58929502 | 2.20E-55 | 3.54E-54 |
| gene-BPHYT_RS33800 | 968.101041 | 1118.00719 | 1070.60496 | 216.91278 | 251.688647 | 212.763333 | 2.21540382 | 3.40E-55 | 5.46E-54 |
| gene-BPHYT_RS18565 | 3329.69295 | 3323.08434 | 3638.8368 | 9861.64376 | 9666.3392 | 10043.3347 | -1.52287509 | 7.58E-55 | 1.21E-53 |
| gene-BPHYT_RS34130 | 99.7630917 | 100.279271 | 116.922669 | 732.573616 | 1061.57786 | 717.963075 | -2.98597382 | 9.47E-55 | 1.51E-53 |
| gene-BPHYT_RS20000 | 3036.78851 | 3409.4952 | 4265.13562 | 808.493089 | 743.852089 | 670.883529 | 2.26849895 | 2.13E-54 | 3.39E-53 |
| gene-BPHYT_RS14575 | 107.744139 | 88.5444623 | 110.822356 | 596.510145 | 767.525773 | 654.586763 | -2.71172049 | 2.62E-54 | 4.16E-53 |
| gene-BPHYT_RS04300 | 1818.08258 | 2100.53068 | 2090.37398 | 6772.60858 | 6598.72808 | 6114.00334 | -1.69773469 | 6.95E-54 | 1.10E-52 |
| gene-BPHYT_RS34980 | 598.57855 | 735.025717 | 730.004141 | 115.35816 | 77.2509707 | 82.3892054 | 2.901428 | 1.03E-53 | 1.63E-52 |
| gene-BPHYT_RS18175 | 7838.98469 | 9443.32024 | 10170.2388 | 32359.4429 | 35960.3269 | 32272.1234 | -1.87363492 | 1.44E-53 | 2.27E-52 |
| gene-BPHYT_RS35240 | 368.724387 | 409.651488 | 398.553793 | 1371.48035 | 1498.91803 | 1454.03367 | -1.87881182 | 1.49E-53 | 2.34E-52 |
| gene-BPHYT_RS10920 | 4247.51339 | 3925.82676 | 2483.84417 | 487.067788 | 412.420505 | 557.711544 | 2.8688279 | 1.74E-53 | 2.72E-52 |
| gene-BPHYT_RS35110 | 1029.55511 | 950.519469 | 847.943529 | 108.45639 | 178.175626 | 136.711758 | 2.7493537 | 2.26E-53 | 3.53E-52 |
| gene-BPHYT_RS21415 | 2186.80697 | 2238.14797 | 2181.87867 | 670.457684 | 716.440454 | 705.287813 | 1.65934518 | 3.37E-53 | 5.23E-52 |
| gene-BPHYT_RS16270 | 5758.32565 | 6088.23188 | 5643.80639 | 15738.9941 | 15578.5304 | 16029.68 | -1.43680509 | 3.41E-53 | 5.28E-52 |
| gene-BPHYT_RS16490 | 397.456157 | 344.576642 | 338.56738 | 1370.49438 | 1365.59781 | 1385.2251 | -1.92862303 | 3.94E-53 | 6.09E-52 |
| gene-BPHYT_RS15535 | 889.088673 | 883.311021 | 896.746034 | 2909.58916 | 2797.23273 | 2624.68469 | -1.64204828 | 3.99E-53 | 6.14E-52 |
| gene-BPHYT_RS34900 | 1050.30583 | 732.892115 | 803.207899 | 123.245898 | 138.304157 | 131.279503 | 2.72251581 | 4.29E-53 | 6.60E-52 |
| gene-BPHYT_RS05375 | 2803.74193 | 2909.16565 | 2758.35827 | 7962.67096 | 7553.15136 | 8002.61743 | -1.47332442 | 4.84E-53 | 7.42E-52 |
| gene-BPHYT_RS06025 | 611.348226 | 601.675623 | 675.101323 | 2423.50733 | 2191.6848 | 2099.56667 | -1.83068585 | 5.55E-53 | 8.48E-52 |
| gene-BPHYT_RS18110 | 9001.02518 | 11959.9032 | 12800.4904 | 45516.189 | 46225.9841 | 46920.1998 | -2.03828531 | 1.72E-52 | 2.62E-51 |
| gene-BPHYT_RS02730 | 1204.34004 | 1321.76613 | 1092.97277 | 263.253238 | 301.527982 | 277.950396 | 2.10449418 | 1.90E-52 | 2.89E-51 |
| gene-BPHYT_RS05220 | 96.5706727 | 73.6092518 | 141.323921 | 778.914074 | 946.947383 | 1065.62741 | -3.16408939 | 2.09E-52 | 3.16E-51 |
| gene-BPHYT_RS14975 | 1087.81675 | 973.989085 | 918.09713 | 192.263601 | 201.849311 | 234.492354 | 2.24332106 | 4.35E-52 | 6.57E-51 |
| gene-BPHYT_RS01755 | 1185.18553 | 1106.27238 | 1177.36044 | 254.379533 | 235.490862 | 303.300921 | 2.12439631 | 6.82E-52 | 1.03E-50 |
| gene-BPHYT_RS01640 | 7099.1416 | 7341.72276 | 7016.37685 | 19170.1599 | 18243.6889 | 19239.2375 | -1.40076487 | 9.05E-52 | 1.36E-50 |
| gene-BPHYT_RS00010 | 12233.3494 | 14753.8544 | 13927.0149 | 40546.9144 | 43306.645 | 41541.3616 | -1.61590435 | 1.81E-51 | 2.71E-50 |
| gene-BPHYT_RS18715 | 8127.1005 | 9027.26795 | 9191.13849 | 24308.0349 | 27140.0104 | 26275.8189 | -1.56089892 | 1.91E-51 | 2.85E-50 |
| gene-BPHYT_RS06965 | 202.718602 | 110.947278 | 154.541267 | 1124.98856 | 1025.44434 | 1089.16719 | -2.78175474 | 1.97E-51 | 2.93E-50 |
| gene-BPHYT_RS18005 | 731.063936 | 754.22813 | 818.458682 | 2360.40543 | 2615.31915 | 2539.57935 | -1.7062586 | 2.02E-51 | 3.00E-50 |
| gene-BPHYT_RS02490 | 2037.56138 | 1917.04095 | 1716.22143 | 5680.15694 | 5960.78458 | 6071.45067 | -1.64264788 | 2.48E-51 | 3.67E-50 |
| gene-BPHYT_RS03415 | 4372.81583 | 4640.58326 | 4261.06874 | 13094.6302 | 12793.7575 | 11825.1144 | -1.50645164 | 3.10E-51 | 4.58E-50 |
| gene-BPHYT_RS04265 | 1116.54852 | 1200.15084 | 1206.84529 | 3369.04986 | 3511.18122 | 3417.79396 | -1.54777047 | 5.51E-51 | 8.12E-50 |
| gene-BPHYT_RS03360 | 3660.10831 | 4521.10158 | 3664.25477 | 1104.28324 | 1123.87703 | 1016.73712 | 1.86840412 | 8.65E-51 | 1.27E-49 |
| gene-BPHYT_RS34975 | 635.291368 | 689.153285 | 817.441963 | 134.091537 | 120.86039 | 124.036496 | 2.49625458 | 1.03E-50 | 1.51E-49 |
| gene-BPHYT_RS07400 | 35.914713 | 51.206436 | 29.4848469 | 487.067788 | 407.436571 | 370.298736 | -3.44722769 | 1.57E-50 | 2.30E-49 |
| gene-BPHYT_RS09855 | 2840.45475 | 2680.87029 | 1657.25174 | 27.6070811 | 9.96786719 | 11.7698865 | 7.17393365 | 2.42E-50 | 3.53E-49 |
| gene-BPHYT_RS11335 | 633.695158 | 640.08045 | 506.325992 | 57.1860966 | 92.2027715 | 79.6730777 | 2.96720265 | 4.67E-50 | 6.80E-49 |
| gene-BPHYT_RS21345 | 2329.66772 | 2640.33186 | 2982.03641 | 738.48942 | 702.734637 | 713.436196 | 1.88315386 | 4.74E-50 | 6.86E-49 |
| gene-BPHYT_RS05500 | 246.614363 | 221.894556 | 231.8119 | 1144.7079 | 912.059848 | 1063.81666 | -2.15501477 | 4.74E-50 | 6.86E-49 |
| gene-BPHYT_RS18995 | 275.346133 | 362.712255 | 325.350035 | 1462.18933 | 1875.20502 | 1444.07453 | -2.31331618 | 4.95E-50 | 7.14E-49 |
| gene-BPHYT_RS21270 | 1442.17525 | 1326.03333 | 1373.58718 | 397.344774 | 363.827152 | 325.935318 | 1.93107987 | 5.54E-50 | 7.97E-49 |
| gene-BPHYT_RS12825 | 14461.6578 | 15397.1352 | 12350.084 | 42762.3827 | 45512.0356 | 45516.8671 | -1.66435633 | 1.02E-49 | 1.46E-48 |
| gene-BPHYT_RS12785 | 3550.76796 | 3552.4465 | 4149.22967 | 1208.79577 | 1106.43326 | 1116.32846 | 1.71290684 | 1.16E-49 | 1.66E-48 |
| gene-BPHYT_RS04280 | 782.142639 | 916.381844 | 885.562126 | 2670.9851 | 2891.92747 | 2980.49741 | -1.72644512 | 1.26E-49 | 1.80E-48 |
| gene-BPHYT_RS18130 | 16892.6848 | 22488.1598 | 20423.8485 | 68061.3146 | 72239.6255 | 68222.7889 | -1.80196321 | 1.36E-49 | 1.94E-48 |
| gene-BPHYT_RS24675 | 1039.13236 | 1372.97257 | 733.054298 | 125.217832 | 122.106373 | 118.604241 | 3.1035022 | 1.41E-49 | 2.00E-48 |
| gene-BPHYT_RS08665 | 474.074212 | 432.054304 | 301.965501 | 30.5649827 | 23.6736846 | 30.78278 | 3.8179582 | 1.79E-49 | 2.54E-48 |
| gene-BPHYT_RS00120 | 634.493263 | 678.485277 | 433.122234 | 2747.89054 | 2976.65434 | 2949.71463 | -2.31201516 | 1.97E-49 | 2.79E-48 |
| gene-BPHYT_RS03660 | 663.225033 | 517.398364 | 578.513031 | 81.8352761 | 99.6786719 | 66.9978153 | 2.83700899 | 2.53E-49 | 3.56E-48 |
| gene-BPHYT_RS21705 | 544.307428 | 532.333574 | 570.37928 | 71.9756043 | 73.5130205 | 103.212851 | 2.71792997 | 4.41E-49 | 6.19E-48 |
| gene-BPHYT_RS18100 | 15505.5788 | 24223.8446 | 23216.7752 | 95801.5013 | 102246.644 | 100296.635 | -2.2448758 | 7.12E-49 | 9.98E-48 |
| gene-BPHYT_RS02725 | 885.098149 | 762.762536 | 1207.86201 | 117.330095 | 103.416622 | 60.6601842 | 3.34977826 | 8.51E-49 | 1.19E-47 |
| gene-BPHYT_RS18155 | 61123.6491 | 69198.0307 | 66530.0153 | 175195.523 | 179076.472 | 172070.308 | -1.41890866 | 1.26E-48 | 1.76E-47 |
| gene-BPHYT_RS01750 | 960.918099 | 1023.06192 | 1101.10653 | 145.923143 | 159.485875 | 227.249347 | 2.52814018 | 1.28E-48 | 1.79E-47 |
| gene-BPHYT_RS16395 | 414.216357 | 521.665567 | 522.593494 | 70.0036699 | 42.3634355 | 54.322553 | 3.11604382 | 1.30E-48 | 1.81E-47 |
| gene-BPHYT_RS24920 | 1755.83041 | 1770.88925 | 2094.44085 | 360.863989 | 490.917459 | 444.539559 | 2.1184728 | 3.81E-48 | 5.26E-47 |
| gene-BPHYT_RS30825 | 4534.03299 | 4131.71931 | 4811.11364 | 1139.77806 | 1373.07371 | 1393.37348 | 1.78697521 | 4.00E-48 | 5.52E-47 |
| gene-BPHYT_RS33915 | 5015.29014 | 5288.13132 | 4896.51803 | 12915.1841 | 14139.4196 | 14128.3907 | -1.43801483 | 5.98E-48 | 8.23E-47 |
| gene-BPHYT_RS19015 | 288.913913 | 329.641432 | 441.255985 | 1999.54145 | 2545.54408 | 1776.34748 | -2.57786361 | 7.14E-48 | 9.80E-47 |
| gene-BPHYT_RS26210 | 1314.4785 | 1420.9786 | 1098.05637 | 221.842616 | 284.084215 | 289.720283 | 2.26944809 | 1.58E-47 | 2.17E-46 |
| gene-BPHYT_RS20295 | 477.26663 | 204.825744 | 323.316597 | 12017.954 | 10927.2744 | 9869.5025 | -5.02666501 | 2.11E-47 | 2.88E-46 |
| gene-BPHYT_RS11440 | 83.800997 | 78.9432555 | 101.671886 | 541.295983 | 513.34516 | 482.565346 | -2.54045837 | 2.18E-47 | 2.96E-46 |
| gene-BPHYT_RS02980 | 1561.09286 | 1825.29608 | 1756.89019 | 5054.06778 | 5436.22557 | 4966.89209 | -1.58805119 | 3.28E-47 | 4.45E-46 |
| gene-BPHYT_RS11175 | 1664.84647 | 1777.29005 | 1871.77942 | 4795.74437 | 5099.81005 | 5068.29419 | -1.49403754 | 3.75E-47 | 5.08E-46 |
| gene-BPHYT_RS18710 | 616.136854 | 521.665567 | 630.365693 | 1902.91666 | 2138.10751 | 2104.99893 | -1.79611963 | 9.78E-47 | 1.32E-45 |
| gene-BPHYT_RS04310 | 1507.61984 | 1731.41762 | 1830.09395 | 5182.24351 | 5144.66545 | 5035.70066 | -1.60036261 | 1.04E-46 | 1.41E-45 |
| gene-BPHYT_RS14905 | 2458.16258 | 2845.1576 | 2586.53278 | 7463.77157 | 7332.6123 | 8061.46686 | -1.53513397 | 1.19E-46 | 1.61E-45 |
| gene-BPHYT_RS34195 | 1119.74094 | 1039.06393 | 788.973835 | 194.235535 | 188.143493 | 190.128935 | 2.36467054 | 1.71E-46 | 2.29E-45 |
| gene-BPHYT_RS16455 | 3914.70372 | 3600.45253 | 2841.72921 | 913.005611 | 875.926329 | 903.565131 | 1.94360562 | 2.25E-46 | 3.02E-45 |
| gene-BPHYT_RS20520 | 400.648576 | 342.443041 | 387.369885 | 1309.36442 | 1275.887 | 1395.18424 | -1.81459853 | 2.27E-46 | 3.03E-45 |
| gene-BPHYT_RS34960 | 408.629623 | 355.24465 | 444.306142 | 57.1860966 | 53.5772861 | 52.5118012 | 2.88781017 | 4.11E-46 | 5.48E-45 |
| gene-BPHYT_RS07745 | 1193.96468 | 1056.13274 | 1110.25699 | 3182.70206 | 3365.40116 | 3613.35515 | -1.5957365 | 4.54E-46 | 6.03E-45 |
| gene-BPHYT_RS18045 | 6380.04924 | 9171.28605 | 9827.60449 | 36404.8663 | 36390.1911 | 36418.7449 | -2.10566025 | 5.52E-46 | 7.32E-45 |
| gene-BPHYT_RS37310 | 396.658052 | 481.127138 | 287.731437 | 29.5790155 | 14.9518008 | 27.1612765 | 3.99682729 | 7.21E-46 | 9.53E-45 |
| gene-BPHYT_RS18050 | 4604.26621 | 6526.68699 | 6839.46777 | 24259.7225 | 25267.2973 | 24245.0608 | -2.03773696 | 9.01E-46 | 1.19E-44 |
| gene-BPHYT_RS04365 | 537.124485 | 509.930759 | 584.613344 | 2151.38039 | 1827.85765 | 1787.21199 | -1.82146922 | 1.24E-45 | 1.63E-44 |
| gene-BPHYT_RS01760 | 1764.60957 | 1312.16492 | 1560.66345 | 383.541234 | 373.79502 | 390.217006 | 2.01505417 | 1.33E-45 | 1.75E-44 |
| gene-BPHYT_RS04795 | 9197.35895 | 9099.8104 | 7888.72163 | 23914.634 | 23936.5871 | 25381.3075 | -1.48364879 | 1.52E-45 | 1.98E-44 |
| gene-BPHYT_RS12945 | 26384.5444 | 25882.7198 | 21168.0867 | 72452.8124 | 82565.0899 | 79500.1509 | -1.6751168 | 1.61E-45 | 2.10E-44 |
| gene-BPHYT_RS04355 | 183.564089 | 195.224537 | 280.614405 | 1116.11485 | 1077.77564 | 1218.63594 | -2.37517883 | 1.83E-45 | 2.38E-44 |
| gene-BPHYT_RS28245 | 276.942342 | 270.967391 | 236.895494 | 9.85967182 | 8.72188379 | 13.5806382 | 4.5851417 | 2.25E-45 | 2.92E-44 |
| gene-BPHYT_RS05540 | 782.142639 | 784.098551 | 867.261187 | 2341.67206 | 2357.40059 | 2387.4762 | -1.54250089 | 2.45E-45 | 3.18E-44 |
| gene-BPHYT_RS20020 | 956.927575 | 1143.6104 | 890.645721 | 157.754749 | 144.534074 | 219.100964 | 2.51292482 | 2.96E-45 | 3.83E-44 |
| gene-BPHYT_RS01680 | 752.612763 | 936.651059 | 834.726184 | 140.993307 | 68.5290869 | 120.414992 | 2.92220559 | 3.26E-45 | 4.20E-44 |
| gene-BPHYT_RS18285 | 950.542737 | 978.256288 | 811.34165 | 3077.20358 | 3334.25157 | 2812.09749 | -1.75040224 | 4.06E-45 | 5.22E-44 |
| gene-BPHYT_RS35715 | 483.651468 | 312.57262 | 306.032377 | 20.7053108 | 24.919668 | 28.0666524 | 3.90323862 | 5.08E-45 | 6.53E-44 |
| gene-BPHYT_RS13305 | 1347.99889 | 1400.70939 | 925.214162 | 83.8072105 | 84.7268711 | 173.83217 | 3.41411162 | 6.21E-45 | 7.96E-44 |
| gene-BPHYT_RS18905 | 532.335857 | 494.995548 | 493.108647 | 1864.46394 | 2564.23383 | 1921.20762 | -2.06057156 | 8.14E-45 | 1.04E-43 |
| gene-BPHYT_RS18685 | 761.391916 | 833.171386 | 1013.6687 | 3097.90889 | 3146.10808 | 2937.94474 | -1.81671453 | 1.27E-44 | 1.62E-43 |
| gene-BPHYT_RS03535 | 1139.69356 | 1175.61443 | 1353.2528 | 4157.82361 | 5481.08097 | 4255.26665 | -1.92114139 | 3.03E-44 | 3.86E-43 |
| gene-BPHYT_RS12470 | 1129.3182 | 1377.23977 | 1199.72825 | 304.663859 | 305.265933 | 229.965474 | 2.14459555 | 3.42E-44 | 4.34E-43 |
| gene-BPHYT_RS28070 | 504.402191 | 365.912657 | 354.834882 | 1660.36874 | 1802.93798 | 1829.76466 | -2.10722851 | 3.57E-44 | 4.52E-43 |
| gene-BPHYT_RS19025 | 3151.71559 | 3258.00949 | 3345.00505 | 8789.89743 | 8087.67824 | 8213.57001 | -1.36320946 | 3.98E-44 | 5.03E-43 |
| gene-BPHYT_RS09090 | 272.153714 | 320.040225 | 314.166128 | 1127.94646 | 1131.35293 | 1030.31776 | -1.86213584 | 4.28E-44 | 5.40E-43 |
| gene-BPHYT_RS28345 | 359.945235 | 348.843845 | 353.818163 | 47.3264248 | 54.8232695 | 50.7010495 | 2.80236243 | 5.66E-44 | 7.12E-43 |
| gene-BPHYT_RS15085 | 664.821243 | 650.748458 | 460.573643 | 84.7931777 | 98.4326885 | 84.1999571 | 2.73733569 | 7.37E-44 | 9.25E-43 |
| gene-BPHYT_RS35725 | 45.4919698 | 24.5364173 | 25.4179715 | 473.264248 | 333.923551 | 357.623474 | -3.58422644 | 1.27E-43 | 1.59E-42 |
| gene-BPHYT_RS07615 | 1853.19919 | 1722.88321 | 1562.69689 | 522.562607 | 545.740729 | 524.212636 | 1.69125715 | 1.57E-43 | 1.96E-42 |
| gene-BPHYT_RS26370 | 1940.99071 | 1460.45023 | 919.113849 | 158.740716 | 169.453742 | 102.307475 | 3.33298982 | 1.61E-43 | 2.01E-42 |
| gene-BPHYT_RS09660 | 3029.60557 | 3020.11292 | 2876.29765 | 1113.15695 | 1128.86096 | 1108.18008 | 1.41403019 | 1.67E-43 | 2.08E-42 |
| gene-BPHYT_RS24550 | 9721.71376 | 7258.51231 | 3478.19522 | 113.386226 | 89.7108047 | 97.7805954 | 6.08658818 | 1.88E-43 | 2.33E-42 |
| gene-BPHYT_RS34830 | 847.587227 | 912.114641 | 919.113849 | 232.688255 | 159.485875 | 194.655815 | 2.18459982 | 2.19E-43 | 2.70E-42 |
| gene-BPHYT_RS32430 | 619.329273 | 596.341619 | 530.727245 | 112.400259 | 69.7750703 | 66.0924395 | 2.81133392 | 3.22E-43 | 3.97E-42 |
| gene-BPHYT_RS33775 | 4043.19858 | 4383.48428 | 3848.28088 | 1449.37176 | 1523.8377 | 1483.0057 | 1.46199928 | 3.39E-43 | 4.18E-42 |
| gene-BPHYT_RS16450 | 1523.58194 | 1339.90174 | 1128.55793 | 301.705958 | 322.7097 | 344.042836 | 2.0436571 | 5.19E-43 | 6.38E-42 |
| gene-BPHYT_RS04305 | 1281.7562 | 1385.77418 | 1434.59031 | 3958.65824 | 4359.69591 | 3724.71638 | -1.5539263 | 5.27E-43 | 6.46E-42 |
| gene-BPHYT_RS34950 | 1148.47271 | 890.778626 | 929.281037 | 188.319732 | 214.309145 | 240.829985 | 2.20585414 | 5.44E-43 | 6.65E-42 |
| gene-BPHYT_RS19095 | 46.2900745 | 36.2712255 | 46.7690675 | 327.341105 | 409.928538 | 323.21919 | -3.02699702 | 6.28E-43 | 7.66E-42 |
| gene-BPHYT_RS18105 | 4020.85165 | 6086.09828 | 5971.18986 | 22076.7912 | 23835.6624 | 21535.2708 | -2.06898001 | 6.77E-43 | 8.24E-42 |
| gene-BPHYT_RS33605 | 1778.17735 | 1662.07557 | 1535.24548 | 4499.95422 | 5276.73969 | 5097.26622 | -1.5792576 | 8.25E-43 | 1.00E-41 |
| gene-BPHYT_RS04270 | 383.090272 | 537.667578 | 488.025053 | 1829.95509 | 1866.48313 | 1728.36256 | -1.94811653 | 8.50E-43 | 1.03E-41 |
| gene-BPHYT_RS14865 | 1483.6767 | 1583.13231 | 1714.188 | 4264.30806 | 4456.88262 | 4365.72251 | -1.45320615 | 1.04E-42 | 1.26E-41 |
| gene-BPHYT_RS12875 | 3405.5129 | 3597.25213 | 2513.32902 | 11506.237 | 12311.562 | 11376.9534 | -1.88676944 | 1.44E-42 | 1.73E-41 |
| gene-BPHYT_RS12055 | 10483.1057 | 12127.3909 | 12200.6263 | 30359.9015 | 33003.6083 | 31260.8185 | -1.44275896 | 1.51E-42 | 1.82E-41 |
| gene-BPHYT_RS16510 | 4731.16486 | 5108.90879 | 5284.90463 | 1631.77569 | 1695.78341 | 1318.22729 | 1.70380779 | 2.13E-42 | 2.56E-41 |
| gene-BPHYT_RS16800 | 1399.8757 | 1504.18906 | 1356.30296 | 3759.49287 | 3745.4261 | 3722.00026 | -1.39816541 | 2.24E-42 | 2.69E-41 |
| gene-BPHYT_RS18125 | 2726.32577 | 3124.6594 | 2596.69997 | 7965.62887 | 7858.41729 | 7745.49068 | -1.48053343 | 2.81E-42 | 3.36E-41 |
| gene-BPHYT_RS23695 | 6633.04844 | 5759.65725 | 4220.39999 | 1082.59197 | 1272.14905 | 1313.70041 | 2.17937193 | 3.20E-42 | 3.82E-41 |
| gene-BPHYT_RS14050 | 198.728079 | 208.026146 | 174.875644 | 879.482727 | 726.408321 | 924.388777 | -2.1224328 | 3.43E-42 | 4.08E-41 |
| gene-BPHYT_RS07600 | 913.82992 | 793.699758 | 666.967572 | 2728.17119 | 2754.86929 | 2755.96419 | -1.79324754 | 4.02E-42 | 4.78E-41 |
| gene-BPHYT_RS04275 | 594.588026 | 773.430544 | 715.770077 | 2237.15954 | 2685.09422 | 2539.57935 | -1.84192597 | 4.75E-42 | 5.63E-41 |
| gene-BPHYT_RS16495 | 577.827827 | 652.882059 | 736.104454 | 2119.82944 | 2103.21998 | 2352.16654 | -1.74303305 | 8.35E-42 | 9.87E-41 |
| gene-BPHYT_RS14420 | 8554.08653 | 8569.61043 | 9328.39553 | 23967.8762 | 21789.7577 | 21831.3287 | -1.35346243 | 1.09E-41 | 1.29E-40 |
| gene-BPHYT_RS08560 | 471.679897 | 695.554089 | 821.508838 | 3153.12305 | 3217.12914 | 3356.2284 | -2.29234604 | 1.46E-41 | 1.72E-40 |
| gene-BPHYT_RS02545 | 3664.89694 | 2871.82762 | 1839.24442 | 299.734023 | 428.618289 | 420.09441 | 2.86779062 | 1.53E-41 | 1.80E-40 |
| gene-BPHYT_RS07735 | 342.386931 | 357.378251 | 351.784725 | 1109.21308 | 1106.43326 | 1256.66173 | -1.724741 | 1.92E-41 | 2.26E-40 |
| gene-BPHYT_RS19005 | 233.844687 | 234.696165 | 219.611274 | 842.015974 | 1171.22439 | 978.71133 | -2.11855907 | 2.42E-41 | 2.84E-40 |
| gene-BPHYT_RS01765 | 10805.54 | 9574.53673 | 9605.95978 | 3324.68134 | 3649.48537 | 3842.41525 | 1.47112503 | 2.43E-41 | 2.84E-40 |
| gene-BPHYT_RS03090 | 2814.11729 | 2403.50209 | 2361.83791 | 688.205093 | 819.857076 | 797.636153 | 1.71807035 | 2.54E-41 | 2.96E-40 |
| gene-BPHYT_RS14860 | 600.174759 | 577.139206 | 636.466006 | 1954.18696 | 1689.55349 | 1867.79045 | -1.60400022 | 4.70E-41 | 5.47E-40 |
| gene-BPHYT_RS02830 | 475.670421 | 409.651488 | 470.740832 | 1488.81045 | 1360.61387 | 1484.81645 | -1.67545766 | 4.94E-41 | 5.73E-40 |
| gene-BPHYT_RS04440 | 605.761493 | 625.14524 | 631.382412 | 1894.04296 | 1711.98119 | 1737.41632 | -1.5213847 | 1.33E-40 | 1.54E-39 |
| gene-BPHYT_RS08030 | 6837.36325 | 8375.45269 | 5998.64127 | 2026.16256 | 1473.99836 | 1593.46155 | 2.05753899 | 1.34E-40 | 1.55E-39 |
| gene-BPHYT_RS18705 | 1379.12498 | 1474.31864 | 1662.33534 | 4103.59541 | 4323.56239 | 4468.93536 | -1.51449545 | 1.47E-40 | 1.69E-39 |
| gene-BPHYT_RS09085 | 296.894961 | 295.503808 | 220.627993 | 1025.40587 | 1075.28367 | 1062.91129 | -1.95827634 | 3.02E-40 | 3.48E-39 |
| gene-BPHYT_RS19580 | 21182.4977 | 19210.9479 | 18882.5027 | 48768.8947 | 47162.9636 | 49759.4585 | -1.29734933 | 3.13E-40 | 3.59E-39 |
| gene-BPHYT_RS12125 | 3593.06751 | 3828.74789 | 3781.17744 | 8735.66924 | 9531.773 | 9316.31784 | -1.30004678 | 3.21E-40 | 3.67E-39 |
| gene-BPHYT_RS15005 | 1612.17156 | 1567.1303 | 1240.39701 | 352.976251 | 416.158455 | 393.838509 | 1.92786305 | 3.32E-40 | 3.79E-39 |
| gene-BPHYT_RS03780 | 10691.411 | 10854.6976 | 8995.92847 | 3440.0395 | 3703.06266 | 3713.85187 | 1.49227271 | 4.91E-40 | 5.60E-39 |
| gene-BPHYT_RS21425 | 1777.37924 | 1571.39751 | 1466.1086 | 463.404576 | 467.243774 | 523.30726 | 1.72696274 | 4.92E-40 | 5.60E-39 |
| gene-BPHYT_RS11695 | 788.527476 | 752.094529 | 532.760682 | 118.316062 | 87.2188379 | 124.941872 | 2.6417518 | 8.03E-40 | 9.12E-39 |
| gene-BPHYT_RS33905 | 2296.14732 | 2079.19466 | 1777.22457 | 5974.96112 | 6837.95689 | 6323.14517 | -1.63636558 | 8.22E-40 | 9.32E-39 |
| gene-BPHYT_RS01635 | 1905.8741 | 2658.46747 | 1989.71881 | 7444.05223 | 7960.58793 | 7453.05427 | -1.80278296 | 1.06E-39 | 1.20E-38 |
| gene-BPHYT_RS12880 | 642.47431 | 448.056315 | 461.590362 | 1949.25712 | 2064.59449 | 2157.51073 | -1.98813226 | 1.14E-39 | 1.28E-38 |
| gene-BPHYT_RS17945 | 4763.88715 | 5732.98723 | 6107.43019 | 1891.08506 | 1739.39282 | 1806.22489 | 1.61024308 | 1.21E-39 | 1.36E-38 |
| gene-BPHYT_RS21865 | 14726.6285 | 13596.3756 | 11463.5051 | 4368.82059 | 4646.27209 | 4540.46005 | 1.5535519 | 1.21E-39 | 1.36E-38 |
| gene-BPHYT_RS18170 | 11619.6068 | 14663.1763 | 15756.0922 | 43306.6366 | 46436.5553 | 43275.1565 | -1.66193392 | 1.86E-39 | 2.08E-38 |
| gene-BPHYT_RS32790 | 764.584334 | 719.023706 | 874.378219 | 186.347797 | 214.309145 | 198.277318 | 1.97950015 | 1.93E-39 | 2.16E-38 |
| gene-BPHYT_RS33780 | 2137.32448 | 2438.70652 | 2137.14304 | 774.970205 | 636.697517 | 617.466352 | 1.72544423 | 2.33E-39 | 2.61E-38 |
| gene-BPHYT_RS15610 | 2334.45635 | 2433.37251 | 2824.44499 | 8518.75646 | 7373.72975 | 7090.90392 | -1.59827445 | 3.29E-39 | 3.67E-38 |
| gene-BPHYT_RS24500 | 626.512216 | 626.21204 | 676.118041 | 157.754749 | 117.122439 | 100.496723 | 2.36129703 | 3.39E-39 | 3.77E-38 |
| gene-BPHYT_RS06405 | 3470.95749 | 4207.46216 | 3891.99979 | 10145.6023 | 10690.5376 | 10215.3561 | -1.42455281 | 3.53E-39 | 3.92E-38 |
| gene-BPHYT_RS04625 | 465.29506 | 474.726334 | 297.898626 | 51.2702935 | 28.6576182 | 39.8365389 | 3.35651346 | 4.08E-39 | 4.52E-38 |
| gene-BPHYT_RS12755 | 987.255555 | 864.108608 | 838.793059 | 2706.47992 | 2445.86541 | 2647.31908 | -1.53478699 | 4.60E-39 | 5.08E-38 |
| gene-BPHYT_RS16935 | 2081.45714 | 2127.2007 | 1909.39802 | 5156.60836 | 5456.1613 | 5033.88991 | -1.35445824 | 4.82E-39 | 5.31E-38 |
| gene-BPHYT_RS04345 | 849.183436 | 1052.93234 | 1040.10339 | 2919.44883 | 2930.55295 | 2895.39207 | -1.57310468 | 5.39E-39 | 5.94E-38 |
| gene-BPHYT_RS21520 | 746.227926 | 805.434566 | 742.204767 | 2076.44689 | 2018.49311 | 2037.09574 | -1.41922958 | 8.31E-39 | 9.14E-38 |
| gene-BPHYT_RS22190 | 3340.86641 | 3010.51172 | 3090.82533 | 7514.0559 | 8272.08378 | 7993.56367 | -1.33213526 | 9.16E-39 | 1.00E-37 |
| gene-BPHYT_RS17705 | 842.798598 | 986.790694 | 991.300888 | 2701.55008 | 2711.25988 | 2552.25461 | -1.4986724 | 2.18E-38 | 2.38E-37 |
| gene-BPHYT_RS04450 | 2152.48847 | 2262.68439 | 2227.63102 | 5125.05741 | 5503.50867 | 5506.49612 | -1.28050075 | 2.76E-38 | 3.01E-37 |
| gene-BPHYT_RS19745 | 706.322689 | 662.483266 | 465.657238 | 105.498489 | 110.892523 | 105.023602 | 2.51536226 | 2.78E-38 | 3.03E-37 |
| gene-BPHYT_RS32625 | 527.547229 | 612.343631 | 590.713657 | 1691.91969 | 1741.88479 | 1611.56907 | -1.54501477 | 3.19E-38 | 3.47E-37 |
| gene-BPHYT_RS31035 | 6.38483787 | 3.20040225 | 6.10031316 | 189.305699 | 225.522995 | 242.640737 | -5.36021503 | 3.60E-38 | 3.91E-37 |
| gene-BPHYT_RS01630 | 1696.77066 | 1976.78179 | 1384.77109 | 5678.185 | 5900.97738 | 5425.01229 | -1.74921836 | 4.94E-38 | 5.35E-37 |
| gene-BPHYT_RS26115 | 381.494063 | 331.775033 | 194.193302 | 9.85967182 | 3.7379502 | 2.71612765 | 5.78968461 | 5.35E-38 | 5.78E-37 |
| gene-BPHYT_RS05170 | 249.806782 | 299.771011 | 262.313466 | 1140.76403 | 884.648213 | 1071.05967 | -1.93475836 | 1.01E-37 | 1.09E-36 |
| gene-BPHYT_RS29165 | 1419.03022 | 1683.41158 | 1879.91317 | 463.404576 | 500.885326 | 491.619105 | 1.7746628 | 1.08E-37 | 1.16E-36 |
| gene-BPHYT_RS04815 | 2446.98911 | 2599.79343 | 2126.97585 | 732.573616 | 829.824943 | 813.932919 | 1.59452498 | 1.70E-37 | 1.83E-36 |
| gene-BPHYT_RS12595 | 9244.44713 | 9962.85221 | 10883.9754 | 24703.4078 | 26404.8802 | 24705.8971 | -1.33319228 | 2.25E-37 | 2.41E-36 |
| gene-BPHYT_RS20445 | 548.297952 | 569.671601 | 518.526618 | 98.5967182 | 109.646539 | 138.52251 | 2.23372403 | 2.49E-37 | 2.67E-36 |
| gene-BPHYT_RS16180 | 2189.99939 | 3044.64934 | 2569.24856 | 752.29296 | 783.723558 | 769.569501 | 1.75867726 | 4.25E-37 | 4.54E-36 |
| gene-BPHYT_RS18755 | 192.343241 | 249.631376 | 259.263309 | 874.552891 | 1153.78063 | 975.089826 | -2.10170875 | 4.29E-37 | 4.58E-36 |
| gene-BPHYT_RS01825 | 1796.53375 | 1640.73955 | 1492.54329 | 4561.08419 | 4240.08151 | 4444.49021 | -1.42539246 | 4.40E-37 | 4.69E-36 |
| gene-BPHYT_RS09235 | 5616.26301 | 5322.26894 | 3926.56824 | 1325.13989 | 1346.90805 | 1498.39709 | 1.83349865 | 5.07E-37 | 5.38E-36 |
| gene-BPHYT_RS08680 | 40870.9434 | 29042.5836 | 14981.3524 | 895.258202 | 1027.9363 | 895.416748 | 4.91276761 | 6.43E-37 | 6.82E-36 |
| gene-BPHYT_RS02835 | 537.124485 | 673.151273 | 575.462874 | 1786.57253 | 1751.85266 | 1904.91086 | -1.61014925 | 8.15E-37 | 8.62E-36 |
| gene-BPHYT_RS08040 | 3465.37075 | 3814.87948 | 3808.62885 | 1359.64874 | 1216.0798 | 1457.65517 | 1.45768916 | 8.47E-37 | 8.95E-36 |
| gene-BPHYT_RS05790 | 58.2616455 | 45.8724323 | 39.6520355 | 305.649827 | 320.217733 | 379.352495 | -2.79496419 | 8.96E-37 | 9.44E-36 |
| gene-BPHYT_RS11065 | 97.3687775 | 91.7448645 | 90.4879785 | 397.344774 | 458.521891 | 502.483615 | -2.27867747 | 1.25E-36 | 1.32E-35 |
| gene-BPHYT_RS18010 | 3304.9517 | 3760.47265 | 3953.00293 | 9588.53085 | 9504.36136 | 9148.8233 | -1.35824109 | 1.31E-36 | 1.37E-35 |
| gene-BPHYT_RS17470 | 248.210572 | 251.764977 | 288.748156 | 1006.67249 | 877.172312 | 859.201713 | -1.79961236 | 1.51E-36 | 1.58E-35 |
| gene-BPHYT_RS22610 | 843.596703 | 788.365754 | 727.970703 | 231.702288 | 181.913576 | 213.668708 | 1.90792338 | 1.62E-36 | 1.69E-35 |
| gene-BPHYT_RS18370 | 1222.69645 | 1233.22167 | 993.334326 | 355.934153 | 295.298065 | 306.922424 | 1.84683153 | 2.11E-36 | 2.20E-35 |
| gene-BPHYT_RS04445 | 6702.48355 | 6732.57954 | 6171.48348 | 16168.8758 | 14766.1493 | 15485.5491 | -1.24343943 | 2.35E-36 | 2.44E-35 |
| gene-BPHYT_RS16655 | 10390.5255 | 13090.712 | 14371.3211 | 39563.9051 | 38823.5967 | 37434.5766 | -1.61357359 | 2.46E-36 | 2.56E-35 |
| gene-BPHYT_RS12045 | 856.366379 | 997.458702 | 1006.55167 | 2498.44084 | 2797.23273 | 2731.51904 | -1.48970754 | 2.65E-36 | 2.75E-35 |
| gene-BPHYT_RS11495 | 1552.31371 | 1787.95806 | 1766.04066 | 4206.136 | 4623.84439 | 4469.84073 | -1.38161821 | 2.74E-36 | 2.84E-35 |
| gene-BPHYT_RS30570 | 133.28349 | 197.358139 | 261.296747 | 1179.21675 | 1153.78063 | 1004.06185 | -2.50058329 | 2.90E-36 | 3.00E-35 |
| gene-BPHYT_RS34765 | 261.778353 | 214.426951 | 219.611274 | 798.633418 | 791.199458 | 844.715699 | -1.80318073 | 3.57E-36 | 3.68E-35 |
| gene-BPHYT_RS15565 | 778.95022 | 632.612845 | 450.406455 | 108.45639 | 77.2509707 | 81.4838295 | 2.79885959 | 3.61E-36 | 3.72E-35 |
| gene-BPHYT_RS34995 | 324.828626 | 392.582676 | 369.068946 | 69.0177028 | 62.2991699 | 51.6064253 | 2.57275635 | 3.90E-36 | 4.01E-35 |
| gene-BPHYT_RS15090 | 1181.19501 | 964.387878 | 1086.87246 | 328.327072 | 313.987816 | 341.326708 | 1.71596481 | 4.92E-36 | 5.04E-35 |
| gene-BPHYT_RS29965 | 253.797305 | 283.769 | 301.965501 | 42.3965888 | 36.1335186 | 35.3096594 | 2.87983338 | 5.18E-36 | 5.30E-35 |
| gene-BPHYT_RS21290 | 2132.53585 | 1907.43974 | 1610.48267 | 555.099524 | 580.628264 | 602.074962 | 1.70151838 | 5.40E-36 | 5.52E-35 |
| gene-BPHYT_RS18765 | 484.449573 | 502.463153 | 443.289423 | 1418.80678 | 1734.40889 | 1424.15626 | -1.67718174 | 5.55E-36 | 5.66E-35 |
| gene-BPHYT_RS18295 | 995.236602 | 1913.84055 | 1765.02394 | 265.225172 | 209.325211 | 266.18051 | 2.65417998 | 6.39E-36 | 6.51E-35 |
| gene-BPHYT_RS33500 | 90.1858349 | 60.8076428 | 76.2539145 | 449.601035 | 578.136297 | 400.17614 | -2.64014827 | 6.65E-36 | 6.75E-35 |
| gene-BPHYT_RS17840 | 465.29506 | 440.58871 | 449.389736 | 1292.60298 | 1258.44323 | 1468.51968 | -1.56854758 | 6.75E-36 | 6.85E-35 |
| gene-BPHYT_RS35030 | 825.240294 | 728.624912 | 430.072078 | 88.7370464 | 72.2670371 | 45.2687942 | 3.27180119 | 7.29E-36 | 7.38E-35 |
| gene-BPHYT_RS33245 | 799.700943 | 842.772593 | 839.809778 | 2100.1101 | 2135.61555 | 2032.56886 | -1.33673932 | 1.09E-35 | 1.10E-34 |
| gene-BPHYT_RS38700 | 365.531968 | 327.50783 | 354.834882 | 54.228195 | 37.379502 | 65.1870636 | 2.72203482 | 1.11E-35 | 1.12E-34 |
| gene-BPHYT_RS08610 | 498.815458 | 500.329552 | 390.420042 | 87.7510792 | 94.6947383 | 96.8752195 | 2.31542718 | 1.21E-35 | 1.22E-34 |
| gene-BPHYT_RS03295 | 3178.85115 | 3742.33703 | 4144.14607 | 10154.476 | 10522.3298 | 10225.3152 | -1.48202386 | 1.30E-35 | 1.31E-34 |
| gene-BPHYT_RS18675 | 1276.96757 | 1195.88364 | 1306.48373 | 3173.82836 | 2989.11417 | 3203.21987 | -1.30932906 | 1.36E-35 | 1.36E-34 |
| gene-BPHYT_RS12130 | 3234.71848 | 3603.65293 | 3576.81695 | 8503.96695 | 7994.22948 | 8342.13339 | -1.25438867 | 1.36E-35 | 1.37E-34 |
| gene-BPHYT_RS32795 | 612.944435 | 674.218074 | 674.084604 | 142.965241 | 169.453742 | 186.507432 | 1.97335844 | 1.37E-35 | 1.37E-34 |
| gene-BPHYT_RS04715 | 939.369271 | 1075.33516 | 1025.86933 | 2526.04792 | 2682.60226 | 2712.50615 | -1.38232168 | 1.38E-35 | 1.37E-34 |
| gene-BPHYT_RS28255 | 291.308228 | 343.509842 | 231.8119 | 29.5790155 | 31.149585 | 36.2150353 | 3.15508142 | 1.56E-35 | 1.56E-34 |
| gene-BPHYT_RS19545 | 1313.68039 | 1339.90174 | 1309.53389 | 3228.05656 | 3637.02554 | 3194.16612 | -1.34344665 | 1.66E-35 | 1.65E-34 |
| gene-BPHYT_RS17950 | 4447.03957 | 5356.40657 | 5723.11046 | 1874.32361 | 1759.32856 | 1782.68511 | 1.51892653 | 1.84E-35 | 1.82E-34 |
| gene-BPHYT_RS34260 | 165.20768 | 122.682086 | 132.173452 | 588.622408 | 581.874247 | 570.386806 | -2.0438453 | 2.26E-35 | 2.24E-34 |
| gene-BPHYT_RS08020 | 3879.58711 | 4522.16838 | 4501.01439 | 1676.14421 | 1679.58562 | 1675.85076 | 1.35829275 | 2.56E-35 | 2.53E-34 |
| gene-BPHYT_RS09565 | 2292.9549 | 1659.94197 | 1711.13784 | 560.02936 | 470.981725 | 464.457828 | 1.92159586 | 3.14E-35 | 3.10E-34 |
| gene-BPHYT_RS28265 | 1829.25605 | 1563.9299 | 1481.35938 | 466.362477 | 550.724662 | 430.95892 | 1.7547186 | 4.44E-35 | 4.37E-34 |
| gene-BPHYT_RS23965 | 34.3185035 | 45.8724323 | 52.8693807 | 343.116579 | 279.100281 | 301.490169 | -2.80925156 | 4.67E-35 | 4.59E-34 |
| gene-BPHYT_RS24925 | 419.80309 | 417.119093 | 449.389736 | 54.228195 | 95.9407217 | 74.2408224 | 2.53150701 | 5.62E-35 | 5.52E-34 |
| gene-BPHYT_RS26540 | 27.1355609 | 26.6700188 | 45.7523487 | 259.309369 | 342.645435 | 276.139644 | -3.14651364 | 6.34E-35 | 6.21E-34 |
| gene-BPHYT_RS17585 | 479.660945 | 404.317484 | 410.754419 | 100.568653 | 74.7590039 | 76.0515742 | 2.36280563 | 1.01E-34 | 9.91E-34 |
| gene-BPHYT_RS20230 | 90.1858349 | 28.8036203 | 43.718911 | 3295.10232 | 2832.12026 | 2554.97074 | -5.7286722 | 1.02E-34 | 9.98E-34 |
| gene-BPHYT_RS15115 | 481.257154 | 494.995548 | 413.804576 | 1424.72258 | 1521.34573 | 1303.74127 | -1.61109457 | 1.04E-34 | 1.02E-33 |
| gene-BPHYT_RS18290 | 83.0028923 | 91.7448645 | 97.6050105 | 376.639464 | 464.751808 | 459.025573 | -2.25841912 | 1.12E-34 | 1.09E-33 |
| gene-BPHYT_RS02560 | 1819.67879 | 1838.09769 | 1595.23189 | 4412.20314 | 4749.68871 | 4291.48169 | -1.35632941 | 1.17E-34 | 1.13E-33 |
| gene-BPHYT_RS18015 | 20092.2867 | 23622.169 | 25986.3173 | 65697.9513 | 61712.3117 | 61376.3365 | -1.43757559 | 1.63E-34 | 1.58E-33 |
| gene-BPHYT_RS09515 | 4969.00007 | 5668.97919 | 5606.18779 | 12475.4428 | 13170.0445 | 12917.9031 | -1.24752065 | 1.78E-34 | 1.72E-33 |
| gene-BPHYT_RS27445 | 19222.3525 | 15493.1473 | 5868.50126 | 184.375863 | 204.341277 | 223.627843 | 6.05033844 | 1.84E-34 | 1.78E-33 |
| gene-BPHYT_RS32770 | 260.980248 | 168.554519 | 196.22674 | 1013.57426 | 847.268711 | 873.687727 | -2.12159515 | 1.99E-34 | 1.91E-33 |
| gene-BPHYT_RS16595 | 2688.01674 | 3391.35959 | 3555.46585 | 9299.64246 | 8937.43892 | 9351.6275 | -1.51830216 | 2.02E-34 | 1.95E-33 |
| gene-BPHYT_RS03725 | 8006.58668 | 11262.2155 | 11227.6264 | 31621.9395 | 32847.8603 | 32551.8845 | -1.66984471 | 2.77E-34 | 2.66E-33 |
| gene-BPHYT_RS05955 | 222.671221 | 295.503808 | 356.86832 | 1213.7256 | 1125.12301 | 1183.32628 | -2.01349482 | 5.44E-34 | 5.21E-33 |
| gene-BPHYT_RS31055 | 3.99052367 | 2.1336015 | 0 | 300.719991 | 331.431584 | 253.505247 | -7.06985129 | 1.24E-33 | 1.19E-32 |
| gene-BPHYT_RS21515 | 852.375855 | 793.699758 | 910.980098 | 278.042745 | 210.571194 | 227.249347 | 1.83403664 | 1.26E-33 | 1.20E-32 |
| gene-BPHYT_RS17055 | 11263.6521 | 10485.5846 | 9933.34326 | 23591.2368 | 24271.7566 | 23839.4524 | -1.1782213 | 1.35E-33 | 1.29E-32 |
| gene-BPHYT_RS14015 | 1060.68119 | 1011.32711 | 957.749165 | 2503.37068 | 2403.50198 | 2578.51052 | -1.30455711 | 1.72E-33 | 1.63E-32 |
| gene-BPHYT_RS16835 | 21907.1768 | 23641.3714 | 22291.561 | 48041.251 | 49352.1564 | 48752.6806 | -1.10722717 | 2.22E-33 | 2.10E-32 |
| gene-BPHYT_RS24135 | 297.693066 | 296.570609 | 509.376149 | 30.5649827 | 47.3473691 | 35.3096594 | 3.29717796 | 2.64E-33 | 2.50E-32 |
| gene-BPHYT_RS23590 | 4111.83559 | 4143.45411 | 3454.81068 | 1506.55785 | 1528.82163 | 1434.1154 | 1.39000303 | 2.95E-33 | 2.80E-32 |
| gene-BPHYT_RS33445 | 691.158699 | 788.365754 | 779.823365 | 1903.90263 | 1936.2582 | 2006.31296 | -1.37310875 | 3.82E-33 | 3.61E-32 |
| gene-BPHYT_RS17400 | 2433.42133 | 2436.57291 | 2226.6143 | 5294.64377 | 5797.56075 | 5561.72405 | -1.23040276 | 4.54E-33 | 4.29E-32 |
| gene-BPHYT_RS04620 | 688.764385 | 627.278841 | 365.002071 | 69.0177028 | 90.9567881 | 63.3763118 | 2.92201204 | 6.37E-33 | 6.00E-32 |
| gene-BPHYT_RS28325 | 786.931267 | 770.230142 | 1169.22669 | 3257.63557 | 3324.28371 | 3869.57652 | -1.93940011 | 6.99E-33 | 6.57E-32 |
| gene-BPHYT_RS30855 | 790.921791 | 712.622901 | 463.6238 | 125.217832 | 113.384489 | 114.982737 | 2.47621345 | 7.34E-33 | 6.89E-32 |
| gene-BPHYT_RS34835 | 1154.05944 | 1209.75205 | 1488.47641 | 420.02202 | 368.811086 | 347.664339 | 1.7606353 | 8.28E-33 | 7.75E-32 |
| gene-BPHYT_RS04335 | 152.438004 | 179.222526 | 143.357359 | 648.766406 | 560.692529 | 574.913686 | -1.91210805 | 1.02E-32 | 9.52E-32 |
| gene-BPHYT_RS04435 | 534.730171 | 528.066371 | 508.35943 | 1497.68415 | 1309.52855 | 1530.99062 | -1.46586305 | 1.03E-32 | 9.57E-32 |
| gene-BPHYT_RS26625 | 260.980248 | 230.428962 | 337.550661 | 39.4386873 | 31.149585 | 28.9720283 | 3.05659244 | 1.10E-32 | 1.03E-31 |
| gene-BPHYT_RS04215 | 704.726479 | 756.361732 | 730.004141 | 1773.75496 | 1817.88978 | 1798.98188 | -1.29940931 | 1.17E-32 | 1.09E-31 |
| gene-BPHYT_RS05515 | 294.500647 | 292.303406 | 368.052227 | 48.3123919 | 49.8393359 | 59.7548083 | 2.59018177 | 1.28E-32 | 1.19E-31 |
| gene-BPHYT_RS21430 | 871.530369 | 960.120675 | 948.598696 | 295.790155 | 327.693634 | 316.881559 | 1.56441959 | 1.65E-32 | 1.53E-31 |
| gene-BPHYT_RS09025 | 9537.35156 | 11577.9885 | 10353.2481 | 27831.8816 | 26781.1672 | 24552.8886 | -1.33107471 | 1.71E-32 | 1.58E-31 |
| gene-BPHYT_RS34690 | 345.57935 | 458.724323 | 455.490049 | 1295.56088 | 1436.61886 | 1343.57781 | -1.69673449 | 1.75E-32 | 1.61E-31 |
| gene-BPHYT_RS24130 | 434.96708 | 407.517887 | 502.259117 | 82.8212433 | 112.138506 | 95.9698436 | 2.21518104 | 1.86E-32 | 1.72E-31 |
| gene-BPHYT_RS34145 | 509.19082 | 480.060338 | 213.51096 | 16.7614421 | 36.1335186 | 27.1612765 | 3.92618359 | 2.38E-32 | 2.19E-31 |
| gene-BPHYT_RS27125 | 941.763585 | 932.383856 | 702.552732 | 146.90911 | 231.752912 | 179.264425 | 2.21397625 | 2.39E-32 | 2.19E-31 |
| gene-BPHYT_RS14895 | 1887.51769 | 2091.99627 | 2217.46383 | 4876.59368 | 4939.07819 | 5173.3178 | -1.2748718 | 2.77E-32 | 2.55E-31 |
| gene-BPHYT_RS18680 | 280.134761 | 288.036203 | 325.350035 | 858.777416 | 895.862063 | 937.969415 | -1.5929626 | 2.82E-32 | 2.58E-31 |
| gene-BPHYT_RS32455 | 968.899146 | 1112.67318 | 905.896504 | 329.313039 | 269.132414 | 305.111673 | 1.72213587 | 2.86E-32 | 2.62E-31 |
| gene-BPHYT_RS19770 | 85.3972065 | 74.6760525 | 65.070007 | 344.102547 | 398.714688 | 356.718098 | -2.28014235 | 3.00E-32 | 2.73E-31 |
| gene-BPHYT_RS00130 | 767.776753 | 903.580235 | 941.481664 | 2310.12111 | 2404.74796 | 2736.95129 | -1.51352481 | 3.39E-32 | 3.09E-31 |
| gene-BPHYT_RS14855 | 1487.66722 | 1619.40354 | 1262.76482 | 3846.25798 | 3973.44106 | 3839.69912 | -1.41578654 | 3.90E-32 | 3.55E-31 |
| gene-BPHYT_RS16660 | 20145.7597 | 26080.0779 | 27986.2033 | 71166.1253 | 74345.3374 | 69486.6936 | -1.53466145 | 4.08E-32 | 3.71E-31 |
| gene-BPHYT_RS34265 | 1170.02154 | 1206.55165 | 1015.70214 | 270.155008 | 342.645435 | 368.487984 | 1.78974765 | 4.67E-32 | 4.23E-31 |
| gene-BPHYT_RS22150 | 988.05366 | 915.315044 | 829.642589 | 289.874352 | 305.265933 | 254.410623 | 1.68941021 | 5.26E-32 | 4.76E-31 |
| gene-BPHYT_RS02320 | 1229.87939 | 1158.54562 | 889.629002 | 295.790155 | 328.939617 | 276.139644 | 1.86664801 | 5.49E-32 | 4.96E-31 |
| gene-BPHYT_RS06795 | 1468.51271 | 1522.32467 | 1784.3416 | 550.169688 | 583.12023 | 562.238423 | 1.49400378 | 5.83E-32 | 5.26E-31 |
| gene-BPHYT_RS03480 | 7072.80415 | 7018.48214 | 6277.22224 | 14941.3467 | 15275.7565 | 15721.8522 | -1.17331677 | 6.31E-32 | 5.68E-31 |
| gene-BPHYT_RS10925 | 23166.5861 | 25657.6248 | 22773.4857 | 10297.4413 | 10526.0678 | 10985.831 | 1.17040359 | 7.40E-32 | 6.65E-31 |
| gene-BPHYT_RS09985 | 1866.76697 | 2231.74717 | 1730.4555 | 4969.2746 | 5565.80784 | 5520.98213 | -1.46209203 | 7.55E-32 | 6.77E-31 |
| gene-BPHYT_RS13295 | 944.157899 | 876.910217 | 717.803515 | 2335.75626 | 2509.41056 | 2402.86759 | -1.51213029 | 9.16E-32 | 8.21E-31 |
| gene-BPHYT_RS13715 | 3333.68347 | 2985.9753 | 2586.53278 | 7480.53301 | 8374.25442 | 7895.78308 | -1.41458805 | 1.51E-31 | 1.35E-30 |
| gene-BPHYT_RS16435 | 5399.97663 | 6432.80852 | 5788.18047 | 13727.6211 | 13449.1448 | 14224.3605 | -1.23264275 | 2.39E-31 | 2.14E-30 |
| gene-BPHYT_RS01745 | 669.609871 | 694.487288 | 752.371956 | 187.333765 | 190.63546 | 235.39773 | 1.78267653 | 4.25E-31 | 3.79E-30 |
| gene-BPHYT_RS06090 | 2304.92647 | 2288.28761 | 2311.00197 | 4834.1971 | 4970.22778 | 5054.71356 | -1.10579313 | 6.18E-31 | 5.50E-30 |
| gene-BPHYT_RS09995 | 1004.81386 | 704.088495 | 405.670825 | 96.6247839 | 61.0531865 | 75.1461983 | 3.1791627 | 7.33E-31 | 6.52E-30 |
| gene-BPHYT_RS33230 | 562.663837 | 460.857924 | 441.255985 | 125.217832 | 115.876456 | 117.698865 | 2.03035458 | 8.24E-31 | 7.31E-30 |
| gene-BPHYT_RS30655 | 806.085781 | 738.226119 | 749.321799 | 199.165371 | 245.45873 | 253.505247 | 1.71739496 | 9.66E-31 | 8.56E-30 |
| gene-BPHYT_RS17855 | 49765.8206 | 51401.6606 | 43987.3247 | 106694.467 | 110461.412 | 109555.914 | -1.17042017 | 1.01E-30 | 8.90E-30 |
| gene-BPHYT_RS13620 | 3014.44158 | 3699.665 | 3755.75947 | 8880.60641 | 8860.18795 | 8610.12465 | -1.33205461 | 1.08E-30 | 9.54E-30 |
| gene-BPHYT_RS34945 | 683.975756 | 496.062349 | 543.94459 | 67.0457684 | 129.582273 | 103.212851 | 2.53364895 | 1.09E-30 | 9.58E-30 |
| gene-BPHYT_RS03720 | 989.649869 | 1175.61443 | 1281.06576 | 3053.54036 | 3055.15129 | 3068.31887 | -1.41416569 | 1.17E-30 | 1.03E-29 |
| gene-BPHYT_RS21420 | 782.142639 | 778.764548 | 663.917415 | 229.730354 | 244.212746 | 225.438595 | 1.67179733 | 1.57E-30 | 1.38E-29 |
| gene-BPHYT_RS04630 | 275.346133 | 242.16377 | 141.323921 | 9.85967182 | 14.9518008 | 13.5806382 | 4.11308377 | 1.64E-30 | 1.44E-29 |
| gene-BPHYT_RS03275 | 1076.64329 | 1072.13475 | 1066.53808 | 394.386873 | 429.864272 | 378.447119 | 1.42102779 | 2.95E-30 | 2.58E-29 |
| gene-BPHYT_RS19160 | 93.3782538 | 112.014079 | 101.671886 | 378.611398 | 426.126322 | 458.120197 | -2.04465235 | 3.15E-30 | 2.76E-29 |
| gene-BPHYT_RS37255 | 12764.8871 | 11560.9197 | 12920.4633 | 34635.0552 | 27422.8486 | 33388.4518 | -1.3576171 | 3.25E-30 | 2.83E-29 |
| gene-BPHYT_RS25985 | 376.705434 | 446.989514 | 285.698 | 2.95790155 | 2.4919668 | 0 | 7.75048667 | 3.86E-30 | 3.36E-29 |
| gene-BPHYT_RS06100 | 2482.10572 | 2575.25701 | 2237.79821 | 1040.19538 | 931.995582 | 922.578025 | 1.3332744 | 4.55E-30 | 3.96E-29 |
| gene-BPHYT_RS18415 | 1138.09735 | 1217.21966 | 1121.4409 | 453.544904 | 412.420505 | 448.161062 | 1.40180892 | 5.07E-30 | 4.41E-29 |
| gene-BPHYT_RS05965 | 4103.05643 | 4544.5712 | 4347.48984 | 9117.23853 | 9408.42064 | 9682.08969 | -1.11836067 | 5.10E-30 | 4.42E-29 |
| gene-BPHYT_RS05160 | 524.35481 | 501.396353 | 537.844277 | 1310.35039 | 1283.3629 | 1435.92615 | -1.36613402 | 5.72E-30 | 4.96E-29 |
| gene-BPHYT_RS15920 | 11123.1857 | 13260.3333 | 12860.4769 | 28012.3136 | 28918.0287 | 28496.7059 | -1.19780318 | 5.83E-30 | 5.04E-29 |
| gene-BPHYT_RS05105 | 3525.22861 | 3602.58613 | 3744.57556 | 7462.7856 | 7712.63724 | 7878.58093 | -1.08447283 | 6.41E-30 | 5.53E-29 |
| gene-BPHYT_RS29430 | 169.996308 | 112.014079 | 126.073139 | 550.169688 | 543.248762 | 598.453459 | -2.04354411 | 7.32E-30 | 6.31E-29 |
| gene-BPHYT_RS35755 | 312.857055 | 291.236605 | 394.486917 | 1023.43394 | 1042.8881 | 1049.33065 | -1.6422368 | 7.70E-30 | 6.63E-29 |
| gene-BPHYT_RS15890 | 3663.30073 | 3775.40786 | 3858.44807 | 7706.3195 | 8229.72035 | 8498.76341 | -1.1130959 | 8.57E-30 | 7.37E-29 |
| gene-BPHYT_RS26570 | 1921.8362 | 1811.42767 | 1620.64986 | 653.696242 | 698.996687 | 711.625444 | 1.37543465 | 8.81E-30 | 7.56E-29 |
| gene-BPHYT_RS33900 | 4773.46441 | 5204.92086 | 4985.98929 | 10504.4944 | 11935.275 | 10997.6009 | -1.15996469 | 9.21E-30 | 7.89E-29 |
| gene-BPHYT_RS02090 | 4948.24935 | 3603.65293 | 2943.4011 | 1087.5218 | 986.818852 | 994.10272 | 1.90572979 | 1.00E-29 | 8.59E-29 |
| gene-BPHYT_RS01875 | 3587.48078 | 3590.85133 | 3234.18269 | 1463.1753 | 1395.50141 | 1563.58415 | 1.23486637 | 1.45E-29 | 1.24E-28 |
| gene-BPHYT_RS14430 | 1767.00388 | 2035.45583 | 2015.13678 | 4393.46976 | 4441.93082 | 4567.62133 | -1.20476222 | 1.46E-29 | 1.24E-28 |
| gene-BPHYT_RS35645 | 504.402191 | 471.525932 | 402.620668 | 95.6388167 | 127.090307 | 105.928978 | 2.07620598 | 1.50E-29 | 1.27E-28 |
| gene-BPHYT_RS33660 | 1638.50902 | 1372.97257 | 1517.96126 | 3597.79425 | 3502.45933 | 3779.03894 | -1.2635609 | 1.94E-29 | 1.65E-28 |
| gene-BPHYT_RS27435 | 7435.14369 | 6454.14454 | 4776.5452 | 1532.193 | 1935.01222 | 1866.88507 | 1.80752809 | 2.10E-29 | 1.78E-28 |
| gene-BPHYT_RS09665 | 668.013662 | 655.015661 | 697.469138 | 196.207469 | 229.260945 | 229.060098 | 1.62726949 | 3.00E-29 | 2.54E-28 |
| gene-BPHYT_RS01590 | 88.5896254 | 71.4756503 | 90.4879785 | 347.060448 | 356.351252 | 361.244977 | -2.08316733 | 3.04E-29 | 2.57E-28 |
| gene-BPHYT_RS12165 | 1794.93755 | 1795.42566 | 1809.75957 | 4061.19882 | 3993.37679 | 3731.95939 | -1.12595056 | 3.08E-29 | 2.60E-28 |
| gene-BPHYT_RS18910 | 117.321396 | 121.615286 | 105.738761 | 451.57297 | 641.68145 | 451.782566 | -2.16115059 | 3.50E-29 | 2.95E-28 |
| gene-BPHYT_RS28205 | 177.977356 | 225.094958 | 263.330185 | 9.85967182 | 16.1977842 | 27.1612765 | 3.62663668 | 3.55E-29 | 2.99E-28 |
| gene-BPHYT_RS01915 | 372.71491 | 380.847868 | 525.64365 | 93.6668823 | 85.9728545 | 87.8214607 | 2.25561398 | 4.50E-29 | 3.78E-28 |
| gene-BPHYT_RS21295 | 521.960496 | 557.936792 | 444.306142 | 113.386226 | 149.518008 | 121.320368 | 1.99478714 | 4.96E-29 | 4.16E-28 |
| gene-BPHYT_RS16440 | 6091.93343 | 6746.44794 | 7140.41655 | 2534.92163 | 2840.84215 | 2198.25264 | 1.3999267 | 6.82E-29 | 5.72E-28 |
| gene-BPHYT_RS34930 | 336.002093 | 284.8358 | 302.98222 | 68.0317356 | 66.0371201 | 61.56556 | 2.24274686 | 6.87E-29 | 5.75E-28 |
| gene-BPHYT_RS26785 | 738.246878 | 549.402386 | 711.703202 | 1883.19732 | 1982.35959 | 1855.11518 | -1.51470709 | 7.25E-29 | 6.05E-28 |
| gene-BPHYT_RS25825 | 256.191619 | 241.09697 | 305.015658 | 50.2843263 | 47.3473691 | 38.931163 | 2.55930522 | 7.54E-29 | 6.29E-28 |
| gene-BPHYT_RS24555 | 1154.05944 | 1048.66514 | 1046.20371 | 431.853626 | 394.976737 | 391.122381 | 1.41580546 | 8.09E-29 | 6.74E-28 |
| gene-BPHYT_RS02820 | 2597.0328 | 2585.92502 | 2272.36665 | 5582.54619 | 5554.59399 | 5502.87462 | -1.15810935 | 9.07E-29 | 7.54E-28 |
| gene-BPHYT_RS37420 | 2522.80906 | 2069.59346 | 969.949792 | 69.0177028 | 56.0692529 | 43.4580424 | 5.04560961 | 1.11E-28 | 9.17E-28 |
| gene-BPHYT_RS06215 | 496.421144 | 634.746446 | 632.399131 | 1649.5231 | 1660.89587 | 1576.25941 | -1.47256318 | 1.12E-28 | 9.30E-28 |
| gene-BPHYT_RS34965 | 251.402991 | 291.236605 | 271.463935 | 56.2001294 | 37.379502 | 31.6881559 | 2.69815365 | 1.40E-28 | 1.16E-27 |
| gene-BPHYT_RS23700 | 9589.22837 | 8329.58026 | 6220.28598 | 2419.56347 | 2662.66652 | 2583.94277 | 1.65505955 | 1.51E-28 | 1.25E-27 |
| gene-BPHYT_RS34955 | 369.522492 | 260.299383 | 399.570512 | 61.1299653 | 62.2991699 | 43.4580424 | 2.63475378 | 1.52E-28 | 1.25E-27 |
| gene-BPHYT_RS26120 | 253.797305 | 208.026146 | 134.206889 | 0.98596718 | 3.7379502 | 7.24300706 | 5.60657792 | 1.56E-28 | 1.28E-27 |
| gene-BPHYT_RS14400 | 4711.21224 | 4688.5893 | 4834.49818 | 2285.47193 | 1962.42385 | 2039.81186 | 1.17835957 | 1.56E-28 | 1.28E-27 |
| gene-BPHYT_RS03845 | 794.912314 | 581.406409 | 562.245529 | 166.628454 | 169.453742 | 138.52251 | 2.03529349 | 1.89E-28 | 1.55E-27 |
| gene-BPHYT_RS19030 | 1240.25476 | 1465.78423 | 1055.35418 | 380.583332 | 381.27092 | 425.526665 | 1.66212062 | 2.36E-28 | 1.94E-27 |
| gene-BPHYT_RS18055 | 10186.2107 | 14711.1824 | 16591.8351 | 45487.596 | 46522.5281 | 44287.3667 | -1.71606649 | 2.69E-28 | 2.20E-27 |
| gene-BPHYT_RS09275 | 2109.39081 | 2013.05302 | 1718.25487 | 4515.7297 | 4737.22888 | 4703.42771 | -1.25623696 | 3.28E-28 | 2.68E-27 |
| gene-BPHYT_RS11435 | 118.917605 | 121.615286 | 118.956107 | 391.428971 | 458.521891 | 449.066438 | -1.85306859 | 4.31E-28 | 3.52E-27 |
| gene-BPHYT_RS05960 | 2269.01176 | 2320.29163 | 2190.01242 | 4746.44602 | 4686.14356 | 5078.25333 | -1.09810203 | 4.45E-28 | 3.62E-27 |
| gene-BPHYT_RS28235 | 324.030522 | 313.639421 | 277.564249 | 50.2843263 | 68.5290869 | 66.9978153 | 2.30489115 | 5.55E-28 | 4.51E-27 |
| gene-BPHYT_RS23940 | 415.812566 | 525.93277 | 648.666632 | 127.189767 | 108.400556 | 116.793489 | 2.17003156 | 5.97E-28 | 4.85E-27 |
| gene-BPHYT_RS18760 | 217.084487 | 204.825744 | 194.193302 | 632.004964 | 849.760678 | 660.019019 | -1.79402777 | 6.13E-28 | 4.98E-27 |
| gene-BPHYT_RS11155 | 339.194512 | 267.766988 | 327.383473 | 54.228195 | 69.7750703 | 40.7419147 | 2.52214618 | 7.56E-28 | 6.13E-27 |
| gene-BPHYT_RS17860 | 2128.54532 | 1929.84256 | 1635.90065 | 552.141622 | 701.488653 | 662.735146 | 1.5726746 | 7.61E-28 | 6.15E-27 |
| gene-BPHYT_RS19565 | 1079.0376 | 1236.42207 | 1065.52137 | 2768.59585 | 2648.96071 | 2561.30837 | -1.23933983 | 8.51E-28 | 6.87E-27 |
| gene-BPHYT_RS03330 | 2783.78931 | 3126.793 | 3312.47004 | 1287.67314 | 1340.67814 | 1293.78214 | 1.23351659 | 9.09E-28 | 7.33E-27 |
| gene-BPHYT_RS13820 | 316.847579 | 371.246661 | 349.751288 | 920.893348 | 1014.23049 | 914.429642 | -1.45855384 | 9.30E-28 | 7.48E-27 |
| gene-BPHYT_RS20645 | 470.881793 | 518.465165 | 500.225679 | 159.726684 | 120.86039 | 136.711758 | 1.83034038 | 9.31E-28 | 7.48E-27 |
| gene-BPHYT_RS15585 | 154.034214 | 231.495763 | 213.51096 | 684.261225 | 711.456521 | 780.434011 | -1.86768813 | 9.91E-28 | 7.95E-27 |
| gene-BPHYT_RS32775 | 521.960496 | 537.667578 | 562.245529 | 161.698618 | 185.651526 | 158.44078 | 1.68508187 | 1.11E-27 | 8.87E-27 |
| gene-BPHYT_RS02570 | 287.317704 | 391.515875 | 356.86832 | 1339.9294 | 1056.59392 | 1056.57366 | -1.74079392 | 1.24E-27 | 9.93E-27 |
| gene-BPHYT_RS12980 | 1038.33426 | 1273.7601 | 1413.23921 | 3476.52029 | 3194.70143 | 3377.05204 | -1.4327941 | 1.26E-27 | 1.00E-26 |
| gene-BPHYT_RS17980 | 3761.46761 | 4349.34666 | 4295.63718 | 10358.5712 | 10420.1592 | 8831.94174 | -1.25517868 | 1.81E-27 | 1.44E-26 |
| gene-BPHYT_RS12120 | 1168.42533 | 1326.03333 | 1370.53702 | 2885.92594 | 3001.57401 | 3085.52101 | -1.21604542 | 1.87E-27 | 1.49E-26 |
| gene-BPHYT_RS06790 | 1221.10024 | 1230.02127 | 1104.15668 | 473.264248 | 485.933525 | 447.255686 | 1.33923882 | 2.22E-27 | 1.77E-26 |
| gene-BPHYT_RS29425 | 455.717803 | 472.592732 | 479.891302 | 1160.48337 | 1101.44932 | 1205.96068 | -1.30150246 | 2.79E-27 | 2.22E-26 |
| gene-BPHYT_RS06705 | 408.629623 | 330.708233 | 324.333316 | 1025.40587 | 1152.53464 | 979.616705 | -1.56563325 | 2.84E-27 | 2.25E-26 |
| gene-BPHYT_RS33705 | 500.411668 | 502.463153 | 408.720981 | 108.45639 | 142.042107 | 107.73973 | 1.98699562 | 2.91E-27 | 2.31E-26 |
| gene-BPHYT_RS22110 | 533.133962 | 612.343631 | 556.145216 | 1359.64874 | 1495.18008 | 1379.79285 | -1.31606283 | 2.93E-27 | 2.32E-26 |
| gene-BPHYT_RS20220 | 231.450373 | 87.4776615 | 231.8119 | 5316.33505 | 4388.35353 | 4238.06451 | -4.66041144 | 3.30E-27 | 2.61E-26 |
| gene-BPHYT_RS28085 | 2095.02493 | 1849.8325 | 2110.70835 | 862.721285 | 846.022728 | 871.876975 | 1.23056761 | 3.39E-27 | 2.67E-26 |
| gene-BPHYT_RS02550 | 6871.68175 | 5269.99571 | 3580.88382 | 1168.37111 | 1369.33576 | 1329.0918 | 2.02407036 | 4.07E-27 | 3.20E-26 |
| gene-BPHYT_RS13645 | 6302.63308 | 6794.45398 | 7008.2431 | 14004.6779 | 13847.8595 | 13538.991 | -1.04189054 | 4.15E-27 | 3.26E-26 |
| gene-BPHYT_RS23065 | 102.955511 | 61.8744435 | 118.956107 | 491.997624 | 451.04599 | 435.4858 | -2.27517513 | 5.27E-27 | 4.14E-26 |
| gene-BPHYT_RS31060 | 4.7886284 | 0 | 0 | 213.954879 | 231.752912 | 192.845063 | -6.82907535 | 6.02E-27 | 4.72E-26 |
| gene-BPHYT_RS06550 | 2249.05914 | 3087.32137 | 3278.91832 | 9012.72601 | 8121.31979 | 7959.15939 | -1.54291035 | 7.60E-27 | 5.95E-26 |
| gene-BPHYT_RS05055 | 2450.97964 | 2647.79946 | 2732.94029 | 5528.31799 | 5382.64828 | 5632.34337 | -1.07929571 | 7.73E-27 | 6.04E-26 |
| gene-BPHYT_RS27000 | 215.488278 | 219.760955 | 241.979089 | 626.089161 | 657.879234 | 648.249132 | -1.51347608 | 1.02E-26 | 7.97E-26 |
| gene-BPHYT_RS29710 | 264.970771 | 212.293349 | 184.026114 | 30.5649827 | 26.1656514 | 34.4042836 | 2.85126144 | 1.07E-26 | 8.35E-26 |
| gene-BPHYT_RS04350 | 104.55172 | 82.1436578 | 108.788918 | 410.162348 | 382.516903 | 362.150353 | -1.96252312 | 1.22E-26 | 9.52E-26 |
| gene-BPHYT_RS16005 | 832.423237 | 748.894127 | 705.602888 | 221.842616 | 270.378397 | 266.18051 | 1.59470294 | 1.27E-26 | 9.90E-26 |
| gene-BPHYT_RS38765 | 2813.31919 | 2657.40067 | 2012.08662 | 822.29663 | 883.402229 | 901.754379 | 1.52131505 | 1.52E-26 | 1.18E-25 |
| gene-BPHYT_RS13710 | 1298.5164 | 1248.15688 | 1227.17966 | 2619.7148 | 2876.97567 | 2729.70829 | -1.12357922 | 1.52E-26 | 1.18E-25 |
| gene-BPHYT_RS29370 | 897.867825 | 661.416465 | 578.513031 | 181.417962 | 139.550141 | 185.602056 | 2.07395755 | 1.60E-26 | 1.23E-25 |
| gene-BPHYT_RS03200 | 8196.53561 | 10636.0035 | 10582.0099 | 23960.9745 | 24010.1001 | 24334.693 | -1.29776794 | 1.62E-26 | 1.26E-25 |
| gene-BPHYT_RS15225 | 6004.94001 | 6279.18922 | 5711.92655 | 11914.4274 | 12272.9365 | 12897.9848 | -1.04322319 | 1.91E-26 | 1.47E-25 |
| gene-BPHYT_RS14205 | 9520.59136 | 11396.6324 | 11017.1656 | 4940.68155 | 4602.66267 | 4735.11587 | 1.16103403 | 1.92E-26 | 1.48E-25 |
| gene-BPHYT_RS18115 | 22995.7917 | 29730.6701 | 32463.8332 | 75025.2008 | 77008.0039 | 72937.0811 | -1.40102835 | 1.95E-26 | 1.50E-25 |
| gene-BPHYT_RS22475 | 2497.26971 | 1840.23129 | 1747.73972 | 629.047062 | 677.814969 | 688.991047 | 1.60921043 | 2.16E-26 | 1.66E-25 |
| gene-BPHYT_RS03690 | 3770.24676 | 4052.77605 | 4037.39059 | 7768.43543 | 8536.23226 | 8463.45375 | -1.06246581 | 2.78E-26 | 2.13E-25 |
| gene-BPHYT_RS23945 | 174.784937 | 176.022124 | 203.343772 | 23.6632124 | 27.4116348 | 26.2559006 | 2.84406511 | 4.32E-26 | 3.31E-25 |
| gene-BPHYT_RS16500 | 60.6559597 | 55.473639 | 70.1536013 | 264.239205 | 289.068148 | 278.855772 | -2.15871234 | 4.98E-26 | 3.81E-25 |
| gene-BPHYT_RS17395 | 1020.77595 | 1221.48686 | 1109.24028 | 2472.80569 | 2681.35627 | 2792.17922 | -1.24649472 | 5.14E-26 | 3.93E-25 |
| gene-BPHYT_RS33785 | 1166.82912 | 1174.54763 | 936.398069 | 407.204446 | 356.351252 | 391.122381 | 1.50397229 | 6.01E-26 | 4.58E-25 |
| gene-BPHYT_RS06680 | 5272.27987 | 5492.95706 | 5054.10945 | 10531.1155 | 11387.0423 | 10503.2656 | -1.03518098 | 6.88E-26 | 5.25E-25 |
| gene-BPHYT_RS02555 | 1934.60587 | 2035.45583 | 1429.50672 | 4959.41493 | 5332.80895 | 4755.03414 | -1.47826019 | 7.06E-26 | 5.37E-25 |
| gene-BPHYT_RS34880 | 1544.33266 | 3874.62033 | 2095.45757 | 126.203799 | 139.550141 | 128.563375 | 4.25257456 | 7.22E-26 | 5.48E-25 |
| gene-BPHYT_RS12475 | 2533.98253 | 2776.88235 | 2768.52545 | 5396.19839 | 5724.04773 | 5762.7175 | -1.06356867 | 7.35E-26 | 5.58E-25 |
| gene-BPHYT_RS02590 | 2491.68298 | 2739.54433 | 2488.92777 | 5181.25754 | 5520.95244 | 5517.36063 | -1.07123892 | 8.47E-26 | 6.42E-25 |
| gene-BPHYT_RS30980 | 7.98104733 | 16.0020113 | 7.11703202 | 175.502158 | 115.876456 | 171.116042 | -3.92412843 | 9.20E-26 | 6.96E-25 |
| gene-BPHYT_RS15845 | 1054.29635 | 1200.15084 | 1166.17653 | 2429.42314 | 2753.62331 | 2675.38573 | -1.2005929 | 9.62E-26 | 7.27E-25 |
| gene-BPHYT_RS08960 | 6210.05293 | 7034.48415 | 7177.01843 | 14178.2081 | 14632.829 | 14098.5133 | -1.0713689 | 1.14E-25 | 8.62E-25 |
| gene-BPHYT_RS33910 | 409.427728 | 409.651488 | 344.667693 | 1046.11118 | 1131.35293 | 966.036067 | -1.43178871 | 1.30E-25 | 9.78E-25 |
| gene-BPHYT_RS27165 | 484.449573 | 509.930759 | 471.757551 | 165.642487 | 156.993908 | 139.427886 | 1.66755018 | 1.43E-25 | 1.08E-24 |
| gene-BPHYT_RS15930 | 428.582242 | 443.789112 | 323.316597 | 1079.63407 | 1234.76955 | 1214.10906 | -1.5601455 | 1.51E-25 | 1.13E-24 |
| gene-BPHYT_RS14850 | 301.683589 | 329.641432 | 332.467067 | 829.1984 | 936.979516 | 811.216791 | -1.4194372 | 1.62E-25 | 1.21E-24 |
| gene-BPHYT_RS17590 | 213.892069 | 157.886511 | 175.892363 | 24.6491796 | 24.919668 | 19.9182694 | 2.98960727 | 1.65E-25 | 1.23E-24 |
| gene-BPHYT_RS25820 | 383.888377 | 325.374229 | 521.576775 | 89.7230136 | 89.7108047 | 70.6193189 | 2.30379877 | 1.70E-25 | 1.27E-24 |
| gene-BPHYT_RS23715 | 979.274508 | 887.578224 | 796.090867 | 225.786485 | 311.49585 | 298.774041 | 1.67413141 | 1.85E-25 | 1.38E-24 |
| gene-BPHYT_RS26615 | 1639.30712 | 1336.70134 | 1004.51823 | 353.962218 | 408.682555 | 363.961105 | 1.82325594 | 1.93E-25 | 1.44E-24 |
| gene-BPHYT_RS34735 | 2627.36078 | 3757.27224 | 5395.72699 | 873.566924 | 1011.73852 | 725.206082 | 2.17466525 | 2.10E-25 | 1.57E-24 |
| gene-BPHYT_RS09445 | 15845.5714 | 18053.4691 | 19471.1829 | 39575.7367 | 38237.9845 | 38748.277 | -1.12707511 | 2.25E-25 | 1.67E-24 |
| gene-BPHYT_RS17710 | 2352.01465 | 2567.78941 | 2560.09809 | 5036.32037 | 5077.38235 | 5205.00595 | -1.03461699 | 2.31E-25 | 1.72E-24 |
| gene-BPHYT_RS03775 | 7534.10868 | 7828.18391 | 7513.55237 | 14732.3216 | 15230.9011 | 14497.784 | -0.95870846 | 2.34E-25 | 1.74E-24 |
| gene-BPHYT_RS28215 | 366.330073 | 360.578654 | 226.728306 | 25.6351467 | 59.8072031 | 38.931163 | 2.95913352 | 2.38E-25 | 1.76E-24 |
| gene-BPHYT_RS15690 | 1272.17895 | 1235.35527 | 1108.22356 | 2738.03087 | 2748.63938 | 2562.21375 | -1.15379984 | 2.51E-25 | 1.86E-24 |
| gene-BPHYT_RS08915 | 4917.92137 | 4999.02832 | 4744.0102 | 9625.01163 | 9708.70264 | 9352.53287 | -0.96832938 | 2.82E-25 | 2.09E-24 |
| gene-BPHYT_RS26930 | 782.940743 | 943.051863 | 996.384482 | 306.635794 | 309.003883 | 231.776226 | 1.68602046 | 2.95E-25 | 2.17E-24 |
| gene-BPHYT_RS20450 | 691.158699 | 682.75248 | 498.192241 | 84.7931777 | 160.731858 | 141.238638 | 2.28094858 | 2.95E-25 | 2.17E-24 |
| gene-BPHYT_RS18120 | 9621.95066 | 11759.3447 | 11329.2983 | 25473.4481 | 23479.3112 | 23939.9491 | -1.15617942 | 2.95E-25 | 2.17E-24 |
| gene-BPHYT_RS34150 | 627.31032 | 705.155296 | 780.840084 | 245.505828 | 244.212746 | 237.208481 | 1.53889816 | 3.37E-25 | 2.48E-24 |
| gene-BPHYT_RS04800 | 1181.19501 | 1126.54159 | 1002.4848 | 2701.55008 | 2399.76403 | 2559.49762 | -1.21024951 | 3.42E-25 | 2.51E-24 |
| gene-BPHYT_RS22770 | 218.680697 | 206.959346 | 190.126427 | 582.706605 | 575.64433 | 597.548083 | -1.51010488 | 3.72E-25 | 2.73E-24 |
| gene-BPHYT_RS19550 | 7064.8231 | 7546.54851 | 5960.00595 | 2686.76057 | 3007.80392 | 2956.95763 | 1.24976589 | 3.90E-25 | 2.86E-24 |
| gene-BPHYT_RS17930 | 757.401392 | 977.189487 | 990.284169 | 2406.74589 | 2326.25101 | 2255.29133 | -1.36039201 | 4.03E-25 | 2.94E-24 |
| gene-BPHYT_RS24000 | 1671.23131 | 1773.02285 | 1881.94661 | 628.061095 | 789.953475 | 714.341572 | 1.32190802 | 4.06E-25 | 2.97E-24 |
| gene-BPHYT_RS29930 | 346.377454 | 308.305417 | 228.761743 | 883.426595 | 1141.32079 | 998.629599 | -1.77142468 | 4.96E-25 | 3.61E-24 |
| gene-BPHYT_RS09685 | 1620.15261 | 1455.11622 | 1357.31968 | 539.324049 | 422.388372 | 583.062069 | 1.51797681 | 5.21E-25 | 3.79E-24 |
| gene-BPHYT_RS35150 | 1102.18264 | 1009.19351 | 864.21103 | 348.046415 | 383.762887 | 330.462197 | 1.48910364 | 7.71E-25 | 5.61E-24 |
| gene-BPHYT_RS38075 | 136.475909 | 213.36015 | 126.073139 | 633.976898 | 711.456521 | 584.87282 | -2.0238985 | 9.22E-25 | 6.69E-24 |
| gene-BPHYT_RS29640 | 687.96628 | 753.16133 | 653.750227 | 251.421632 | 251.688647 | 200.08807 | 1.57797317 | 9.38E-25 | 6.80E-24 |
| gene-BPHYT_RS35080 | 2912.28417 | 3311.34953 | 3116.2433 | 1207.8098 | 1436.61886 | 1397.90036 | 1.2084304 | 9.57E-25 | 6.93E-24 |
| gene-BPHYT_RS26770 | 313.65516 | 269.90059 | 292.815032 | 854.833547 | 767.525773 | 749.651231 | -1.43444667 | 9.97E-25 | 7.21E-24 |
| gene-BPHYT_RS14425 | 2005.6372 | 2716.07471 | 2764.45858 | 6330.89528 | 6502.78736 | 6582.08267 | -1.37563017 | 1.05E-24 | 7.58E-24 |
| gene-BPHYT_RS22245 | 354.358502 | 324.307428 | 474.807707 | 1111.18501 | 1147.55071 | 1227.6897 | -1.59662211 | 1.33E-24 | 9.62E-24 |
| gene-BPHYT_RS14210 | 3666.49314 | 4156.25572 | 3787.27775 | 1732.34434 | 1882.68092 | 1820.7109 | 1.09489479 | 1.50E-24 | 1.08E-23 |
| gene-BPHYT_RS18305 | 9785.56213 | 12190.3322 | 13308.8499 | 4942.65349 | 4663.71586 | 4716.10297 | 1.30058486 | 1.61E-24 | 1.16E-23 |
| gene-BPHYT_RS24545 | 3331.28916 | 3313.48313 | 2233.73133 | 887.370464 | 1040.39614 | 959.698436 | 1.62127972 | 1.66E-24 | 1.19E-23 |
| gene-BPHYT_RS07620 | 4027.23648 | 3386.02558 | 2755.30811 | 1203.86593 | 1108.92523 | 1288.34988 | 1.49732551 | 1.68E-24 | 1.21E-23 |
| gene-BPHYT_RS18060 | 5182.09403 | 6974.74331 | 8561.78951 | 20689.5354 | 21747.3942 | 20371.8627 | -1.60023874 | 1.74E-24 | 1.25E-23 |
| gene-BPHYT_RS22560 | 6536.47777 | 5815.13089 | 6302.64021 | 12797.854 | 12337.7276 | 12278.7077 | -1.00393415 | 1.82E-24 | 1.31E-23 |
| gene-BPHYT_RS26590 | 407.033414 | 377.647466 | 410.754419 | 129.161701 | 112.138506 | 99.5913471 | 1.81180519 | 1.83E-24 | 1.31E-23 |
| gene-BPHYT_RS37205 | 1671.23131 | 1226.82086 | 857.093998 | 330.299006 | 249.19668 | 260.748254 | 2.1597702 | 1.85E-24 | 1.32E-23 |
| gene-BPHYT_RS16115 | 1975.30922 | 1941.57737 | 1767.05738 | 840.044039 | 887.14018 | 812.122167 | 1.16360078 | 1.86E-24 | 1.33E-23 |
| gene-BPHYT_RS17180 | 887.492463 | 932.383856 | 960.799322 | 2031.0924 | 1922.55238 | 2132.1602 | -1.13105008 | 2.05E-24 | 1.46E-23 |
| gene-BPHYT_RS12290 | 500.411668 | 557.936792 | 585.630063 | 174.516191 | 198.11136 | 163.873035 | 1.61898528 | 2.41E-24 | 1.72E-23 |
| gene-BPHYT_RS03730 | 642.47431 | 812.902172 | 949.615415 | 2466.88989 | 2084.53023 | 2464.43315 | -1.54656538 | 2.57E-24 | 1.82E-23 |
| gene-BPHYT_RS20990 | 229.056058 | 281.635398 | 221.644711 | 658.626078 | 733.884222 | 725.206082 | -1.53395425 | 2.63E-24 | 1.87E-23 |
| gene-BPHYT_RS06805 | 4363.23858 | 4915.81786 | 5354.04151 | 2137.57685 | 2272.67372 | 2027.1366 | 1.1848934 | 3.22E-24 | 2.28E-23 |
| gene-BPHYT_RS38835 | 282.529076 | 251.764977 | 203.343772 | 43.382556 | 53.5772861 | 38.931163 | 2.45415537 | 3.82E-24 | 2.70E-23 |
| gene-BPHYT_RS25765 | 350.367978 | 298.70421 | 157.591423 | 27.6070811 | 37.379502 | 22.6343971 | 3.22108866 | 4.77E-24 | 3.37E-23 |
| gene-BPHYT_RS09540 | 1707.94413 | 1780.49045 | 1783.32488 | 729.615715 | 841.038794 | 691.707175 | 1.2223862 | 5.09E-24 | 3.59E-23 |
| gene-BPHYT_RS00015 | 652.849672 | 891.845427 | 836.759621 | 2091.23639 | 2062.10252 | 2003.59683 | -1.37232512 | 5.62E-24 | 3.96E-23 |
| gene-BPHYT_RS19840 | 3994.51419 | 5357.47337 | 5257.45322 | 12208.2457 | 11872.9758 | 11597.8651 | -1.28854183 | 7.82E-24 | 5.50E-23 |
| gene-BPHYT_RS20510 | 245.816258 | 197.358139 | 199.276896 | 26.6211139 | 43.6094189 | 37.1204112 | 2.59475789 | 8.97E-24 | 6.30E-23 |
| gene-BPHYT_RS31115 | 816.461142 | 674.218074 | 619.181785 | 137.049438 | 228.014962 | 191.939687 | 1.92740751 | 1.10E-23 | 7.69E-23 |
| gene-BPHYT_RS15080 | 1195.56089 | 1042.26433 | 887.595564 | 400.302676 | 327.693634 | 325.935318 | 1.56835564 | 1.14E-23 | 8.00E-23 |
| gene-BPHYT_RS17720 | 2960.17046 | 3439.36562 | 3381.60693 | 6630.6293 | 6771.91977 | 6943.32765 | -1.05710067 | 1.22E-23 | 8.57E-23 |
| gene-BPHYT_RS17565 | 91.7820443 | 109.880477 | 87.4378219 | 342.130612 | 327.693634 | 392.933133 | -1.88291001 | 1.34E-23 | 9.38E-23 |
| gene-BPHYT_RS26010 | 296.096856 | 348.843845 | 216.561117 | 25.6351467 | 42.3634355 | 56.1333047 | 2.79054931 | 1.72E-23 | 1.20E-22 |
| gene-BPHYT_RS03310 | 182.765984 | 204.825744 | 154.541267 | 631.018997 | 523.313027 | 548.657785 | -1.65263607 | 2.10E-23 | 1.47E-22 |
| gene-BPHYT_RS21340 | 1133.30872 | 1266.29249 | 906.913223 | 365.793825 | 377.53297 | 408.324523 | 1.52090342 | 2.11E-23 | 1.47E-22 |
| gene-BPHYT_RS24295 | 1527.57246 | 1593.80032 | 1472.20891 | 729.615715 | 675.323002 | 681.74804 | 1.13791116 | 2.16E-23 | 1.50E-22 |
| gene-BPHYT_RS22155 | 88.5896254 | 93.878466 | 59.9864127 | 329.313039 | 348.875352 | 316.881559 | -2.03369155 | 2.75E-23 | 1.91E-22 |
| gene-BPHYT_RS24160 | 514.777553 | 618.744435 | 814.391806 | 179.446027 | 186.89751 | 149.387021 | 1.91910765 | 3.06E-23 | 2.12E-22 |
| gene-BPHYT_RS29255 | 1015.18922 | 897.179431 | 628.332255 | 2529.99179 | 2915.60115 | 2453.56864 | -1.63499357 | 3.07E-23 | 2.12E-22 |
| gene-BPHYT_RS01465 | 597.780445 | 575.005604 | 593.763814 | 1448.38579 | 1318.25044 | 1261.18861 | -1.18871454 | 3.90E-23 | 2.70E-22 |
| gene-BPHYT_RS24915 | 735.054459 | 759.562134 | 724.920547 | 293.81822 | 310.249866 | 292.43641 | 1.30906273 | 3.99E-23 | 2.76E-22 |
| gene-BPHYT_RS05465 | 95.772568 | 52.2732368 | 69.1368824 | 371.709628 | 397.468704 | 296.96329 | -2.28061222 | 4.40E-23 | 3.03E-22 |
| gene-BPHYT_RS01740 | 996.034707 | 1030.52953 | 989.26745 | 394.386873 | 428.618289 | 451.782566 | 1.24186379 | 5.33E-23 | 3.67E-22 |
| gene-BPHYT_RS30970 | 667.215557 | 853.4406 | 995.367763 | 2321.95271 | 2312.54519 | 2217.26554 | -1.44691726 | 5.70E-23 | 3.92E-22 |
| gene-BPHYT_RS05755 | 9.5772568 | 20.2692143 | 19.3176583 | 166.628454 | 125.844323 | 168.399914 | -3.26272947 | 6.05E-23 | 4.16E-22 |
| gene-BPHYT_RS03840 | 581.020246 | 526.999571 | 564.278967 | 147.895077 | 199.357344 | 118.604241 | 1.85452797 | 6.07E-23 | 4.17E-22 |
| gene-BPHYT_RS33790 | 612.944435 | 625.14524 | 524.626931 | 175.502158 | 170.699726 | 220.911715 | 1.63187993 | 6.65E-23 | 4.56E-22 |
| gene-BPHYT_RS02355 | 6127.05004 | 6789.11997 | 7562.35488 | 14907.8238 | 15863.8606 | 13960.8961 | -1.12730395 | 9.02E-23 | 6.17E-22 |
| gene-BPHYT_RS29590 | 8364.1376 | 7276.64792 | 6022.0258 | 2829.72581 | 3112.46653 | 2862.79854 | 1.29923539 | 1.09E-22 | 7.44E-22 |
| gene-BPHYT_RS29770 | 88.5896254 | 87.4776615 | 111.839075 | 320.439334 | 343.891418 | 363.961105 | -1.83916031 | 1.18E-22 | 8.07E-22 |
| gene-BPHYT_RS11360 | 156.428528 | 116.281282 | 165.725174 | 454.530871 | 488.425492 | 484.376097 | -1.69912031 | 1.72E-22 | 1.17E-21 |
| gene-BPHYT_RS02690 | 2735.10492 | 3153.46302 | 3414.14193 | 1449.37176 | 1299.56068 | 1269.33699 | 1.21058772 | 1.77E-22 | 1.21E-21 |
| gene-BPHYT_RS22085 | 830.028923 | 880.110619 | 546.994746 | 148.881045 | 228.014962 | 181.075177 | 2.02122039 | 1.77E-22 | 1.21E-21 |
| gene-BPHYT_RS16255 | 3678.46472 | 3245.20788 | 3171.14612 | 6864.30352 | 6720.83445 | 6857.31694 | -1.01759037 | 2.12E-22 | 1.44E-21 |
| gene-BPHYT_RS12750 | 419.004985 | 361.645454 | 292.815032 | 1062.87262 | 944.455416 | 1008.58873 | -1.48795394 | 2.34E-22 | 1.59E-21 |
| gene-BPHYT_RS04290 | 842.000494 | 1033.72993 | 1142.792 | 2533.93566 | 2648.96071 | 2325.00527 | -1.31557824 | 3.00E-22 | 2.03E-21 |
| gene-BPHYT_RS13175 | 719.092365 | 602.742424 | 348.734569 | 123.245898 | 92.2027715 | 110.455858 | 2.35539288 | 3.01E-22 | 2.03E-21 |
| gene-BPHYT_RS07625 | 375.907329 | 370.17986 | 398.553793 | 124.231865 | 110.892523 | 127.658 | 1.65342111 | 3.47E-22 | 2.35E-21 |
| gene-BPHYT_RS14395 | 5545.23169 | 6480.81456 | 6553.76977 | 3090.02115 | 2665.15849 | 2717.9384 | 1.13237409 | 3.92E-22 | 2.65E-21 |
| gene-BPHYT_RS08950 | 5803.81762 | 6394.4037 | 5573.65279 | 11513.1388 | 12218.1132 | 11518.192 | -0.98802974 | 4.06E-22 | 2.74E-21 |
| gene-BPHYT_RS25810 | 449.332965 | 559.003593 | 535.810839 | 181.417962 | 163.223825 | 172.021418 | 1.57605473 | 5.00E-22 | 3.37E-21 |
| gene-BPHYT_RS28210 | 165.20768 | 162.153714 | 136.240327 | 25.6351467 | 18.689751 | 19.9182694 | 2.84578404 | 5.12E-22 | 3.44E-21 |
| gene-BPHYT_RS15765 | 2603.41764 | 2513.38257 | 1966.33427 | 5244.35944 | 5892.25549 | 5655.88314 | -1.24494 | 5.16E-22 | 3.47E-21 |
| gene-BPHYT_RS18980 | 110.138453 | 128.01609 | 142.34064 | 387.485103 | 421.142389 | 400.17614 | -1.6716415 | 5.25E-22 | 3.52E-21 |
| gene-BPHYT_RS21335 | 352.762292 | 468.325529 | 421.938327 | 121.273963 | 88.4648213 | 124.036496 | 1.88665962 | 6.38E-22 | 4.27E-21 |
| gene-BPHYT_RS26055 | 316.049474 | 297.637409 | 149.457672 | 14.7895077 | 38.6254854 | 26.2559006 | 3.28161158 | 6.55E-22 | 4.38E-21 |
| gene-BPHYT_RS34340 | 366.330073 | 423.519898 | 494.125366 | 1100.33938 | 1492.68811 | 1171.55639 | -1.5525321 | 6.64E-22 | 4.44E-21 |
| gene-BPHYT_RS14820 | 265.768876 | 282.702199 | 397.537074 | 1053.01295 | 864.712479 | 1028.507 | -1.64186564 | 6.96E-22 | 4.65E-21 |
| gene-BPHYT_RS08955 | 5039.23329 | 5726.58643 | 6304.67365 | 11836.536 | 11977.6384 | 12167.3465 | -1.07599485 | 6.98E-22 | 4.65E-21 |
| gene-BPHYT_RS09835 | 572.241094 | 703.021694 | 396.520355 | 0 | 0 | 0 | 11.5135512 | 7.13E-22 | 4.75E-21 |
| gene-BPHYT_RS03100 | 407.033414 | 376.580665 | 383.30301 | 118.316062 | 137.058174 | 124.036496 | 1.62532123 | 7.96E-22 | 5.30E-21 |
| gene-BPHYT_RS14405 | 6670.55936 | 7281.98192 | 6659.50853 | 3567.22927 | 3562.26654 | 3226.75965 | 0.99314179 | 8.10E-22 | 5.38E-21 |
| gene-BPHYT_RS05175 | 339.992616 | 722.224108 | 414.821295 | 75.919473 | 94.6947383 | 94.1590918 | 2.47912358 | 8.40E-22 | 5.57E-21 |
| gene-BPHYT_RS06970 | 750.218449 | 519.531965 | 789.990554 | 1811.22171 | 2034.69089 | 1995.44845 | -1.50245015 | 8.66E-22 | 5.74E-21 |
| gene-BPHYT_RS02960 | 624.916006 | 331.775033 | 571.395999 | 1869.39378 | 1672.10972 | 1883.18184 | -1.82568777 | 9.09E-22 | 6.02E-21 |
| gene-BPHYT_RS12925 | 268.961295 | 320.040225 | 353.818163 | 781.871976 | 874.680346 | 841.999571 | -1.40864486 | 9.56E-22 | 6.32E-21 |
| gene-BPHYT_RS01720 | 119.71571 | 178.155725 | 188.092989 | 587.636441 | 499.639343 | 616.560976 | -1.81795028 | 9.85E-22 | 6.50E-21 |
| gene-BPHYT_RS09490 | 952.138947 | 970.788683 | 880.478532 | 374.667529 | 396.222721 | 420.09441 | 1.23462154 | 9.99E-22 | 6.59E-21 |
| gene-BPHYT_RS29435 | 262.576457 | 162.153714 | 144.374078 | 718.770076 | 718.932421 | 719.773827 | -1.914979 | 1.02E-21 | 6.71E-21 |
| gene-BPHYT_RS13450 | 321.636208 | 384.04827 | 238.928932 | 0 | 1.2459834 | 1.81075177 | 8.26070959 | 1.11E-21 | 7.33E-21 |
| gene-BPHYT_RS10935 | 627.31032 | 462.991526 | 461.590362 | 148.881045 | 133.320224 | 169.30529 | 1.77882409 | 1.16E-21 | 7.65E-21 |
| gene-BPHYT_RS16095 | 1021.57406 | 938.78466 | 936.398069 | 410.162348 | 441.078123 | 401.081516 | 1.2121301 | 1.17E-21 | 7.68E-21 |
| gene-BPHYT_RS06430 | 4552.3894 | 4302.40743 | 4200.06561 | 2213.49632 | 2316.28314 | 2200.96877 | 0.95620456 | 1.17E-21 | 7.68E-21 |
| gene-BPHYT_RS05045 | 667.215557 | 765.962939 | 602.914284 | 1486.83851 | 1716.96512 | 1735.60557 | -1.27891658 | 1.23E-21 | 8.05E-21 |
| gene-BPHYT_RS09325 | 605.761493 | 617.677634 | 503.275835 | 182.403929 | 199.357344 | 216.384836 | 1.52899857 | 1.38E-21 | 9.01E-21 |
| gene-BPHYT_RS05785 | 20.7507231 | 28.8036203 | 20.3343772 | 154.796848 | 153.255958 | 146.670893 | -2.71138629 | 1.51E-21 | 9.84E-21 |
| gene-BPHYT_RS08010 | 8228.4598 | 7007.81413 | 4938.2035 | 2388.99848 | 2169.2571 | 2339.49128 | 1.5483362 | 1.52E-21 | 9.89E-21 |
| gene-BPHYT_RS28260 | 592.193712 | 461.924725 | 342.634256 | 113.386226 | 103.416622 | 120.414992 | 2.04939871 | 1.59E-21 | 1.04E-20 |
| gene-BPHYT_RS25175 | 641.676206 | 733.958916 | 625.282099 | 266.211139 | 185.651526 | 210.047205 | 1.59184726 | 1.71E-21 | 1.11E-20 |
| gene-BPHYT_RS14745 | 1070.25845 | 1318.56573 | 1134.65825 | 510.731 | 447.30804 | 485.281473 | 1.28543103 | 2.01E-21 | 1.31E-20 |
| gene-BPHYT_RS35420 | 1069.46034 | 1059.33315 | 999.434639 | 418.050085 | 274.116348 | 391.122381 | 1.52542566 | 2.03E-21 | 1.32E-20 |
| gene-BPHYT_RS03595 | 4296.99588 | 5091.83998 | 5281.85447 | 10369.4169 | 10354.122 | 9927.44656 | -1.06327698 | 2.09E-21 | 1.36E-20 |
| gene-BPHYT_RS18340 | 3246.69006 | 3031.84773 | 2403.52338 | 6515.27114 | 6634.8616 | 7128.9297 | -1.22360115 | 2.17E-21 | 1.41E-20 |
| gene-BPHYT_RS08705 | 105.349825 | 103.479673 | 131.156733 | 2.95790155 | 4.98393359 | 3.62150353 | 4.90586317 | 2.39E-21 | 1.55E-20 |
| gene-BPHYT_RS19310 | 966.504832 | 1079.60236 | 896.746034 | 2060.67141 | 2096.99006 | 2154.7946 | -1.10145706 | 2.78E-21 | 1.80E-20 |
| gene-BPHYT_RS26475 | 169.996308 | 135.483695 | 136.240327 | 23.6632124 | 16.1977842 | 18.1075177 | 2.92368518 | 2.80E-21 | 1.81E-20 |
| gene-BPHYT_RS26915 | 4972.19249 | 5177.18404 | 5247.28603 | 2476.74956 | 2648.96071 | 2775.88246 | 0.96223894 | 2.83E-21 | 1.83E-20 |
| gene-BPHYT_RS35460 | 896.271615 | 847.039796 | 915.046973 | 353.962218 | 394.976737 | 391.122381 | 1.22238957 | 2.97E-21 | 1.91E-20 |
| gene-BPHYT_RS00115 | 31.9241893 | 29.870421 | 28.4681281 | 162.684585 | 154.501941 | 186.507432 | -2.47894891 | 3.51E-21 | 2.25E-20 |
| gene-BPHYT_RS09480 | 1914.65326 | 2291.48801 | 2524.51293 | 5480.0056 | 5098.56407 | 4845.57173 | -1.1969615 | 3.51E-21 | 2.25E-20 |
| gene-BPHYT_RS09320 | 363.137654 | 308.305417 | 388.386604 | 98.5967182 | 115.876456 | 88.7268365 | 1.81356923 | 4.80E-21 | 3.08E-20 |
| gene-BPHYT_RS03585 | 3476.54422 | 4465.62794 | 3362.28927 | 8508.89678 | 9134.30429 | 8449.87312 | -1.2069832 | 5.02E-21 | 3.21E-20 |
| gene-BPHYT_RS09845 | 541.913114 | 594.208018 | 310.099252 | 0 | 0 | 0 | 11.3049957 | 5.23E-21 | 3.35E-20 |
| gene-BPHYT_RS14490 | 3122.18572 | 3404.16119 | 3230.11582 | 1647.55116 | 1339.43215 | 1553.62502 | 1.10230462 | 5.36E-21 | 3.43E-20 |
| gene-BPHYT_RS35665 | 81.4066828 | 84.2772593 | 68.1201636 | 263.253238 | 309.003883 | 282.477276 | -1.86742899 | 5.43E-21 | 3.47E-20 |
| gene-BPHYT_RS26715 | 451.727279 | 545.135183 | 462.607081 | 1133.86226 | 1111.41719 | 1113.61234 | -1.20433915 | 5.82E-21 | 3.71E-20 |
| gene-BPHYT_RS11315 | 104.55172 | 75.7428533 | 132.173452 | 920.893348 | 467.243774 | 440.012679 | -2.5480623 | 6.61E-21 | 4.21E-20 |
| gene-BPHYT_RS18320 | 2059.11021 | 2584.85822 | 2762.42514 | 5393.24049 | 5746.47543 | 5807.98629 | -1.19486756 | 8.34E-21 | 5.30E-20 |
| gene-BPHYT_RS37150 | 688.764385 | 411.78509 | 679.168198 | 2761.69408 | 1859.00723 | 1893.14097 | -1.87067857 | 8.50E-21 | 5.40E-20 |
| gene-BPHYT_RS15060 | 603.367178 | 784.098551 | 502.259117 | 165.642487 | 199.357344 | 194.655815 | 1.75615384 | 1.02E-20 | 6.47E-20 |
| gene-BPHYT_RS20205 | 805.287676 | 776.630946 | 689.335387 | 1586.4212 | 1726.93299 | 1603.42069 | -1.11299063 | 1.02E-20 | 6.47E-20 |
| gene-BPHYT_RS16840 | 14106.5012 | 16099.0901 | 14863.413 | 27802.3026 | 28666.34 | 28621.6478 | -0.91693175 | 1.05E-20 | 6.66E-20 |
| gene-BPHYT_RS07785 | 367.926282 | 435.254706 | 539.877714 | 1165.41321 | 1216.0798 | 1186.94778 | -1.41201484 | 1.07E-20 | 6.74E-20 |
| gene-BPHYT_RS26110 | 153.236109 | 167.487718 | 92.5214162 | 4.92983591 | 1.2459834 | 0.90537588 | 5.84734032 | 1.08E-20 | 6.85E-20 |
| gene-BPHYT_RS15265 | 1367.15341 | 1490.32065 | 1607.43252 | 690.177028 | 667.847102 | 575.819062 | 1.20772761 | 1.19E-20 | 7.50E-20 |
| gene-BPHYT_RS28115 | 877.915207 | 839.57219 | 491.075209 | 208.039076 | 188.143493 | 173.83217 | 1.95515445 | 1.30E-20 | 8.18E-20 |
| gene-BPHYT_RS01710 | 194.737555 | 158.953312 | 216.561117 | 568.903064 | 545.740729 | 514.253502 | -1.51230214 | 1.43E-20 | 8.99E-20 |
| gene-BPHYT_RS02790 | 477.26663 | 336.042236 | 363.985352 | 979.065412 | 1151.28866 | 1276.58 | -1.52983794 | 1.45E-20 | 9.12E-20 |
| gene-BPHYT_RS11075 | 1502.03311 | 1348.43615 | 1905.33114 | 651.724308 | 586.858181 | 545.941657 | 1.41422671 | 1.48E-20 | 9.30E-20 |
| gene-BPHYT_RS13635 | 12333.1124 | 14509.557 | 13970.7339 | 27562.7126 | 26092.1383 | 26058.5287 | -0.96589343 | 1.85E-20 | 1.16E-19 |
| gene-BPHYT_RS18640 | 657.6383 | 628.345642 | 708.653045 | 1359.64874 | 1533.80556 | 1421.44014 | -1.11257831 | 1.89E-20 | 1.18E-19 |
| gene-BPHYT_RS24930 | 172.390622 | 147.218504 | 248.079402 | 27.6070811 | 18.689751 | 32.5935318 | 2.82725019 | 1.95E-20 | 1.22E-19 |
| gene-BPHYT_RS02085 | 4024.04407 | 3227.07227 | 2900.69891 | 1332.04166 | 1488.95016 | 1366.21221 | 1.2786069 | 2.00E-20 | 1.25E-19 |
| gene-BPHYT_RS03340 | 744.631716 | 792.632957 | 857.093998 | 1803.33398 | 1617.28645 | 1677.66151 | -1.09154786 | 2.43E-20 | 1.52E-19 |
| gene-BPHYT_RS12140 | 13874.2527 | 16670.8953 | 19931.7565 | 40429.5843 | 41103.7463 | 37763.2281 | -1.2409284 | 2.62E-20 | 1.64E-19 |
| gene-BPHYT_RS04980 | 2672.05465 | 3219.60466 | 3489.37913 | 6731.19795 | 7002.4267 | 6630.97297 | -1.11865765 | 2.73E-20 | 1.70E-19 |
| gene-BPHYT_RS18645 | 447.736755 | 454.45712 | 489.041771 | 986.953149 | 1005.5086 | 1090.07256 | -1.14880552 | 3.00E-20 | 1.86E-19 |
| gene-BPHYT_RS07995 | 827.634608 | 899.313032 | 728.987422 | 245.505828 | 237.982829 | 342.232084 | 1.56819044 | 3.24E-20 | 2.01E-19 |
| gene-BPHYT_RS14880 | 6266.71837 | 7965.8012 | 10124.4864 | 22511.6027 | 20728.1798 | 21651.1589 | -1.41386514 | 3.30E-20 | 2.05E-19 |
| gene-BPHYT_RS33960 | 2510.03939 | 2843.024 | 2819.3614 | 1376.41019 | 1375.56567 | 1331.80792 | 1.00062557 | 3.31E-20 | 2.05E-19 |
| gene-BPHYT_RS17165 | 1824.46742 | 1812.49448 | 1556.59657 | 3604.69602 | 3437.6682 | 3481.17027 | -1.01860056 | 3.57E-20 | 2.21E-19 |
| gene-BPHYT_RS11490 | 177.977356 | 167.487718 | 117.939388 | 25.6351467 | 9.96786719 | 16.2967659 | 3.13623811 | 3.87E-20 | 2.40E-19 |
| gene-BPHYT_RS13625 | 2789.37604 | 3251.60869 | 3490.39584 | 6815.00516 | 6748.24609 | 6332.19893 | -1.06205431 | 4.51E-20 | 2.79E-19 |
| gene-BPHYT_RS21350 | 5357.67707 | 5355.33977 | 5934.58798 | 2273.64032 | 2757.36126 | 2792.17922 | 1.08960038 | 4.63E-20 | 2.86E-19 |
| gene-BPHYT_RS35315 | 332.809674 | 268.833789 | 291.798313 | 694.120896 | 809.889209 | 905.375883 | -1.42882089 | 4.68E-20 | 2.89E-19 |
| gene-BPHYT_RS24860 | 47.886284 | 84.2772593 | 38.6353167 | 272.126942 | 346.383385 | 269.802013 | -2.38582074 | 4.72E-20 | 2.90E-19 |
| gene-BPHYT_RS13315 | 1352.78752 | 1307.89772 | 808.291493 | 20.7053108 | 67.2831035 | 28.0666524 | 4.91378566 | 4.89E-20 | 3.01E-19 |
| gene-BPHYT_RS24900 | 317.645684 | 251.764977 | 312.13269 | 70.0036699 | 83.4808877 | 86.9160848 | 1.87670832 | 5.55E-20 | 3.41E-19 |
| gene-BPHYT_RS02485 | 305.674113 | 323.240627 | 295.865188 | 703.980568 | 770.01774 | 928.01028 | -1.37832691 | 6.26E-20 | 3.84E-19 |
| gene-BPHYT_RS02505 | 446.938651 | 456.590721 | 381.269572 | 994.840887 | 948.193366 | 994.10272 | -1.19269381 | 6.47E-20 | 3.97E-19 |
| gene-BPHYT_RS22160 | 1937.00019 | 2067.45985 | 2173.74492 | 4055.28302 | 3813.95518 | 4072.38072 | -0.95132941 | 6.50E-20 | 3.98E-19 |
| gene-BPHYT_RS12740 | 1427.80937 | 1927.70896 | 2133.07617 | 4544.32274 | 4906.68262 | 4404.65367 | -1.33668888 | 6.85E-20 | 4.19E-19 |
| gene-BPHYT_RS02780 | 233.046582 | 185.623331 | 185.042832 | 47.3264248 | 42.3634355 | 31.6881559 | 2.32328134 | 6.89E-20 | 4.21E-19 |
| gene-BPHYT_RS08870 | 6708.86839 | 7499.60927 | 8893.23986 | 17777.9743 | 16655.0601 | 16062.2735 | -1.12829883 | 7.04E-20 | 4.30E-19 |
| gene-BPHYT_RS24730 | 930.590119 | 774.497345 | 781.856803 | 298.748056 | 307.757899 | 363.961105 | 1.35681039 | 7.87E-20 | 4.80E-19 |
| gene-BPHYT_RS09435 | 782.940743 | 790.499356 | 764.572582 | 1505.57189 | 1576.169 | 1653.21636 | -1.01819504 | 8.04E-20 | 4.89E-19 |
| gene-BPHYT_RS24315 | 1055.09446 | 1074.26836 | 971.98323 | 474.250215 | 469.735741 | 487.092225 | 1.11541551 | 8.48E-20 | 5.16E-19 |
| gene-BPHYT_RS06735 | 2636.13993 | 2568.85621 | 1949.05005 | 955.4022 | 1064.06982 | 926.199528 | 1.2813147 | 1.06E-19 | 6.46E-19 |
| gene-BPHYT_RS17425 | 1785.36029 | 2081.32826 | 1908.3813 | 3701.3208 | 3774.08371 | 3836.98299 | -0.9706107 | 1.09E-19 | 6.62E-19 |
| gene-BPHYT_RS15425 | 1039.93047 | 952.65307 | 944.53182 | 1943.34132 | 1898.8787 | 2047.05487 | -1.00321263 | 1.38E-19 | 8.35E-19 |
| gene-BPHYT_RS34770 | 216.286383 | 187.756932 | 200.293615 | 524.534541 | 490.917459 | 574.913686 | -1.39487454 | 1.39E-19 | 8.44E-19 |
| gene-BPHYT_RS06205 | 275.346133 | 217.627353 | 171.825487 | 34.5088514 | 14.9518008 | 43.4580424 | 2.8118794 | 1.43E-19 | 8.65E-19 |
| gene-BPHYT_RS04470 | 5260.3083 | 5118.51 | 3843.19729 | 10195.8866 | 11434.3897 | 11379.6695 | -1.21459418 | 1.47E-19 | 8.90E-19 |
| gene-BPHYT_RS13780 | 113.330872 | 88.5444623 | 110.822356 | 0.98596718 | 7.47590039 | 3.62150353 | 4.77620633 | 1.51E-19 | 9.14E-19 |
| gene-BPHYT_RS16555 | 3846.06671 | 4708.85851 | 4357.65703 | 2148.42249 | 2136.86153 | 2044.33874 | 1.02838569 | 1.56E-19 | 9.37E-19 |
| gene-BPHYT_RS22330 | 522.7586 | 623.011638 | 546.994746 | 164.656519 | 224.277012 | 207.331077 | 1.50753664 | 2.01E-19 | 1.21E-18 |
| gene-BPHYT_RS34125 | 514.777553 | 418.185894 | 630.365693 | 174.516191 | 132.07424 | 160.251531 | 1.7396136 | 2.02E-19 | 1.22E-18 |
| gene-BPHYT_RS15860 | 17133.7124 | 15884.6632 | 14497.3942 | 29241.8147 | 32123.944 | 30559.1522 | -0.95195768 | 2.06E-19 | 1.23E-18 |
| gene-BPHYT_RS06480 | 1263.39979 | 1451.91582 | 1595.23189 | 580.73467 | 667.847102 | 524.212636 | 1.28349264 | 2.06E-19 | 1.24E-18 |
| gene-BPHYT_RS05575 | 3796.58422 | 3901.29034 | 4436.9611 | 8073.09929 | 7578.07103 | 7899.40458 | -0.9568498 | 2.08E-19 | 1.24E-18 |
| gene-BPHYT_RS06065 | 3543.58502 | 3731.66902 | 3327.72083 | 6659.22235 | 6614.92586 | 6442.65478 | -0.89496441 | 2.12E-19 | 1.27E-18 |
| gene-BPHYT_RS23815 | 738.246878 | 597.40842 | 578.513031 | 250.435664 | 241.720779 | 211.857957 | 1.44630366 | 2.40E-19 | 1.44E-18 |
| gene-BPHYT_RS37710 | 493.228725 | 344.576642 | 724.920547 | 1722.48467 | 2485.73688 | 1852.39906 | -1.95479145 | 2.57E-19 | 1.53E-18 |
| gene-BPHYT_RS12985 | 3085.4729 | 3179.06624 | 2767.50874 | 5580.57425 | 5862.35189 | 5807.98629 | -0.93344744 | 2.75E-19 | 1.64E-18 |
| gene-BPHYT_RS16795 | 423.793613 | 544.068383 | 488.025053 | 1113.15695 | 1088.98949 | 1137.15211 | -1.20042766 | 2.78E-19 | 1.66E-18 |
| Novel00352 | 1574.66064 | 1560.7295 | 1631.83377 | 3104.81066 | 3025.24769 | 3658.62394 | -1.03844285 | 3.21E-19 | 1.91E-18 |
| gene-BPHYT_RS01935 | 5303.40595 | 6958.74129 | 8517.05388 | 2535.90759 | 2834.61223 | 2326.81602 | 1.43286329 | 3.79E-19 | 2.26E-18 |
| gene-BPHYT_RS30690 | 203.516707 | 224.028158 | 227.745025 | 56.2001294 | 24.919668 | 46.17417 | 2.34274951 | 4.44E-19 | 2.64E-18 |
| gene-BPHYT_RS02300 | 895.473511 | 848.106596 | 727.970703 | 1778.6848 | 1706.99726 | 1761.86147 | -1.08545894 | 4.52E-19 | 2.68E-18 |
| gene-BPHYT_RS06270 | 938.571166 | 918.515446 | 829.642589 | 1778.6848 | 1809.16789 | 1777.25286 | -0.99710461 | 4.74E-19 | 2.81E-18 |
| gene-BPHYT_RS15655 | 14871.8836 | 16520.4764 | 17124.5958 | 8296.91384 | 8977.31039 | 8180.97648 | 0.93058659 | 4.75E-19 | 2.81E-18 |
| gene-BPHYT_RS08925 | 1522.78383 | 1664.20917 | 1598.28205 | 2981.56476 | 3306.83994 | 2998.60492 | -0.95666225 | 4.92E-19 | 2.91E-18 |
| gene-BPHYT_RS17185 | 1856.39161 | 2293.62161 | 2200.17961 | 4566.01402 | 4151.61668 | 4399.22142 | -1.04740522 | 5.04E-19 | 2.98E-18 |
| gene-BPHYT_RS16445 | 5196.45992 | 7011.01453 | 7924.30679 | 2529.00582 | 2889.4355 | 2445.42026 | 1.35629001 | 5.05E-19 | 2.98E-18 |
| gene-BPHYT_RS14530 | 359.14713 | 359.511853 | 359.918476 | 835.114203 | 755.065939 | 799.446905 | -1.14840836 | 5.07E-19 | 2.99E-18 |
| gene-BPHYT_RS15715 | 6959.47327 | 6326.12845 | 5795.2975 | 3175.80029 | 3360.41723 | 2995.8888 | 1.00175864 | 5.22E-19 | 3.07E-18 |
| gene-BPHYT_RS03590 | 20474.5788 | 37087.3281 | 22816.1879 | 80677.7507 | 87247.4955 | 78096.8183 | -1.61396221 | 5.29E-19 | 3.12E-18 |
| gene-BPHYT_RS17100 | 13888.6186 | 14691.9799 | 14372.3378 | 8333.39462 | 7874.61508 | 7922.03898 | 0.83184382 | 5.57E-19 | 3.28E-18 |
| gene-BPHYT_RS22055 | 622.521692 | 612.343631 | 661.883977 | 1239.36075 | 1409.20722 | 1317.32191 | -1.06361717 | 5.71E-19 | 3.35E-18 |
| gene-BPHYT_RS15730 | 7163.78809 | 7638.29337 | 6779.48135 | 12730.8083 | 13597.4168 | 13535.3695 | -0.88528668 | 6.43E-19 | 3.77E-18 |
| gene-BPHYT_RS11815 | 345.57935 | 304.038214 | 277.564249 | 733.559584 | 776.247657 | 717.963075 | -1.26125562 | 6.70E-19 | 3.93E-18 |
| gene-BPHYT_RS12555 | 6598.72993 | 6478.68096 | 5861.38422 | 11911.4695 | 11433.1437 | 11520.9081 | -0.88038506 | 6.95E-19 | 4.07E-18 |
| gene-BPHYT_RS03790 | 1554.70802 | 1417.7782 | 1612.51611 | 772.012304 | 603.055965 | 636.479246 | 1.18765101 | 8.08E-19 | 4.72E-18 |
| gene-BPHYT_RS07760 | 709.515108 | 660.349664 | 557.161935 | 191.277633 | 265.394464 | 227.249347 | 1.49930407 | 8.36E-19 | 4.87E-18 |
| gene-BPHYT_RS17865 | 802.095257 | 685.952882 | 525.64365 | 1808.26381 | 1623.51637 | 1758.23997 | -1.3644735 | 8.36E-19 | 4.87E-18 |
| gene-BPHYT_RS01610 | 4224.36835 | 5057.70236 | 4426.79391 | 8926.94687 | 9022.16579 | 8619.17841 | -0.95482406 | 8.40E-19 | 4.90E-18 |
| gene-BPHYT_RS17405 | 1522.78383 | 1551.12829 | 1426.45656 | 2772.53972 | 2799.7247 | 2941.56624 | -0.91984115 | 9.04E-19 | 5.26E-18 |
| gene-BPHYT_RS01715 | 83.0028923 | 39.4716278 | 50.835943 | 372.695595 | 257.918564 | 266.18051 | -2.35623148 | 9.27E-19 | 5.39E-18 |
| gene-BPHYT_RS26060 | 161.217156 | 161.086913 | 107.772199 | 14.7895077 | 26.1656514 | 11.7698865 | 3.06602698 | 9.45E-19 | 5.49E-18 |
| gene-BPHYT_RS13280 | 2349.62034 | 1739.95202 | 1855.51192 | 4293.88708 | 5189.52085 | 6355.7387 | -1.41321854 | 9.70E-19 | 5.63E-18 |
| gene-BPHYT_RS34275 | 964.110518 | 1422.0454 | 1181.42731 | 2928.32253 | 2843.33412 | 2784.03084 | -1.26331414 | 9.85E-19 | 5.71E-18 |
| gene-BPHYT_RS14145 | 1878.73854 | 1986.383 | 1634.88393 | 3559.34153 | 3744.18011 | 3674.01533 | -0.99693064 | 1.19E-18 | 6.89E-18 |
| gene-BPHYT_RS09205 | 1028.757 | 1008.12671 | 818.458682 | 359.878022 | 351.367318 | 430.053544 | 1.32124112 | 1.25E-18 | 7.25E-18 |
| gene-BPHYT_RS09530 | 3263.45025 | 3866.08592 | 3600.20148 | 6634.57317 | 7098.36742 | 6879.95134 | -0.94223539 | 1.40E-18 | 8.06E-18 |
| gene-BPHYT_RS18850 | 696.745432 | 608.076428 | 579.52975 | 1329.08376 | 1724.44102 | 1422.34551 | -1.24576529 | 1.42E-18 | 8.17E-18 |
| gene-BPHYT_RS34940 | 1664.84647 | 1047.59834 | 795.074148 | 290.860319 | 313.987816 | 325.029942 | 1.91653189 | 1.43E-18 | 8.25E-18 |
| gene-BPHYT_RS18135 | 12456.8187 | 16583.4177 | 16927.3523 | 33930.0886 | 33749.9523 | 33068.8541 | -1.13219034 | 1.44E-18 | 8.32E-18 |
| gene-BPHYT_RS22495 | 11511.0646 | 8720.02933 | 1648.10127 | 54.228195 | 84.7268711 | 69.713943 | 6.713118 | 1.67E-18 | 9.61E-18 |
| gene-BPHYT_RS32765 | 512.383239 | 337.109037 | 238.928932 | 1543.03864 | 1194.89808 | 1317.32191 | -1.89425092 | 1.71E-18 | 9.81E-18 |
| gene-BPHYT_RS33760 | 414.216357 | 368.046259 | 335.517224 | 804.549221 | 919.535748 | 995.913471 | -1.28109019 | 1.73E-18 | 9.89E-18 |
| gene-BPHYT_RS07035 | 699.937851 | 706.222097 | 658.833821 | 1398.10146 | 1340.67814 | 1563.58415 | -1.05962834 | 1.73E-18 | 9.89E-18 |
| gene-BPHYT_RS16865 | 3150.91749 | 3210.00346 | 3492.42928 | 1738.26014 | 1642.20612 | 1787.21199 | 0.93044539 | 1.75E-18 | 1.00E-17 |
| gene-BPHYT_RS23245 | 636.089472 | 697.687691 | 732.037579 | 1419.79274 | 1365.59781 | 1437.7369 | -1.0331242 | 1.76E-18 | 1.00E-17 |
| gene-BPHYT_RS07930 | 1136.50114 | 1292.96251 | 1218.02919 | 471.292313 | 608.039898 | 504.294367 | 1.2058661 | 1.84E-18 | 1.05E-17 |
| gene-BPHYT_RS02050 | 450.929174 | 481.127138 | 397.537074 | 959.346068 | 976.850984 | 976.900578 | -1.13154785 | 2.17E-18 | 1.24E-17 |
| gene-BPHYT_RS10605 | 19.1545136 | 25.603218 | 40.6687544 | 175.502158 | 206.833244 | 143.954765 | -2.63502757 | 2.24E-18 | 1.28E-17 |
| gene-BPHYT_RS26470 | 110.138453 | 92.8116653 | 136.240327 | 11.8316062 | 14.9518008 | 9.05375883 | 3.26672468 | 2.32E-18 | 1.32E-17 |
| gene-BPHYT_RS24155 | 793.316105 | 983.590292 | 1576.93095 | 306.635794 | 326.44765 | 299.679417 | 1.846158 | 2.55E-18 | 1.45E-17 |
| gene-BPHYT_RS18975 | 134.081595 | 103.479673 | 184.026114 | 491.997624 | 527.050978 | 429.148169 | -1.77891159 | 2.59E-18 | 1.47E-17 |
| gene-BPHYT_RS08085 | 1956.95281 | 2018.38702 | 2105.62476 | 1086.53584 | 963.145167 | 1050.23602 | 0.9709202 | 2.67E-18 | 1.52E-17 |
| gene-BPHYT_RS23080 | 389.47511 | 357.378251 | 451.423174 | 862.721285 | 955.669267 | 1034.84463 | -1.25188156 | 2.76E-18 | 1.57E-17 |
| gene-BPHYT_RS17850 | 11355.4341 | 13579.3068 | 10496.6055 | 23305.3063 | 25193.7843 | 24207.9404 | -1.0371119 | 2.87E-18 | 1.63E-17 |
| gene-BPHYT_RS02470 | 604.165283 | 657.149262 | 557.161935 | 1389.22776 | 1192.40611 | 1382.50897 | -1.12549356 | 2.90E-18 | 1.64E-17 |
| gene-BPHYT_RS05235 | 2452.57585 | 2738.47753 | 2764.45858 | 4800.67421 | 5047.47875 | 4993.148 | -0.89995684 | 3.08E-18 | 1.74E-17 |
| gene-BPHYT_RS33795 | 329.617255 | 327.50783 | 275.530811 | 74.9335059 | 112.138506 | 78.7677018 | 1.82291167 | 3.16E-18 | 1.79E-17 |
| gene-BPHYT_RS13475 | 173.188727 | 128.01609 | 157.591423 | 464.390543 | 398.714688 | 453.593317 | -1.51750138 | 3.18E-18 | 1.79E-17 |
| gene-BPHYT_RS05630 | 2070.28368 | 2311.75723 | 1931.76583 | 987.939117 | 1088.98949 | 940.685542 | 1.06606 | 3.39E-18 | 1.91E-17 |
| gene-BPHYT_RS08475 | 110.936558 | 119.481684 | 91.5046973 | 304.663859 | 378.778953 | 331.367573 | -1.65409033 | 3.45E-18 | 1.94E-17 |
| gene-BPHYT_RS05805 | 129.292967 | 97.0788683 | 102.688605 | 341.144645 | 316.479783 | 401.986892 | -1.68258578 | 3.98E-18 | 2.24E-17 |
| gene-BPHYT_RS10215 | 152.438004 | 177.088925 | 93.5381351 | 17.7474093 | 19.9357344 | 11.7698865 | 3.11559932 | 4.11E-18 | 2.31E-17 |
| gene-BPHYT_RS35465 | 666.417452 | 637.946849 | 659.85054 | 291.846286 | 285.330198 | 298.774041 | 1.16445161 | 4.35E-18 | 2.44E-17 |
| gene-BPHYT_RS19035 | 498.815458 | 698.754491 | 528.693807 | 1533.17897 | 1416.68312 | 1309.17353 | -1.3047439 | 4.38E-18 | 2.45E-17 |
| gene-BPHYT_RS02765 | 250.604886 | 235.762966 | 187.07627 | 550.169688 | 589.350147 | 621.087856 | -1.38405012 | 4.57E-18 | 2.56E-17 |
| gene-BPHYT_RS23200 | 437.361394 | 346.710244 | 310.099252 | 100.568653 | 63.5451533 | 113.171985 | 1.97021619 | 4.72E-18 | 2.64E-17 |
| gene-BPHYT_RS29635 | 461.304536 | 536.600777 | 503.275835 | 186.347797 | 198.11136 | 208.236453 | 1.33894668 | 5.02E-18 | 2.81E-17 |
| gene-BPHYT_RS12675 | 19008.4604 | 21783.0045 | 20977.9602 | 36413.74 | 37413.1435 | 38051.1376 | -0.85702812 | 5.76E-18 | 3.22E-17 |
| gene-BPHYT_RS22515 | 516.373762 | 414.985492 | 303.998939 | 1311.33635 | 1196.14406 | 1082.82956 | -1.5362844 | 5.99E-18 | 3.34E-17 |
| gene-BPHYT_RS19020 | 1712.73276 | 2087.72907 | 1998.86928 | 4403.32944 | 4329.79231 | 3626.03041 | -1.09200655 | 6.11E-18 | 3.41E-17 |
| gene-BPHYT_RS17380 | 2773.41395 | 2677.66988 | 2500.11168 | 1376.41019 | 1420.42107 | 1435.92615 | 0.90980965 | 6.60E-18 | 3.67E-17 |
| gene-BPHYT_RS08465 | 1585.036 | 1719.68281 | 1600.31549 | 3095.93695 | 3014.03384 | 2947.90388 | -0.88523237 | 6.71E-18 | 3.73E-17 |
| gene-BPHYT_RS24495 | 478.064735 | 334.975436 | 432.105515 | 141.979274 | 129.582273 | 135.806382 | 1.61266225 | 7.56E-18 | 4.20E-17 |
| gene-BPHYT_RS14785 | 264.172667 | 248.564575 | 400.587231 | 921.879315 | 1166.24046 | 824.797429 | -1.67318282 | 8.23E-18 | 4.57E-17 |
| gene-BPHYT_RS20935 | 371.118701 | 422.453097 | 391.436761 | 863.707252 | 857.236578 | 824.797429 | -1.10458724 | 8.23E-18 | 4.57E-17 |
| gene-BPHYT_RS02495 | 221.075011 | 235.762966 | 234.862057 | 529.464377 | 537.018845 | 549.563161 | -1.22577905 | 8.84E-18 | 4.90E-17 |
| gene-BPHYT_RS15980 | 2378.35211 | 2388.56688 | 2123.9257 | 1106.25518 | 1204.86595 | 1215.91981 | 0.96651334 | 1.07E-17 | 5.93E-17 |
| gene-BPHYT_RS23155 | 513.181343 | 627.278841 | 433.122234 | 160.712651 | 184.405543 | 191.939687 | 1.55044894 | 1.07E-17 | 5.94E-17 |
| gene-BPHYT_RS03485 | 1660.85595 | 1684.47839 | 1595.23189 | 850.889678 | 823.595026 | 891.795245 | 0.94421993 | 1.18E-17 | 6.54E-17 |
| gene-BPHYT_RS19995 | 188.352717 | 233.629364 | 295.865188 | 65.073834 | 37.379502 | 47.0795459 | 2.25164691 | 1.23E-17 | 6.80E-17 |
| gene-BPHYT_RS23725 | 363.935758 | 440.58871 | 389.403323 | 869.623055 | 849.760678 | 908.997387 | -1.14112499 | 1.30E-17 | 7.17E-17 |
| gene-BPHYT_RS31065 | 0 | 0 | 3.05015658 | 166.628454 | 160.731858 | 155.724652 | -7.40748789 | 1.30E-17 | 7.17E-17 |
| gene-BPHYT_RS10915 | 719.890469 | 679.552078 | 566.312405 | 286.91645 | 220.539062 | 237.208481 | 1.39894824 | 1.31E-17 | 7.20E-17 |
| gene-BPHYT_RS14830 | 908.243186 | 1037.99713 | 1032.98636 | 439.741363 | 461.013857 | 485.281473 | 1.10268399 | 1.35E-17 | 7.43E-17 |
| gene-BPHYT_RS12075 | 3576.30731 | 4069.84486 | 3623.58601 | 6810.07533 | 6818.02116 | 7011.23084 | -0.87322578 | 1.38E-17 | 7.57E-17 |
| gene-BPHYT_RS08240 | 253.797305 | 211.226549 | 207.410647 | 522.562607 | 571.90638 | 583.062069 | -1.31496286 | 1.40E-17 | 7.65E-17 |
| gene-BPHYT_RS28275 | 313.65516 | 359.511853 | 401.603949 | 130.147668 | 119.614406 | 112.26661 | 1.56865749 | 1.46E-17 | 8.01E-17 |
| gene-BPHYT_RS24345 | 369.522492 | 277.368195 | 382.286291 | 806.521155 | 854.744611 | 861.917841 | -1.29153768 | 1.57E-17 | 8.56E-17 |
| gene-BPHYT_RS14000 | 7092.75676 | 8090.61689 | 7605.05707 | 13647.7577 | 14184.275 | 13330.7545 | -0.85314013 | 1.78E-17 | 9.74E-17 |
| gene-BPHYT_RS19375 | 586.606979 | 460.857924 | 421.938327 | 1081.606 | 1239.75348 | 1282.01225 | -1.29138808 | 1.90E-17 | 1.04E-16 |
| gene-BPHYT_RS15205 | 385.484586 | 352.044248 | 317.216284 | 117.330095 | 110.892523 | 133.090255 | 1.54270208 | 1.92E-17 | 1.05E-16 |
| gene-BPHYT_RS34160 | 173.188727 | 202.692143 | 161.658299 | 31.5509498 | 46.1013857 | 41.6472906 | 2.17808201 | 2.02E-17 | 1.10E-16 |
| gene-BPHYT_RS21505 | 861.155007 | 838.50539 | 659.85054 | 239.590025 | 335.169534 | 299.679417 | 1.43533 | 2.05E-17 | 1.11E-16 |
| gene-BPHYT_RS31045 | 1.59620947 | 1.06680075 | 0 | 148.881045 | 163.223825 | 159.346155 | -7.38042802 | 2.08E-17 | 1.13E-16 |
| gene-BPHYT_RS30685 | 214.690173 | 177.088925 | 181.992676 | 34.5088514 | 54.8232695 | 42.5526665 | 2.13605861 | 2.09E-17 | 1.13E-16 |
| gene-BPHYT_RS13055 | 944.157899 | 821.436578 | 682.218355 | 280.01468 | 346.383385 | 320.503063 | 1.37313784 | 2.15E-17 | 1.16E-16 |
| gene-BPHYT_RS24715 | 2736.70113 | 2503.78136 | 2794.96014 | 1430.63838 | 1250.96733 | 1429.58852 | 0.96602432 | 2.19E-17 | 1.18E-16 |
| gene-BPHYT_RS08995 | 1812.49585 | 1727.15042 | 1600.31549 | 741.447321 | 812.381176 | 908.997387 | 1.06118195 | 2.54E-17 | 1.37E-16 |
| gene-BPHYT_RS24140 | 671.206081 | 762.762536 | 1293.26639 | 247.477763 | 262.902497 | 262.559006 | 1.81838487 | 2.64E-17 | 1.43E-16 |
| gene-BPHYT_RS19370 | 1277.76568 | 1147.87761 | 1091.95606 | 2240.11744 | 2379.82829 | 2229.03542 | -0.96009852 | 2.76E-17 | 1.49E-16 |
| gene-BPHYT_RS06605 | 956.12947 | 1179.88163 | 1176.34372 | 2185.88924 | 2369.86042 | 2267.06121 | -1.04357527 | 2.80E-17 | 1.51E-16 |
| gene-BPHYT_RS12760 | 1655.26922 | 1754.88723 | 1730.4555 | 3477.50625 | 2979.14631 | 3301.00047 | -0.925271 | 2.83E-17 | 1.52E-16 |
| gene-BPHYT_RS24190 | 2823.69455 | 2300.02242 | 3098.95908 | 5666.3534 | 5954.55466 | 5617.85735 | -1.06767301 | 2.96E-17 | 1.59E-16 |
| gene-BPHYT_RS18145 | 1016.78543 | 1198.01724 | 1072.6384 | 2088.27849 | 2204.14463 | 2116.76881 | -0.96389996 | 2.98E-17 | 1.61E-16 |
| gene-BPHYT_RS02685 | 1007.20817 | 1262.02529 | 1139.74184 | 507.773099 | 549.478679 | 487.092225 | 1.14278605 | 3.24E-17 | 1.74E-16 |
| gene-BPHYT_RS07395 | 6.38483787 | 3.20040225 | 1.01671886 | 101.55462 | 88.4648213 | 69.713943 | -4.53898624 | 3.26E-17 | 1.75E-16 |
| gene-BPHYT_RS16110 | 711.909422 | 635.813247 | 633.415849 | 287.902417 | 296.544049 | 298.774041 | 1.16663531 | 3.55E-17 | 1.91E-16 |
| gene-BPHYT_RS02640 | 10087.2457 | 11220.6103 | 11126.9712 | 19021.2789 | 19568.1693 | 18667.9453 | -0.81999499 | 3.80E-17 | 2.04E-16 |
| gene-BPHYT_RS14035 | 187.554612 | 227.22856 | 257.229871 | 40.4246545 | 68.5290869 | 36.2150353 | 2.23007921 | 4.81E-17 | 2.58E-16 |
| gene-BPHYT_RS28225 | 155.630423 | 154.686109 | 152.507829 | 28.5930483 | 26.1656514 | 7.24300706 | 2.92656715 | 5.00E-17 | 2.67E-16 |
| gene-BPHYT_RS24890 | 121.311919 | 122.682086 | 110.822356 | 16.7614421 | 17.4437676 | 21.7290212 | 2.65754948 | 5.02E-17 | 2.69E-16 |
| Novel00768 | 261.778353 | 181.356128 | 265.363622 | 44.3685232 | 37.379502 | 67.9031912 | 2.22993063 | 5.03E-17 | 2.69E-16 |
| gene-BPHYT_RS04945 | 1677.61615 | 1622.60394 | 1821.9602 | 905.117873 | 885.894196 | 876.403855 | 0.94128523 | 5.07E-17 | 2.71E-16 |
| gene-BPHYT_RS03345 | 3158.10043 | 3956.76398 | 4246.83468 | 7863.08828 | 7831.00566 | 7829.69064 | -1.05037418 | 5.25E-17 | 2.80E-16 |
| gene-BPHYT_RS29190 | 1043.12289 | 1003.85951 | 1057.38761 | 1845.73057 | 2106.95793 | 1983.67856 | -0.93465863 | 5.73E-17 | 3.05E-16 |
| gene-BPHYT_RS12175 | 5799.02899 | 6775.25157 | 6898.43746 | 11878.9326 | 11951.4728 | 12515.0108 | -0.90057434 | 6.04E-17 | 3.21E-16 |
| gene-BPHYT_RS23840 | 515.575658 | 266.700188 | 407.704263 | 92.6809151 | 54.8232695 | 105.023602 | 2.22763218 | 6.52E-17 | 3.47E-16 |
| gene-BPHYT_RS34245 | 151.639899 | 82.1436578 | 140.307203 | 546.225819 | 385.00887 | 444.539559 | -1.87362334 | 6.63E-17 | 3.52E-16 |
| gene-BPHYT_RS05920 | 4130.19199 | 3599.38573 | 3586.98414 | 1866.43588 | 1954.94795 | 2085.98603 | 0.93799941 | 6.82E-17 | 3.62E-16 |
| gene-BPHYT_RS24670 | 203.516707 | 229.362161 | 130.140014 | 25.6351467 | 38.6254854 | 38.0257871 | 2.46520363 | 6.97E-17 | 3.69E-16 |
| gene-BPHYT_RS06070 | 292.106332 | 348.843845 | 238.928932 | 723.699912 | 745.098072 | 724.300706 | -1.31825101 | 7.20E-17 | 3.81E-16 |
| gene-BPHYT_RS03355 | 2743.08597 | 3283.61271 | 3986.55465 | 1428.66645 | 1542.52745 | 1425.96702 | 1.18723243 | 7.98E-17 | 4.22E-16 |
| gene-BPHYT_RS09095 | 5844.52096 | 3642.05776 | 2978.98626 | 1379.36809 | 1344.41609 | 1347.19931 | 1.61488454 | 8.91E-17 | 4.71E-16 |
| gene-BPHYT_RS17305 | 3923.48287 | 4417.62191 | 3727.29134 | 2047.85384 | 2156.79726 | 2176.52362 | 0.9192414 | 9.19E-17 | 4.85E-16 |
| Novel00759 | 352.762292 | 293.370206 | 363.985352 | 856.805481 | 712.702504 | 990.481216 | -1.34212498 | 9.38E-17 | 4.95E-16 |
| gene-BPHYT_RS14010 | 194.737555 | 257.098981 | 192.159864 | 516.646804 | 566.922446 | 568.576055 | -1.36227825 | 9.65E-17 | 5.08E-16 |
| gene-BPHYT_RS16785 | 7708.09551 | 8624.01727 | 9153.51989 | 15709.4151 | 16056.9881 | 15038.2934 | -0.87707672 | 9.71E-17 | 5.11E-16 |
| gene-BPHYT_RS04395 | 19.9526183 | 5.33400375 | 23.3845338 | 117.330095 | 150.763991 | 130.374127 | -3.01118831 | 1.01E-16 | 5.33E-16 |
| gene-BPHYT_RS02400 | 366.330073 | 416.052293 | 458.540206 | 161.698618 | 148.272024 | 156.630028 | 1.40789885 | 1.10E-16 | 5.79E-16 |
| gene-BPHYT_RS28295 | 133.28349 | 114.14768 | 71.1703202 | 10.845639 | 11.2138506 | 8.14838295 | 3.41454134 | 1.12E-16 | 5.87E-16 |
| gene-BPHYT_RS14340 | 1608.97914 | 1343.10215 | 1168.20997 | 3333.55504 | 2767.32913 | 3100.00702 | -1.15837153 | 1.19E-16 | 6.22E-16 |
| gene-BPHYT_RS14415 | 15209.4819 | 16597.2861 | 18972.9906 | 32595.0891 | 31990.6238 | 31271.683 | -0.9167007 | 1.43E-16 | 7.51E-16 |
| gene-BPHYT_RS24660 | 249.008677 | 268.833789 | 237.912213 | 85.7791449 | 44.8554023 | 63.3763118 | 1.94966254 | 1.45E-16 | 7.57E-16 |
| gene-BPHYT_RS32780 | 324.828626 | 388.315473 | 379.236135 | 125.217832 | 51.0853193 | 82.3892054 | 2.06636636 | 1.57E-16 | 8.20E-16 |
| gene-BPHYT_RS08865 | 5094.30251 | 6555.49061 | 7462.71643 | 13916.9268 | 14762.4113 | 13554.3823 | -1.14408568 | 1.59E-16 | 8.32E-16 |
| gene-BPHYT_RS30260 | 155.630423 | 180.289327 | 145.390797 | 36.4807857 | 23.6736846 | 38.931163 | 2.26119346 | 1.64E-16 | 8.55E-16 |
| gene-BPHYT_RS03290 | 5920.34091 | 7101.69259 | 7053.99545 | 12821.5172 | 12401.2728 | 12262.411 | -0.90108471 | 1.69E-16 | 8.82E-16 |
| gene-BPHYT_RS19615 | 1501.235 | 1450.84902 | 1690.80346 | 2842.54339 | 2955.47262 | 2884.52756 | -0.90310765 | 1.76E-16 | 9.16E-16 |
| gene-BPHYT_RS15680 | 5182.09403 | 6781.65237 | 7494.23471 | 14091.443 | 14525.6745 | 13744.5113 | -1.12262686 | 1.89E-16 | 9.81E-16 |
| gene-BPHYT_RS14840 | 1166.82912 | 1223.62046 | 1234.2967 | 2107.99784 | 2262.70585 | 2261.62896 | -0.87191673 | 2.03E-16 | 1.06E-15 |
| gene-BPHYT_RS29740 | 2243.47241 | 2723.54232 | 2625.1681 | 1311.33635 | 1308.28257 | 1277.48537 | 0.9616644 | 2.15E-16 | 1.12E-15 |
| gene-BPHYT_RS06655 | 15923.7856 | 16424.4644 | 17230.3345 | 27686.9444 | 28323.6946 | 27782.3644 | -0.75713761 | 2.26E-16 | 1.17E-15 |
| gene-BPHYT_RS26130 | 102.157406 | 103.479673 | 79.304071 | 12.8175734 | 7.47590039 | 9.05375883 | 3.26560269 | 2.32E-16 | 1.20E-15 |
| gene-BPHYT_RS14245 | 2189.99939 | 2295.75521 | 2791.90999 | 1234.43091 | 1052.85597 | 1153.44888 | 1.07974619 | 2.35E-16 | 1.22E-15 |
| gene-BPHYT_RS10600 | 235.440896 | 165.354116 | 215.544398 | 55.2141622 | 54.8232695 | 43.4580424 | 2.01391728 | 2.39E-16 | 1.24E-15 |
| gene-BPHYT_RS12990 | 536.326381 | 583.54001 | 512.426305 | 1036.25151 | 1145.05874 | 1121.76072 | -1.01706728 | 2.46E-16 | 1.27E-15 |
| gene-BPHYT_RS12060 | 1722.31001 | 2078.12786 | 2153.41054 | 3669.76985 | 4152.86267 | 4190.07959 | -1.01328598 | 2.59E-16 | 1.34E-15 |
| gene-BPHYT_RS05590 | 1991.27131 | 1688.74559 | 1402.05531 | 774.970205 | 735.130205 | 734.259841 | 1.17974321 | 2.74E-16 | 1.41E-15 |
| gene-BPHYT_RS08920 | 1485.27291 | 1525.52507 | 1611.49939 | 2696.62024 | 2764.83716 | 2949.71463 | -0.86415121 | 2.78E-16 | 1.43E-15 |
| gene-BPHYT_RS29750 | 8939.57112 | 9163.81844 | 7506.43534 | 4469.38924 | 4572.75907 | 4568.52671 | 0.91201823 | 2.94E-16 | 1.51E-15 |
| gene-BPHYT_RS26600 | 172.390622 | 203.758943 | 166.741893 | 431.853626 | 439.83214 | 477.13309 | -1.31582139 | 3.08E-16 | 1.58E-15 |
| gene-BPHYT_RS26845 | 16254.201 | 17189.3605 | 16249.2008 | 27446.3685 | 31240.5418 | 28608.0672 | -0.81283977 | 3.22E-16 | 1.66E-15 |
| gene-BPHYT_RS11425 | 14.3658852 | 12.801609 | 29.4848469 | 138.035406 | 112.138506 | 130.374127 | -2.75984807 | 3.24E-16 | 1.66E-15 |
| gene-BPHYT_RS33950 | 3057.53923 | 3377.49118 | 3197.58081 | 1662.34067 | 1244.73742 | 1633.29809 | 1.083672 | 3.25E-16 | 1.67E-15 |
| gene-BPHYT_RS18795 | 821.249771 | 760.628935 | 657.817102 | 273.11291 | 357.597235 | 265.275134 | 1.32725981 | 3.74E-16 | 1.91E-15 |
| gene-BPHYT_RS08045 | 8819.85541 | 11438.2376 | 14953.901 | 4683.34412 | 4941.57016 | 4196.41722 | 1.34919668 | 3.86E-16 | 1.97E-15 |
| gene-BPHYT_RS01470 | 3138.14781 | 3210.00346 | 2572.29871 | 5735.3711 | 5761.42723 | 5668.5584 | -0.9441537 | 3.99E-16 | 2.04E-15 |
| gene-BPHYT_RS34445 | 171.592518 | 195.224537 | 160.64158 | 413.120249 | 507.115243 | 435.4858 | -1.36131562 | 4.03E-16 | 2.06E-15 |
| gene-BPHYT_RS11120 | 711.111317 | 681.685679 | 581.563188 | 1602.19667 | 1274.64102 | 1418.72401 | -1.12132328 | 4.23E-16 | 2.16E-15 |
| gene-BPHYT_RS22500 | 3075.09754 | 2876.09482 | 1604.38236 | 854.833547 | 864.712479 | 784.960891 | 1.59359291 | 4.27E-16 | 2.18E-15 |
| gene-BPHYT_RS04845 | 1209.92678 | 1118.00719 | 1230.22982 | 2252.93501 | 2080.79228 | 2250.76445 | -0.88800499 | 4.37E-16 | 2.23E-15 |
| gene-BPHYT_RS11575 | 989.649869 | 1032.66313 | 975.033386 | 514.674869 | 509.60721 | 507.91587 | 0.96788833 | 4.51E-16 | 2.29E-15 |
| gene-BPHYT_RS34345 | 926.599595 | 1110.53958 | 953.68229 | 2086.30656 | 3365.40116 | 2339.49128 | -1.38102328 | 4.52E-16 | 2.30E-15 |
| gene-BPHYT_RS09135 | 2159.67141 | 2420.5709 | 2766.49202 | 1241.33268 | 1209.84988 | 1213.20368 | 1.00300345 | 4.98E-16 | 2.53E-15 |
| gene-BPHYT_RS14270 | 470.083688 | 677.418476 | 714.753358 | 171.55829 | 206.833244 | 240.829985 | 1.58572402 | 5.32E-16 | 2.70E-15 |
| gene-BPHYT_RS12080 | 4889.9877 | 5423.61501 | 5385.5598 | 8906.24156 | 9085.71094 | 9252.03615 | -0.79547363 | 5.90E-16 | 2.99E-15 |
| gene-BPHYT_RS29225 | 90.1858349 | 107.746876 | 133.190171 | 331.284973 | 328.939617 | 314.165431 | -1.56269674 | 6.24E-16 | 3.16E-15 |
| gene-BPHYT_RS08290 | 3973.76347 | 2536.85218 | 1810.77629 | 318.4674 | 432.356239 | 303.300921 | 2.98193013 | 6.47E-16 | 3.28E-15 |
| gene-BPHYT_RS03005 | 94.9744633 | 87.4776615 | 101.671886 | 292.832253 | 251.688647 | 282.477276 | -1.54245293 | 6.60E-16 | 3.34E-15 |
| gene-BPHYT_RS33610 | 3273.82562 | 3846.88351 | 3792.36135 | 6868.24739 | 6930.15966 | 6351.21182 | -0.88496617 | 7.37E-16 | 3.72E-15 |
| gene-BPHYT_RS23635 | 476.468526 | 691.286886 | 600.880846 | 207.053108 | 246.704713 | 190.128935 | 1.46009322 | 7.53E-16 | 3.80E-15 |
| gene-BPHYT_RS35445 | 623.319797 | 534.467176 | 485.991615 | 219.870682 | 241.720779 | 195.561191 | 1.32791398 | 7.93E-16 | 4.00E-15 |
| gene-BPHYT_RS02775 | 6613.09582 | 6161.84113 | 5451.64652 | 11309.0436 | 11811.9226 | 10710.5967 | -0.89208004 | 8.09E-16 | 4.07E-15 |
| gene-BPHYT_RS29705 | 138.072119 | 114.14768 | 95.5715728 | 20.7053108 | 18.689751 | 14.4860141 | 2.70037004 | 9.18E-16 | 4.62E-15 |
| gene-BPHYT_RS10360 | 438.957603 | 406.451086 | 278.580967 | 86.765112 | 130.828257 | 107.73973 | 1.79588686 | 9.20E-16 | 4.62E-15 |
| gene-BPHYT_RS19900 | 141.264538 | 148.285304 | 93.5381351 | 18.7333765 | 22.4277012 | 7.24300706 | 3.01899475 | 9.36E-16 | 4.70E-15 |
| gene-BPHYT_RS06415 | 741.439297 | 780.898149 | 682.218355 | 1422.75064 | 1369.33576 | 1416.00788 | -0.93300006 | 9.54E-16 | 4.79E-15 |
| gene-BPHYT_RS17685 | 5666.54361 | 5769.25846 | 4814.1638 | 9632.89937 | 9994.03284 | 10228.9367 | -0.87751398 | 9.82E-16 | 4.92E-15 |
| gene-BPHYT_RS15695 | 885.098149 | 832.104585 | 749.321799 | 1573.60362 | 1659.64989 | 1544.57126 | -0.95254952 | 9.87E-16 | 4.95E-15 |
| gene-BPHYT_RS26190 | 1797.33186 | 1790.09166 | 1654.20158 | 880.468694 | 994.294752 | 889.079117 | 0.92471717 | 1.02E-15 | 5.13E-15 |
| gene-BPHYT_RS26445 | 151.639899 | 114.14768 | 132.173452 | 18.7333765 | 27.4116348 | 28.0666524 | 2.42911345 | 1.03E-15 | 5.13E-15 |
| gene-BPHYT_RS09255 | 5443.07428 | 5194.25285 | 4140.0792 | 2473.79166 | 2273.9197 | 2518.75571 | 1.02391839 | 1.12E-15 | 5.58E-15 |
| gene-BPHYT_RS14815 | 53.4730171 | 83.2104585 | 155.557986 | 451.57297 | 444.816073 | 438.201927 | -2.19911258 | 1.28E-15 | 6.41E-15 |
| gene-BPHYT_RS25020 | 2855.61874 | 2346.96165 | 2090.37398 | 952.444298 | 1143.81276 | 1173.36714 | 1.15809759 | 1.33E-15 | 6.64E-15 |
| gene-BPHYT_RS29875 | 373.513015 | 403.250684 | 432.105515 | 138.035406 | 154.501941 | 174.737545 | 1.36865718 | 1.34E-15 | 6.69E-15 |
| gene-BPHYT_RS34215 | 4723.18381 | 4177.59174 | 4484.74689 | 2481.6794 | 2576.69367 | 2534.1471 | 0.81836679 | 1.43E-15 | 7.11E-15 |
| gene-BPHYT_RS17715 | 1985.68458 | 2176.27353 | 2211.36352 | 3747.66126 | 3764.11585 | 3700.27123 | -0.81535049 | 1.44E-15 | 7.16E-15 |
| gene-BPHYT_RS21485 | 859.558798 | 761.695736 | 697.469138 | 325.36917 | 375.041003 | 348.569715 | 1.14710111 | 1.48E-15 | 7.36E-15 |
| gene-BPHYT_RS11365 | 307.270322 | 322.173827 | 342.634256 | 705.952503 | 659.125218 | 676.315785 | -1.07206294 | 1.52E-15 | 7.55E-15 |
| gene-BPHYT_RS24695 | 7502.18449 | 6824.3244 | 6187.75098 | 3533.70638 | 3694.34078 | 3860.52277 | 0.88766896 | 1.70E-15 | 8.43E-15 |
| gene-BPHYT_RS35310 | 376.705434 | 361.645454 | 271.463935 | 796.661483 | 771.263724 | 779.528635 | -1.21528157 | 1.90E-15 | 9.39E-15 |
| gene-BPHYT_RS13825 | 404.6391 | 395.783078 | 334.500505 | 808.493089 | 768.771757 | 836.567316 | -1.08821915 | 1.92E-15 | 9.51E-15 |
| gene-BPHYT_RS15825 | 454.121593 | 469.39233 | 407.704263 | 179.446027 | 190.63546 | 191.034311 | 1.24693073 | 1.99E-15 | 9.85E-15 |
| gene-BPHYT_RS13290 | 334.405883 | 419.252695 | 288.748156 | 865.679186 | 771.263724 | 841.094195 | -1.25127025 | 2.35E-15 | 1.16E-14 |
| gene-BPHYT_RS26105 | 126.898653 | 182.422928 | 95.5715728 | 0 | 2.4919668 | 0.90537588 | 7.02849816 | 2.38E-15 | 1.18E-14 |
| gene-BPHYT_RS13855 | 8572.44294 | 9179.82046 | 9523.60556 | 14978.8134 | 15643.3216 | 15873.9554 | -0.76958289 | 2.39E-15 | 1.18E-14 |
| gene-BPHYT_RS09550 | 2301.73405 | 2340.56085 | 1806.70941 | 986.953149 | 1095.21941 | 1033.03388 | 1.05046658 | 2.47E-15 | 1.22E-14 |
| gene-BPHYT_RS07560 | 879.511416 | 856.641002 | 647.649913 | 277.056778 | 287.822165 | 366.677233 | 1.35380043 | 2.49E-15 | 1.23E-14 |
| gene-BPHYT_RS32515 | 316.049474 | 332.841834 | 382.286291 | 746.377157 | 698.996687 | 849.242578 | -1.15670877 | 2.80E-15 | 1.38E-14 |
| gene-BPHYT_RS08755 | 2022.39739 | 1847.6989 | 1452.89125 | 863.707252 | 761.295856 | 798.541529 | 1.13511119 | 3.01E-15 | 1.48E-14 |
| gene-BPHYT_RS19335 | 1494.05206 | 1588.46632 | 1834.16082 | 3100.86679 | 3112.46653 | 3023.05007 | -0.91011436 | 3.11E-15 | 1.52E-14 |
| gene-BPHYT_RS06650 | 12107.2488 | 12977.6311 | 11767.5041 | 21023.7782 | 21410.9787 | 20052.2651 | -0.76179807 | 3.13E-15 | 1.53E-14 |
| gene-BPHYT_RS26185 | 1456.54114 | 1512.72346 | 1170.24341 | 555.099524 | 660.371201 | 671.788905 | 1.13370185 | 3.15E-15 | 1.54E-14 |
| gene-BPHYT_RS23075 | 79.0123686 | 81.076857 | 74.2204767 | 230.716321 | 218.047095 | 263.464382 | -1.60608073 | 3.45E-15 | 1.69E-14 |
| gene-BPHYT_RS02645 | 18914.2841 | 20084.6577 | 21797.4356 | 34491.104 | 35650.077 | 34430.5395 | -0.78246924 | 3.49E-15 | 1.71E-14 |
| gene-BPHYT_RS28270 | 1181.99311 | 1196.95044 | 895.729315 | 413.120249 | 529.542944 | 445.444934 | 1.24081332 | 3.60E-15 | 1.76E-14 |
| gene-BPHYT_RS26135 | 103.753615 | 88.5444623 | 63.0365693 | 6.90177028 | 7.47590039 | 8.14838295 | 3.5032981 | 3.64E-15 | 1.78E-14 |
| gene-BPHYT_RS01420 | 892.281092 | 1046.53154 | 870.311344 | 441.713298 | 421.142389 | 474.416963 | 1.06868152 | 4.16E-15 | 2.03E-14 |
| gene-BPHYT_RS33475 | 1227.48508 | 1151.07801 | 1022.81917 | 548.197753 | 472.227708 | 585.778196 | 1.08064181 | 4.26E-15 | 2.07E-14 |
| gene-BPHYT_RS03095 | 761.391916 | 519.531965 | 392.45348 | 123.245898 | 193.127427 | 140.333262 | 1.88068195 | 4.28E-15 | 2.08E-14 |
| gene-BPHYT_RS09865 | 92.5801491 | 96.0120675 | 72.187039 | 10.845639 | 0 | 6.33763118 | 3.84928983 | 4.43E-15 | 2.15E-14 |
| gene-BPHYT_RS18140 | 11950.8203 | 16329.5191 | 16942.6031 | 32181.9688 | 31757.6249 | 30497.5866 | -1.06241337 | 5.08E-15 | 2.47E-14 |
| gene-BPHYT_RS17420 | 767.776753 | 882.24422 | 652.733508 | 1522.33333 | 1602.33465 | 1758.23997 | -1.08493329 | 5.16E-15 | 2.51E-14 |
| gene-BPHYT_RS06110 | 3652.92536 | 3418.0296 | 2677.02076 | 1538.1088 | 1586.13687 | 1618.81208 | 1.03954869 | 5.69E-15 | 2.76E-14 |
| gene-BPHYT_RS38580 | 384.686481 | 556.869992 | 497.175522 | 183.389896 | 186.89751 | 172.021418 | 1.40652561 | 5.70E-15 | 2.77E-14 |
| gene-BPHYT_RS26230 | 402.244786 | 480.060338 | 516.493181 | 197.193437 | 190.63546 | 183.791304 | 1.28959465 | 5.78E-15 | 2.80E-14 |
| gene-BPHYT_RS25800 | 138.072119 | 140.817699 | 150.474391 | 34.5088514 | 36.1335186 | 33.4989077 | 2.04598324 | 6.12E-15 | 2.96E-14 |
| gene-BPHYT_RS06625 | 7716.87467 | 8860.84703 | 10364.4321 | 4754.33375 | 4228.86765 | 3645.94868 | 1.09308939 | 6.24E-15 | 3.02E-14 |
| gene-BPHYT_RS12530 | 1954.55849 | 2040.78984 | 2165.61117 | 3877.80893 | 3450.12803 | 3639.61105 | -0.83253456 | 6.31E-15 | 3.05E-14 |
| gene-BPHYT_RS15945 | 5099.09114 | 3578.04972 | 2083.25694 | 439.741363 | 337.661501 | 521.496509 | 3.04974874 | 6.59E-15 | 3.18E-14 |
| gene-BPHYT_RS17480 | 2883.5524 | 3320.95074 | 2863.08031 | 5164.4961 | 5811.26657 | 5538.18428 | -0.86502356 | 6.95E-15 | 3.35E-14 |
| gene-BPHYT_RS17390 | 2686.42053 | 3319.88394 | 3130.47737 | 5381.40888 | 6065.44718 | 5735.55622 | -0.91152635 | 7.27E-15 | 3.50E-14 |
| gene-BPHYT_RS31050 | 0 | 0 | 0 | 102.540587 | 135.81219 | 123.13112 | -9.42293071 | 7.51E-15 | 3.62E-14 |
| gene-BPHYT_RS18785 | 1039.13236 | 886.511423 | 941.481664 | 410.162348 | 495.901393 | 351.285843 | 1.19382754 | 7.66E-15 | 3.68E-14 |
| gene-BPHYT_RS34910 | 1733.48348 | 1634.33875 | 1511.86094 | 773.984238 | 923.273698 | 760.515742 | 0.99167662 | 7.66E-15 | 3.68E-14 |
| gene-BPHYT_RS17120 | 10326.6771 | 12910.4227 | 11768.5208 | 22166.5142 | 21697.5549 | 20441.5767 | -0.87747818 | 8.41E-15 | 4.04E-14 |
| gene-BPHYT_RS01900 | 396.658052 | 370.17986 | 355.851601 | 149.867012 | 80.9889209 | 128.563375 | 1.63388938 | 8.60E-15 | 4.12E-14 |
| gene-BPHYT_RS34210 | 10273.2041 | 10320.2305 | 10930.7445 | 5526.34606 | 6295.95411 | 6281.49788 | 0.80022416 | 8.67E-15 | 4.15E-14 |
| gene-BPHYT_RS27440 | 10333.062 | 8905.65266 | 3142.67799 | 508.759066 | 539.510812 | 532.361019 | 3.82378024 | 9.36E-15 | 4.48E-14 |
| gene-BPHYT_RS12150 | 7829.40743 | 9803.8989 | 10223.1081 | 4570.94386 | 5048.72473 | 4627.37614 | 0.96734015 | 9.38E-15 | 4.49E-14 |
| gene-BPHYT_RS06685 | 7273.92654 | 7051.55296 | 7201.41968 | 11932.1748 | 12104.7287 | 11386.9125 | -0.71847551 | 9.39E-15 | 4.49E-14 |
| gene-BPHYT_RS24350 | 150.841795 | 169.621319 | 160.64158 | 351.990284 | 408.682555 | 415.56753 | -1.29160473 | 9.60E-15 | 4.58E-14 |
| gene-BPHYT_RS09525 | 174.784937 | 163.220515 | 166.741893 | 408.190414 | 421.142389 | 369.39336 | -1.24501892 | 9.77E-15 | 4.66E-14 |
| gene-BPHYT_RS19890 | 189.150822 | 213.36015 | 155.557986 | 31.5509498 | 59.8072031 | 39.8365389 | 2.10600249 | 1.09E-14 | 5.19E-14 |
| gene-BPHYT_RS22080 | 2022.39739 | 1861.56731 | 1183.46075 | 635.948833 | 677.814969 | 641.006125 | 1.37511082 | 1.09E-14 | 5.21E-14 |
| gene-BPHYT_RS01730 | 389.47511 | 419.252695 | 397.537074 | 762.152632 | 829.824943 | 797.636153 | -0.98654393 | 1.18E-14 | 5.63E-14 |
| gene-BPHYT_RS20305 | 234.642792 | 457.657522 | 295.865188 | 90.7089808 | 89.7108047 | 58.8494324 | 2.04927109 | 1.24E-14 | 5.89E-14 |
| gene-BPHYT_RS03835 | 1107.76937 | 849.173397 | 900.812909 | 434.811527 | 416.158455 | 463.552452 | 1.12056135 | 1.30E-14 | 6.16E-14 |
| gene-BPHYT_RS26650 | 131.687281 | 132.283293 | 117.939388 | 341.144645 | 312.741833 | 313.260056 | -1.33996514 | 1.33E-14 | 6.31E-14 |
| gene-BPHYT_RS22325 | 272.153714 | 370.17986 | 247.062683 | 94.6528495 | 102.170639 | 77.8623259 | 1.6990694 | 1.35E-14 | 6.42E-14 |
| gene-BPHYT_RS29745 | 4712.80845 | 5282.79732 | 5602.12092 | 2744.93264 | 3045.18343 | 2803.04373 | 0.86018762 | 1.38E-14 | 6.52E-14 |
| gene-BPHYT_RS15475 | 589.799398 | 544.068383 | 664.934134 | 1101.32534 | 1309.52855 | 1274.76924 | -1.03438647 | 1.44E-14 | 6.81E-14 |
| gene-BPHYT_RS07580 | 323.232417 | 333.908635 | 427.021921 | 139.021373 | 130.828257 | 134.901007 | 1.41929102 | 1.44E-14 | 6.81E-14 |
| gene-BPHYT_RS12305 | 5884.4262 | 4096.51488 | 2111.72507 | 428.895724 | 424.880339 | 458.120197 | 3.20440341 | 1.45E-14 | 6.83E-14 |
| gene-BPHYT_RS17125 | 14213.4472 | 17500.8663 | 17899.3355 | 30677.3829 | 32113.9761 | 29621.1828 | -0.89742871 | 1.50E-14 | 7.06E-14 |
| gene-BPHYT_RS04285 | 415.812566 | 472.592732 | 555.128497 | 989.911051 | 1046.62606 | 1007.68336 | -1.07816656 | 1.54E-14 | 7.26E-14 |
| gene-BPHYT_RS35210 | 39.9052367 | 46.939233 | 33.5517224 | 156.768782 | 147.026041 | 159.346155 | -1.94687663 | 1.66E-14 | 7.83E-14 |
| gene-BPHYT_RS03035 | 713.505632 | 548.335586 | 663.917415 | 240.575993 | 311.49585 | 243.546113 | 1.28089829 | 1.74E-14 | 8.20E-14 |
| gene-BPHYT_RS12670 | 939.369271 | 1086.00316 | 1045.18699 | 1874.32361 | 1824.1197 | 1888.61409 | -0.86483434 | 1.87E-14 | 8.79E-14 |
| gene-BPHYT_RS20640 | 1542.73645 | 1454.04942 | 1652.16815 | 881.454661 | 813.627159 | 776.812508 | 0.91143755 | 1.96E-14 | 9.20E-14 |
| gene-BPHYT_RS35760 | 188.352717 | 102.412872 | 135.223608 | 413.120249 | 480.949592 | 414.662154 | -1.60963596 | 1.99E-14 | 9.36E-14 |
| gene-BPHYT_RS02680 | 402.244786 | 497.12915 | 392.45348 | 174.516191 | 175.683659 | 178.359049 | 1.28772865 | 2.06E-14 | 9.66E-14 |
| gene-BPHYT_RS22830 | 7.1829426 | 4.267203 | 7.11703202 | 60.1439981 | 74.7590039 | 85.105333 | -3.54989914 | 2.09E-14 | 9.81E-14 |
| gene-BPHYT_RS10095 | 350.367978 | 263.499785 | 397.537074 | 94.6528495 | 127.090307 | 71.5246948 | 1.79797814 | 2.10E-14 | 9.81E-14 |
| gene-BPHYT_RS13480 | 51.0787029 | 39.4716278 | 61.0031316 | 188.319732 | 216.801111 | 162.062283 | -1.89830301 | 2.27E-14 | 1.06E-13 |
| gene-BPHYT_RS01560 | 3247.48816 | 3920.49276 | 3951.98621 | 6570.4853 | 6942.6195 | 6738.7127 | -0.86525947 | 2.28E-14 | 1.07E-13 |
| gene-BPHYT_RS05565 | 310.462741 | 213.36015 | 229.778462 | 596.510145 | 604.301948 | 777.717884 | -1.38915847 | 2.39E-14 | 1.12E-13 |
| gene-BPHYT_RS17030 | 8104.75357 | 7974.33561 | 6616.80634 | 13181.3953 | 13895.2069 | 14172.7541 | -0.86185033 | 2.41E-14 | 1.12E-13 |
| gene-BPHYT_RS25805 | 411.822042 | 448.056315 | 477.857864 | 117.330095 | 142.042107 | 200.993446 | 1.53381804 | 2.45E-14 | 1.14E-13 |
| gene-BPHYT_RS17495 | 5447.86291 | 5283.86412 | 5120.19618 | 8479.31777 | 8713.16191 | 8832.84711 | -0.71516443 | 2.63E-14 | 1.22E-13 |
| gene-BPHYT_RS16170 | 12175.8858 | 13349.9446 | 12269.7632 | 20969.55 | 20809.1687 | 20529.3982 | -0.72125782 | 2.65E-14 | 1.23E-13 |
| gene-BPHYT_RS11405 | 1743.06074 | 2023.72102 | 1636.91736 | 3184.674 | 3467.5718 | 3414.17246 | -0.89777533 | 2.72E-14 | 1.26E-13 |
| gene-BPHYT_RS12650 | 296.096856 | 417.119093 | 380.252853 | 138.035406 | 124.59834 | 136.711758 | 1.44817054 | 2.80E-14 | 1.30E-13 |
| gene-BPHYT_RS18360 | 310.462741 | 313.639421 | 435.155672 | 136.063471 | 107.154572 | 119.509617 | 1.54165836 | 2.82E-14 | 1.31E-13 |
| gene-BPHYT_RS13455 | 5.58673313 | 3.20040225 | 4.06687544 | 80.8493089 | 66.0371201 | 57.0386806 | -3.95697523 | 2.96E-14 | 1.37E-13 |
| gene-BPHYT_RS29945 | 476.468526 | 538.734379 | 474.807707 | 214.940846 | 237.982829 | 218.195588 | 1.15201571 | 2.98E-14 | 1.38E-13 |
| gene-BPHYT_RS14520 | 555.480894 | 365.912657 | 434.138953 | 1012.5883 | 1127.61498 | 1021.264 | -1.21787314 | 3.08E-14 | 1.43E-13 |
| gene-BPHYT_RS01440 | 10998.6813 | 11660.1322 | 10569.8093 | 18145.74 | 18222.5072 | 18207.109 | -0.71584983 | 3.18E-14 | 1.47E-13 |
| gene-BPHYT_RS19065 | 120.513815 | 116.281282 | 73.2037579 | 328.327072 | 378.778953 | 281.5719 | -1.66708626 | 3.26E-14 | 1.51E-13 |
| gene-BPHYT_RS06710 | 3592.2694 | 2788.61716 | 2173.74492 | 1230.48704 | 1191.16013 | 1212.29831 | 1.23559795 | 3.27E-14 | 1.51E-13 |
| gene-BPHYT_RS24515 | 934.580643 | 679.552078 | 469.724113 | 225.786485 | 240.474796 | 190.128935 | 1.67039445 | 3.50E-14 | 1.62E-13 |
| gene-BPHYT_RS35125 | 346.377454 | 304.038214 | 403.637387 | 124.231865 | 132.07424 | 143.04939 | 1.39931279 | 3.55E-14 | 1.64E-13 |
| gene-BPHYT_RS15575 | 2158.0752 | 2392.83408 | 2612.96747 | 4581.7895 | 4250.04937 | 4171.97207 | -0.86060267 | 3.58E-14 | 1.65E-13 |
| gene-BPHYT_RS01725 | 1717.52139 | 1537.25988 | 1418.32281 | 2885.92594 | 2762.34519 | 3291.94671 | -0.93569215 | 3.62E-14 | 1.67E-13 |
| gene-BPHYT_RS21435 | 244.220048 | 252.831778 | 222.66143 | 72.9615715 | 72.2670371 | 94.1590918 | 1.58170903 | 3.68E-14 | 1.69E-13 |
| gene-BPHYT_RS04385 | 2290.56058 | 2306.42322 | 2170.69477 | 3961.61614 | 3913.63385 | 3646.85406 | -0.76745272 | 3.73E-14 | 1.71E-13 |
| gene-BPHYT_RS04635 | 514.777553 | 507.797157 | 328.400192 | 4.92983591 | 14.9518008 | 28.9720283 | 4.78159084 | 3.77E-14 | 1.73E-13 |
| gene-BPHYT_RS29395 | 276.942342 | 266.700188 | 285.698 | 556.085491 | 561.938513 | 618.371728 | -1.06662397 | 3.81E-14 | 1.75E-13 |
| gene-BPHYT_RS10965 | 114.927082 | 101.346071 | 50.835943 | 4.92983591 | 7.47590039 | 9.05375883 | 3.6370623 | 3.93E-14 | 1.80E-13 |
| gene-BPHYT_RS38670 | 248.210572 | 234.696165 | 152.507829 | 50.2843263 | 58.5612197 | 26.2559006 | 2.25134322 | 4.09E-14 | 1.87E-13 |
| gene-BPHYT_RS03475 | 210.69965 | 199.49174 | 307.049096 | 595.524178 | 671.585052 | 580.345941 | -1.36552916 | 4.13E-14 | 1.89E-13 |
| gene-BPHYT_RS21975 | 2838.06043 | 2612.59504 | 2135.1096 | 1142.73596 | 1318.25044 | 1288.34988 | 1.01736816 | 4.21E-14 | 1.92E-13 |
| gene-BPHYT_RS20555 | 391.071319 | 347.777045 | 334.500505 | 757.222796 | 779.985607 | 685.369543 | -1.04710134 | 4.32E-14 | 1.97E-13 |
| gene-BPHYT_RS06135 | 966.504832 | 1012.39391 | 1036.03652 | 518.618738 | 564.430479 | 501.578239 | 0.92930101 | 4.46E-14 | 2.03E-13 |
| gene-BPHYT_RS28780 | 1996.85804 | 1750.62003 | 1554.56314 | 3271.43911 | 3384.09091 | 3301.90585 | -0.90840309 | 4.64E-14 | 2.12E-13 |
| gene-BPHYT_RS04865 | 219.478802 | 208.026146 | 255.196434 | 537.352114 | 469.735741 | 625.614735 | -1.26050939 | 4.76E-14 | 2.17E-13 |
| gene-BPHYT_RS24030 | 237.835211 | 250.698176 | 225.711587 | 85.7791449 | 79.7429375 | 87.8214607 | 1.49207671 | 4.86E-14 | 2.21E-13 |
| gene-BPHYT_RS34095 | 1033.54563 | 872.643014 | 629.348974 | 1941.36938 | 1947.47205 | 2043.43337 | -1.22498771 | 4.93E-14 | 2.24E-13 |
| gene-BPHYT_RS33440 | 158.822842 | 121.615286 | 185.042832 | 375.653496 | 461.013857 | 410.135275 | -1.41824671 | 5.09E-14 | 2.31E-13 |
| gene-BPHYT_RS35220 | 146.053166 | 122.682086 | 120.989544 | 318.4674 | 356.351252 | 316.881559 | -1.34128685 | 5.12E-14 | 2.33E-13 |
| gene-BPHYT_RS34515 | 28.7317704 | 27.7368195 | 22.3678149 | 102.540587 | 240.474796 | 134.901007 | -2.59026026 | 5.18E-14 | 2.35E-13 |
| gene-BPHYT_RS00150 | 1803.7167 | 2254.14999 | 2600.76684 | 4333.32577 | 4565.28317 | 5021.21465 | -1.06454615 | 5.26E-14 | 2.38E-13 |
| gene-BPHYT_RS09870 | 11.1734663 | 10.6680075 | 15.2507829 | 98.5967182 | 79.7429375 | 81.4838295 | -2.81431033 | 5.35E-14 | 2.42E-13 |
| gene-BPHYT_RS07085 | 271.355609 | 277.368195 | 355.851601 | 629.047062 | 676.568985 | 737.881345 | -1.177676 | 5.35E-14 | 2.42E-13 |
| gene-BPHYT_RS12865 | 1566.67959 | 1801.82647 | 2018.18694 | 3238.90219 | 3483.76958 | 3458.53587 | -0.91906075 | 5.49E-14 | 2.48E-13 |
| gene-BPHYT_RS16615 | 193.141345 | 215.493752 | 253.162996 | 490.02569 | 490.917459 | 540.509402 | -1.20470763 | 5.50E-14 | 2.48E-13 |
| gene-BPHYT_RS06140 | 114.128977 | 117.348083 | 100.655167 | 23.6632124 | 23.6736846 | 18.1075177 | 2.35253552 | 5.53E-14 | 2.50E-13 |
| gene-BPHYT_RS13915 | 779.748324 | 685.952882 | 876.411657 | 309.593695 | 371.303053 | 379.352495 | 1.14415295 | 5.75E-14 | 2.59E-13 |
| gene-BPHYT_RS35235 | 80.6085781 | 88.5444623 | 99.6384482 | 223.81455 | 265.394464 | 294.247162 | -1.54730962 | 5.75E-14 | 2.59E-13 |
| gene-BPHYT_RS14435 | 3043.97145 | 3777.54146 | 3462.94444 | 6176.09843 | 6026.8217 | 6272.44412 | -0.84565002 | 6.08E-14 | 2.74E-13 |
| gene-BPHYT_RS21890 | 3657.71399 | 3462.83524 | 3134.54424 | 1959.11679 | 1959.93189 | 1943.84202 | 0.80694297 | 6.22E-14 | 2.80E-13 |
| gene-BPHYT_RS17630 | 7756.7799 | 8545.07401 | 6552.75305 | 14013.5516 | 14513.2146 | 13827.8059 | -0.89001575 | 6.27E-14 | 2.82E-13 |
| gene-BPHYT_RS09705 | 352.762292 | 325.374229 | 300.948782 | 110.428324 | 138.304157 | 132.184879 | 1.36588119 | 6.70E-14 | 3.01E-13 |
| gene-BPHYT_RS01490 | 1585.83411 | 1727.15042 | 1624.71674 | 2789.30116 | 2853.30198 | 2760.49107 | -0.76734295 | 6.73E-14 | 3.02E-13 |
| gene-BPHYT_RS18195 | 4735.95349 | 5042.76715 | 6100.31316 | 10550.8348 | 10258.1813 | 9305.45333 | -0.92345403 | 6.77E-14 | 3.03E-13 |
| gene-BPHYT_RS37730 | 2067.88936 | 2621.12944 | 2181.87867 | 1244.29058 | 1009.24655 | 1153.44888 | 1.0105923 | 7.30E-14 | 3.27E-13 |
| gene-BPHYT_RS38720 | 155.630423 | 130.149692 | 130.140014 | 30.5649827 | 32.3955684 | 38.0257871 | 2.04020309 | 7.40E-14 | 3.31E-13 |
| gene-BPHYT_RS15130 | 245.816258 | 232.562564 | 268.413779 | 510.731 | 524.559011 | 556.806168 | -1.09254585 | 7.75E-14 | 3.47E-13 |
| gene-BPHYT_RS09045 | 7198.10659 | 8423.45872 | 9191.13849 | 15466.8672 | 15105.0567 | 14588.3216 | -0.86413948 | 8.12E-14 | 3.63E-13 |
| gene-BPHYT_RS15290 | 80.6085781 | 61.8744435 | 98.6217294 | 3.94386873 | 7.47590039 | 10.8645106 | 3.42440875 | 8.98E-14 | 4.01E-13 |
| gene-BPHYT_RS17760 | 1436.58852 | 1707.948 | 1499.66032 | 852.861613 | 834.808877 | 760.515742 | 0.92370307 | 9.09E-14 | 4.06E-13 |
| gene-BPHYT_RS18790 | 1996.85804 | 1699.4136 | 1653.18487 | 795.675516 | 1008.00057 | 772.285628 | 1.05682644 | 9.57E-14 | 4.27E-13 |
| gene-BPHYT_RS35005 | 9.5772568 | 2.1336015 | 10.1671886 | 80.8493089 | 90.9567881 | 64.2816877 | -3.39601114 | 1.04E-13 | 4.63E-13 |
| gene-BPHYT_RS12100 | 6815.01632 | 8212.23218 | 9538.85634 | 16335.5043 | 16140.4689 | 15459.2932 | -0.96458345 | 1.04E-13 | 4.64E-13 |
| gene-BPHYT_RS30880 | 123.706234 | 115.214481 | 127.089857 | 20.7053108 | 33.6415518 | 12.6752624 | 2.48529366 | 1.11E-13 | 4.91E-13 |
| gene-BPHYT_RS06545 | 9091.21102 | 11508.6465 | 11555.0098 | 22279.9004 | 19493.4103 | 19420.3127 | -0.92850697 | 1.14E-13 | 5.05E-13 |
| gene-BPHYT_RS21490 | 550.692266 | 452.323518 | 549.028184 | 226.772452 | 247.950696 | 216.384836 | 1.17097107 | 1.14E-13 | 5.07E-13 |
| gene-BPHYT_RS03765 | 1288.14104 | 1324.96653 | 1171.26013 | 2097.1522 | 2366.12247 | 2387.4762 | -0.85599889 | 1.15E-13 | 5.11E-13 |
| gene-BPHYT_RS06660 | 5348.09982 | 5393.74459 | 5501.46575 | 8985.11893 | 9169.19183 | 8406.41507 | -0.70937209 | 1.20E-13 | 5.31E-13 |
| gene-BPHYT_RS06075 | 740.641192 | 786.232153 | 631.382412 | 338.186744 | 363.827152 | 318.692311 | 1.08238333 | 1.20E-13 | 5.32E-13 |
| gene-BPHYT_RS00165 | 178.77546 | 133.350094 | 100.655167 | 398.330742 | 382.516903 | 407.419147 | -1.51855932 | 1.22E-13 | 5.40E-13 |
| gene-BPHYT_RS14900 | 706.322689 | 962.254277 | 855.060561 | 1614.02828 | 1704.50529 | 1967.38179 | -1.06857495 | 1.24E-13 | 5.49E-13 |
| gene-BPHYT_RS13980 | 10059.3121 | 11231.2783 | 12216.8938 | 19648.354 | 19194.3743 | 19084.4182 | -0.7898733 | 1.40E-13 | 6.19E-13 |
| gene-BPHYT_RS23550 | 286.519599 | 205.892545 | 211.477523 | 53.2422278 | 84.7268711 | 67.9031912 | 1.78548297 | 1.41E-13 | 6.22E-13 |
| gene-BPHYT_RS20215 | 1172.41585 | 428.853902 | 1292.24967 | 11203.5451 | 9931.73367 | 10191.8163 | -3.43627847 | 1.51E-13 | 6.65E-13 |
| gene-BPHYT_RS02295 | 2518.02043 | 2668.06868 | 2383.18901 | 4235.71502 | 4215.16184 | 4219.05162 | -0.74327253 | 1.57E-13 | 6.92E-13 |
| gene-BPHYT_RS18065 | 13941.2935 | 17544.6051 | 21887.9236 | 38573.0081 | 37930.2266 | 36986.4156 | -1.08843485 | 1.65E-13 | 7.25E-13 |
| gene-BPHYT_RS29615 | 92.5801491 | 94.9452668 | 104.722043 | 12.8175734 | 22.4277012 | 15.39139 | 2.55056795 | 1.82E-13 | 8.00E-13 |
| gene-BPHYT_RS16205 | 508.392715 | 375.513864 | 292.815032 | 1052.02698 | 931.995582 | 985.048961 | -1.33215821 | 1.91E-13 | 8.39E-13 |
| gene-BPHYT_RS21245 | 909.839396 | 907.847438 | 713.736639 | 1531.20703 | 1812.90585 | 1752.80771 | -1.00880649 | 1.94E-13 | 8.54E-13 |
| gene-BPHYT_RS34230 | 233.844687 | 249.631376 | 216.561117 | 469.320379 | 500.885326 | 561.333047 | -1.13056989 | 2.13E-13 | 9.35E-13 |
| gene-BPHYT_RS12340 | 397.456157 | 366.979458 | 385.336448 | 182.403929 | 163.223825 | 149.387021 | 1.21724103 | 2.24E-13 | 9.83E-13 |
| gene-BPHYT_RS17415 | 450.13107 | 404.317484 | 326.366754 | 835.114203 | 842.284777 | 824.797429 | -1.08099558 | 2.50E-13 | 1.10E-12 |
| gene-BPHYT_RS24565 | 182.765984 | 119.481684 | 169.79205 | 33.5228842 | 27.4116348 | 46.17417 | 2.12765878 | 2.79E-13 | 1.22E-12 |
| gene-BPHYT_RS25815 | 529.143438 | 600.608822 | 623.248661 | 216.91278 | 282.838231 | 278.855772 | 1.17135286 | 2.86E-13 | 1.25E-12 |
| gene-BPHYT_RS30080 | 1441.37715 | 1483.91984 | 1249.54748 | 683.275257 | 718.932421 | 789.48777 | 0.92923065 | 3.08E-13 | 1.35E-12 |
| gene-BPHYT_RS34935 | 381.494063 | 279.501797 | 231.8119 | 93.6668823 | 103.416622 | 91.4429642 | 1.63576147 | 3.12E-13 | 1.36E-12 |
| gene-BPHYT_RS01845 | 436.563289 | 458.724323 | 384.319729 | 174.516191 | 169.453742 | 205.520325 | 1.21613373 | 3.15E-13 | 1.38E-12 |
| gene-BPHYT_RS01595 | 272.951819 | 304.038214 | 350.768007 | 687.219126 | 630.4676 | 635.57387 | -1.07675899 | 3.43E-13 | 1.49E-12 |
| gene-BPHYT_RS12095 | 2005.6372 | 2424.83811 | 2512.3123 | 4143.0341 | 4463.11253 | 4092.29899 | -0.87151022 | 3.51E-13 | 1.53E-12 |
| gene-BPHYT_RS19590 | 403.840995 | 513.131161 | 466.673956 | 896.244169 | 902.09198 | 937.969415 | -0.98648559 | 3.68E-13 | 1.60E-12 |
| gene-BPHYT_RS13660 | 1291.33346 | 1258.82489 | 1324.78467 | 2157.2962 | 2322.51306 | 2131.25483 | -0.77019956 | 3.92E-13 | 1.71E-12 |
| gene-BPHYT_RS15710 | 82228.7307 | 79647.344 | 67300.6882 | 41291.3196 | 45726.3447 | 43478.866 | 0.81247568 | 4.03E-13 | 1.75E-12 |
| gene-BPHYT_RS15285 | 1076.64329 | 1186.28243 | 1202.77841 | 589.608375 | 671.585052 | 567.670679 | 0.92365535 | 4.19E-13 | 1.82E-12 |
| gene-BPHYT_RS17095 | 5845.31907 | 6788.05317 | 6314.84084 | 10424.631 | 10820.1198 | 10396.4313 | -0.73989645 | 4.25E-13 | 1.85E-12 |
| gene-BPHYT_RS30955 | 526.749124 | 605.942826 | 491.075209 | 1102.31131 | 1173.71636 | 972.373698 | -1.00012902 | 4.61E-13 | 2.00E-12 |
| gene-BPHYT_RS22365 | 1005.61196 | 1338.83494 | 1079.75543 | 560.02936 | 581.874247 | 486.186849 | 1.07309355 | 4.86E-13 | 2.10E-12 |
| gene-BPHYT_RS07950 | 96.5706727 | 109.880477 | 102.688605 | 10.845639 | 16.1977842 | 25.3505247 | 2.5459144 | 5.16E-13 | 2.24E-12 |
| gene-BPHYT_RS33770 | 2593.84038 | 2198.67635 | 1862.62895 | 1155.55354 | 941.963449 | 1106.36933 | 1.05422709 | 5.26E-13 | 2.28E-12 |
| gene-BPHYT_RS23335 | 8.77915207 | 9.60120675 | 3.05015658 | 95.6388167 | 107.154572 | 47.0795459 | -3.52703932 | 5.39E-13 | 2.33E-12 |
| gene-BPHYT_RS07755 | 1233.86992 | 1108.40598 | 1275.98217 | 2134.61895 | 2381.07427 | 2039.81186 | -0.8564321 | 5.52E-13 | 2.38E-12 |
| gene-BPHYT_RS17280 | 3059.93355 | 3011.57852 | 3070.49096 | 5314.36311 | 4863.0732 | 4841.95022 | -0.71627558 | 5.59E-13 | 2.41E-12 |
| gene-BPHYT_RS02605 | 4573.93823 | 5473.75465 | 5582.80326 | 9289.78279 | 9605.28602 | 8745.93103 | -0.82264883 | 5.59E-13 | 2.41E-12 |
| gene-BPHYT_RS14795 | 2027.18602 | 1910.64014 | 1603.36564 | 3333.55504 | 3493.73745 | 3268.40694 | -0.86481262 | 5.64E-13 | 2.43E-12 |
| gene-BPHYT_RS22555 | 7751.99127 | 7631.89257 | 7370.19501 | 11877.9466 | 12336.4816 | 11873.0993 | -0.66527861 | 5.93E-13 | 2.55E-12 |
| gene-BPHYT_RS13630 | 8379.30159 | 9968.18621 | 10635.896 | 18451.3899 | 16956.5881 | 16702.3743 | -0.84650487 | 6.55E-13 | 2.82E-12 |
| gene-BPHYT_RS18610 | 6505.35168 | 6507.48458 | 5810.54828 | 9918.82985 | 10624.5004 | 10829.2009 | -0.73690561 | 6.64E-13 | 2.86E-12 |
| gene-BPHYT_RS24020 | 500.411668 | 482.193939 | 370.085665 | 185.36183 | 148.272024 | 200.993446 | 1.33485777 | 6.74E-13 | 2.89E-12 |
| gene-BPHYT_RS14810 | 185.958403 | 347.777045 | 555.128497 | 2795.21696 | 3202.17733 | 3154.32958 | -3.07225273 | 6.79E-13 | 2.91E-12 |
| gene-BPHYT_RS14925 | 9118.34658 | 11397.6992 | 12641.8823 | 20218.243 | 21530.5931 | 21236.4967 | -0.92579586 | 6.81E-13 | 2.92E-12 |
| gene-BPHYT_RS08930 | 1359.17236 | 1438.04741 | 1341.05218 | 2243.07534 | 2361.13854 | 2350.35579 | -0.74906851 | 6.86E-13 | 2.94E-12 |
| gene-BPHYT_RS02970 | 2522.80906 | 3411.6288 | 3264.68426 | 6263.84951 | 6279.75633 | 5437.68755 | -0.96733596 | 7.37E-13 | 3.15E-12 |
| gene-BPHYT_RS01600 | 168.400099 | 148.285304 | 178.942519 | 403.260578 | 342.645435 | 408.324523 | -1.22052937 | 7.60E-13 | 3.25E-12 |
| gene-BPHYT_RS24665 | 113.330872 | 109.880477 | 105.738761 | 17.7474093 | 31.149585 | 19.9182694 | 2.2806887 | 7.69E-13 | 3.29E-12 |
| gene-BPHYT_RS01570 | 2560.31998 | 2831.28919 | 2654.65294 | 4344.17141 | 4914.15852 | 4419.13969 | -0.76539345 | 8.09E-13 | 3.46E-12 |
| gene-BPHYT_RS04955 | 5114.25513 | 5501.49147 | 5623.47201 | 8894.40995 | 9109.38463 | 8475.22364 | -0.70544055 | 8.23E-13 | 3.51E-12 |
| gene-BPHYT_RS15100 | 1442.97336 | 1597.00072 | 1752.82331 | 840.044039 | 863.466495 | 906.281259 | 0.87605693 | 8.27E-13 | 3.53E-12 |
| gene-BPHYT_RS12960 | 1183.58932 | 1123.34119 | 1247.51404 | 679.331389 | 660.371201 | 646.43838 | 0.83984749 | 8.43E-13 | 3.59E-12 |
| gene-BPHYT_RS35470 | 485.247678 | 456.590721 | 473.790988 | 238.604058 | 210.571194 | 226.343971 | 1.06586516 | 8.73E-13 | 3.72E-12 |
| gene-BPHYT_RS29400 | 189.948927 | 231.495763 | 195.210021 | 415.092184 | 479.703608 | 476.227714 | -1.15487553 | 8.83E-13 | 3.76E-12 |
| gene-BPHYT_RS09280 | 2261.82881 | 2189.07514 | 1966.33427 | 1119.07275 | 1283.3629 | 1197.81229 | 0.83484053 | 9.16E-13 | 3.90E-12 |
| gene-BPHYT_RS20600 | 213.892069 | 107.746876 | 173.858925 | 485.095854 | 448.554023 | 496.145984 | -1.52256836 | 9.97E-13 | 4.23E-12 |
| gene-BPHYT_RS19460 | 802.095257 | 851.306999 | 931.314475 | 1482.89464 | 1604.82662 | 1525.55836 | -0.83621308 | 1.02E-12 | 4.31E-12 |
| gene-BPHYT_RS03685 | 9467.11835 | 10211.4168 | 10207.8574 | 15286.4352 | 16460.6867 | 16737.684 | -0.6981202 | 1.02E-12 | 4.31E-12 |
| gene-BPHYT_RS34685 | 2727.92198 | 3514.04167 | 3301.28614 | 5666.3534 | 5823.7264 | 5708.39494 | -0.85022594 | 1.03E-12 | 4.37E-12 |
| gene-BPHYT_RS26420 | 106.946034 | 140.817699 | 115.90595 | 14.7895077 | 34.8875352 | 22.6343971 | 2.35234264 | 1.06E-12 | 4.47E-12 |
| gene-BPHYT_RS01685 | 2023.9936 | 2176.27353 | 1900.24755 | 3304.962 | 3607.12194 | 3453.10362 | -0.76467716 | 1.13E-12 | 4.79E-12 |
| gene-BPHYT_RS13565 | 598.57855 | 525.93277 | 391.436761 | 56.2001294 | 13.7058174 | 30.78278 | 3.90358584 | 1.15E-12 | 4.85E-12 |
| gene-BPHYT_RS36895 | 12357.8537 | 11614.2598 | 12379.5688 | 7710.26337 | 7688.96355 | 7051.97275 | 0.69537582 | 1.20E-12 | 5.05E-12 |
| gene-BPHYT_RS20290 | 66.2426929 | 50.1396353 | 62.0198504 | 196.207469 | 160.731858 | 210.952581 | -1.66805979 | 1.21E-12 | 5.09E-12 |
| gene-BPHYT_RS02575 | 2621.77405 | 2783.28316 | 2577.38231 | 4251.49049 | 4291.16682 | 4393.78916 | -0.696727 | 1.32E-12 | 5.59E-12 |
| gene-BPHYT_RS16610 | 135.677805 | 135.483695 | 139.290484 | 314.523531 | 333.923551 | 301.490169 | -1.20941831 | 1.33E-12 | 5.62E-12 |
| gene-BPHYT_RS17845 | 213.093964 | 308.305417 | 229.778462 | 548.197753 | 585.612197 | 574.913686 | -1.1894322 | 1.38E-12 | 5.81E-12 |
| gene-BPHYT_RS17540 | 1210.72488 | 1388.97458 | 1387.82124 | 2430.4091 | 2190.43881 | 2374.80094 | -0.81216907 | 1.40E-12 | 5.88E-12 |
| gene-BPHYT_RS13970 | 157.226632 | 145.084902 | 175.892363 | 26.6211139 | 57.3152363 | 33.4989077 | 2.04838642 | 1.41E-12 | 5.94E-12 |
| gene-BPHYT_RS29495 | 15.9620947 | 21.336015 | 18.3009395 | 98.5967182 | 117.122439 | 78.7677018 | -2.4097284 | 1.61E-12 | 6.77E-12 |
| gene-BPHYT_RS16225 | 393.465634 | 436.321507 | 351.784725 | 793.703582 | 750.082006 | 762.326494 | -0.96549687 | 1.76E-12 | 7.39E-12 |
| gene-BPHYT_RS10285 | 156.428528 | 172.821722 | 135.223608 | 355.934153 | 350.121335 | 360.339601 | -1.19986604 | 1.76E-12 | 7.40E-12 |
| gene-BPHYT_RS07575 | 639.281891 | 493.928747 | 573.429437 | 279.028713 | 259.164547 | 261.65363 | 1.09458308 | 1.78E-12 | 7.48E-12 |
| gene-BPHYT_RS17275 | 5063.17643 | 4483.76355 | 4085.17638 | 7783.22494 | 7980.52367 | 7750.01756 | -0.78615567 | 1.84E-12 | 7.69E-12 |
| gene-BPHYT_RS03205 | 825.240294 | 742.493322 | 756.438831 | 1333.02763 | 1336.94019 | 1473.04656 | -0.83345682 | 1.85E-12 | 7.73E-12 |
| gene-BPHYT_RS13440 | 83.0028923 | 130.149692 | 43.718911 | 0 | 0 | 0 | 8.81008843 | 1.87E-12 | 7.82E-12 |
| gene-BPHYT_RS12200 | 1320.86333 | 1548.99469 | 1518.97798 | 2620.70077 | 2538.06818 | 2452.66327 | -0.79512928 | 1.94E-12 | 8.12E-12 |
| gene-BPHYT_RS12195 | 988.851764 | 1135.076 | 1129.57465 | 1876.29555 | 1877.69698 | 1878.65496 | -0.79285834 | 1.98E-12 | 8.25E-12 |
| gene-BPHYT_RS24250 | 62.2521692 | 58.6740413 | 73.2037579 | 193.249568 | 181.913576 | 190.128935 | -1.5435884 | 2.01E-12 | 8.37E-12 |
| gene-BPHYT_RS12445 | 14843.1518 | 16179.1002 | 14973.2186 | 23906.7463 | 25545.1516 | 24060.3641 | -0.67650206 | 2.07E-12 | 8.63E-12 |
| gene-BPHYT_RS33825 | 3192.41893 | 2767.28115 | 2589.58294 | 5461.27222 | 4683.65159 | 5224.92422 | -0.84599715 | 2.16E-12 | 8.99E-12 |
| gene-BPHYT_RS10355 | 275.346133 | 254.965379 | 178.942519 | 78.8773746 | 80.9889209 | 72.4300706 | 1.61478399 | 2.16E-12 | 9.01E-12 |
| gene-BPHYT_RS03560 | 2349.62034 | 2321.35843 | 2212.38024 | 3583.00474 | 3979.67097 | 3776.32281 | -0.71974645 | 2.25E-12 | 9.35E-12 |
| gene-BPHYT_RS19510 | 5121.43807 | 5139.84602 | 3779.144 | 2189.83311 | 2432.15959 | 2525.99871 | 0.97410165 | 2.26E-12 | 9.38E-12 |
| gene-BPHYT_RS04745 | 2684.02622 | 3818.07989 | 4219.38327 | 7253.76056 | 7696.43945 | 7513.71445 | -1.06754308 | 2.27E-12 | 9.41E-12 |
| gene-BPHYT_RS04910 | 1261.00548 | 1216.15286 | 859.127436 | 535.38018 | 484.687542 | 508.821246 | 1.12574397 | 2.31E-12 | 9.57E-12 |
| gene-BPHYT_RS16235 | 1557.90044 | 1159.61242 | 836.759621 | 2679.8588 | 3056.39728 | 2878.18993 | -1.27590505 | 2.32E-12 | 9.62E-12 |
| gene-BPHYT_RS20975 | 158.024737 | 139.750898 | 61.0031316 | 12.8175734 | 9.96786719 | 22.6343971 | 2.95942656 | 2.33E-12 | 9.67E-12 |
| gene-BPHYT_RS06170 | 4567.55339 | 5093.97358 | 4943.28709 | 8145.07489 | 7670.2738 | 7854.13579 | -0.69686236 | 2.36E-12 | 9.75E-12 |
| gene-BPHYT_RS02735 | 410.225833 | 481.127138 | 448.373017 | 208.039076 | 156.993908 | 212.763333 | 1.20613088 | 2.40E-12 | 9.91E-12 |
| gene-BPHYT_RS04260 | 898.66593 | 1000.6591 | 1096.02293 | 1787.5585 | 1873.95903 | 1719.3088 | -0.84571561 | 2.47E-12 | 1.02E-11 |
| gene-BPHYT_RS21510 | 239.43142 | 161.086913 | 202.327053 | 69.0177028 | 58.5612197 | 62.4709359 | 1.66611755 | 2.68E-12 | 1.11E-11 |
| gene-BPHYT_RS12715 | 1707.94413 | 1730.35082 | 1799.59238 | 1060.90069 | 1032.92024 | 919.861897 | 0.79802958 | 2.88E-12 | 1.19E-11 |
| gene-BPHYT_RS03210 | 537.124485 | 489.661544 | 445.32286 | 901.174005 | 994.294752 | 900.849004 | -0.92321819 | 3.05E-12 | 1.26E-11 |
| gene-BPHYT_RS02565 | 1442.97336 | 1430.57981 | 1542.36251 | 2653.23769 | 2405.99394 | 2393.81384 | -0.75535017 | 3.12E-12 | 1.29E-11 |
| gene-BPHYT_RS29675 | 45.4919698 | 50.1396353 | 43.718911 | 142.965241 | 190.63546 | 142.144014 | -1.7684637 | 3.15E-12 | 1.30E-11 |
| gene-BPHYT_RS26840 | 3386.35838 | 3764.73985 | 3378.55677 | 5532.26186 | 5903.46934 | 5813.41855 | -0.71220483 | 3.22E-12 | 1.32E-11 |
| gene-BPHYT_RS08265 | 222.671221 | 245.364173 | 143.357359 | 36.4807857 | 67.2831035 | 53.4171771 | 1.96998499 | 3.22E-12 | 1.33E-11 |
| gene-BPHYT_RS04235 | 1298.5164 | 1476.45224 | 1593.19845 | 2595.06562 | 2737.42553 | 2439.98801 | -0.83176908 | 3.74E-12 | 1.54E-11 |
| gene-BPHYT_RS23915 | 888.290568 | 1001.7259 | 1004.51823 | 522.562607 | 534.526878 | 531.455643 | 0.8648829 | 3.77E-12 | 1.55E-11 |
| gene-BPHYT_RS23865 | 268.961295 | 217.627353 | 268.413779 | 106.484456 | 89.7108047 | 86.9160848 | 1.41585951 | 3.85E-12 | 1.58E-11 |
| gene-BPHYT_RS10365 | 1021.57406 | 941.985063 | 762.539145 | 407.204446 | 389.992804 | 487.092225 | 1.08457396 | 3.87E-12 | 1.59E-11 |
| gene-BPHYT_RS23850 | 420.601194 | 366.979458 | 345.684412 | 181.417962 | 132.07424 | 158.44078 | 1.2605637 | 3.88E-12 | 1.59E-11 |
| gene-BPHYT_RS20440 | 134.8797 | 105.613274 | 133.190171 | 317.481433 | 272.870364 | 344.042836 | -1.32127998 | 3.90E-12 | 1.60E-11 |
| gene-BPHYT_RS10785 | 22.3469325 | 46.939233 | 39.6520355 | 181.417962 | 115.876456 | 176.548297 | -2.14220147 | 4.04E-12 | 1.65E-11 |
| gene-BPHYT_RS06045 | 12987.5583 | 13545.1691 | 10602.3443 | 6565.55547 | 7232.93363 | 7060.12114 | 0.83222308 | 4.09E-12 | 1.67E-11 |
| gene-BPHYT_RS07585 | 401.446681 | 445.922714 | 418.88817 | 180.431994 | 220.539062 | 168.399914 | 1.15817974 | 4.11E-12 | 1.68E-11 |
| gene-BPHYT_RS03910 | 402.244786 | 360.578654 | 526.660369 | 191.277633 | 153.255958 | 172.021418 | 1.31675175 | 4.22E-12 | 1.72E-11 |
| gene-BPHYT_RS08810 | 1997.65615 | 2744.87833 | 2375.05526 | 4175.57102 | 4828.18567 | 4530.50092 | -0.92766919 | 4.25E-12 | 1.73E-11 |
| gene-BPHYT_RS35010 | 25.5393515 | 7.46760525 | 26.4346903 | 93.6668823 | 133.320224 | 126.752624 | -2.5524615 | 4.38E-12 | 1.78E-11 |
| gene-BPHYT_RS34890 | 90.9839396 | 77.8764548 | 69.1368824 | 214.940846 | 211.817178 | 222.722467 | -1.44307911 | 4.51E-12 | 1.84E-11 |
| gene-BPHYT_RS10975 | 3484.52527 | 2783.28316 | 367.035508 | 40.4246545 | 23.6736846 | 36.2150353 | 6.0454307 | 4.59E-12 | 1.87E-11 |
| gene-BPHYT_RS08220 | 422.197404 | 455.52392 | 484.974896 | 818.352761 | 848.514694 | 840.188819 | -0.88073859 | 4.66E-12 | 1.90E-11 |
| gene-BPHYT_RS34620 | 2952.98751 | 2581.65782 | 439.222547 | 7.88773746 | 14.9518008 | 38.0257871 | 6.61212893 | 4.69E-12 | 1.91E-11 |
| gene-BPHYT_RS12575 | 4375.21015 | 4791.00217 | 4849.74896 | 7460.81367 | 7490.85219 | 7439.47363 | -0.67607178 | 4.85E-12 | 1.97E-11 |
| gene-BPHYT_RS08850 | 476.468526 | 508.863958 | 495.142085 | 218.884715 | 269.132414 | 222.722467 | 1.06247886 | 4.91E-12 | 1.99E-11 |
| gene-BPHYT_RS15915 | 4521.26331 | 5237.99168 | 5074.44383 | 8115.49588 | 8147.48544 | 7986.32066 | -0.70931795 | 4.96E-12 | 2.01E-11 |
| gene-BPHYT_RS08480 | 84.5991017 | 77.8764548 | 93.5381351 | 239.590025 | 231.752912 | 206.425701 | -1.40309682 | 4.96E-12 | 2.01E-11 |
| gene-BPHYT_RS05915 | 587.405084 | 552.602789 | 535.810839 | 298.748056 | 265.394464 | 218.195588 | 1.10131668 | 5.19E-12 | 2.10E-11 |
| gene-BPHYT_RS15750 | 28185.0687 | 31242.3268 | 27182.9954 | 17033.569 | 18553.9388 | 17470.133 | 0.70700822 | 5.38E-12 | 2.18E-11 |
| gene-BPHYT_RS16100 | 272.951819 | 275.234594 | 302.98222 | 110.428324 | 61.0531865 | 113.171985 | 1.56699625 | 5.45E-12 | 2.20E-11 |
| gene-BPHYT_RS08815 | 532.335857 | 602.742424 | 596.81397 | 1236.40285 | 1000.52467 | 1056.57366 | -0.92915552 | 5.46E-12 | 2.20E-11 |
| gene-BPHYT_RS15985 | 2604.21574 | 3109.72419 | 2624.15138 | 4971.24653 | 4951.53803 | 4516.0149 | -0.79238668 | 5.46E-12 | 2.20E-11 |
| gene-BPHYT_RS34520 | 7.98104733 | 6.4008045 | 5.0835943 | 38.4527201 | 112.138506 | 78.7677018 | -3.53727336 | 5.60E-12 | 2.26E-11 |
| gene-BPHYT_RS12160 | 972.88967 | 962.254277 | 897.762753 | 1545.01058 | 1723.19504 | 1602.51531 | -0.78099144 | 5.64E-12 | 2.27E-11 |
| gene-BPHYT_RS08555 | 39.9052367 | 46.939233 | 63.0365693 | 191.277633 | 181.913576 | 142.144014 | -1.78726582 | 5.81E-12 | 2.34E-11 |
| gene-BPHYT_RS09495 | 727.073412 | 744.626924 | 662.900696 | 384.527201 | 380.024937 | 373.92024 | 0.90716467 | 5.96E-12 | 2.40E-11 |
| gene-BPHYT_RS26255 | 109.340348 | 93.878466 | 124.039701 | 23.6632124 | 28.6576182 | 11.7698865 | 2.37931805 | 6.04E-12 | 2.43E-11 |
| gene-BPHYT_RS06475 | 932.984433 | 844.906194 | 956.732447 | 532.422278 | 413.666488 | 445.444934 | 0.9733301 | 6.06E-12 | 2.43E-11 |
| gene-BPHYT_RS14155 | 3369.59818 | 3735.93623 | 4103.47732 | 6119.8983 | 6416.8145 | 6415.49351 | -0.75799692 | 6.12E-12 | 2.46E-11 |
| gene-BPHYT_RS27175 | 1229.87939 | 1218.28646 | 1265.81498 | 722.713945 | 717.686437 | 736.975969 | 0.77005502 | 6.35E-12 | 2.55E-11 |
| gene-BPHYT_RS17680 | 1341.61406 | 1322.83293 | 1301.40014 | 2175.0436 | 2111.94186 | 2164.75374 | -0.70206037 | 6.43E-12 | 2.58E-11 |
| gene-BPHYT_RS01895 | 78.2142639 | 107.746876 | 113.872512 | 20.7053108 | 21.1817178 | 19.0128935 | 2.29922581 | 7.05E-12 | 2.82E-11 |
| gene-BPHYT_RS21355 | 717.496155 | 726.491311 | 971.98323 | 411.148315 | 323.955684 | 365.771857 | 1.13144757 | 7.09E-12 | 2.84E-11 |
| gene-BPHYT_RS03010 | 299.289275 | 322.173827 | 331.450348 | 619.187391 | 621.745716 | 581.251317 | -0.9361189 | 8.06E-12 | 3.22E-11 |
| gene-BPHYT_RS06590 | 181.967879 | 195.224537 | 193.176583 | 429.881692 | 362.581169 | 429.148169 | -1.10237807 | 8.40E-12 | 3.36E-11 |
| gene-BPHYT_RS22765 | 106.946034 | 94.9452668 | 109.805637 | 250.435664 | 241.720779 | 273.423517 | -1.29622895 | 8.40E-12 | 3.36E-11 |
| gene-BPHYT_RS25975 | 82.2047875 | 89.611263 | 73.2037579 | 10.845639 | 1.2459834 | 15.39139 | 3.08905625 | 8.69E-12 | 3.47E-11 |
| gene-BPHYT_RS23160 | 311.260846 | 393.649477 | 289.764875 | 141.979274 | 138.304157 | 124.941872 | 1.29564964 | 8.80E-12 | 3.51E-11 |
| gene-BPHYT_RS09675 | 19136.1572 | 15553.9549 | 12380.5856 | 7030.93198 | 8073.97242 | 8057.84536 | 1.02314627 | 9.18E-12 | 3.66E-11 |
| gene-BPHYT_RS15885 | 4610.65104 | 5183.58485 | 4819.24739 | 7713.22127 | 8206.04666 | 7639.5617 | -0.68905131 | 9.28E-12 | 3.70E-11 |
| gene-BPHYT_RS01415 | 245.816258 | 228.295361 | 247.062683 | 100.568653 | 88.4648213 | 99.5913471 | 1.31802603 | 9.52E-12 | 3.79E-11 |
| gene-BPHYT_RS09545 | 2324.87909 | 2381.09928 | 1928.71568 | 940.612692 | 1277.13298 | 1154.35425 | 0.9774273 | 9.70E-12 | 3.86E-11 |
| gene-BPHYT_RS12645 | 1083.82623 | 847.039796 | 652.733508 | 384.527201 | 336.415518 | 377.541743 | 1.23402827 | 1.03E-11 | 4.09E-11 |
| gene-BPHYT_RS29890 | 1462.92598 | 1742.08563 | 1774.17441 | 993.85492 | 817.365109 | 798.541529 | 0.9310157 | 1.10E-11 | 4.38E-11 |
| gene-BPHYT_RS06675 | 1022.37216 | 1082.80276 | 1053.32074 | 1891.08506 | 1830.34961 | 1655.02711 | -0.76745121 | 1.13E-11 | 4.50E-11 |
| gene-BPHYT_RS01910 | 5347.30171 | 6703.77591 | 6473.44898 | 10179.1252 | 11140.3376 | 10768.5408 | -0.79281583 | 1.18E-11 | 4.66E-11 |
| gene-BPHYT_RS04245 | 794.11421 | 1145.74401 | 1001.46808 | 2094.1943 | 1773.03438 | 2027.1366 | -1.00484303 | 1.18E-11 | 4.68E-11 |
| gene-BPHYT_RS27150 | 213.093964 | 201.625342 | 163.691736 | 36.4807857 | 46.1013857 | 75.1461983 | 1.86592764 | 1.23E-11 | 4.86E-11 |
| gene-BPHYT_RS13675 | 7159.79756 | 8600.54765 | 7347.8272 | 3829.49654 | 4687.38955 | 4467.12461 | 0.8317367 | 1.25E-11 | 4.96E-11 |
| gene-BPHYT_RS02285 | 1811.69774 | 2137.8687 | 1861.61223 | 3228.05656 | 3228.34299 | 3285.60908 | -0.74597589 | 1.26E-11 | 5.00E-11 |
| gene-BPHYT_RS18890 | 414.216357 | 403.250684 | 403.637387 | 682.28929 | 818.611093 | 854.674834 | -0.94737601 | 1.37E-11 | 5.41E-11 |
| gene-BPHYT_RS08895 | 783.738848 | 763.829337 | 882.51197 | 455.516838 | 388.74682 | 436.391176 | 0.92195781 | 1.44E-11 | 5.68E-11 |
| gene-BPHYT_RS31100 | 24.7412467 | 30.9372218 | 29.4848469 | 106.484456 | 112.138506 | 109.550482 | -1.95347672 | 1.66E-11 | 6.56E-11 |
| gene-BPHYT_RS23060 | 264.172667 | 261.366184 | 221.644711 | 466.362477 | 637.9435 | 533.266395 | -1.12894887 | 1.69E-11 | 6.68E-11 |
| gene-BPHYT_RS14580 | 1618.5564 | 2227.47997 | 2304.90165 | 984.981215 | 944.455416 | 1109.99083 | 1.01555423 | 1.71E-11 | 6.73E-11 |
| gene-BPHYT_RS17895 | 557.077104 | 610.210029 | 625.282099 | 307.621761 | 315.2338 | 317.786935 | 0.92933056 | 1.71E-11 | 6.73E-11 |
| gene-BPHYT_RS38370 | 290.510123 | 318.973424 | 328.400192 | 148.881045 | 129.582273 | 101.402099 | 1.30578969 | 1.71E-11 | 6.74E-11 |
| gene-BPHYT_RS07780 | 407.831519 | 496.062349 | 554.111778 | 979.065412 | 983.080901 | 899.038252 | -0.97451666 | 1.80E-11 | 7.08E-11 |
| gene-BPHYT_RS13215 | 221.075011 | 234.696165 | 246.045964 | 476.222149 | 451.04599 | 465.363204 | -0.99071818 | 1.85E-11 | 7.25E-11 |
| gene-BPHYT_RS33480 | 4161.31808 | 7049.41936 | 9627.31088 | 1356.69084 | 1482.72024 | 1018.54787 | 2.4334669 | 1.90E-11 | 7.46E-11 |
| gene-BPHYT_RS05505 | 425.389823 | 334.975436 | 271.463935 | 878.496759 | 728.900288 | 729.732962 | -1.17658875 | 1.93E-11 | 7.55E-11 |
| gene-BPHYT_RS19740 | 204.314812 | 254.965379 | 222.66143 | 65.073834 | 102.170639 | 66.9978153 | 1.5527097 | 2.01E-11 | 7.87E-11 |
| gene-BPHYT_RS30735 | 219.478802 | 228.295361 | 201.310334 | 95.6388167 | 71.0210537 | 70.6193189 | 1.44929564 | 2.04E-11 | 7.99E-11 |
| gene-BPHYT_RS08860 | 3818.93115 | 4968.09109 | 5796.31422 | 9211.89138 | 9901.83007 | 9370.64039 | -0.96612411 | 2.13E-11 | 8.32E-11 |
| gene-BPHYT_RS12405 | 170.794413 | 262.432985 | 208.427366 | 647.780439 | 434.848206 | 528.739516 | -1.33419956 | 2.13E-11 | 8.33E-11 |
| gene-BPHYT_RS16120 | 216.286383 | 222.961357 | 215.544398 | 81.8352761 | 62.2991699 | 94.1590918 | 1.44715335 | 2.26E-11 | 8.82E-11 |
| gene-BPHYT_RS02415 | 3749.49604 | 4447.49233 | 4260.05202 | 7058.53906 | 6730.80232 | 6691.63315 | -0.71768517 | 2.30E-11 | 8.97E-11 |
| gene-BPHYT_RS02720 | 411.023938 | 370.17986 | 336.533942 | 677.359454 | 711.456521 | 833.851188 | -0.99075651 | 2.36E-11 | 9.19E-11 |
| gene-BPHYT_RS07740 | 1597.80568 | 1838.09769 | 1335.96858 | 729.615715 | 878.418296 | 827.513557 | 0.97091469 | 2.39E-11 | 9.31E-11 |
| gene-BPHYT_RS12335 | 348.771768 | 261.366184 | 283.664562 | 136.063471 | 99.6786719 | 80.5784536 | 1.50140684 | 2.40E-11 | 9.36E-11 |
| gene-BPHYT_RS01625 | 836.41376 | 951.586269 | 824.558995 | 1417.82081 | 1663.38784 | 1551.81426 | -0.82658292 | 2.49E-11 | 9.68E-11 |
| gene-BPHYT_RS20060 | 232.248477 | 200.558541 | 211.477523 | 44.3685232 | 87.2188379 | 74.2408224 | 1.65606674 | 2.60E-11 | 1.01E-10 |
| gene-BPHYT_RS19050 | 419.80309 | 401.117082 | 336.533942 | 166.628454 | 194.37341 | 155.724652 | 1.16927344 | 2.70E-11 | 1.05E-10 |
| gene-BPHYT_RS02210 | 2144.50742 | 2670.20228 | 3016.60486 | 1429.65241 | 1255.95127 | 1397.90036 | 0.93838249 | 2.81E-11 | 1.09E-10 |
| gene-BPHYT_RS00145 | 412.620147 | 540.86798 | 553.095059 | 925.823184 | 1108.92523 | 982.332833 | -1.00348732 | 3.01E-11 | 1.17E-10 |
| gene-BPHYT_RS32660 | 59.857855 | 50.1396353 | 70.1536013 | 3.94386873 | 7.47590039 | 7.24300706 | 3.28171425 | 3.03E-11 | 1.17E-10 |
| gene-BPHYT_RS30015 | 72.6275307 | 84.2772593 | 46.7690675 | 8.87370464 | 6.22991699 | 0 | 3.79721196 | 3.15E-11 | 1.22E-10 |
| gene-BPHYT_RS33870 | 2058.31211 | 2271.2188 | 2375.05526 | 3738.78756 | 3527.379 | 3678.54221 | -0.70763702 | 3.20E-11 | 1.24E-10 |
| gene-BPHYT_RS22650 | 196.333764 | 233.629364 | 302.98222 | 91.694948 | 89.7108047 | 76.9569501 | 1.50448062 | 3.23E-11 | 1.25E-10 |
| gene-BPHYT_RS12460 | 283.32718 | 269.90059 | 281.631124 | 103.526554 | 137.058174 | 112.26661 | 1.24943281 | 3.33E-11 | 1.29E-10 |
| gene-BPHYT_RS12090 | 3974.56157 | 4659.78568 | 4717.57551 | 7273.4799 | 7159.42061 | 7575.28001 | -0.72138603 | 3.42E-11 | 1.32E-10 |
| gene-BPHYT_RS14915 | 3352.83798 | 4031.44004 | 3933.68527 | 6663.16622 | 6253.59068 | 6058.77541 | -0.74590815 | 3.56E-11 | 1.37E-10 |
| gene-BPHYT_RS35065 | 6653.79916 | 7012.08133 | 6197.91817 | 3520.88881 | 3953.50532 | 4284.23868 | 0.75633795 | 3.58E-11 | 1.38E-10 |
| gene-BPHYT_RS18190 | 1047.91152 | 1302.56372 | 1324.78467 | 2381.11075 | 2211.62053 | 2066.06777 | -0.85845049 | 3.60E-11 | 1.39E-10 |
| gene-BPHYT_RS17915 | 904.252663 | 783.031751 | 1013.6687 | 1632.76165 | 1596.10473 | 1607.04219 | -0.84004486 | 3.73E-11 | 1.44E-10 |
| gene-BPHYT_RS26485 | 118.917605 | 148.285304 | 132.173452 | 36.4807857 | 43.6094189 | 19.9182694 | 2.01622061 | 4.10E-11 | 1.58E-10 |
| gene-BPHYT_RS14910 | 1009.60249 | 1259.89169 | 1229.2131 | 2104.05397 | 1973.6377 | 2069.68927 | -0.81461749 | 4.19E-11 | 1.61E-10 |
| gene-BPHYT_RS01770 | 2581.86881 | 2510.18217 | 2098.50773 | 1417.82081 | 1326.97232 | 1399.71112 | 0.7948213 | 4.27E-11 | 1.64E-10 |
| gene-BPHYT_RS04295 | 530.739648 | 512.06436 | 614.098191 | 1172.31498 | 1041.64212 | 934.347911 | -0.92615647 | 4.27E-11 | 1.64E-10 |
| gene-BPHYT_RS31070 | 3.19241893 | 4.267203 | 2.03343772 | 70.0036699 | 32.3955684 | 58.8494324 | -4.09812288 | 4.29E-11 | 1.65E-10 |
| gene-BPHYT_RS15625 | 785.335058 | 855.574202 | 904.879785 | 499.885361 | 444.816073 | 442.728807 | 0.87442551 | 4.33E-11 | 1.66E-10 |
| gene-BPHYT_RS22485 | 3885.97195 | 3251.60869 | 2514.34574 | 1385.28389 | 1630.99227 | 1680.37764 | 1.0396448 | 4.35E-11 | 1.67E-10 |
| gene-BPHYT_RS03350 | 2786.98173 | 2872.89442 | 3201.64769 | 5262.10685 | 4815.72584 | 4608.36324 | -0.72903045 | 4.49E-11 | 1.72E-10 |
| gene-BPHYT_RS21500 | 217.882592 | 311.505819 | 244.012526 | 112.400259 | 80.9889209 | 68.8085671 | 1.55822246 | 4.52E-11 | 1.73E-10 |
| gene-BPHYT_RS26260 | 399.850471 | 405.384285 | 362.968633 | 190.291666 | 124.59834 | 180.169801 | 1.2314222 | 4.56E-11 | 1.75E-10 |
| gene-BPHYT_RS02110 | 1070.25845 | 1104.13878 | 958.765884 | 528.47841 | 605.547932 | 604.79109 | 0.85013779 | 4.74E-11 | 1.81E-10 |
| gene-BPHYT_RS08325 | 1283.35241 | 1184.14883 | 896.746034 | 482.137952 | 551.970646 | 593.926579 | 1.04769182 | 4.85E-11 | 1.85E-10 |
| gene-BPHYT_RS01840 | 602.569074 | 678.485277 | 538.860995 | 281.000647 | 241.720779 | 331.367573 | 1.08688845 | 4.94E-11 | 1.89E-10 |
| gene-BPHYT_RS15745 | 475.670421 | 408.584687 | 510.392867 | 905.117873 | 915.797798 | 805.784536 | -0.91180085 | 4.96E-11 | 1.89E-10 |
| gene-BPHYT_RS04915 | 1424.61695 | 1199.08404 | 1128.55793 | 2159.26813 | 2156.79726 | 2256.1967 | -0.80762508 | 5.01E-11 | 1.91E-10 |
| gene-BPHYT_RS14250 | 5852.50201 | 7621.22456 | 6867.93589 | 3859.07555 | 4093.05546 | 3692.12285 | 0.80478662 | 5.01E-11 | 1.91E-10 |
| gene-BPHYT_RS24015 | 434.96708 | 440.58871 | 435.155672 | 210.01101 | 225.522995 | 229.060098 | 0.97990536 | 5.22E-11 | 1.99E-10 |
| gene-BPHYT_RS04375 | 217.882592 | 201.625342 | 259.263309 | 478.194083 | 477.211642 | 442.728807 | -1.04222698 | 5.31E-11 | 2.02E-10 |
| gene-BPHYT_RS03215 | 9185.38737 | 5223.05647 | 5112.06242 | 2858.31886 | 2786.01888 | 2611.10405 | 1.24190949 | 5.40E-11 | 2.05E-10 |
| gene-BPHYT_RS02250 | 2592.24417 | 2626.46345 | 2453.34261 | 1545.99654 | 1545.01941 | 1668.60775 | 0.68839341 | 5.42E-11 | 2.06E-10 |
| gene-BPHYT_RS17725 | 498.017354 | 668.88407 | 773.723052 | 1296.54685 | 1328.2183 | 1341.76706 | -1.03349549 | 5.67E-11 | 2.15E-10 |
| gene-BPHYT_RS17535 | 267.365086 | 297.637409 | 174.875644 | 80.8493089 | 88.4648213 | 83.2945812 | 1.55257403 | 5.67E-11 | 2.15E-10 |
| gene-BPHYT_RS29055 | 63.8483787 | 83.2104585 | 96.5882916 | 218.884715 | 223.031028 | 210.047205 | -1.42714676 | 5.74E-11 | 2.18E-10 |
| gene-BPHYT_RS16360 | 10054.5234 | 11615.3266 | 13361.7193 | 19817.9404 | 20786.741 | 20044.1167 | -0.79192303 | 5.79E-11 | 2.19E-10 |
| gene-BPHYT_RS16340 | 18047.5423 | 23166.6451 | 23174.073 | 35509.6081 | 38796.1851 | 37808.4969 | -0.80017058 | 5.94E-11 | 2.25E-10 |
| gene-BPHYT_RS12965 | 1684.00099 | 1853.0329 | 2104.60804 | 1093.43761 | 1102.69531 | 1007.68336 | 0.81631649 | 6.08E-11 | 2.30E-10 |
| gene-BPHYT_RS02440 | 3818.93115 | 4432.55712 | 3819.81275 | 2526.04792 | 2453.34131 | 2299.65474 | 0.72969736 | 6.23E-11 | 2.36E-10 |
| gene-BPHYT_RS23545 | 169.996308 | 125.882489 | 198.260178 | 46.3404576 | 47.3473691 | 56.1333047 | 1.71902643 | 6.38E-11 | 2.41E-10 |
| gene-BPHYT_RS07565 | 2512.4337 | 2574.19021 | 2015.13678 | 1317.25216 | 1350.646 | 1351.72619 | 0.82132276 | 6.57E-11 | 2.48E-10 |
| gene-BPHYT_RS16175 | 5718.42041 | 6689.90751 | 5159.84821 | 9640.78711 | 10096.2035 | 10442.6054 | -0.78071643 | 6.71E-11 | 2.53E-10 |
| gene-BPHYT_RS08100 | 1778.97545 | 2166.67232 | 2443.17542 | 1212.73963 | 1103.94129 | 929.821032 | 0.97658985 | 6.80E-11 | 2.56E-10 |
| gene-BPHYT_RS21265 | 197.929974 | 163.220515 | 231.8119 | 68.0317356 | 71.0210537 | 72.4300706 | 1.48797628 | 6.86E-11 | 2.58E-10 |
| gene-BPHYT_RS06425 | 2292.9549 | 2327.75924 | 2134.09289 | 1070.76036 | 1396.74739 | 1315.51116 | 0.83718358 | 7.08E-11 | 2.66E-10 |
| gene-BPHYT_RS33845 | 1302.50693 | 1424.179 | 1447.80766 | 876.524825 | 776.247657 | 748.745855 | 0.7970201 | 7.33E-11 | 2.76E-10 |
| gene-BPHYT_RS24735 | 367.128177 | 327.50783 | 318.233003 | 149.867012 | 159.485875 | 162.967659 | 1.10222588 | 7.50E-11 | 2.82E-10 |
| gene-BPHYT_RS26555 | 287.317704 | 334.975436 | 324.333316 | 133.10557 | 134.566207 | 155.724652 | 1.15632008 | 8.13E-11 | 3.05E-10 |
| gene-BPHYT_RS24895 | 131.687281 | 117.348083 | 122.006263 | 29.5790155 | 38.6254854 | 11.7698865 | 2.24378619 | 8.67E-11 | 3.25E-10 |
| gene-BPHYT_RS08670 | 93.3782538 | 80.0100563 | 55.9195373 | 8.87370464 | 2.4919668 | 15.39139 | 3.04402558 | 8.80E-11 | 3.30E-10 |
| gene-BPHYT_RS22075 | 5586.73313 | 4958.48989 | 3276.88488 | 1919.6781 | 2318.7751 | 2140.30859 | 1.11608056 | 8.97E-11 | 3.36E-10 |
| gene-BPHYT_RS31155 | 25.5393515 | 39.4716278 | 30.5015658 | 126.203799 | 109.646539 | 112.26661 | -1.87885733 | 8.99E-11 | 3.37E-10 |
| gene-BPHYT_RS13420 | 67.8389023 | 73.6092518 | 38.6353167 | 1.97193437 | 3.7379502 | 8.14838295 | 3.66641854 | 9.27E-11 | 3.47E-10 |
| gene-BPHYT_RS19685 | 319.241893 | 337.109037 | 322.299878 | 157.754749 | 156.993908 | 150.292397 | 1.07373761 | 9.40E-11 | 3.52E-10 |
| gene-BPHYT_RS13325 | 463.69885 | 424.586699 | 383.30301 | 193.249568 | 213.063161 | 213.668708 | 1.03827659 | 9.96E-11 | 3.72E-10 |
| gene-BPHYT_RS01695 | 2599.42712 | 2942.23647 | 2438.09183 | 4322.48013 | 4576.49702 | 4267.03654 | -0.72244373 | 1.04E-10 | 3.89E-10 |
| gene-BPHYT_RS05980 | 3281.80666 | 6877.66444 | 3883.86604 | 20888.7007 | 23949.0469 | 21720.8728 | -2.24483933 | 1.07E-10 | 3.99E-10 |
| gene-BPHYT_RS22730 | 462.102641 | 453.390319 | 507.342711 | 254.379533 | 245.45873 | 229.965474 | 0.96373954 | 1.07E-10 | 4.00E-10 |
| gene-BPHYT_RS02280 | 2928.24627 | 3049.98335 | 2716.67279 | 4531.50517 | 4976.45769 | 4475.27299 | -0.68519772 | 1.07E-10 | 4.01E-10 |
| gene-BPHYT_RS06665 | 3767.85245 | 3681.52939 | 3532.08132 | 5599.30763 | 6069.18513 | 5512.83375 | -0.6454431 | 1.11E-10 | 4.13E-10 |
| gene-BPHYT_RS35730 | 559.471418 | 202.692143 | 467.690675 | 3501.16946 | 2516.88647 | 2711.60077 | -2.82659262 | 1.21E-10 | 4.50E-10 |
| gene-BPHYT_RS12525 | 11299.5668 | 12250.073 | 13236.6628 | 19238.1917 | 19223.0319 | 19255.5343 | -0.64991332 | 1.40E-10 | 5.21E-10 |
| gene-BPHYT_RS02615 | 114.927082 | 87.4776615 | 81.3375087 | 21.691278 | 18.689751 | 23.539773 | 2.145721 | 1.45E-10 | 5.38E-10 |
| gene-BPHYT_RS09345 | 168.400099 | 209.092947 | 191.143146 | 73.9475387 | 74.7590039 | 64.2816877 | 1.41784211 | 1.54E-10 | 5.73E-10 |
| gene-BPHYT_RS09670 | 3091.85774 | 3626.05575 | 3385.6738 | 1959.11679 | 2035.93687 | 2165.65911 | 0.71317436 | 1.58E-10 | 5.88E-10 |
| gene-BPHYT_RS06155 | 4747.92506 | 5473.75465 | 5718.02687 | 3249.74783 | 3340.48149 | 2892.67595 | 0.74932468 | 1.60E-10 | 5.93E-10 |
| gene-BPHYT_RS33855 | 1674.42373 | 1979.98219 | 2090.37398 | 1190.06239 | 903.337964 | 921.672649 | 0.92897177 | 1.65E-10 | 6.13E-10 |
| gene-BPHYT_RS09440 | 1620.15261 | 1610.86913 | 1608.44924 | 2479.70746 | 2571.70973 | 2987.74041 | -0.73254095 | 1.71E-10 | 6.34E-10 |
| gene-BPHYT_RS17110 | 1142.88598 | 1349.50295 | 1608.44924 | 2669.01316 | 2353.66264 | 2571.26751 | -0.89007782 | 1.72E-10 | 6.36E-10 |
| gene-BPHYT_RS04320 | 30.3279799 | 32.0040225 | 35.5851601 | 116.344128 | 112.138506 | 107.73973 | -1.78288864 | 1.72E-10 | 6.36E-10 |
| gene-BPHYT_RS20970 | 260.182143 | 216.560552 | 63.0365693 | 25.6351467 | 28.6576182 | 34.4042836 | 2.60505144 | 1.75E-10 | 6.46E-10 |
| gene-BPHYT_RS03545 | 6112.68415 | 5726.58643 | 4898.55146 | 3057.48423 | 3513.67318 | 3278.36607 | 0.76540645 | 1.79E-10 | 6.63E-10 |
| gene-BPHYT_RS16085 | 579.424036 | 658.216063 | 758.472269 | 1257.10816 | 1169.97841 | 1184.23166 | -0.85704073 | 1.88E-10 | 6.94E-10 |
| gene-BPHYT_RS17200 | 9363.36473 | 9543.59951 | 10384.7664 | 6476.81842 | 6378.18902 | 6024.37113 | 0.63370178 | 1.96E-10 | 7.22E-10 |
| gene-BPHYT_RS33565 | 1358.37426 | 1257.75809 | 1476.27578 | 842.015974 | 788.707491 | 717.057699 | 0.80220966 | 1.96E-10 | 7.23E-10 |
| gene-BPHYT_RS28160 | 75.0218449 | 25.603218 | 87.4378219 | 3.94386873 | 1.2459834 | 5.4322553 | 4.08964723 | 1.97E-10 | 7.26E-10 |
| gene-BPHYT_RS35255 | 286.519599 | 250.698176 | 265.363622 | 127.189767 | 112.138506 | 116.793489 | 1.17197435 | 2.14E-10 | 7.87E-10 |
| gene-BPHYT_RS02040 | 1228.28318 | 1082.80276 | 772.706333 | 492.983591 | 483.441559 | 492.52448 | 1.07069037 | 2.17E-10 | 8.00E-10 |
| gene-BPHYT_RS35475 | 133.28349 | 178.155725 | 153.524548 | 50.2843263 | 46.1013857 | 57.0386806 | 1.5903449 | 2.21E-10 | 8.14E-10 |
| gene-BPHYT_RS29150 | 83.800997 | 80.0100563 | 93.5381351 | 224.800518 | 211.817178 | 195.561191 | -1.29633496 | 2.25E-10 | 8.26E-10 |
| gene-BPHYT_RS17890 | 614.540645 | 617.677634 | 564.278967 | 1127.94646 | 970.621067 | 1021.264 | -0.79663963 | 2.31E-10 | 8.47E-10 |
| gene-BPHYT_RS21235 | 24544.913 | 20382.2951 | 15105.3921 | 37003.3484 | 40125.6494 | 41624.6562 | -0.98408731 | 2.40E-10 | 8.80E-10 |
| gene-BPHYT_RS20615 | 232.248477 | 208.026146 | 159.624861 | 49.2983591 | 47.3473691 | 85.105333 | 1.71264836 | 2.47E-10 | 9.07E-10 |
| gene-BPHYT_RS06540 | 1982.49216 | 2186.94154 | 2389.28932 | 1337.95747 | 1334.44822 | 1260.28323 | 0.73771611 | 2.50E-10 | 9.15E-10 |
| gene-BPHYT_RS16155 | 942.56169 | 998.525502 | 935.381351 | 471.292313 | 505.86926 | 602.074962 | 0.86341593 | 2.56E-10 | 9.36E-10 |
| gene-BPHYT_RS03820 | 246.614363 | 150.418906 | 298.915345 | 495.941493 | 666.601118 | 662.735146 | -1.38885806 | 2.59E-10 | 9.49E-10 |
| gene-BPHYT_RS34775 | 306.472218 | 249.631376 | 255.196434 | 506.787132 | 561.938513 | 515.158877 | -0.96071047 | 2.81E-10 | 1.03E-09 |
| gene-BPHYT_RS25795 | 1968.92438 | 1702.614 | 1992.76896 | 1012.5883 | 1204.86595 | 1075.58655 | 0.78384543 | 2.86E-10 | 1.05E-09 |
| gene-BPHYT_RS06410 | 2233.89515 | 2584.85822 | 3441.59334 | 5302.53151 | 5483.57294 | 5099.98235 | -0.943833 | 2.89E-10 | 1.06E-09 |
| gene-BPHYT_RS23690 | 4233.94561 | 5103.57479 | 4896.51803 | 7257.70443 | 7938.16023 | 8119.41092 | -0.71221756 | 2.97E-10 | 1.08E-09 |
| gene-BPHYT_RS19645 | 431.774661 | 421.386296 | 446.339579 | 742.433288 | 855.990595 | 729.732962 | -0.83960943 | 2.98E-10 | 1.09E-09 |
| gene-BPHYT_RS28775 | 306.472218 | 263.499785 | 270.447217 | 133.10557 | 129.582273 | 102.307475 | 1.20898303 | 3.05E-10 | 1.11E-09 |
| gene-BPHYT_RS04080 | 284.125285 | 395.783078 | 416.854732 | 777.928107 | 793.691425 | 686.274919 | -1.0444666 | 3.10E-10 | 1.13E-09 |
| gene-BPHYT_RS02475 | 1375.93256 | 1378.30657 | 1524.06157 | 2489.56714 | 2194.17676 | 2294.22249 | -0.70630322 | 3.24E-10 | 1.18E-09 |
| gene-BPHYT_RS17215 | 3326.50053 | 3592.98493 | 4087.20981 | 5994.68047 | 6206.24331 | 5775.39276 | -0.70788553 | 3.45E-10 | 1.26E-09 |
| gene-BPHYT_RS35225 | 185.160298 | 203.758943 | 214.527679 | 401.288643 | 423.634355 | 376.636367 | -0.9945282 | 3.61E-10 | 1.31E-09 |
| gene-BPHYT_RS15025 | 1081.43191 | 1215.08606 | 1177.36044 | 2275.61226 | 2065.84047 | 1758.23997 | -0.81246553 | 3.68E-10 | 1.34E-09 |
| gene-BPHYT_RS22855 | 113.330872 | 135.483695 | 128.106576 | 263.253238 | 271.624381 | 295.152538 | -1.14352574 | 3.78E-10 | 1.37E-09 |
| gene-BPHYT_RS12935 | 2314.50373 | 2351.22885 | 1535.24548 | 1021.462 | 1057.83991 | 1023.07475 | 0.99954059 | 3.80E-10 | 1.38E-09 |
| gene-BPHYT_RS18425 | 153.236109 | 152.552507 | 156.574704 | 48.3123919 | 64.7911367 | 50.7010495 | 1.50657685 | 3.95E-10 | 1.43E-09 |
| gene-BPHYT_RS06760 | 2690.41106 | 2901.69804 | 2492.99464 | 1616.98618 | 1457.80058 | 1745.5647 | 0.74516285 | 4.08E-10 | 1.48E-09 |
| gene-BPHYT_RS06670 | 2884.35051 | 3063.85176 | 3459.89428 | 5353.8018 | 5264.27986 | 4771.3309 | -0.71010672 | 4.40E-10 | 1.59E-09 |
| gene-BPHYT_RS09215 | 743.035507 | 663.550067 | 641.5496 | 403.260578 | 264.14848 | 316.881559 | 1.05472093 | 4.68E-10 | 1.69E-09 |
| gene-BPHYT_RS18985 | 27.1355609 | 35.2044248 | 24.4012526 | 98.5967182 | 109.646539 | 104.118227 | -1.8525981 | 4.73E-10 | 1.71E-09 |
| gene-BPHYT_RS24010 | 252.9992 | 260.299383 | 241.979089 | 95.6388167 | 109.646539 | 123.13112 | 1.19991407 | 4.74E-10 | 1.71E-09 |
| gene-BPHYT_RS30865 | 58.2616455 | 81.076857 | 94.5548539 | 7.88773746 | 19.9357344 | 3.62150353 | 2.95690613 | 4.96E-10 | 1.79E-09 |
| gene-BPHYT_RS28055 | 83.0028923 | 71.4756503 | 58.9696938 | 0 | 1.2459834 | 0.90537588 | 6.69907547 | 5.33E-10 | 1.92E-09 |
| gene-BPHYT_RS26285 | 227.459849 | 180.289327 | 162.675018 | 65.073834 | 57.3152363 | 79.6730777 | 1.49245301 | 5.34E-10 | 1.93E-09 |
| gene-BPHYT_RS02290 | 4682.48047 | 4985.15991 | 4603.703 | 7067.41276 | 7119.54914 | 7625.07569 | -0.61217562 | 5.39E-10 | 1.94E-09 |
| gene-BPHYT_RS08790 | 932.984433 | 975.055886 | 2047.67178 | 6903.74221 | 7520.75579 | 8658.10957 | -2.54495845 | 5.48E-10 | 1.97E-09 |
| gene-BPHYT_RS25970 | 75.0218449 | 76.809654 | 45.7523487 | 5.91580309 | 12.459834 | 9.95913471 | 2.82228522 | 5.67E-10 | 2.04E-09 |
| gene-BPHYT_RS22960 | 0 | 9.60120675 | 8.13375087 | 364.807857 | 441.078123 | 601.169586 | -6.335325 | 5.96E-10 | 2.14E-09 |
| gene-BPHYT_RS35655 | 2383.14073 | 2388.56688 | 2153.41054 | 3495.25366 | 3650.73136 | 3629.65192 | -0.63765701 | 6.02E-10 | 2.16E-09 |
| gene-BPHYT_RS12190 | 6419.15637 | 6831.792 | 7412.8972 | 10156.4479 | 11038.1669 | 11281.8889 | -0.652404 | 6.36E-10 | 2.28E-09 |
| gene-BPHYT_RS16770 | 1340.81595 | 1379.37337 | 1351.21936 | 2069.54512 | 2224.08037 | 2076.93228 | -0.64563705 | 6.50E-10 | 2.33E-09 |
| gene-BPHYT_RS37345 | 596.184236 | 510.997559 | 477.857864 | 275.084844 | 282.838231 | 269.802013 | 0.93984288 | 6.52E-10 | 2.34E-09 |
| gene-BPHYT_RS04830 | 2553.13704 | 2568.85621 | 2657.7031 | 1518.38946 | 1558.72523 | 1767.29372 | 0.68277636 | 6.55E-10 | 2.35E-09 |
| gene-BPHYT_RS06105 | 158.024737 | 166.420917 | 80.3207899 | 39.4386873 | 27.4116348 | 29.8774041 | 2.06154816 | 6.60E-10 | 2.36E-09 |
| gene-BPHYT_RS19705 | 272.153714 | 384.04827 | 384.319729 | 683.275257 | 716.440454 | 671.788905 | -0.99672118 | 6.64E-10 | 2.38E-09 |
| gene-BPHYT_RS14260 | 2415.06492 | 2480.31174 | 2537.73027 | 1671.21437 | 1538.7895 | 1437.7369 | 0.67745415 | 6.75E-10 | 2.41E-09 |
| gene-BPHYT_RS35650 | 252.9992 | 205.892545 | 185.042832 | 80.8493089 | 95.9407217 | 71.5246948 | 1.38505365 | 6.76E-10 | 2.42E-09 |
| gene-BPHYT_RS35280 | 67.0407976 | 77.8764548 | 72.187039 | 187.333765 | 180.667593 | 174.737545 | -1.32530403 | 6.81E-10 | 2.43E-09 |
| gene-BPHYT_RS16780 | 567.452465 | 808.634969 | 821.508838 | 1558.81412 | 1349.40002 | 1370.73909 | -0.96355744 | 6.96E-10 | 2.49E-09 |
| gene-BPHYT_RS14605 | 181.967879 | 182.422928 | 232.828619 | 468.334412 | 360.089202 | 449.066438 | -1.10077256 | 7.03E-10 | 2.51E-09 |
| gene-BPHYT_RS02335 | 1043.92099 | 1098.80477 | 1074.67183 | 1654.45293 | 1704.50529 | 1703.91741 | -0.65438456 | 7.07E-10 | 2.52E-09 |
| gene-BPHYT_RS07515 | 842.000494 | 839.57219 | 1043.15355 | 499.885361 | 389.992804 | 511.537374 | 0.95597087 | 7.12E-10 | 2.54E-09 |
| gene-BPHYT_RS20840 | 1326.45007 | 1168.14682 | 730.004141 | 446.643134 | 523.313027 | 428.242793 | 1.20772301 | 7.15E-10 | 2.54E-09 |
| gene-BPHYT_RS29350 | 76.6180544 | 85.34406 | 88.4545408 | 15.7754749 | 13.7058174 | 24.4451488 | 2.19316875 | 7.42E-10 | 2.64E-09 |
| gene-BPHYT_RS12410 | 5517.29802 | 6894.73325 | 8158.15213 | 12889.549 | 12047.4135 | 12306.7744 | -0.85667851 | 7.49E-10 | 2.66E-09 |
| gene-BPHYT_RS21280 | 104.55172 | 105.613274 | 92.5214162 | 30.5649827 | 28.6576182 | 25.3505247 | 1.84290172 | 7.74E-10 | 2.75E-09 |
| gene-BPHYT_RS16125 | 327.222941 | 232.562564 | 311.115971 | 126.203799 | 132.07424 | 92.3483401 | 1.32042162 | 7.75E-10 | 2.75E-09 |
| gene-BPHYT_RS17435 | 1122.13526 | 1518.05747 | 1406.12218 | 2402.80202 | 2281.3956 | 2414.63748 | -0.81236403 | 7.91E-10 | 2.81E-09 |
| gene-BPHYT_RS34220 | 814.864933 | 739.29292 | 683.235073 | 425.937823 | 391.238787 | 431.864296 | 0.84053674 | 7.95E-10 | 2.82E-09 |
| gene-BPHYT_RS27450 | 91.7820443 | 77.8764548 | 39.6520355 | 9.85967182 | 11.2138506 | 8.14838295 | 2.8570967 | 8.40E-10 | 2.98E-09 |
| gene-BPHYT_RS17210 | 18966.959 | 18487.657 | 19300.3741 | 27292.5576 | 28309.9888 | 27662.8547 | -0.55294639 | 8.47E-10 | 3.00E-09 |
| gene-BPHYT_RS06750 | 633.695158 | 740.359721 | 789.990554 | 426.92379 | 351.367318 | 364.866481 | 0.91827354 | 8.54E-10 | 3.02E-09 |
| gene-BPHYT_RS02525 | 2827.68507 | 3012.64532 | 2813.26108 | 1945.31325 | 1748.11471 | 1584.4078 | 0.71341812 | 8.56E-10 | 3.03E-09 |
| gene-BPHYT_RS29725 | 328.021045 | 300.837812 | 291.798313 | 126.203799 | 152.009975 | 153.9139 | 1.09349559 | 8.64E-10 | 3.05E-09 |
| gene-BPHYT_RS03300 | 1680.80857 | 1641.80636 | 2359.80447 | 3502.15543 | 3492.49147 | 3528.24982 | -0.88926983 | 8.85E-10 | 3.13E-09 |
| gene-BPHYT_RS33585 | 2314.50373 | 2327.75924 | 2274.40009 | 1529.2351 | 1500.16401 | 1355.3477 | 0.65808759 | 8.96E-10 | 3.16E-09 |
| gene-BPHYT_RS34895 | 277.740447 | 336.042236 | 324.333316 | 644.822537 | 570.660396 | 549.563161 | -0.91461266 | 9.69E-10 | 3.42E-09 |
| gene-BPHYT_RS06400 | 1068.66224 | 1201.21765 | 1131.60809 | 1777.69883 | 1776.77233 | 1867.79045 | -0.67369336 | 9.91E-10 | 3.49E-09 |
| gene-BPHYT_RS24795 | 603.367178 | 566.471198 | 584.613344 | 337.200776 | 327.693634 | 328.651446 | 0.82072346 | 1.04E-09 | 3.68E-09 |
| gene-BPHYT_RS13655 | 2774.21205 | 2790.75076 | 2688.20466 | 4070.07253 | 4142.8948 | 4152.95918 | -0.58330814 | 1.05E-09 | 3.71E-09 |
| gene-BPHYT_RS17190 | 9012.99675 | 11432.9036 | 11190.0078 | 17626.1353 | 17626.9271 | 16725.9141 | -0.71650939 | 1.12E-09 | 3.94E-09 |
| gene-BPHYT_RS17790 | 2609.80248 | 2822.75479 | 2505.19527 | 3944.8547 | 4100.53136 | 4386.54615 | -0.64749494 | 1.15E-09 | 4.04E-09 |
| gene-BPHYT_RS20680 | 788.527476 | 630.479243 | 780.840084 | 1344.85924 | 1374.31969 | 1182.4209 | -0.82489201 | 1.16E-09 | 4.09E-09 |
| gene-BPHYT_RS12355 | 1804.5148 | 1923.44175 | 2007.00303 | 3006.21394 | 2848.31805 | 3081.89951 | -0.64059857 | 1.16E-09 | 4.09E-09 |
| gene-BPHYT_RS02420 | 1589.82463 | 1837.03089 | 2159.51086 | 3332.56908 | 3093.77678 | 3232.1919 | -0.79068583 | 1.18E-09 | 4.15E-09 |
| gene-BPHYT_RS12365 | 5359.27328 | 5651.91038 | 8236.43948 | 3330.59714 | 3240.80282 | 3442.23911 | 0.94249949 | 1.20E-09 | 4.20E-09 |
| gene-BPHYT_RS04675 | 3851.65344 | 4164.79013 | 4311.90468 | 6145.53345 | 6274.77239 | 6294.17314 | -0.6024044 | 1.21E-09 | 4.22E-09 |
| gene-BPHYT_RS27140 | 345.57935 | 396.849879 | 488.025053 | 196.207469 | 199.357344 | 140.333262 | 1.20195081 | 1.29E-09 | 4.51E-09 |
| gene-BPHYT_RS15385 | 3281.80666 | 2976.37409 | 2835.6289 | 1727.4145 | 1763.06651 | 2037.09574 | 0.71803694 | 1.33E-09 | 4.65E-09 |
| gene-BPHYT_RS23205 | 394.263738 | 327.50783 | 264.346903 | 642.850603 | 691.520786 | 637.384622 | -0.99580418 | 1.34E-09 | 4.69E-09 |
| gene-BPHYT_RS16230 | 1363.16288 | 908.914239 | 748.30508 | 406.218479 | 422.388372 | 487.092225 | 1.19962959 | 1.35E-09 | 4.73E-09 |
| gene-BPHYT_RS06720 | 928.195805 | 673.151273 | 586.646782 | 350.01835 | 350.121335 | 341.326708 | 1.07313418 | 1.36E-09 | 4.74E-09 |
| gene-BPHYT_RS11300 | 193.93945 | 238.963368 | 231.8119 | 79.8633418 | 78.4969541 | 106.834354 | 1.31688652 | 1.38E-09 | 4.82E-09 |
| gene-BPHYT_RS16245 | 2225.116 | 2146.40311 | 2375.05526 | 3683.57339 | 3321.79174 | 3489.31865 | -0.63765562 | 1.44E-09 | 5.02E-09 |
| gene-BPHYT_RS26155 | 14.3658852 | 24.5364173 | 21.351096 | 76.9054402 | 87.2188379 | 87.8214607 | -2.08136773 | 1.44E-09 | 5.03E-09 |
| gene-BPHYT_RS25160 | 94.9744633 | 86.4108608 | 114.889231 | 199.165371 | 297.790032 | 249.883744 | -1.331204 | 1.48E-09 | 5.15E-09 |
| gene-BPHYT_RS23750 | 1606.58483 | 1584.19911 | 1380.70421 | 2492.52504 | 2354.90862 | 2455.3794 | -0.67564444 | 1.51E-09 | 5.27E-09 |
| gene-BPHYT_RS13435 | 217.084487 | 245.364173 | 87.4378219 | 3.94386873 | 0 | 0 | 7.08485915 | 1.54E-09 | 5.37E-09 |
| gene-BPHYT_RS18800 | 256.191619 | 280.568597 | 213.51096 | 77.8914074 | 79.7429375 | 124.941872 | 1.40083917 | 1.55E-09 | 5.38E-09 |
| gene-BPHYT_RS34695 | 1087.01865 | 1487.12025 | 1325.80139 | 2355.4756 | 2374.84436 | 2138.49784 | -0.81756663 | 1.55E-09 | 5.40E-09 |
| gene-BPHYT_RS12540 | 1561.89096 | 2001.31821 | 1829.07723 | 3119.60017 | 2915.60115 | 2941.56624 | -0.73623191 | 1.56E-09 | 5.41E-09 |
| gene-BPHYT_RS06645 | 4429.48127 | 4508.29997 | 4827.38114 | 7045.72148 | 6966.29318 | 6682.57939 | -0.58828287 | 1.60E-09 | 5.55E-09 |
| gene-BPHYT_RS24060 | 356.752816 | 304.038214 | 327.383473 | 129.161701 | 154.501941 | 175.642921 | 1.1055422 | 1.64E-09 | 5.70E-09 |
| gene-BPHYT_RS34850 | 442.150022 | 328.574631 | 353.818163 | 195.221502 | 135.81219 | 103.212851 | 1.37510538 | 1.65E-09 | 5.72E-09 |
| gene-BPHYT_RS26720 | 304.077903 | 396.849879 | 444.306142 | 780.886008 | 703.98062 | 743.3136 | -0.96383372 | 1.77E-09 | 6.12E-09 |
| gene-BPHYT_RS30505 | 271.355609 | 296.570609 | 330.433629 | 149.867012 | 122.106373 | 143.954765 | 1.10517158 | 1.81E-09 | 6.28E-09 |
| gene-BPHYT_RS17875 | 703.13027 | 601.675623 | 443.289423 | 1252.17832 | 1128.86096 | 1100.93707 | -0.99237399 | 1.83E-09 | 6.33E-09 |
| gene-BPHYT_RS11580 | 129.292967 | 166.420917 | 154.541267 | 56.2001294 | 51.0853193 | 54.322553 | 1.47267979 | 1.85E-09 | 6.40E-09 |
| gene-BPHYT_RS19305 | 1118.94284 | 1266.29249 | 1502.71047 | 2374.20898 | 2382.32026 | 2076.93228 | -0.81410444 | 1.87E-09 | 6.48E-09 |
| gene-BPHYT_RS04975 | 1426.21316 | 1662.07557 | 1648.10127 | 2564.50064 | 2544.2981 | 2438.17725 | -0.67279159 | 1.90E-09 | 6.55E-09 |
| gene-BPHYT_RS27095 | 896.271615 | 1062.53355 | 716.786796 | 444.671199 | 494.655409 | 420.999786 | 0.97723023 | 1.96E-09 | 6.76E-09 |
| gene-BPHYT_RS15530 | 144.456957 | 151.485707 | 194.193302 | 338.186744 | 343.891418 | 340.421332 | -1.06345051 | 1.96E-09 | 6.78E-09 |
| gene-BPHYT_RS10565 | 213.892069 | 148.285304 | 199.276896 | 70.9896371 | 59.8072031 | 76.9569501 | 1.43075069 | 2.04E-09 | 7.03E-09 |
| gene-BPHYT_RS06225 | 304.876008 | 305.105015 | 329.41691 | 153.81088 | 150.763991 | 162.062283 | 1.00749242 | 2.12E-09 | 7.32E-09 |
| gene-BPHYT_RS34240 | 93.3782538 | 55.473639 | 92.5214162 | 237.618091 | 199.357344 | 206.425701 | -1.40840431 | 2.21E-09 | 7.61E-09 |
| gene-BPHYT_RS05240 | 109.340348 | 147.218504 | 141.323921 | 292.832253 | 348.875352 | 262.559006 | -1.18670942 | 2.33E-09 | 8.00E-09 |
| gene-BPHYT_RS18885 | 488.440097 | 591.007616 | 483.958177 | 865.679186 | 1014.23049 | 901.754379 | -0.83157061 | 2.34E-09 | 8.04E-09 |
| gene-BPHYT_RS14280 | 532.335857 | 812.902172 | 677.13476 | 293.81822 | 355.105269 | 329.556821 | 1.04693109 | 2.41E-09 | 8.29E-09 |
| gene-BPHYT_RS06165 | 934.580643 | 1017.72792 | 953.68229 | 1660.36874 | 1478.98229 | 1521.03148 | -0.68230319 | 2.57E-09 | 8.82E-09 |
| gene-BPHYT_RS26745 | 238.633315 | 238.963368 | 247.062683 | 444.671199 | 418.650422 | 472.606211 | -0.88418 | 2.67E-09 | 9.18E-09 |
| gene-BPHYT_RS37135 | 1464.52219 | 1215.08606 | 991.300888 | 661.583979 | 662.863168 | 631.04699 | 0.90994153 | 2.72E-09 | 9.33E-09 |
| gene-BPHYT_RS19700 | 360.743339 | 394.716278 | 419.904889 | 669.471717 | 847.268711 | 668.167402 | -0.89373794 | 2.87E-09 | 9.85E-09 |
| gene-BPHYT_RS06990 | 531.537752 | 681.685679 | 725.937266 | 361.849956 | 323.955684 | 312.35468 | 0.95622287 | 2.93E-09 | 1.00E-08 |
| gene-BPHYT_RS06265 | 564.260046 | 571.805202 | 531.743963 | 850.889678 | 984.326885 | 989.57584 | -0.75972165 | 2.97E-09 | 1.02E-08 |
| gene-BPHYT_RS24005 | 409.427728 | 443.789112 | 443.289423 | 245.505828 | 226.768979 | 181.980553 | 0.98841895 | 2.98E-09 | 1.02E-08 |
| gene-BPHYT_RS11340 | 47.0881793 | 58.6740413 | 37.6185978 | 4.92983591 | 4.98393359 | 5.4322553 | 3.21912132 | 3.05E-09 | 1.04E-08 |
| gene-BPHYT_RS34825 | 5056.79159 | 5104.64159 | 5522.81684 | 3483.42206 | 3575.97235 | 3323.63487 | 0.59523656 | 3.06E-09 | 1.04E-08 |
| gene-BPHYT_RS24595 | 245.018153 | 267.766988 | 274.514092 | 113.386226 | 125.844323 | 130.374127 | 1.08966781 | 3.17E-09 | 1.08E-08 |
| gene-BPHYT_RS16995 | 2111.78512 | 2468.57694 | 2062.92257 | 1450.35773 | 1221.06373 | 1305.55202 | 0.73930722 | 3.19E-09 | 1.09E-08 |
| gene-BPHYT_RS12425 | 3040.77903 | 3474.57004 | 3667.30493 | 5437.60901 | 5159.61725 | 5395.13489 | -0.65175358 | 3.25E-09 | 1.11E-08 |
| gene-BPHYT_RS18880 | 1449.3582 | 1425.2458 | 1131.60809 | 2169.1278 | 2875.72968 | 2275.20959 | -0.86860357 | 3.41E-09 | 1.16E-08 |
| gene-BPHYT_RS18875 | 1593.01705 | 1594.86712 | 1675.55268 | 2531.96372 | 2934.2909 | 2394.71921 | -0.69211078 | 3.61E-09 | 1.23E-08 |
| gene-BPHYT_RS08910 | 1720.71381 | 1910.64014 | 1893.13052 | 2946.06994 | 3028.98564 | 2684.43949 | -0.64857121 | 3.63E-09 | 1.24E-08 |
| gene-BPHYT_RS03105 | 191.545136 | 171.754921 | 252.146277 | 73.9475387 | 84.7268711 | 83.2945812 | 1.3477358 | 3.67E-09 | 1.25E-08 |
| gene-BPHYT_RS28910 | 124.504338 | 99.2124698 | 117.939388 | 250.435664 | 281.592248 | 229.965474 | -1.15071242 | 3.72E-09 | 1.27E-08 |
| gene-BPHYT_RS16335 | 687.96628 | 594.208018 | 702.552732 | 1110.19905 | 1065.31581 | 1116.32846 | -0.72942807 | 3.95E-09 | 1.34E-08 |
| gene-BPHYT_RS15790 | 646.464834 | 611.27683 | 435.155672 | 214.940846 | 286.576182 | 283.382651 | 1.111078 | 3.95E-09 | 1.34E-08 |
| gene-BPHYT_RS22605 | 1021.57406 | 1059.33315 | 928.264319 | 469.320379 | 482.195575 | 651.870636 | 0.90607795 | 3.95E-09 | 1.34E-08 |
| gene-BPHYT_RS13860 | 723.082888 | 764.896138 | 673.067885 | 1102.31131 | 1214.83381 | 1232.21658 | -0.71582771 | 3.99E-09 | 1.35E-08 |
| Novel00858 | 229.056058 | 252.831778 | 190.126427 | 89.7230136 | 103.416622 | 95.0644677 | 1.22420403 | 4.02E-09 | 1.36E-08 |
| gene-BPHYT_RS14940 | 1701.55929 | 1738.88522 | 1769.09082 | 2987.48056 | 2705.02996 | 2514.22883 | -0.65571918 | 4.06E-09 | 1.37E-08 |
| gene-BPHYT_RS22175 | 320.039998 | 334.975436 | 268.413779 | 151.838946 | 155.747925 | 133.995631 | 1.06745996 | 4.13E-09 | 1.40E-08 |
| gene-BPHYT_RS32785 | 114.128977 | 115.214481 | 168.775331 | 34.5088514 | 44.8554023 | 45.2687942 | 1.67576129 | 4.18E-09 | 1.41E-08 |
| gene-BPHYT_RS19075 | 17923.8361 | 21827.8102 | 23509.5902 | 13765.0878 | 12260.4766 | 11648.5661 | 0.7476787 | 4.19E-09 | 1.41E-08 |
| gene-BPHYT_RS05555 | 407.831519 | 394.716278 | 406.687544 | 717.784109 | 772.509707 | 631.952366 | -0.80952529 | 4.20E-09 | 1.42E-08 |
| gene-BPHYT_RS32610 | 139.668328 | 97.0788683 | 179.959238 | 316.495466 | 330.185601 | 348.569715 | -1.25470806 | 4.23E-09 | 1.43E-08 |
| gene-BPHYT_RS10300 | 719.092365 | 950.519469 | 745.254924 | 1336.9715 | 1492.68811 | 1411.481 | -0.81352725 | 4.24E-09 | 1.43E-08 |
| gene-BPHYT_RS09595 | 2733.50871 | 2534.71858 | 2273.38337 | 3820.62283 | 4056.92195 | 3966.45174 | -0.65071419 | 4.24E-09 | 1.43E-08 |
| gene-BPHYT_RS08805 | 478.064735 | 639.013649 | 895.729315 | 1430.63838 | 1355.62994 | 1541.85513 | -1.10649671 | 4.31E-09 | 1.45E-08 |
| gene-BPHYT_RS23110 | 4257.88875 | 4140.25371 | 3264.68426 | 2285.47193 | 2434.65156 | 2230.84618 | 0.74710411 | 4.41E-09 | 1.49E-08 |
| gene-BPHYT_RS33755 | 1011.9968 | 672.084473 | 699.502575 | 1472.049 | 1487.70418 | 1629.67659 | -0.94322677 | 4.53E-09 | 1.52E-08 |
| gene-BPHYT_RS26385 | 432.572765 | 473.659533 | 344.667693 | 162.684585 | 206.833244 | 218.195588 | 1.09028415 | 4.75E-09 | 1.60E-08 |
| gene-BPHYT_RS33850 | 2749.47081 | 3143.86181 | 3796.42822 | 1979.8221 | 1718.21111 | 1858.73669 | 0.80150481 | 4.79E-09 | 1.61E-08 |
| gene-BPHYT_RS20610 | 387.8789 | 362.712255 | 281.631124 | 580.73467 | 698.996687 | 761.421118 | -0.98202096 | 4.85E-09 | 1.63E-08 |
| gene-BPHYT_RS24055 | 748.62224 | 609.143228 | 511.409586 | 285.930483 | 306.511916 | 341.326708 | 1.00216448 | 4.88E-09 | 1.64E-08 |
| gene-BPHYT_RS08455 | 8064.84833 | 8689.09211 | 9233.84068 | 12604.6045 | 13228.6057 | 13171.4084 | -0.5859106 | 5.01E-09 | 1.68E-08 |
| gene-BPHYT_RS22240 | 85.3972065 | 71.4756503 | 112.855793 | 222.828583 | 225.522995 | 206.425701 | -1.27881038 | 5.10E-09 | 1.71E-08 |
| gene-BPHYT_RS29985 | 127.696757 | 139.750898 | 160.64158 | 56.2001294 | 32.3955684 | 52.5118012 | 1.58502114 | 5.19E-09 | 1.74E-08 |
| gene-BPHYT_RS22145 | 72.6275307 | 53.3400375 | 83.3709465 | 16.7614421 | 12.459834 | 12.6752624 | 2.31836656 | 5.24E-09 | 1.75E-08 |
| gene-BPHYT_RS35660 | 151.639899 | 134.416895 | 127.089857 | 323.397236 | 251.688647 | 305.111673 | -1.09083128 | 5.56E-09 | 1.86E-08 |
| gene-BPHYT_RS32725 | 698.341642 | 645.414454 | 723.903828 | 419.036053 | 370.057069 | 409.229899 | 0.78550699 | 5.66E-09 | 1.89E-08 |
| gene-BPHYT_RS22235 | 312.857055 | 278.434996 | 306.032377 | 160.712651 | 143.288091 | 112.26661 | 1.11198805 | 6.08E-09 | 2.03E-08 |
| gene-BPHYT_RS33550 | 1056.69067 | 1140.41 | 1308.51717 | 2062.64335 | 2073.31638 | 1761.86147 | -0.75072194 | 6.13E-09 | 2.05E-08 |
| gene-BPHYT_RS34370 | 1591.42084 | 1633.27195 | 1302.41686 | 2669.01316 | 2331.23494 | 2518.75571 | -0.73204569 | 6.13E-09 | 2.05E-08 |
| gene-BPHYT_RS22695 | 1525.97625 | 1429.51301 | 1582.01455 | 2511.25841 | 2322.51306 | 2235.37306 | -0.63945712 | 6.14E-09 | 2.05E-08 |
| gene-BPHYT_RS05560 | 2120.56428 | 2054.65825 | 1839.24442 | 3292.14442 | 3031.47761 | 3039.34684 | -0.63831026 | 6.18E-09 | 2.06E-08 |
| gene-BPHYT_RS17955 | 8485.44952 | 9052.87117 | 8887.13955 | 6189.90197 | 5979.47433 | 5892.18625 | 0.54892497 | 6.20E-09 | 2.07E-08 |
| gene-BPHYT_RS38845 | 82.2047875 | 224.028158 | 233.845338 | 1078.6481 | 1132.59891 | 1028.507 | -2.58701575 | 6.36E-09 | 2.12E-08 |
| gene-BPHYT_RS18870 | 1408.65485 | 1322.83293 | 1235.31341 | 2110.95574 | 2970.42442 | 2134.87633 | -0.86206156 | 6.51E-09 | 2.17E-08 |
| gene-BPHYT_RS05765 | 2.3943142 | 12.801609 | 4.06687544 | 69.0177028 | 38.6254854 | 60.6601842 | -3.18489534 | 6.56E-09 | 2.18E-08 |
| gene-BPHYT_RS12205 | 1355.18184 | 1535.12628 | 1717.23815 | 2463.93199 | 2468.29311 | 2528.71484 | -0.69619427 | 6.57E-09 | 2.19E-08 |
| gene-BPHYT_RS10380 | 68.6370071 | 40.5384285 | 29.4848469 | 4.92983591 | 1.2459834 | 3.62150353 | 3.78172359 | 6.58E-09 | 2.19E-08 |
| gene-BPHYT_RS31075 | 0 | 1.06680075 | 0 | 52.2562607 | 59.8072031 | 42.5526665 | -7.23388554 | 6.58E-09 | 2.19E-08 |
| gene-BPHYT_RS26780 | 250.604886 | 248.564575 | 253.162996 | 547.211786 | 520.821061 | 400.17614 | -0.96296342 | 6.68E-09 | 2.21E-08 |
| gene-BPHYT_RS23935 | 1848.41056 | 1976.78179 | 1783.32488 | 1113.15695 | 1207.35791 | 1246.70259 | 0.6526658 | 6.74E-09 | 2.24E-08 |
| gene-BPHYT_RS12440 | 3205.18861 | 4140.25371 | 3964.18683 | 5906.92939 | 6180.07766 | 6353.02257 | -0.70574792 | 6.78E-09 | 2.25E-08 |
| gene-BPHYT_RS15800 | 528.345333 | 466.191928 | 525.64365 | 928.781086 | 829.824943 | 815.743671 | -0.7591735 | 7.18E-09 | 2.38E-08 |
| gene-BPHYT_RS01645 | 2324.08098 | 2721.40871 | 2130.02601 | 3708.22257 | 4129.18898 | 3870.4819 | -0.70638907 | 7.21E-09 | 2.39E-08 |
| gene-BPHYT_RS14495 | 152.438004 | 174.955323 | 161.658299 | 47.3264248 | 79.7429375 | 49.7956736 | 1.48172762 | 7.40E-09 | 2.45E-08 |
| gene-BPHYT_RS21230 | 1374.33635 | 1380.44017 | 1255.64779 | 2005.45725 | 2383.56624 | 2059.73013 | -0.68439802 | 7.43E-09 | 2.46E-08 |
| gene-BPHYT_RS28075 | 112.532767 | 81.076857 | 84.3876653 | 250.435664 | 203.095294 | 210.952581 | -1.25072969 | 7.70E-09 | 2.54E-08 |
| gene-BPHYT_RS19975 | 320.039998 | 313.639421 | 321.28316 | 527.492443 | 545.740729 | 580.345941 | -0.7923328 | 8.08E-09 | 2.67E-08 |
| gene-BPHYT_RS38760 | 55.0692266 | 44.8056315 | 56.9362561 | 7.88773746 | 2.4919668 | 9.95913471 | 2.89561318 | 8.12E-09 | 2.68E-08 |
| gene-BPHYT_RS29530 | 2543.55979 | 2546.45339 | 2545.86402 | 3572.1591 | 3889.96017 | 4002.66678 | -0.58633472 | 8.33E-09 | 2.75E-08 |
| gene-BPHYT_RS28340 | 118.917605 | 105.613274 | 79.304071 | 12.8175734 | 27.4116348 | 32.5935318 | 2.06349356 | 8.34E-09 | 2.75E-08 |
| gene-BPHYT_RS29950 | 604.165283 | 572.872003 | 550.044903 | 320.439334 | 342.645435 | 337.705204 | 0.78851801 | 8.41E-09 | 2.77E-08 |
| gene-BPHYT_RS03870 | 694.351118 | 708.355698 | 476.841145 | 297.762089 | 345.137401 | 269.802013 | 1.04553779 | 9.34E-09 | 3.07E-08 |
| gene-BPHYT_RS15220 | 464.496955 | 501.396353 | 495.142085 | 243.533894 | 292.806099 | 271.612765 | 0.85612215 | 9.37E-09 | 3.08E-08 |
| gene-BPHYT_RS11585 | 2308.91699 | 2458.97573 | 2053.7721 | 1494.72625 | 1437.86484 | 1341.76706 | 0.6746395 | 9.51E-09 | 3.13E-08 |
| gene-BPHYT_RS21480 | 309.664637 | 237.896567 | 212.494242 | 86.765112 | 124.59834 | 104.118227 | 1.27715898 | 9.90E-09 | 3.25E-08 |
| gene-BPHYT_RS07595 | 216.286383 | 233.629364 | 307.049096 | 74.9335059 | 128.33629 | 93.253716 | 1.35955878 | 1.00E-08 | 3.28E-08 |
| gene-BPHYT_RS13020 | 1269.78463 | 1375.10617 | 757.45555 | 517.632771 | 559.446546 | 507.91587 | 1.10291526 | 1.05E-08 | 3.43E-08 |
| gene-BPHYT_RS20310 | 122.908129 | 170.68812 | 111.839075 | 306.635794 | 266.640447 | 338.61058 | -1.17528598 | 1.06E-08 | 3.47E-08 |
| gene-BPHYT_RS20755 | 341.588826 | 285.902601 | 288.748156 | 157.754749 | 137.058174 | 155.724652 | 1.02306496 | 1.06E-08 | 3.47E-08 |
| gene-BPHYT_RS20320 | 106.946034 | 86.4108608 | 93.5381351 | 20.7053108 | 24.919668 | 34.4042836 | 1.83618004 | 1.07E-08 | 3.52E-08 |
| gene-BPHYT_RS14290 | 1083.82623 | 975.055886 | 769.656177 | 567.917097 | 429.864272 | 477.13309 | 0.9388116 | 1.07E-08 | 3.52E-08 |
| gene-BPHYT_RS06640 | 8467.89122 | 9489.19267 | 8809.86892 | 13073.9248 | 12949.5055 | 13276.432 | -0.55420738 | 1.07E-08 | 3.52E-08 |
| gene-BPHYT_RS05770 | 14.3658852 | 12.801609 | 9.15046973 | 73.9475387 | 39.8714688 | 96.8752195 | -2.53603723 | 1.08E-08 | 3.52E-08 |
| gene-BPHYT_RS03490 | 826.836504 | 842.772593 | 888.612283 | 1443.45596 | 1360.61387 | 1262.99936 | -0.66895474 | 1.09E-08 | 3.56E-08 |
| gene-BPHYT_RS29040 | 164.409575 | 214.426951 | 191.143146 | 410.162348 | 360.089202 | 353.096594 | -0.98325706 | 1.10E-08 | 3.60E-08 |
| gene-BPHYT_RS14615 | 4017.65923 | 3994.10201 | 3744.57556 | 2528.01986 | 2700.04602 | 2676.29111 | 0.57292425 | 1.11E-08 | 3.63E-08 |
| gene-BPHYT_RS17015 | 6586.75836 | 9126.48042 | 10041.1155 | 15715.3309 | 14839.6623 | 15309.0008 | -0.83275913 | 1.12E-08 | 3.65E-08 |
| gene-BPHYT_RS29990 | 189.150822 | 164.287316 | 173.858925 | 63.1018997 | 76.0049873 | 79.6730777 | 1.27129906 | 1.13E-08 | 3.69E-08 |
| gene-BPHYT_RS23565 | 112.532767 | 100.279271 | 97.6050105 | 22.6772452 | 32.3955684 | 38.0257871 | 1.73741814 | 1.17E-08 | 3.80E-08 |
| gene-BPHYT_RS19605 | 695.149223 | 722.224108 | 934.364632 | 1392.18566 | 1353.13797 | 1329.99717 | -0.7940804 | 1.19E-08 | 3.88E-08 |
| gene-BPHYT_RS18810 | 422.995509 | 433.121105 | 413.804576 | 690.177028 | 858.482562 | 686.274919 | -0.81352373 | 1.21E-08 | 3.93E-08 |
| gene-BPHYT_RS06295 | 517.171867 | 505.663556 | 418.88817 | 802.577286 | 807.397242 | 858.296337 | -0.77503429 | 1.24E-08 | 4.04E-08 |
| gene-BPHYT_RS07630 | 565.856256 | 508.863958 | 385.336448 | 242.547927 | 235.490862 | 252.599871 | 0.9994024 | 1.25E-08 | 4.07E-08 |
| gene-BPHYT_RS02500 | 87.7915207 | 106.680075 | 48.8025053 | 220.856649 | 223.031028 | 217.290212 | -1.4418404 | 1.30E-08 | 4.22E-08 |
| gene-BPHYT_RS19600 | 2046.34054 | 2198.67635 | 2522.47949 | 3600.75215 | 3522.39507 | 3492.03478 | -0.64986695 | 1.32E-08 | 4.29E-08 |
| gene-BPHYT_RS31090 | 0 | 7.46760525 | 8.13375087 | 155.782815 | 183.15956 | 193.750439 | -5.12824993 | 1.38E-08 | 4.49E-08 |
| gene-BPHYT_RS34390 | 873.924683 | 818.236175 | 587.663501 | 387.485103 | 348.875352 | 424.621289 | 0.97259841 | 1.39E-08 | 4.50E-08 |
| gene-BPHYT_RS07070 | 166.005785 | 140.817699 | 140.307203 | 284.944516 | 289.068148 | 328.651446 | -1.01108417 | 1.41E-08 | 4.56E-08 |
| gene-BPHYT_RS03120 | 794.912314 | 709.422499 | 867.261187 | 501.857296 | 414.912472 | 430.95892 | 0.81447936 | 1.42E-08 | 4.59E-08 |
| gene-BPHYT_RS01620 | 706.322689 | 825.703781 | 856.07728 | 1239.36075 | 1294.57675 | 1403.33262 | -0.7229788 | 1.43E-08 | 4.62E-08 |
| gene-BPHYT_RS09450 | 8317.04943 | 8714.69533 | 7338.67673 | 12256.558 | 12591.9082 | 12016.1487 | -0.59705359 | 1.43E-08 | 4.62E-08 |
| gene-BPHYT_RS17385 | 2265.81934 | 2444.04052 | 2180.86195 | 1571.63169 | 1476.49033 | 1441.35841 | 0.61793834 | 1.50E-08 | 4.86E-08 |
| gene-BPHYT_RS08900 | 9804.71665 | 10377.8377 | 8727.51469 | 13811.4283 | 15022.8218 | 15812.3898 | -0.62698924 | 1.56E-08 | 5.05E-08 |
| gene-BPHYT_RS04500 | 273.749924 | 297.637409 | 254.179715 | 136.063471 | 103.416622 | 143.954765 | 1.09864603 | 1.57E-08 | 5.05E-08 |
| gene-BPHYT_RS08945 | 1551.5156 | 1640.73955 | 1629.80033 | 990.897018 | 1057.83991 | 1084.64031 | 0.62163132 | 1.65E-08 | 5.33E-08 |
| gene-BPHYT_RS20090 | 66.2426929 | 81.076857 | 50.835943 | 188.319732 | 164.469809 | 156.630028 | -1.36393121 | 1.70E-08 | 5.47E-08 |
| gene-BPHYT_RS18180 | 5559.59757 | 6916.06926 | 6998.07591 | 10681.9685 | 10367.8279 | 9889.42077 | -0.66813848 | 1.70E-08 | 5.49E-08 |
| gene-BPHYT_RS22425 | 87.7915207 | 83.2104585 | 93.5381351 | 181.417962 | 229.260945 | 191.939687 | -1.18421476 | 1.71E-08 | 5.52E-08 |
| gene-BPHYT_RS05480 | 2346.42792 | 2835.55639 | 2616.01763 | 3950.7705 | 4387.10755 | 3908.50769 | -0.65139386 | 1.71E-08 | 5.52E-08 |
| gene-BPHYT_RS06975 | 650.455358 | 700.888093 | 820.492119 | 432.839593 | 365.073136 | 418.283658 | 0.83360323 | 1.72E-08 | 5.55E-08 |
| gene-BPHYT_RS29475 | 43.0976556 | 69.3420488 | 49.8192241 | 143.951209 | 142.042107 | 151.197773 | -1.44089569 | 1.73E-08 | 5.56E-08 |
| gene-BPHYT_RS22100 | 548.297952 | 485.394341 | 433.122234 | 238.604058 | 282.838231 | 266.18051 | 0.90038643 | 1.73E-08 | 5.57E-08 |
| gene-BPHYT_RS14315 | 482.853364 | 396.849879 | 437.18911 | 738.48942 | 736.376188 | 751.461983 | -0.755479 | 1.76E-08 | 5.65E-08 |
| gene-BPHYT_RS20210 | 26.3374562 | 33.0708233 | 40.6687544 | 102.540587 | 98.4326885 | 111.361234 | -1.65336045 | 1.87E-08 | 6.00E-08 |
| gene-BPHYT_RS14360 | 867.539845 | 952.65307 | 1019.76902 | 542.28195 | 550.724662 | 612.939473 | 0.73353051 | 1.89E-08 | 6.07E-08 |
| gene-BPHYT_RS12520 | 2745.48028 | 2676.60308 | 3360.25583 | 4739.54425 | 4496.75409 | 4740.54812 | -0.67061663 | 1.91E-08 | 6.11E-08 |
| gene-BPHYT_RS23820 | 154.034214 | 118.414883 | 156.574704 | 64.0878669 | 36.1335186 | 45.2687942 | 1.55289704 | 2.04E-08 | 6.54E-08 |
| gene-BPHYT_RS17755 | 1453.34872 | 1523.39147 | 1664.36877 | 1028.36377 | 986.818852 | 892.700621 | 0.67463522 | 2.05E-08 | 6.56E-08 |
| gene-BPHYT_RS34710 | 381.494063 | 428.853902 | 347.71785 | 208.039076 | 166.961775 | 213.668708 | 0.97093004 | 2.13E-08 | 6.83E-08 |
| gene-BPHYT_RS24105 | 2936.22731 | 2723.54232 | 2247.9654 | 1379.36809 | 1758.08258 | 1440.45303 | 0.78982752 | 2.26E-08 | 7.23E-08 |
| gene-BPHYT_RS16955 | 315.25137 | 317.906624 | 235.878775 | 539.324049 | 502.13131 | 613.844849 | -0.92987012 | 2.30E-08 | 7.36E-08 |
| gene-BPHYT_RS24110 | 71.829426 | 69.3420488 | 122.006263 | 22.6772452 | 19.9357344 | 21.7290212 | 2.02642326 | 2.34E-08 | 7.46E-08 |
| gene-BPHYT_RS22510 | 77.4161591 | 86.4108608 | 55.9195373 | 215.926813 | 216.801111 | 147.576269 | -1.39735861 | 2.34E-08 | 7.48E-08 |
| gene-BPHYT_RS37290 | 138.870224 | 118.414883 | 85.4043842 | 39.4386873 | 36.1335186 | 31.6881559 | 1.68150993 | 2.42E-08 | 7.72E-08 |
| gene-BPHYT_RS23920 | 378.301644 | 414.985492 | 502.259117 | 210.996977 | 194.37341 | 248.072992 | 0.98246503 | 2.45E-08 | 7.82E-08 |
| gene-BPHYT_RS23735 | 260.182143 | 282.702199 | 361.951914 | 136.063471 | 139.550141 | 152.103148 | 1.07796823 | 2.45E-08 | 7.82E-08 |
| gene-BPHYT_RS15830 | 4010.47628 | 4217.06337 | 2976.95282 | 2182.93134 | 2070.82441 | 2266.15584 | 0.78099908 | 2.53E-08 | 8.05E-08 |
| gene-BPHYT_RS16430 | 3917.89614 | 4344.01266 | 4140.0792 | 5877.35037 | 6173.84774 | 6114.90871 | -0.55085975 | 2.53E-08 | 8.06E-08 |
| gene-BPHYT_RS32760 | 149.245585 | 96.0120675 | 136.240327 | 317.481433 | 260.41053 | 272.518141 | -1.15169333 | 2.56E-08 | 8.15E-08 |
| gene-BPHYT_RS07050 | 107.744139 | 250.698176 | 162.675018 | 51.2702935 | 36.1335186 | 61.56556 | 1.79189961 | 2.61E-08 | 8.31E-08 |
| gene-BPHYT_RS19325 | 3321.7119 | 3288.94671 | 3484.29553 | 4647.8493 | 5410.05992 | 5119.90062 | -0.58815761 | 2.62E-08 | 8.33E-08 |
| gene-BPHYT_RS14885 | 62303.2479 | 85580.8898 | 102081.624 | 154304.85 | 156273.73 | 144499.802 | -0.86440134 | 2.66E-08 | 8.45E-08 |
| gene-BPHYT_RS04495 | 446.140546 | 388.315473 | 379.236135 | 232.688255 | 206.833244 | 153.008524 | 1.03850833 | 2.67E-08 | 8.47E-08 |
| gene-BPHYT_RS08245 | 321.636208 | 288.036203 | 267.39706 | 529.464377 | 500.885326 | 511.537374 | -0.81195125 | 2.71E-08 | 8.58E-08 |
| gene-BPHYT_RS28285 | 413.418252 | 401.117082 | 442.272704 | 209.025043 | 260.41053 | 198.277318 | 0.91783236 | 2.72E-08 | 8.63E-08 |
| gene-BPHYT_RS07000 | 17486.4747 | 18869.5717 | 16492.1966 | 25484.2938 | 26831.0065 | 25304.3506 | -0.55455661 | 2.75E-08 | 8.71E-08 |
| gene-BPHYT_RS09560 | 8992.24603 | 5269.99571 | 4357.65703 | 1690.93372 | 1846.5474 | 1594.36693 | 1.85948617 | 2.82E-08 | 8.92E-08 |
| gene-BPHYT_RS29960 | 2190.79749 | 2154.93752 | 2023.27053 | 1436.55419 | 1239.75348 | 1427.77777 | 0.63314103 | 2.84E-08 | 8.97E-08 |
| gene-BPHYT_RS20490 | 185.160298 | 206.959346 | 164.708455 | 44.3685232 | 80.9889209 | 81.4838295 | 1.43206248 | 2.84E-08 | 8.98E-08 |
| gene-BPHYT_RS17205 | 6313.80654 | 5747.92244 | 6179.61723 | 8693.27265 | 8730.60567 | 9018.44917 | -0.53554146 | 2.86E-08 | 9.04E-08 |
| gene-BPHYT_RS29735 | 110.138453 | 90.6780638 | 90.4879785 | 29.5790155 | 18.689751 | 35.3096594 | 1.78403019 | 2.87E-08 | 9.08E-08 |
| gene-BPHYT_RS25790 | 168.400099 | 228.295361 | 302.98222 | 76.9054402 | 99.6786719 | 93.253716 | 1.37420605 | 2.90E-08 | 9.16E-08 |
| gene-BPHYT_RS35035 | 122.110024 | 142.951301 | 122.006263 | 57.1860966 | 33.6415518 | 42.5526665 | 1.52600423 | 2.91E-08 | 9.17E-08 |
| Novel00755 | 7830.20554 | 9533.99831 | 10820.9388 | 15650.2571 | 14521.9365 | 16371.0067 | -0.72384361 | 2.96E-08 | 9.33E-08 |
| gene-BPHYT_RS03150 | 5652.17772 | 6680.3063 | 5975.25674 | 9301.6144 | 9131.81233 | 8956.88361 | -0.58140854 | 2.97E-08 | 9.36E-08 |
| gene-BPHYT_RS29160 | 349.569873 | 416.052293 | 417.871451 | 214.940846 | 185.651526 | 221.817091 | 0.92177191 | 2.97E-08 | 9.36E-08 |
| gene-BPHYT_RS06725 | 1652.0768 | 1354.83695 | 1179.39388 | 821.310663 | 809.889209 | 788.582394 | 0.79191501 | 3.00E-08 | 9.45E-08 |
| gene-BPHYT_RS12420 | 4192.44416 | 4642.71687 | 4707.40832 | 7062.48293 | 6770.67379 | 6371.13009 | -0.5773386 | 3.02E-08 | 9.51E-08 |
| gene-BPHYT_RS15110 | 80.6085781 | 58.6740413 | 64.0532881 | 169.586355 | 166.961775 | 158.44078 | -1.27475404 | 3.04E-08 | 9.57E-08 |
| gene-BPHYT_RS26040 | 82.2047875 | 68.275248 | 36.6018789 | 6.90177028 | 2.4919668 | 14.4860141 | 2.91993456 | 3.11E-08 | 9.76E-08 |
| gene-BPHYT_RS04725 | 3424.66741 | 3633.52336 | 6966.55762 | 17181.4641 | 18541.479 | 19966.2544 | -1.98947718 | 3.11E-08 | 9.77E-08 |
| gene-BPHYT_RS15810 | 422.995509 | 304.038214 | 329.41691 | 678.345421 | 585.612197 | 722.489955 | -0.9089133 | 3.36E-08 | 1.05E-07 |
| gene-BPHYT_RS19525 | 351.964187 | 326.44103 | 259.263309 | 150.852979 | 160.731858 | 151.197773 | 1.02190567 | 3.50E-08 | 1.10E-07 |
| gene-BPHYT_RS26920 | 297.693066 | 285.902601 | 306.032377 | 136.063471 | 165.715792 | 155.724652 | 0.96226971 | 3.52E-08 | 1.10E-07 |
| gene-BPHYT_RS27145 | 52.6749124 | 66.1416465 | 105.738761 | 9.85967182 | 12.459834 | 20.8236453 | 2.36153143 | 3.67E-08 | 1.15E-07 |
| gene-BPHYT_RS29915 | 581.020246 | 682.75248 | 680.184917 | 1016.53217 | 1167.48644 | 1022.16937 | -0.7223478 | 3.68E-08 | 1.15E-07 |
| gene-BPHYT_RS07465 | 48.6843887 | 59.740842 | 97.6050105 | 153.81088 | 210.571194 | 221.817091 | -1.51516387 | 3.77E-08 | 1.18E-07 |
| gene-BPHYT_RS26320 | 968.899146 | 1273.7601 | 1036.03652 | 625.103194 | 569.414413 | 685.369543 | 0.79997555 | 3.98E-08 | 1.24E-07 |
| gene-BPHYT_RS13975 | 244.220048 | 258.165782 | 347.71785 | 148.881045 | 120.86039 | 99.5913471 | 1.2025366 | 4.15E-08 | 1.30E-07 |
| gene-BPHYT_RS13930 | 104.55172 | 92.8116653 | 109.805637 | 21.691278 | 24.919668 | 42.5526665 | 1.77119967 | 4.18E-08 | 1.30E-07 |
| gene-BPHYT_RS35115 | 781.344534 | 669.950871 | 758.472269 | 441.713298 | 468.489758 | 409.229899 | 0.74663957 | 4.37E-08 | 1.36E-07 |
| gene-BPHYT_RS17510 | 3611.42392 | 4333.34465 | 3945.88589 | 2763.66601 | 2454.5873 | 2419.16436 | 0.63827007 | 4.43E-08 | 1.38E-07 |
| gene-BPHYT_RS16105 | 318.443789 | 318.973424 | 280.614405 | 91.694948 | 173.191692 | 134.901007 | 1.20688626 | 4.52E-08 | 1.41E-07 |
| gene-BPHYT_RS15490 | 686.370071 | 907.847438 | 569.362561 | 1194.99223 | 1399.23936 | 1588.0293 | -0.95173565 | 4.54E-08 | 1.41E-07 |
| gene-BPHYT_RS07095 | 119.71571 | 85.34406 | 122.006263 | 212.968911 | 239.228813 | 317.786935 | -1.23361377 | 4.64E-08 | 1.44E-07 |
| gene-BPHYT_RS01800 | 5196.45992 | 6191.71155 | 6588.33821 | 9542.19039 | 9473.21178 | 8937.87072 | -0.63711539 | 4.64E-08 | 1.45E-07 |
| gene-BPHYT_RS14650 | 937.773062 | 836.371788 | 799.141023 | 1474.02094 | 1263.42717 | 1397.90036 | -0.68404183 | 4.66E-08 | 1.45E-07 |
| gene-BPHYT_RS09180 | 6644.2219 | 8053.27886 | 8177.46979 | 11204.5311 | 12109.7127 | 11963.6369 | -0.62517297 | 4.76E-08 | 1.48E-07 |
| gene-BPHYT_RS33235 | 137.274014 | 98.145669 | 130.140014 | 53.2422278 | 31.149585 | 32.5935318 | 1.64056751 | 4.80E-08 | 1.49E-07 |
| gene-BPHYT_RS27025 | 114.128977 | 92.8116653 | 95.5715728 | 36.4807857 | 34.8875352 | 19.0128935 | 1.75853303 | 4.82E-08 | 1.50E-07 |
| gene-BPHYT_RS19690 | 276.144238 | 300.837812 | 319.249722 | 119.302029 | 159.485875 | 161.156907 | 1.02700339 | 4.85E-08 | 1.51E-07 |
| gene-BPHYT_RS03030 | 83.800997 | 92.8116653 | 94.5548539 | 32.536917 | 21.1817178 | 28.0666524 | 1.71596009 | 4.97E-08 | 1.54E-07 |
| gene-BPHYT_RS02610 | 779.748324 | 787.298954 | 1068.57152 | 1556.84218 | 1422.91304 | 1595.27231 | -0.79676453 | 5.02E-08 | 1.56E-07 |
| gene-BPHYT_RS05475 | 746.227926 | 889.711826 | 803.207899 | 483.123919 | 503.377293 | 498.862112 | 0.71470272 | 5.09E-08 | 1.58E-07 |
| gene-BPHYT_RS02240 | 1370.34583 | 1338.83494 | 1147.87559 | 1950.24309 | 2170.50308 | 1952.89578 | -0.65412275 | 5.25E-08 | 1.63E-07 |
| gene-BPHYT_RS06755 | 1023.96837 | 1166.01322 | 1158.04278 | 699.050732 | 659.125218 | 738.786721 | 0.67302404 | 5.47E-08 | 1.69E-07 |
| gene-BPHYT_RS05070 | 4023.24596 | 4132.78611 | 3097.94236 | 5881.29424 | 6440.48819 | 5987.25071 | -0.70190197 | 5.57E-08 | 1.72E-07 |
| gene-BPHYT_RS15395 | 2543.55979 | 2792.88436 | 3208.76472 | 4425.02071 | 4292.41281 | 4535.93317 | -0.63366162 | 5.86E-08 | 1.81E-07 |
| gene-BPHYT_RS14945 | 600.972864 | 760.628935 | 570.37928 | 1072.73229 | 1071.54572 | 1119.04459 | -0.7576902 | 6.12E-08 | 1.89E-07 |
| Novel00777 | 128.494862 | 117.348083 | 164.708455 | 272.126942 | 261.656514 | 331.367573 | -1.07840601 | 6.20E-08 | 1.92E-07 |
| gene-BPHYT_RS33510 | 219.478802 | 162.153714 | 145.390797 | 317.481433 | 446.062057 | 372.109488 | -1.10029245 | 6.31E-08 | 1.95E-07 |
| gene-BPHYT_RS13670 | 7920.39137 | 9024.06755 | 6421.59632 | 3950.7705 | 5160.86324 | 4384.7354 | 0.7921031 | 6.35E-08 | 1.96E-07 |
| gene-BPHYT_RS09690 | 3759.8714 | 5193.18605 | 3540.21507 | 2290.40176 | 2417.20779 | 2483.44605 | 0.79661652 | 6.40E-08 | 1.97E-07 |
| gene-BPHYT_RS09930 | 34.3185035 | 38.404827 | 44.7356298 | 129.161701 | 123.352356 | 90.5375883 | -1.54822962 | 6.50E-08 | 2.00E-07 |
| gene-BPHYT_RS14110 | 1828.45794 | 2129.3343 | 1725.3719 | 3098.89485 | 2838.35018 | 2910.78346 | -0.63914794 | 6.60E-08 | 2.03E-07 |
| gene-BPHYT_RS11515 | 1990.47321 | 2741.67793 | 2974.91938 | 1506.55785 | 1370.58174 | 1503.82934 | 0.81395844 | 6.62E-08 | 2.04E-07 |
| gene-BPHYT_RS26675 | 703.13027 | 620.878037 | 655.783664 | 397.344774 | 367.565103 | 421.905162 | 0.73720915 | 7.04E-08 | 2.17E-07 |
| gene-BPHYT_RS19715 | 43.0976556 | 51.206436 | 29.4848469 | 121.273963 | 119.614406 | 112.26661 | -1.51198611 | 7.16E-08 | 2.20E-07 |
| gene-BPHYT_RS03435 | 2431.02702 | 2369.36447 | 1607.43252 | 3616.52763 | 3831.39895 | 3852.37438 | -0.81810014 | 7.20E-08 | 2.21E-07 |
| gene-BPHYT_RS20095 | 150.841795 | 124.815688 | 149.457672 | 296.776122 | 254.180613 | 288.814907 | -0.98144562 | 7.23E-08 | 2.22E-07 |
| gene-BPHYT_RS03255 | 31.9241893 | 7.46760525 | 28.4681281 | 91.694948 | 110.892523 | 91.4429642 | -2.09239021 | 7.28E-08 | 2.23E-07 |
| gene-BPHYT_RS09915 | 68.6370071 | 112.014079 | 81.3375087 | 200.151338 | 206.833244 | 200.08807 | -1.22047656 | 7.37E-08 | 2.26E-07 |
| gene-BPHYT_RS10295 | 1649.68248 | 1901.03894 | 2123.9257 | 2879.02417 | 2990.36016 | 3066.50812 | -0.65577077 | 7.44E-08 | 2.28E-07 |
| gene-BPHYT_RS17160 | 151.639899 | 134.416895 | 156.574704 | 298.748056 | 299.036016 | 261.65363 | -0.95442124 | 7.53E-08 | 2.31E-07 |
| gene-BPHYT_RS13735 | 1213.11919 | 1116.94039 | 1336.9853 | 763.138599 | 770.01774 | 792.203898 | 0.65706457 | 7.64E-08 | 2.34E-07 |
| gene-BPHYT_RS06620 | 1875.54612 | 2281.88681 | 2697.35513 | 1357.67681 | 1415.43714 | 1163.40801 | 0.80033763 | 7.77E-08 | 2.38E-07 |
| gene-BPHYT_RS15190 | 227.459849 | 208.026146 | 230.795181 | 401.288643 | 456.029924 | 363.961105 | -0.87068115 | 7.80E-08 | 2.38E-07 |
| gene-BPHYT_RS15415 | 3500.48736 | 3602.58613 | 3414.14193 | 2392.94235 | 2460.81721 | 2460.81165 | 0.52391959 | 8.05E-08 | 2.46E-07 |
| gene-BPHYT_RS02900 | 50195.9991 | 42308.251 | 31018.059 | 21771.1414 | 23960.2608 | 23807.7642 | 0.82891328 | 8.07E-08 | 2.47E-07 |
| gene-BPHYT_RS20515 | 130.091072 | 149.352105 | 111.839075 | 46.3404576 | 13.7058174 | 46.17417 | 1.85641415 | 8.28E-08 | 2.53E-07 |
| gene-BPHYT_RS22615 | 874.722788 | 708.355698 | 596.81397 | 401.288643 | 422.388372 | 351.285843 | 0.89489453 | 8.67E-08 | 2.65E-07 |
| gene-BPHYT_RS17765 | 3761.46761 | 4042.10804 | 3861.49823 | 2669.99913 | 2697.55406 | 2743.28893 | 0.52406149 | 8.72E-08 | 2.66E-07 |
| gene-BPHYT_RS16345 | 31367.1122 | 40091.439 | 38418.7555 | 54542.7186 | 58304.5471 | 56735.3797 | -0.62613644 | 8.78E-08 | 2.67E-07 |
| gene-BPHYT_RS28950 | 3003.26811 | 3134.2606 | 3425.32584 | 2201.66472 | 2077.05433 | 2167.46986 | 0.56851928 | 8.85E-08 | 2.70E-07 |
| gene-BPHYT_RS06730 | 940.167376 | 874.776615 | 665.950853 | 452.558937 | 464.751808 | 481.65997 | 0.82723644 | 8.92E-08 | 2.72E-07 |
| gene-BPHYT_RS15840 | 23.943142 | 17.068812 | 9.15046973 | 68.0317356 | 71.0210537 | 75.1461983 | -2.07342827 | 9.17E-08 | 2.79E-07 |
| gene-BPHYT_RS15700 | 30.3279799 | 19.2024135 | 19.3176583 | 82.8212433 | 90.9567881 | 72.4300706 | -1.81814887 | 9.21E-08 | 2.80E-07 |
| gene-BPHYT_RS02955 | 2106.9965 | 2151.73711 | 2204.24649 | 3024.94732 | 3212.1452 | 3100.00702 | -0.53073534 | 9.28E-08 | 2.82E-07 |
| gene-BPHYT_RS32615 | 80.6085781 | 89.611263 | 94.5548539 | 206.067141 | 166.961775 | 205.520325 | -1.13412104 | 9.35E-08 | 2.84E-07 |
| gene-BPHYT_RS03600 | 312.058951 | 358.445052 | 375.169259 | 661.583979 | 544.494745 | 602.980338 | -0.79415977 | 9.52E-08 | 2.89E-07 |
| Novel00601 | 1268.18842 | 834.238187 | 697.469138 | 424.951856 | 386.254854 | 508.821246 | 1.08470513 | 9.91E-08 | 3.01E-07 |
| gene-BPHYT_RS30000 | 81.4066828 | 72.542451 | 59.9864127 | 22.6772452 | 14.9518008 | 17.2021418 | 1.95826524 | 9.98E-08 | 3.03E-07 |
| gene-BPHYT_RS01690 | 182.765984 | 234.696165 | 213.51096 | 408.190414 | 342.645435 | 468.984707 | -0.95710983 | 1.01E-07 | 3.07E-07 |
| gene-BPHYT_RS30860 | 105.349825 | 57.6072405 | 48.8025053 | 20.7053108 | 7.47590039 | 6.33763118 | 2.61373143 | 1.02E-07 | 3.10E-07 |
| gene-BPHYT_RS17920 | 613.74254 | 630.479243 | 447.356298 | 905.117873 | 1020.4604 | 1088.26181 | -0.83265965 | 1.08E-07 | 3.26E-07 |
| gene-BPHYT_RS01955 | 2013.61824 | 2162.40512 | 2105.62476 | 2942.12607 | 3063.87318 | 3088.23714 | -0.53409659 | 1.10E-07 | 3.34E-07 |
| gene-BPHYT_RS22675 | 242.623839 | 236.829767 | 346.701131 | 136.063471 | 115.876456 | 128.563375 | 1.11481173 | 1.10E-07 | 3.34E-07 |
| gene-BPHYT_RS08125 | 1459.73356 | 1947.97817 | 2119.85882 | 1084.5639 | 1106.43326 | 1023.98012 | 0.78135613 | 1.16E-07 | 3.50E-07 |
| Novel00353 | 23.1450373 | 12.801609 | 23.3845338 | 81.8352761 | 63.5451533 | 79.6730777 | -1.91509335 | 1.16E-07 | 3.51E-07 |
| gene-BPHYT_RS17880 | 736.650669 | 735.025717 | 606.981159 | 1030.33571 | 1207.35791 | 1168.84027 | -0.71161596 | 1.17E-07 | 3.52E-07 |
| gene-BPHYT_RS19885 | 268.961295 | 252.831778 | 230.795181 | 138.035406 | 95.9407217 | 124.941872 | 1.0625602 | 1.18E-07 | 3.56E-07 |
| gene-BPHYT_RS09075 | 880.309521 | 1141.4768 | 1208.87872 | 1684.03195 | 1832.84158 | 1871.41195 | -0.73931631 | 1.21E-07 | 3.63E-07 |
| gene-BPHYT_RS38880 | 191.545136 | 141.8845 | 193.176583 | 340.158678 | 367.565103 | 317.786935 | -0.95670527 | 1.24E-07 | 3.74E-07 |
| gene-BPHYT_RS29470 | 65.4445881 | 61.8744435 | 57.952975 | 175.502158 | 130.828257 | 142.144014 | -1.27546568 | 1.25E-07 | 3.76E-07 |
| gene-BPHYT_RS03640 | 6310.61413 | 6516.01898 | 6855.73527 | 9942.49307 | 9312.47992 | 9025.69218 | -0.52299319 | 1.26E-07 | 3.80E-07 |
| gene-BPHYT_RS16285 | 9629.13361 | 10973.1125 | 10513.8897 | 14051.0183 | 16145.4529 | 16017.0048 | -0.57071843 | 1.29E-07 | 3.89E-07 |
| gene-BPHYT_RS38605 | 13.5677805 | 16.0020113 | 16.2675018 | 61.1299653 | 58.5612197 | 63.3763118 | -2.00605732 | 1.31E-07 | 3.95E-07 |
| gene-BPHYT_RS15325 | 1786.15839 | 1695.14639 | 1539.31235 | 2489.56714 | 2444.61943 | 2546.82236 | -0.57505161 | 1.32E-07 | 3.97E-07 |
| gene-BPHYT_RS14265 | 2075.07231 | 1952.24537 | 2090.37398 | 1440.49805 | 1379.30362 | 1306.4574 | 0.5685229 | 1.33E-07 | 3.99E-07 |
| gene-BPHYT_RS06220 | 146.053166 | 131.216492 | 120.989544 | 249.449697 | 259.164547 | 267.991261 | -0.96041735 | 1.33E-07 | 3.99E-07 |
| gene-BPHYT_RS02100 | 58.2616455 | 66.1416465 | 89.4712596 | 186.347797 | 149.518008 | 177.453673 | -1.27130369 | 1.33E-07 | 4.00E-07 |
| gene-BPHYT_RS07325 | 1092.60538 | 1435.91381 | 1882.96333 | 772.012304 | 745.098072 | 805.784536 | 0.92415017 | 1.34E-07 | 4.02E-07 |
| gene-BPHYT_RS09520 | 316.847579 | 244.297372 | 305.015658 | 548.197753 | 485.933525 | 503.388991 | -0.82590172 | 1.34E-07 | 4.02E-07 |
| gene-BPHYT_RS11200 | 199.526183 | 162.153714 | 181.992676 | 349.032383 | 368.811086 | 306.922424 | -0.90924344 | 1.35E-07 | 4.05E-07 |
| gene-BPHYT_RS13610 | 526.749124 | 593.141217 | 661.883977 | 925.823184 | 999.278686 | 964.225315 | -0.69860092 | 1.39E-07 | 4.15E-07 |
| gene-BPHYT_RS12745 | 51.0787029 | 64.008045 | 48.8025053 | 8.87370464 | 11.2138506 | 13.5806382 | 2.2765276 | 1.39E-07 | 4.16E-07 |
| gene-BPHYT_RS14890 | 3423.86931 | 4020.77203 | 4886.35084 | 2520.13212 | 2605.35129 | 2252.5752 | 0.7409847 | 1.45E-07 | 4.32E-07 |
| gene-BPHYT_RS02875 | 802.893362 | 719.023706 | 850.993685 | 1307.39248 | 1177.45431 | 1229.50045 | -0.64645902 | 1.45E-07 | 4.33E-07 |
| gene-BPHYT_RS29075 | 229.056058 | 208.026146 | 217.577836 | 345.088514 | 484.687542 | 406.513772 | -0.91330799 | 1.45E-07 | 4.33E-07 |
| gene-BPHYT_RS02395 | 1528.37056 | 1284.4281 | 1589.13158 | 875.538858 | 796.183392 | 996.818847 | 0.7210226 | 1.45E-07 | 4.34E-07 |
| gene-BPHYT_RS18670 | 1502.83121 | 1378.30657 | 1483.39282 | 2054.75561 | 2151.81333 | 2352.16654 | -0.58748534 | 1.46E-07 | 4.37E-07 |
| gene-BPHYT_RS19640 | 1037.53615 | 1042.26433 | 1177.36044 | 1570.64572 | 1669.61775 | 1713.87655 | -0.60538481 | 1.50E-07 | 4.46E-07 |
| gene-BPHYT_RS29315 | 51.0787029 | 61.8744435 | 85.4043842 | 163.670552 | 144.534074 | 188.318184 | -1.33372879 | 1.50E-07 | 4.48E-07 |
| gene-BPHYT_RS28835 | 176.381146 | 152.552507 | 114.889231 | 61.1299653 | 62.2991699 | 47.9849218 | 1.38101834 | 1.52E-07 | 4.53E-07 |
| gene-BPHYT_RS02810 | 1039.93047 | 1057.19954 | 1101.10653 | 1618.95811 | 1495.18008 | 1722.93031 | -0.59787902 | 1.56E-07 | 4.65E-07 |
| gene-BPHYT_RS07455 | 17.5583041 | 8.534406 | 12.2006263 | 47.3264248 | 72.2670371 | 61.56556 | -2.21445729 | 1.60E-07 | 4.76E-07 |
| gene-BPHYT_RS16420 | 728.669621 | 721.157307 | 841.843216 | 477.208116 | 464.751808 | 476.227714 | 0.69158048 | 1.61E-07 | 4.80E-07 |
| gene-BPHYT_RS06560 | 1492.45585 | 1642.87316 | 1628.78361 | 2334.77029 | 2293.85544 | 2335.86978 | -0.54847428 | 1.64E-07 | 4.87E-07 |
| gene-BPHYT_RS14655 | 2937.02542 | 2950.77088 | 2267.28306 | 4111.48315 | 4408.28926 | 4412.80205 | -0.66499844 | 1.68E-07 | 4.99E-07 |
| gene-BPHYT_RS02305 | 3209.17913 | 3771.14065 | 3809.64557 | 5299.57361 | 5522.19842 | 5242.12636 | -0.57442924 | 1.69E-07 | 5.03E-07 |
| gene-BPHYT_RS15120 | 94.1763585 | 125.882489 | 145.390797 | 249.449697 | 291.560115 | 239.019233 | -1.09770184 | 1.71E-07 | 5.07E-07 |
| gene-BPHYT_RS04720 | 420.601194 | 499.262751 | 598.847408 | 845.959842 | 934.487549 | 852.864082 | -0.79544809 | 1.71E-07 | 5.08E-07 |
| gene-BPHYT_RS09165 | 711.111317 | 821.436578 | 957.749165 | 526.506475 | 433.602223 | 469.890083 | 0.79792522 | 1.76E-07 | 5.22E-07 |
| gene-BPHYT_RS17075 | 475.670421 | 459.791123 | 537.844277 | 289.874352 | 299.036016 | 276.139644 | 0.76925357 | 1.81E-07 | 5.36E-07 |
| gene-BPHYT_RS22745 | 2087.04388 | 2181.60753 | 1990.73553 | 1482.89464 | 1234.76955 | 1383.41435 | 0.60905334 | 1.81E-07 | 5.36E-07 |
| gene-BPHYT_RS06945 | 223.469325 | 243.230571 | 263.330185 | 117.330095 | 114.630473 | 133.995631 | 0.99152569 | 1.84E-07 | 5.43E-07 |
| gene-BPHYT_RS01830 | 313.65516 | 343.509842 | 309.082533 | 539.324049 | 615.515799 | 498.862112 | -0.77391248 | 1.84E-07 | 5.43E-07 |
| gene-BPHYT_RS13485 | 20.7507231 | 41.6052293 | 28.4681281 | 105.498489 | 80.9889209 | 105.928978 | -1.70693895 | 1.87E-07 | 5.53E-07 |
| gene-BPHYT_RS13695 | 1775.78303 | 1302.56372 | 1225.14623 | 2471.81973 | 2402.25599 | 2421.88049 | -0.76039518 | 1.87E-07 | 5.53E-07 |
| gene-BPHYT_RS12625 | 2731.9125 | 2962.50568 | 2779.70936 | 3963.58807 | 4081.84161 | 4047.93557 | -0.51326232 | 1.91E-07 | 5.64E-07 |
| gene-BPHYT_RS02105 | 126.898653 | 116.281282 | 107.772199 | 210.996977 | 257.918564 | 238.113857 | -1.00586665 | 2.04E-07 | 6.01E-07 |
| gene-BPHYT_RS13905 | 320.039998 | 320.040225 | 331.450348 | 187.333765 | 184.405543 | 168.399914 | 0.84825492 | 2.04E-07 | 6.01E-07 |
| gene-BPHYT_RS09260 | 3263.45025 | 3044.64934 | 3198.59753 | 2120.81541 | 2196.66873 | 2283.35798 | 0.52636795 | 2.13E-07 | 6.29E-07 |
| gene-BPHYT_RS13155 | 108.542244 | 100.279271 | 66.0867259 | 28.5930483 | 31.149585 | 24.4451488 | 1.71729573 | 2.18E-07 | 6.43E-07 |
| gene-BPHYT_RS26535 | 134.8797 | 164.287316 | 136.240327 | 259.309369 | 271.624381 | 312.35468 | -0.9578045 | 2.18E-07 | 6.43E-07 |
| gene-BPHYT_RS06600 | 16928.5995 | 26081.1447 | 25942.5984 | 39289.8063 | 38873.436 | 41559.4692 | -0.79611182 | 2.19E-07 | 6.45E-07 |
| gene-BPHYT_RS19595 | 3732.73584 | 4179.72534 | 4291.57031 | 6199.76164 | 5882.28762 | 5727.40784 | -0.54556762 | 2.20E-07 | 6.46E-07 |
| gene-BPHYT_RS26050 | 59.857855 | 39.4716278 | 29.4848469 | 7.88773746 | 1.2459834 | 5.4322553 | 3.1002165 | 2.21E-07 | 6.50E-07 |
| gene-BPHYT_RS17045 | 43459.197 | 41070.7621 | 41852.2151 | 57899.9368 | 57188.146 | 60319.7628 | -0.47291613 | 2.27E-07 | 6.68E-07 |
| gene-BPHYT_RS24035 | 343.98314 | 293.370206 | 211.477523 | 135.077504 | 133.320224 | 128.563375 | 1.09949718 | 2.29E-07 | 6.73E-07 |
| gene-BPHYT_RS12300 | 268.16319 | 204.825744 | 73.2037579 | 3.94386873 | 17.4437676 | 14.4860141 | 3.93780451 | 2.31E-07 | 6.77E-07 |
| gene-BPHYT_RS01555 | 3565.13384 | 4127.4521 | 4487.79705 | 6299.34433 | 5950.81671 | 6157.46138 | -0.5961503 | 2.31E-07 | 6.78E-07 |
| gene-BPHYT_RS06420 | 2032.77276 | 1768.75564 | 1418.32281 | 971.177675 | 1046.62606 | 1085.54568 | 0.75081857 | 2.35E-07 | 6.88E-07 |
| gene-BPHYT_RS25785 | 105.349825 | 117.348083 | 120.989544 | 53.2422278 | 36.1335186 | 36.2150353 | 1.44680059 | 2.35E-07 | 6.90E-07 |
| gene-BPHYT_RS18185 | 4743.13643 | 5615.63915 | 6055.57753 | 8878.63448 | 8278.3137 | 8004.42818 | -0.61651182 | 2.36E-07 | 6.92E-07 |
| gene-BPHYT_RS16845 | 318.443789 | 326.44103 | 273.497373 | 185.36183 | 143.288091 | 143.04939 | 0.95993283 | 2.46E-07 | 7.19E-07 |
| gene-BPHYT_RS16375 | 2135.72827 | 2455.77533 | 1852.46176 | 3244.818 | 3437.6682 | 3396.06494 | -0.64536738 | 2.49E-07 | 7.29E-07 |
| gene-BPHYT_RS15640 | 1542.73645 | 1733.55122 | 1504.74391 | 2333.78432 | 2367.36846 | 2353.07192 | -0.56152231 | 2.60E-07 | 7.62E-07 |
| gene-BPHYT_RS08855 | 10080.0628 | 11561.9865 | 13053.6534 | 18804.3661 | 18140.2723 | 16544.8389 | -0.62458184 | 2.64E-07 | 7.72E-07 |
| gene-BPHYT_RS27105 | 814.066828 | 815.035773 | 769.656177 | 410.162348 | 543.248762 | 493.429856 | 0.73152439 | 2.67E-07 | 7.79E-07 |
| gene-BPHYT_RS26525 | 314.453265 | 284.8358 | 302.98222 | 536.366147 | 470.981725 | 500.672863 | -0.74075625 | 2.68E-07 | 7.82E-07 |
| gene-BPHYT_RS04785 | 485.247678 | 576.072405 | 476.841145 | 968.219773 | 822.349043 | 781.339387 | -0.74285078 | 2.73E-07 | 7.96E-07 |
| gene-BPHYT_RS07045 | 4.7886284 | 9.60120675 | 5.0835943 | 43.382556 | 29.9036016 | 56.1333047 | -2.76338418 | 2.74E-07 | 7.98E-07 |
| gene-BPHYT_RS07935 | 282.529076 | 294.437007 | 316.199565 | 162.684585 | 132.07424 | 172.926794 | 0.92678664 | 2.74E-07 | 7.99E-07 |
| gene-BPHYT_RS24280 | 634.493263 | 641.147251 | 570.37928 | 370.723661 | 338.907484 | 403.797644 | 0.72716095 | 2.75E-07 | 8.00E-07 |
| gene-BPHYT_RS22670 | 1175.60827 | 1238.55567 | 1197.69482 | 813.422925 | 829.824943 | 765.947997 | 0.58466917 | 2.80E-07 | 8.17E-07 |
| gene-BPHYT_RS24115 | 46.2900745 | 39.4716278 | 110.822356 | 9.85967182 | 13.7058174 | 10.8645106 | 2.52135801 | 2.87E-07 | 8.36E-07 |
| gene-BPHYT_RS26950 | 45.4919698 | 51.206436 | 70.1536013 | 5.91580309 | 12.459834 | 14.4860141 | 2.34237285 | 2.94E-07 | 8.54E-07 |
| gene-BPHYT_RS33535 | 14444.0995 | 15264.8519 | 11692.2669 | 21317.5965 | 21369.8613 | 20645.2863 | -0.61325095 | 2.95E-07 | 8.57E-07 |
| gene-BPHYT_RS06285 | 590.597503 | 556.869992 | 444.306142 | 300.719991 | 295.298065 | 312.35468 | 0.809717 | 2.97E-07 | 8.63E-07 |
| gene-BPHYT_RS06525 | 292.904437 | 350.977447 | 359.918476 | 183.389896 | 194.37341 | 159.346155 | 0.90359282 | 2.98E-07 | 8.64E-07 |
| gene-BPHYT_RS35215 | 106.946034 | 78.9432555 | 79.304071 | 202.123272 | 178.175626 | 197.371943 | -1.11720584 | 2.99E-07 | 8.66E-07 |
| gene-BPHYT_RS14845 | 1921.03809 | 1880.76972 | 1778.24129 | 3002.27007 | 2657.68259 | 2604.76642 | -0.56658774 | 3.00E-07 | 8.69E-07 |
| gene-BPHYT_RS15570 | 731.063936 | 972.922284 | 943.515101 | 526.506475 | 519.575077 | 531.455643 | 0.74514729 | 3.02E-07 | 8.75E-07 |
| gene-BPHYT_RS21385 | 47.0881793 | 44.8056315 | 43.718911 | 5.91580309 | 12.459834 | 5.4322553 | 2.55505241 | 3.04E-07 | 8.82E-07 |
| gene-BPHYT_RS07040 | 220.276906 | 219.760955 | 229.778462 | 377.625431 | 371.303053 | 399.270764 | -0.77869711 | 3.07E-07 | 8.90E-07 |
| gene-BPHYT_RS10655 | 79.8104733 | 55.473639 | 56.9362561 | 146.90911 | 189.389477 | 139.427886 | -1.29390144 | 3.10E-07 | 8.98E-07 |
| gene-BPHYT_RS12950 | 1205.13815 | 904.647036 | 913.013536 | 568.903064 | 502.13131 | 650.96526 | 0.81115494 | 3.17E-07 | 9.16E-07 |
| gene-BPHYT_RS21900 | 369.522492 | 379.781067 | 389.403323 | 223.81455 | 225.522995 | 214.574084 | 0.77875105 | 3.19E-07 | 9.23E-07 |
| gene-BPHYT_RS19985 | 21.5488278 | 23.4696165 | 17.2842206 | 66.0598012 | 77.2509707 | 69.713943 | -1.7707904 | 3.21E-07 | 9.27E-07 |
| gene-BPHYT_RS13245 | 5991.37223 | 6701.64231 | 7375.27861 | 10014.4687 | 9863.20458 | 9713.77785 | -0.56044564 | 3.23E-07 | 9.33E-07 |
| gene-BPHYT_RS34380 | 2526.00148 | 2554.9878 | 2284.56728 | 3495.25366 | 3542.3308 | 3553.60034 | -0.52388038 | 3.23E-07 | 9.34E-07 |
| Novel00757 | 173.986832 | 166.420917 | 202.327053 | 309.593695 | 322.7097 | 363.961105 | -0.8779271 | 3.30E-07 | 9.53E-07 |
| gene-BPHYT_RS20460 | 75.0218449 | 82.1436578 | 81.3375087 | 21.691278 | 22.4277012 | 29.8774041 | 1.67866465 | 3.31E-07 | 9.53E-07 |
| gene-BPHYT_RS21220 | 1281.7562 | 1136.1428 | 990.284169 | 1898.97279 | 1674.60169 | 1827.04853 | -0.66357915 | 3.31E-07 | 9.53E-07 |
| gene-BPHYT_RS12170 | 1776.58114 | 1695.14639 | 1944.98318 | 2624.64464 | 2555.51195 | 2880.00068 | -0.57377091 | 3.36E-07 | 9.68E-07 |
| gene-BPHYT_RS16715 | 3831.70082 | 4348.27986 | 4808.06349 | 6422.59023 | 6489.08154 | 6475.24832 | -0.57819619 | 3.37E-07 | 9.70E-07 |
| gene-BPHYT_RS18570 | 759.795706 | 775.564145 | 746.271643 | 1217.66947 | 1107.67924 | 1112.70696 | -0.5918761 | 3.38E-07 | 9.72E-07 |
| gene-BPHYT_RS22270 | 467.689374 | 501.396353 | 688.318668 | 978.079445 | 933.241565 | 981.427457 | -0.80518713 | 3.46E-07 | 9.94E-07 |
| gene-BPHYT_RS23670 | 179.573565 | 145.084902 | 135.223608 | 305.649827 | 289.068148 | 284.288027 | -0.92961774 | 3.47E-07 | 9.98E-07 |
| gene-BPHYT_RS21690 | 89.3877301 | 94.9452668 | 101.671886 | 32.536917 | 32.3955684 | 37.1204112 | 1.48147193 | 3.47E-07 | 9.98E-07 |
| gene-BPHYT_RS38660 | 87.7915207 | 162.153714 | 124.039701 | 37.4667529 | 46.1013857 | 45.2687942 | 1.53404816 | 3.49E-07 | 1.00E-06 |
| gene-BPHYT_RS15180 | 212.295859 | 244.297372 | 229.778462 | 420.02202 | 375.041003 | 391.122381 | -0.79240027 | 3.55E-07 | 1.02E-06 |
| gene-BPHYT_RS17740 | 351.964187 | 384.04827 | 392.45348 | 173.530224 | 219.293078 | 227.249347 | 0.8635912 | 3.65E-07 | 1.05E-06 |
| gene-BPHYT_RS02530 | 225.065535 | 256.03218 | 282.647843 | 90.7089808 | 152.009975 | 110.455858 | 1.11980694 | 3.74E-07 | 1.07E-06 |
| gene-BPHYT_RS24990 | 124.504338 | 105.613274 | 148.440954 | 32.536917 | 54.8232695 | 51.6064253 | 1.45176924 | 3.78E-07 | 1.08E-06 |
| gene-BPHYT_RS02535 | 308.068427 | 302.971413 | 331.450348 | 178.46006 | 160.731858 | 182.885928 | 0.8490117 | 3.78E-07 | 1.08E-06 |
| gene-BPHYT_RS16325 | 239.43142 | 252.831778 | 221.644711 | 501.857296 | 376.286986 | 409.229899 | -0.85236603 | 3.81E-07 | 1.09E-06 |
| gene-BPHYT_RS02635 | 2917.0728 | 3013.71212 | 3209.78144 | 2198.70682 | 2116.92579 | 1961.04416 | 0.5423369 | 3.95E-07 | 1.13E-06 |
| gene-BPHYT_RS19465 | 2743.88407 | 3106.52379 | 3253.50035 | 1999.54145 | 2033.44491 | 2120.39032 | 0.56458118 | 4.07E-07 | 1.16E-06 |
| gene-BPHYT_RS08000 | 245.018153 | 241.09697 | 171.825487 | 103.526554 | 98.4326885 | 106.834354 | 1.09101293 | 4.14E-07 | 1.18E-06 |
| gene-BPHYT_RS29995 | 39.9052367 | 33.0708233 | 43.718911 | 2.95790155 | 6.22991699 | 7.24300706 | 2.83044868 | 4.29E-07 | 1.22E-06 |
| gene-BPHYT_RS17130 | 3340.06831 | 4034.64044 | 3910.30073 | 2612.81303 | 2427.17566 | 2531.43097 | 0.57517872 | 4.50E-07 | 1.28E-06 |
| gene-BPHYT_RS17025 | 1047.11341 | 1090.27037 | 1066.53808 | 1690.93372 | 1437.86484 | 1754.61846 | -0.60930879 | 4.78E-07 | 1.36E-06 |
| gene-BPHYT_RS32655 | 435.765184 | 475.793135 | 355.851601 | 247.477763 | 203.095294 | 241.735361 | 0.86928829 | 4.83E-07 | 1.38E-06 |
| gene-BPHYT_RS33360 | 372.71491 | 294.437007 | 349.751288 | 159.726684 | 152.009975 | 210.952581 | 0.95684782 | 4.86E-07 | 1.39E-06 |
| gene-BPHYT_RS35695 | 62.2521692 | 105.613274 | 78.2873522 | 29.5790155 | 22.4277012 | 12.6752624 | 1.93181238 | 4.87E-07 | 1.39E-06 |
| gene-BPHYT_RS17270 | 181.967879 | 214.426951 | 172.842206 | 306.635794 | 413.666488 | 350.380467 | -0.91049558 | 4.97E-07 | 1.41E-06 |
| gene-BPHYT_RS03445 | 538.720695 | 577.139206 | 516.493181 | 309.593695 | 326.44765 | 356.718098 | 0.71616627 | 4.97E-07 | 1.42E-06 |
| gene-BPHYT_RS25980 | 39.1071319 | 32.0040225 | 25.4179715 | 0 | 1.2459834 | 0 | 6.44122605 | 5.13E-07 | 1.46E-06 |
| gene-BPHYT_RS16805 | 2040.7538 | 2157.07112 | 2431.99151 | 3575.117 | 3208.40725 | 3150.70807 | -0.58384834 | 5.15E-07 | 1.46E-06 |
| gene-BPHYT_RS16185 | 267.365086 | 330.708233 | 357.885039 | 170.572323 | 175.683659 | 162.062283 | 0.9101011 | 5.15E-07 | 1.46E-06 |
| gene-BPHYT_RS04700 | 399.850471 | 398.983481 | 391.436761 | 229.730354 | 254.180613 | 212.763333 | 0.77634289 | 5.27E-07 | 1.50E-06 |
| gene-BPHYT_RS09425 | 430.976556 | 327.50783 | 313.149409 | 635.948833 | 636.697517 | 592.115828 | -0.79513856 | 5.31E-07 | 1.51E-06 |
| Novel00550 | 1137.29925 | 1064.66715 | 1106.19012 | 1647.55116 | 1522.59171 | 1862.35819 | -0.60579872 | 5.32E-07 | 1.51E-06 |
| gene-BPHYT_RS04835 | 150.04369 | 113.08088 | 149.457672 | 260.295336 | 277.854298 | 258.937503 | -0.9453024 | 5.32E-07 | 1.51E-06 |
| gene-BPHYT_RS10690 | 23.1450373 | 35.2044248 | 19.3176583 | 63.1018997 | 102.170639 | 96.8752195 | -1.76009952 | 5.46E-07 | 1.55E-06 |
| gene-BPHYT_RS34235 | 24.7412467 | 19.2024135 | 29.4848469 | 84.7931777 | 74.7590039 | 73.3354465 | -1.66300815 | 5.46E-07 | 1.55E-06 |
| gene-BPHYT_RS02880 | 1122.13526 | 1058.26634 | 932.331194 | 563.973228 | 685.290869 | 681.74804 | 0.69003513 | 5.55E-07 | 1.57E-06 |
| gene-BPHYT_RS22345 | 75.8199497 | 77.8764548 | 99.6384482 | 32.536917 | 16.1977842 | 27.1612765 | 1.71969267 | 5.59E-07 | 1.58E-06 |
| gene-BPHYT_RS15095 | 889.088673 | 1014.52751 | 826.592433 | 607.355784 | 555.708596 | 554.09004 | 0.66823185 | 5.66E-07 | 1.60E-06 |
| gene-BPHYT_RS29630 | 336.800197 | 339.242639 | 357.885039 | 220.856649 | 180.667593 | 163.873035 | 0.87057061 | 5.69E-07 | 1.61E-06 |
| gene-BPHYT_RS15185 | 156.428528 | 137.617297 | 130.140014 | 263.253238 | 287.822165 | 248.978368 | -0.90997536 | 5.95E-07 | 1.68E-06 |
| gene-BPHYT_RS34120 | 1664.84647 | 1490.32065 | 1393.92156 | 877.510792 | 1037.90417 | 1014.02099 | 0.63610959 | 6.01E-07 | 1.70E-06 |
| gene-BPHYT_RS30895 | 137.274014 | 119.481684 | 93.5381351 | 28.5930483 | 57.3152363 | 34.4042836 | 1.56245954 | 6.22E-07 | 1.75E-06 |
| gene-BPHYT_RS24080 | 529.143438 | 423.519898 | 571.395999 | 276.070811 | 282.838231 | 310.543928 | 0.809637 | 6.32E-07 | 1.78E-06 |
| gene-BPHYT_RS29480 | 22.3469325 | 27.7368195 | 24.4012526 | 62.1159325 | 80.9889209 | 95.0644677 | -1.68373732 | 6.44E-07 | 1.81E-06 |
| gene-BPHYT_RS17285 | 2415.06492 | 2103.73108 | 2141.20992 | 3329.61117 | 3083.80891 | 3285.60908 | -0.54199751 | 6.46E-07 | 1.82E-06 |
| gene-BPHYT_RS23540 | 24.7412467 | 23.4696165 | 41.6854732 | 1.97193437 | 2.4919668 | 0.90537588 | 4.11315832 | 6.47E-07 | 1.82E-06 |
| gene-BPHYT_RS17365 | 2613.793 | 2629.66385 | 2965.76891 | 1871.36571 | 1834.08756 | 1944.7474 | 0.53849731 | 6.68E-07 | 1.88E-06 |
| gene-BPHYT_RS15820 | 315.25137 | 428.853902 | 423.971764 | 648.766406 | 746.344056 | 631.04699 | -0.79641238 | 6.75E-07 | 1.90E-06 |
| gene-BPHYT_RS03760 | 331.213464 | 326.44103 | 292.815032 | 479.180051 | 578.136297 | 526.928764 | -0.73482095 | 6.88E-07 | 1.94E-06 |
| gene-BPHYT_RS02665 | 4104.65264 | 3796.74387 | 4182.78139 | 5511.55655 | 5831.2023 | 5657.69389 | -0.49224399 | 6.91E-07 | 1.94E-06 |
| gene-BPHYT_RS23585 | 419.80309 | 377.647466 | 377.202697 | 143.951209 | 251.688647 | 198.277318 | 0.99026453 | 7.08E-07 | 1.99E-06 |
| gene-BPHYT_RS11685 | 422.197404 | 372.313462 | 229.778462 | 741.447321 | 680.306936 | 612.939473 | -0.98767411 | 7.21E-07 | 2.02E-06 |
| gene-BPHYT_RS22195 | 378.301644 | 338.175838 | 388.386604 | 594.538211 | 601.809981 | 571.292182 | -0.67651189 | 7.27E-07 | 2.04E-06 |
| gene-BPHYT_RS28015 | 459.708326 | 493.928747 | 553.095059 | 271.140975 | 296.544049 | 322.313814 | 0.75780462 | 7.30E-07 | 2.05E-06 |
| gene-BPHYT_RS03770 | 12802.398 | 12164.729 | 9661.87932 | 16770.3158 | 18151.4862 | 18152.7865 | -0.61595005 | 7.40E-07 | 2.07E-06 |
| gene-BPHYT_RS10970 | 225.065535 | 205.892545 | 36.6018789 | 4.92983591 | 0 | 4.52687942 | 5.58899645 | 7.45E-07 | 2.09E-06 |
| gene-BPHYT_RS05080 | 518.768077 | 483.26074 | 581.563188 | 868.637088 | 766.27979 | 882.741486 | -0.67003589 | 7.56E-07 | 2.12E-06 |
| gene-BPHYT_RS15405 | 1199.55141 | 1499.92186 | 1676.5694 | 2392.94235 | 2290.11749 | 2285.16873 | -0.67219676 | 7.57E-07 | 2.12E-06 |
| gene-BPHYT_RS10940 | 237.835211 | 282.702199 | 248.079402 | 106.484456 | 114.630473 | 156.630028 | 1.01850218 | 8.12E-07 | 2.27E-06 |
| gene-BPHYT_RS33350 | 82.2047875 | 107.746876 | 113.872512 | 30.5649827 | 43.6094189 | 35.3096594 | 1.47771865 | 8.12E-07 | 2.27E-06 |
| gene-BPHYT_RS04480 | 956.927575 | 1100.93837 | 943.515101 | 1492.75431 | 1451.57066 | 1539.139 | -0.58000213 | 8.20E-07 | 2.29E-06 |
| gene-BPHYT_RS33745 | 267.365086 | 225.094958 | 255.196434 | 153.81088 | 80.9889209 | 96.8752195 | 1.16797335 | 8.59E-07 | 2.40E-06 |
| gene-BPHYT_RS04250 | 306.472218 | 364.845857 | 442.272704 | 702.008634 | 635.451533 | 600.26421 | -0.80157942 | 8.89E-07 | 2.48E-06 |
| gene-BPHYT_RS33355 | 233.844687 | 222.961357 | 225.711587 | 125.217832 | 123.352356 | 114.077361 | 0.91441581 | 8.93E-07 | 2.49E-06 |
| gene-BPHYT_RS31130 | 106.946034 | 43.7388308 | 92.5214162 | 221.842616 | 189.389477 | 213.668708 | -1.35311456 | 8.95E-07 | 2.50E-06 |
| gene-BPHYT_RS02655 | 3557.1528 | 4071.97846 | 4284.45327 | 5700.86225 | 6003.14801 | 5627.81649 | -0.54103248 | 9.03E-07 | 2.52E-06 |
| gene-BPHYT_RS19905 | 106.14793 | 59.740842 | 52.8693807 | 12.8175734 | 23.6736846 | 18.1075177 | 2.02335429 | 9.12E-07 | 2.54E-06 |
| gene-BPHYT_RS37330 | 3265.04646 | 3048.91654 | 3163.01237 | 2262.79468 | 2202.89865 | 2290.60098 | 0.48818302 | 9.27E-07 | 2.58E-06 |
| gene-BPHYT_RS19560 | 12162.318 | 12384.4899 | 11145.2721 | 15716.3169 | 17219.4906 | 17572.4405 | -0.50089081 | 9.30E-07 | 2.59E-06 |
| gene-BPHYT_RS07670 | 648.859148 | 735.025717 | 566.312405 | 379.597365 | 337.661501 | 426.432041 | 0.76693932 | 9.33E-07 | 2.59E-06 |
| gene-BPHYT_RS22665 | 457.314012 | 510.997559 | 523.610213 | 290.860319 | 325.201667 | 290.625658 | 0.71943085 | 9.50E-07 | 2.64E-06 |
| gene-BPHYT_RS03250 | 501.209772 | 510.997559 | 546.994746 | 857.791449 | 1108.92523 | 736.070593 | -0.7918276 | 9.74E-07 | 2.71E-06 |
| gene-BPHYT_RS10025 | 35.914713 | 27.7368195 | 35.5851601 | 3.94386873 | 4.98393359 | 3.62150353 | 3.00327602 | 9.86E-07 | 2.74E-06 |
| gene-BPHYT_RS12350 | 1514.00468 | 1415.6446 | 1535.24548 | 1002.72862 | 1000.52467 | 720.679203 | 0.71453195 | 9.89E-07 | 2.74E-06 |
| gene-BPHYT_RS32685 | 348.771768 | 403.250684 | 337.550661 | 197.193437 | 230.506929 | 187.412808 | 0.828015 | 1.00E-06 | 2.78E-06 |
| gene-BPHYT_RS19455 | 223.469325 | 244.297372 | 252.146277 | 375.653496 | 398.714688 | 462.647076 | -0.78387447 | 1.01E-06 | 2.81E-06 |
| gene-BPHYT_RS16075 | 290.510123 | 268.833789 | 251.129558 | 139.021373 | 161.977842 | 141.238638 | 0.87902754 | 1.04E-06 | 2.88E-06 |
| gene-BPHYT_RS24305 | 1719.9157 | 1721.81641 | 1624.71674 | 1180.20272 | 1203.61996 | 1147.11124 | 0.52141921 | 1.04E-06 | 2.88E-06 |
| gene-BPHYT_RS18330 | 491.632516 | 589.940815 | 600.880846 | 991.882985 | 842.284777 | 861.012465 | -0.68209558 | 1.04E-06 | 2.89E-06 |
| gene-BPHYT_RS12610 | 4153.33703 | 4935.02027 | 5128.32993 | 7088.11807 | 6800.57739 | 6943.32765 | -0.5515788 | 1.05E-06 | 2.90E-06 |
| gene-BPHYT_RS19365 | 3113.40656 | 3382.82518 | 3396.85771 | 4765.17939 | 4785.82223 | 4417.32893 | -0.49777187 | 1.05E-06 | 2.90E-06 |
| gene-BPHYT_RS23930 | 371.916806 | 436.321507 | 436.172391 | 259.309369 | 230.506929 | 239.924609 | 0.76697986 | 1.06E-06 | 2.92E-06 |
| gene-BPHYT_RS30555 | 494.02683 | 428.853902 | 434.138953 | 175.502158 | 300.281999 | 231.776226 | 0.94560593 | 1.07E-06 | 2.95E-06 |
| gene-BPHYT_RS17885 | 4196.43469 | 4592.57723 | 3862.51495 | 6212.57922 | 5760.18125 | 6343.06344 | -0.53401439 | 1.09E-06 | 3.00E-06 |
| gene-BPHYT_RS33835 | 2197.98044 | 2816.35398 | 2470.62683 | 3966.54597 | 3694.34078 | 3616.07128 | -0.59191563 | 1.09E-06 | 3.00E-06 |
| gene-BPHYT_RS15295 | 63.0502739 | 45.8724323 | 71.1703202 | 14.7895077 | 19.9357344 | 10.8645106 | 2.00581479 | 1.09E-06 | 3.01E-06 |
| gene-BPHYT_RS26310 | 39.9052367 | 23.4696165 | 49.8192241 | 3.94386873 | 6.22991699 | 5.4322553 | 2.87300645 | 1.10E-06 | 3.03E-06 |
| gene-BPHYT_RS02770 | 51.8768077 | 38.404827 | 28.4681281 | 108.45639 | 140.796124 | 91.4429642 | -1.50390262 | 1.12E-06 | 3.09E-06 |
| gene-BPHYT_RS27110 | 2395.1123 | 2468.57694 | 2305.91837 | 1684.03195 | 1728.17897 | 1674.04001 | 0.49548427 | 1.17E-06 | 3.21E-06 |
| gene-BPHYT_RS22880 | 92.5801491 | 100.279271 | 106.75548 | 287.902417 | 153.255958 | 238.113857 | -1.18717578 | 1.17E-06 | 3.21E-06 |
| gene-BPHYT_RS15540 | 184.362193 | 221.894556 | 239.945651 | 387.485103 | 351.367318 | 392.933133 | -0.8135014 | 1.19E-06 | 3.29E-06 |
| gene-BPHYT_RS10580 | 1729.49296 | 1997.051 | 1999.886 | 2774.51165 | 2675.12636 | 2857.36629 | -0.53756635 | 1.22E-06 | 3.37E-06 |
| gene-BPHYT_RS28230 | 620.925482 | 507.797157 | 532.760682 | 304.663859 | 316.479783 | 368.487984 | 0.74750304 | 1.23E-06 | 3.38E-06 |
| gene-BPHYT_RS07440 | 181.169774 | 171.754921 | 248.079402 | 410.162348 | 338.907484 | 372.109488 | -0.90216787 | 1.23E-06 | 3.38E-06 |
| gene-BPHYT_RS03305 | 737.448774 | 813.968972 | 996.384482 | 1399.08743 | 1405.46927 | 1265.71548 | -0.67657171 | 1.23E-06 | 3.38E-06 |
| gene-BPHYT_RS02905 | 16941.3692 | 18307.3677 | 16610.136 | 12024.8558 | 12707.7847 | 12723.2473 | 0.46938561 | 1.24E-06 | 3.39E-06 |
| gene-BPHYT_RS14225 | 893.079196 | 1050.79874 | 947.581977 | 1575.57556 | 1376.81166 | 1414.19713 | -0.59599997 | 1.24E-06 | 3.39E-06 |
| gene-BPHYT_RS29505 | 2.3943142 | 12.801609 | 18.3009395 | 60.1439981 | 58.5612197 | 56.1333047 | -2.42248154 | 1.26E-06 | 3.46E-06 |
| gene-BPHYT_RS02995 | 33.5203988 | 28.8036203 | 16.2675018 | 70.9896371 | 99.6786719 | 83.2945812 | -1.67776461 | 1.28E-06 | 3.51E-06 |
| gene-BPHYT_RS09160 | 14455.2729 | 16469.27 | 15262.9835 | 10440.4065 | 11630.009 | 10399.1474 | 0.50847447 | 1.29E-06 | 3.52E-06 |
| gene-BPHYT_RS30810 | 312.058951 | 248.564575 | 464.640519 | 169.586355 | 164.469809 | 143.954765 | 1.10190389 | 1.29E-06 | 3.52E-06 |
| gene-BPHYT_RS06700 | 1282.55431 | 1402.84299 | 1111.27371 | 1848.68847 | 1935.01222 | 1928.45063 | -0.58930877 | 1.36E-06 | 3.72E-06 |
| gene-BPHYT_RS23310 | 94.1763585 | 97.0788683 | 92.5214162 | 43.382556 | 31.149585 | 30.78278 | 1.42664533 | 1.41E-06 | 3.87E-06 |
| gene-BPHYT_RS22860 | 114.927082 | 142.951301 | 88.4545408 | 226.772452 | 260.41053 | 222.722467 | -1.03495966 | 1.42E-06 | 3.88E-06 |
| gene-BPHYT_RS09655 | 546.701742 | 577.139206 | 510.392867 | 328.327072 | 362.581169 | 332.272949 | 0.67713533 | 1.43E-06 | 3.89E-06 |
| gene-BPHYT_RS19620 | 431.774661 | 470.459131 | 565.295686 | 723.699912 | 884.648213 | 812.122167 | -0.72242184 | 1.43E-06 | 3.89E-06 |
| gene-BPHYT_RS34725 | 390.273215 | 358.445052 | 302.98222 | 178.46006 | 213.063161 | 197.371943 | 0.8404637 | 1.44E-06 | 3.92E-06 |
| gene-BPHYT_RS17675 | 550.692266 | 504.596755 | 624.26538 | 856.805481 | 903.337964 | 849.242578 | -0.6350199 | 1.52E-06 | 4.16E-06 |
| gene-BPHYT_RS24700 | 273.749924 | 231.495763 | 222.66143 | 148.881045 | 109.646539 | 103.212851 | 1.00962391 | 1.53E-06 | 4.17E-06 |
| gene-BPHYT_RS12490 | 2332.06203 | 2498.44736 | 2417.75745 | 3287.21459 | 3369.13911 | 3415.98321 | -0.47496348 | 1.56E-06 | 4.24E-06 |
| gene-BPHYT_RS13895 | 1876.34423 | 1503.12226 | 1449.84109 | 945.542528 | 1050.36401 | 1078.30268 | 0.65257854 | 1.60E-06 | 4.36E-06 |
| gene-BPHYT_RS07570 | 174.784937 | 149.352105 | 170.808768 | 78.8773746 | 88.4648213 | 69.713943 | 1.06980669 | 1.63E-06 | 4.42E-06 |
| gene-BPHYT_RS04475 | 415.812566 | 470.459131 | 452.439892 | 746.377157 | 753.819956 | 629.236239 | -0.66971139 | 1.63E-06 | 4.42E-06 |
| gene-BPHYT_RS08295 | 6475.82181 | 3719.93422 | 2803.0939 | 1239.36075 | 1300.80667 | 1292.87676 | 1.76196647 | 1.65E-06 | 4.48E-06 |
| gene-BPHYT_RS16740 | 166.803889 | 164.287316 | 188.092989 | 365.793825 | 292.806099 | 285.193403 | -0.86354507 | 1.65E-06 | 4.48E-06 |
| gene-BPHYT_RS34925 | 232.248477 | 173.888522 | 151.49111 | 89.7230136 | 80.9889209 | 86.0107089 | 1.12147873 | 1.66E-06 | 4.51E-06 |
| gene-BPHYT_RS29195 | 293.702542 | 311.505819 | 413.804576 | 565.945163 | 569.414413 | 608.412593 | -0.77733681 | 1.67E-06 | 4.54E-06 |
| gene-BPHYT_RS04210 | 381.494063 | 333.908635 | 301.965501 | 589.608375 | 512.099177 | 578.535189 | -0.7227622 | 1.68E-06 | 4.55E-06 |
| gene-BPHYT_RS06745 | 565.058151 | 548.335586 | 551.061622 | 284.944516 | 378.778953 | 345.853587 | 0.72401831 | 1.69E-06 | 4.59E-06 |
| gene-BPHYT_RS14195 | 2048.73485 | 2515.51617 | 2700.40529 | 3832.45444 | 3412.74853 | 3927.52058 | -0.62187325 | 1.70E-06 | 4.61E-06 |
| gene-BPHYT_RS13140 | 201.920498 | 262.432985 | 311.115971 | 148.881045 | 94.6947383 | 115.888113 | 1.10175576 | 1.78E-06 | 4.82E-06 |
| gene-BPHYT_RS02815 | 1338.42164 | 1388.97458 | 1438.65719 | 784.829877 | 1017.96844 | 910.808138 | 0.61959937 | 1.79E-06 | 4.86E-06 |
| gene-BPHYT_RS23660 | 457.314012 | 296.570609 | 370.085665 | 185.36183 | 174.437676 | 223.627843 | 0.9455797 | 1.81E-06 | 4.90E-06 |
| gene-BPHYT_RS13730 | 1673.62563 | 1586.33272 | 1425.43984 | 1075.6902 | 971.867051 | 1095.50482 | 0.5754893 | 1.81E-06 | 4.90E-06 |
| gene-BPHYT_RS29655 | 264.970771 | 296.570609 | 300.948782 | 145.923143 | 184.405543 | 139.427886 | 0.8815504 | 1.81E-06 | 4.90E-06 |
| gene-BPHYT_RS20085 | 25.5393515 | 19.2024135 | 36.6018789 | 89.7230136 | 82.2349043 | 74.2408224 | -1.59692472 | 1.82E-06 | 4.92E-06 |
| gene-BPHYT_RS18780 | 1296.92019 | 1081.73596 | 1089.92262 | 729.615715 | 817.365109 | 649.154508 | 0.66210972 | 1.87E-06 | 5.05E-06 |
| gene-BPHYT_RS35400 | 452.525384 | 476.859935 | 531.743963 | 940.612692 | 671.585052 | 822.081302 | -0.73881426 | 1.88E-06 | 5.07E-06 |
| gene-BPHYT_RS04985 | 169.198203 | 172.821722 | 199.276896 | 310.579662 | 320.217733 | 307.8278 | -0.79505803 | 1.88E-06 | 5.09E-06 |
| gene-BPHYT_RS30250 | 837.211865 | 700.888093 | 659.85054 | 429.881692 | 406.190588 | 498.862112 | 0.71872133 | 1.90E-06 | 5.13E-06 |
| gene-BPHYT_RS17690 | 1153.26134 | 1272.6933 | 1327.83483 | 1808.26381 | 1756.83659 | 1868.69582 | -0.53467519 | 1.96E-06 | 5.29E-06 |
| gene-BPHYT_RS28875 | 112.532767 | 99.2124698 | 78.2873522 | 41.4106217 | 26.1656514 | 36.2150353 | 1.47436276 | 2.02E-06 | 5.43E-06 |
| gene-BPHYT_RS31170 | 97.3687775 | 98.145669 | 103.705324 | 48.3123919 | 21.1817178 | 34.4042836 | 1.50996357 | 2.06E-06 | 5.54E-06 |
| gene-BPHYT_RS34715 | 74.2237402 | 93.878466 | 77.2706333 | 22.6772452 | 37.379502 | 13.5806382 | 1.76384609 | 2.09E-06 | 5.62E-06 |
| gene-BPHYT_RS14070 | 36.7128177 | 56.5404398 | 42.7021921 | 101.55462 | 105.908589 | 134.901007 | -1.34426869 | 2.11E-06 | 5.68E-06 |
| gene-BPHYT_RS12550 | 6038.46041 | 6970.4761 | 7333.59313 | 4921.94817 | 4674.92971 | 4424.57194 | 0.53671652 | 2.16E-06 | 5.81E-06 |
| gene-BPHYT_RS16640 | 3855.64397 | 3853.28431 | 4184.81483 | 5512.54252 | 5438.71753 | 5420.48541 | -0.46109172 | 2.16E-06 | 5.82E-06 |
| gene-BPHYT_RS12295 | 204.314812 | 229.362161 | 275.530811 | 127.189767 | 128.33629 | 110.455858 | 0.95490803 | 2.19E-06 | 5.87E-06 |
| gene-BPHYT_RS08015 | 86.1953112 | 66.1416465 | 41.6854732 | 13.8035406 | 17.4437676 | 19.0128935 | 1.95384235 | 2.19E-06 | 5.89E-06 |
| gene-BPHYT_RS11000 | 142.860747 | 158.953312 | 101.671886 | 234.660189 | 321.463717 | 262.559006 | -1.0175552 | 2.25E-06 | 6.05E-06 |
| gene-BPHYT_RS25955 | 322.434312 | 226.161759 | 275.530811 | 443.685232 | 571.90638 | 463.552452 | -0.83824879 | 2.26E-06 | 6.07E-06 |
| gene-BPHYT_RS11280 | 287.317704 | 281.635398 | 316.199565 | 159.726684 | 189.389477 | 137.617134 | 0.86883654 | 2.31E-06 | 6.19E-06 |
| gene-BPHYT_RS06615 | 806.883885 | 1059.33315 | 1152.95919 | 1669.24244 | 1725.68701 | 1496.58634 | -0.69710913 | 2.31E-06 | 6.20E-06 |
| gene-BPHYT_RS04380 | 229.854163 | 200.558541 | 198.260178 | 362.835923 | 342.645435 | 356.718098 | -0.75451941 | 2.32E-06 | 6.22E-06 |
| gene-BPHYT_RS12035 | 1332.0368 | 1463.65063 | 1227.17966 | 974.135576 | 846.022728 | 787.677018 | 0.62523323 | 2.33E-06 | 6.24E-06 |
| gene-BPHYT_RS15400 | 1520.38952 | 1953.31217 | 1895.16395 | 2806.0626 | 2697.55406 | 2589.37503 | -0.59288803 | 2.37E-06 | 6.33E-06 |
| gene-BPHYT_RS20845 | 727.871517 | 687.019683 | 433.122234 | 327.341105 | 336.415518 | 329.556821 | 0.89695365 | 2.41E-06 | 6.45E-06 |
| gene-BPHYT_RS29895 | 57.4635408 | 85.34406 | 76.2539145 | 22.6772452 | 19.9357344 | 26.2559006 | 1.65552991 | 2.48E-06 | 6.63E-06 |
| gene-BPHYT_RS33680 | 113.330872 | 110.947278 | 146.407516 | 52.2562607 | 53.5772861 | 53.4171771 | 1.21794584 | 2.50E-06 | 6.67E-06 |
| gene-BPHYT_RS37340 | 800.499047 | 872.643014 | 765.589301 | 1261.05203 | 1136.33686 | 1204.14992 | -0.56342763 | 2.52E-06 | 6.73E-06 |
| gene-BPHYT_RS08735 | 191.545136 | 154.686109 | 184.026114 | 328.327072 | 348.875352 | 277.950396 | -0.84387568 | 2.52E-06 | 6.73E-06 |
| gene-BPHYT_RS19670 | 860.356902 | 908.914239 | 940.464945 | 640.878669 | 588.104164 | 586.683572 | 0.57677569 | 2.62E-06 | 6.98E-06 |
| gene-BPHYT_RS28220 | 33.5203988 | 34.137624 | 25.4179715 | 4.92983591 | 4.98393359 | 0.90537588 | 3.15421189 | 2.67E-06 | 7.12E-06 |
| gene-BPHYT_RS26250 | 23.1450373 | 37.3380263 | 25.4179715 | 0 | 1.2459834 | 4.52687942 | 3.82438051 | 2.67E-06 | 7.12E-06 |
| gene-BPHYT_RS28840 | 126.898653 | 187.756932 | 145.390797 | 67.0457684 | 32.3955684 | 73.3354465 | 1.39450902 | 2.75E-06 | 7.33E-06 |
| gene-BPHYT_RS22630 | 118.917605 | 155.75291 | 154.541267 | 71.9756043 | 48.5933525 | 65.1870636 | 1.19631927 | 2.77E-06 | 7.38E-06 |
| gene-BPHYT_RS06715 | 1852.40109 | 1253.49088 | 930.297756 | 723.699912 | 713.948487 | 671.788905 | 0.93739002 | 2.86E-06 | 7.60E-06 |
| gene-BPHYT_RS36675 | 629.704635 | 745.693724 | 601.897565 | 321.425301 | 469.735741 | 343.13746 | 0.80519209 | 2.88E-06 | 7.66E-06 |
| gene-BPHYT_RS30575 | 295.298751 | 508.863958 | 861.160874 | 2227.29987 | 2158.04325 | 2271.58809 | -1.99971148 | 3.04E-06 | 8.07E-06 |
| gene-BPHYT_RS04520 | 186.756508 | 157.886511 | 65.070007 | 45.3544904 | 48.5933525 | 36.2150353 | 1.66231489 | 3.06E-06 | 8.14E-06 |
| gene-BPHYT_RS32830 | 279.336657 | 283.769 | 311.115971 | 184.375863 | 115.876456 | 157.535404 | 0.92625502 | 3.08E-06 | 8.18E-06 |
| gene-BPHYT_RS07855 | 87.7915207 | 57.6072405 | 67.1034447 | 162.684585 | 133.320224 | 179.264425 | -1.15620473 | 3.14E-06 | 8.34E-06 |
| gene-BPHYT_RS05570 | 41.5014461 | 30.9372218 | 42.7021921 | 105.498489 | 90.9567881 | 95.0644677 | -1.33637369 | 3.22E-06 | 8.55E-06 |
| gene-BPHYT_RS22710 | 252.201096 | 256.03218 | 318.233003 | 501.857296 | 422.388372 | 462.647076 | -0.74949107 | 3.33E-06 | 8.83E-06 |
| gene-BPHYT_RS17670 | 1215.51351 | 1275.8937 | 1293.26639 | 1736.28821 | 2001.04934 | 1740.13245 | -0.53288095 | 3.36E-06 | 8.90E-06 |
| gene-BPHYT_RS17090 | 784.536953 | 739.29292 | 992.317607 | 1308.37845 | 1360.61387 | 1261.18861 | -0.64337374 | 3.36E-06 | 8.91E-06 |
| gene-BPHYT_RS15170 | 316.847579 | 307.238616 | 316.199565 | 476.222149 | 473.473691 | 539.604026 | -0.66440867 | 3.41E-06 | 9.04E-06 |
| gene-BPHYT_RS07970 | 937.773062 | 848.106596 | 908.94666 | 1205.83786 | 1399.23936 | 1369.83371 | -0.55969393 | 3.50E-06 | 9.27E-06 |
| gene-BPHYT_RS06200 | 240.229525 | 296.570609 | 246.045964 | 381.5693 | 478.457625 | 479.849218 | -0.77678287 | 3.53E-06 | 9.33E-06 |
| gene-BPHYT_RS11350 | 126.100548 | 92.8116653 | 94.5548539 | 34.5088514 | 38.6254854 | 48.8902977 | 1.36051915 | 3.55E-06 | 9.38E-06 |
| gene-BPHYT_RS09150 | 34935.4385 | 36031.1953 | 34646.7286 | 24230.1435 | 26561.8741 | 26854.3541 | 0.44380665 | 3.60E-06 | 9.51E-06 |
| gene-BPHYT_RS23265 | 1407.05865 | 1423.1122 | 1463.05844 | 1971.93437 | 2323.75904 | 1949.27428 | -0.53990891 | 3.63E-06 | 9.58E-06 |
| gene-BPHYT_RS34100 | 223.469325 | 213.36015 | 198.260178 | 327.341105 | 353.859285 | 390.217006 | -0.75384855 | 3.63E-06 | 9.60E-06 |
| gene-BPHYT_RS28090 | 58.2616455 | 54.4068383 | 62.0198504 | 15.7754749 | 22.4277012 | 11.7698865 | 1.82977956 | 3.70E-06 | 9.77E-06 |
| gene-BPHYT_RS25965 | 34.3185035 | 56.5404398 | 34.5684412 | 5.91580309 | 12.459834 | 4.52687942 | 2.49535873 | 3.71E-06 | 9.79E-06 |
| gene-BPHYT_RS19935 | 91.7820443 | 74.6760525 | 103.705324 | 39.4386873 | 19.9357344 | 33.4989077 | 1.52531404 | 3.71E-06 | 9.79E-06 |
| gene-BPHYT_RS30125 | 383.090272 | 468.325529 | 356.86832 | 197.193437 | 269.132414 | 207.331077 | 0.84672423 | 3.75E-06 | 9.87E-06 |
| gene-BPHYT_RS21275 | 185.958403 | 140.817699 | 140.307203 | 56.2001294 | 49.8393359 | 88.7268365 | 1.25370844 | 3.79E-06 | 9.99E-06 |
| gene-BPHYT_RS33645 | 1304.10313 | 1388.97458 | 1183.46075 | 1773.75496 | 1896.38673 | 1935.69364 | -0.53220011 | 3.79E-06 | 9.99E-06 |
| gene-BPHYT_RS20735 | 90.9839396 | 99.2124698 | 96.5882916 | 179.446027 | 184.405543 | 182.885928 | -0.93265116 | 3.81E-06 | 1.00E-05 |
| gene-BPHYT_RS29755 | 58.2616455 | 56.5404398 | 41.6854732 | 131.133635 | 102.170639 | 133.995631 | -1.23143659 | 3.82E-06 | 1.00E-05 |
| gene-BPHYT_RS02785 | 809.2782 | 684.886082 | 581.563188 | 381.5693 | 465.997791 | 391.122381 | 0.74846565 | 3.83E-06 | 1.01E-05 |
| gene-BPHYT_RS15370 | 193.93945 | 201.625342 | 185.042832 | 93.6668823 | 108.400556 | 104.118227 | 0.92506233 | 3.84E-06 | 1.01E-05 |
| gene-BPHYT_RS08800 | 261.778353 | 272.034191 | 614.098191 | 1317.25216 | 1505.14795 | 1435.92615 | -1.89180016 | 3.88E-06 | 1.02E-05 |
| gene-BPHYT_RS04925 | 2033.57086 | 2391.76728 | 2534.68012 | 3560.3275 | 3602.13801 | 3141.65431 | -0.56634928 | 3.97E-06 | 1.04E-05 |
| gene-BPHYT_RS28815 | 413.418252 | 379.781067 | 323.316597 | 553.127589 | 594.334081 | 688.991047 | -0.71703545 | 4.09E-06 | 1.07E-05 |
| gene-BPHYT_RS22520 | 33.5203988 | 49.0728345 | 35.5851601 | 112.400259 | 92.2027715 | 93.253716 | -1.34300464 | 4.09E-06 | 1.07E-05 |
| gene-BPHYT_RS02390 | 604.165283 | 498.19595 | 644.599757 | 306.635794 | 388.74682 | 355.812722 | 0.73544985 | 4.17E-06 | 1.09E-05 |
| gene-BPHYT_RS19315 | 340.790721 | 478.993537 | 391.436761 | 217.898747 | 255.426597 | 210.047205 | 0.82660287 | 4.23E-06 | 1.11E-05 |
| gene-BPHYT_RS08750 | 114.927082 | 109.880477 | 148.440954 | 232.688255 | 230.506929 | 232.681602 | -0.90037035 | 4.37E-06 | 1.14E-05 |
| gene-BPHYT_RS07790 | 188.352717 | 258.165782 | 279.597686 | 444.671199 | 446.062057 | 392.027757 | -0.82445701 | 4.42E-06 | 1.16E-05 |
| gene-BPHYT_RS33970 | 2193.98991 | 2509.11537 | 2359.80447 | 3238.90219 | 3531.11695 | 3205.936 | -0.49830452 | 4.42E-06 | 1.16E-05 |
| gene-BPHYT_RS28885 | 416.610671 | 314.706221 | 366.018789 | 182.403929 | 224.277012 | 219.100964 | 0.81399987 | 4.44E-06 | 1.16E-05 |
| gene-BPHYT_RS26390 | 359.945235 | 375.513864 | 429.055359 | 255.3655 | 193.127427 | 227.249347 | 0.78070812 | 4.45E-06 | 1.16E-05 |
| gene-BPHYT_RS03875 | 83.0028923 | 76.809654 | 77.2706333 | 29.5790155 | 28.6576182 | 29.8774041 | 1.42777033 | 4.45E-06 | 1.16E-05 |
| gene-BPHYT_RS17410 | 137.274014 | 112.014079 | 92.5214162 | 234.660189 | 203.095294 | 229.060098 | -0.96000687 | 4.64E-06 | 1.21E-05 |
| gene-BPHYT_RS15620 | 3373.58871 | 3583.38372 | 3616.46898 | 2620.70077 | 2612.82719 | 2364.84181 | 0.47682927 | 4.73E-06 | 1.24E-05 |
| gene-BPHYT_RS26235 | 87.7915207 | 115.214481 | 79.304071 | 33.5228842 | 27.4116348 | 42.5526665 | 1.43358384 | 4.86E-06 | 1.27E-05 |
| gene-BPHYT_RS25035 | 1225.09077 | 600.608822 | 672.051166 | 417.064118 | 428.618289 | 387.500878 | 1.02066015 | 4.90E-06 | 1.28E-05 |
| gene-BPHYT_RS26995 | 54.2711219 | 58.6740413 | 53.8860995 | 140.00734 | 110.892523 | 117.698865 | -1.14706403 | 5.00E-06 | 1.30E-05 |
| gene-BPHYT_RS16250 | 1624.14313 | 1472.18504 | 1639.96752 | 2184.90328 | 2275.16569 | 2181.0505 | -0.48708957 | 5.15E-06 | 1.34E-05 |
| gene-BPHYT_RS02945 | 316.049474 | 416.052293 | 369.068946 | 604.397883 | 588.104164 | 567.670679 | -0.67949853 | 5.16E-06 | 1.34E-05 |
| gene-BPHYT_RS15440 | 829.230818 | 879.043818 | 853.027123 | 1170.34305 | 1206.11193 | 1389.75198 | -0.55731722 | 5.16E-06 | 1.34E-05 |
| gene-BPHYT_RS16350 | 5656.16824 | 6216.24797 | 6476.49913 | 4711.93716 | 3952.25934 | 4149.33767 | 0.51767519 | 5.37E-06 | 1.40E-05 |
| gene-BPHYT_RS26990 | 472.478002 | 418.185894 | 432.105515 | 685.247192 | 684.044886 | 638.289998 | -0.59977515 | 5.40E-06 | 1.40E-05 |
| gene-BPHYT_RS28315 | 158.822842 | 154.686109 | 78.2873522 | 55.2141622 | 52.3313027 | 34.4042836 | 1.47291968 | 5.50E-06 | 1.43E-05 |
| gene-BPHYT_RS30180 | 131.687281 | 110.947278 | 101.671886 | 43.382556 | 49.8393359 | 55.2279289 | 1.21552322 | 5.52E-06 | 1.43E-05 |
| gene-BPHYT_RS02095 | 502.007877 | 341.37624 | 429.055359 | 259.309369 | 232.998896 | 233.586978 | 0.81180688 | 5.53E-06 | 1.44E-05 |
| gene-BPHYT_RS16620 | 2872.37894 | 3501.24006 | 3285.01863 | 4862.79014 | 4519.18179 | 4440.86871 | -0.51763131 | 5.53E-06 | 1.44E-05 |
| gene-BPHYT_RS08460 | 1135.70304 | 1037.99713 | 1063.48793 | 1545.99654 | 1596.10473 | 1463.08743 | -0.507363 | 5.55E-06 | 1.44E-05 |
| gene-BPHYT_RS21740 | 149.245585 | 168.554519 | 159.624861 | 71.9756043 | 77.2509707 | 87.8214607 | 1.00615102 | 5.63E-06 | 1.46E-05 |
| gene-BPHYT_RS04920 | 960.918099 | 1174.54763 | 1089.92262 | 1640.64939 | 1579.90695 | 1505.64009 | -0.55224328 | 5.69E-06 | 1.47E-05 |
| gene-BPHYT_RS14175 | 1636.1147 | 1944.77777 | 2125.95914 | 1324.15393 | 1292.08478 | 1128.09835 | 0.60785212 | 5.74E-06 | 1.49E-05 |
| gene-BPHYT_RS29910 | 756.603287 | 722.224108 | 831.676027 | 520.590672 | 530.788928 | 416.472906 | 0.65661527 | 5.78E-06 | 1.50E-05 |
| gene-BPHYT_RS30085 | 197.131869 | 188.823733 | 247.062683 | 100.568653 | 97.1867051 | 124.941872 | 0.96658893 | 5.88E-06 | 1.52E-05 |
| gene-BPHYT_RS03830 | 254.59541 | 241.09697 | 211.477523 | 376.639464 | 367.565103 | 430.95892 | -0.73260433 | 5.88E-06 | 1.52E-05 |
| gene-BPHYT_RS02015 | 572.241094 | 812.902172 | 645.616476 | 448.615068 | 387.500837 | 372.109488 | 0.74728308 | 5.90E-06 | 1.52E-05 |
| gene-BPHYT_RS16400 | 182.765984 | 106.680075 | 230.795181 | 70.0036699 | 76.0049873 | 68.8085671 | 1.2797165 | 5.91E-06 | 1.53E-05 |
| gene-BPHYT_RS26820 | 118.119501 | 101.346071 | 114.889231 | 191.277633 | 211.817178 | 216.384836 | -0.88685046 | 6.10E-06 | 1.58E-05 |
| gene-BPHYT_RS20395 | 81.4066828 | 68.275248 | 52.8693807 | 131.133635 | 145.780058 | 167.494538 | -1.12806363 | 6.12E-06 | 1.58E-05 |
| gene-BPHYT_RS13760 | 205.911021 | 171.754921 | 179.959238 | 115.35816 | 57.3152363 | 75.1461983 | 1.16384059 | 6.16E-06 | 1.59E-05 |
| gene-BPHYT_RS02850 | 632.098949 | 806.501367 | 870.311344 | 442.699265 | 512.099177 | 299.679417 | 0.88247934 | 6.18E-06 | 1.59E-05 |
| gene-BPHYT_RS34625 | 440.553813 | 390.449075 | 75.2371956 | 0 | 0 | 5.4322553 | 7.33348747 | 6.21E-06 | 1.60E-05 |
| gene-BPHYT_RS06895 | 10457.5663 | 10194.348 | 9018.29628 | 6762.7489 | 7514.52588 | 6946.94915 | 0.48351268 | 6.29E-06 | 1.62E-05 |
| gene-BPHYT_RS19055 | 399.850471 | 491.795146 | 357.885039 | 682.28929 | 682.798902 | 641.006125 | -0.68382323 | 6.29E-06 | 1.62E-05 |
| gene-BPHYT_RS28150 | 42.2995509 | 49.0728345 | 44.7356298 | 11.8316062 | 12.459834 | 9.95913471 | 1.99668256 | 6.34E-06 | 1.63E-05 |
| gene-BPHYT_RS02855 | 5089.51388 | 5670.04599 | 5975.25674 | 4007.9566 | 4068.1358 | 3236.71878 | 0.56510033 | 6.35E-06 | 1.63E-05 |
| gene-BPHYT_RS17825 | 141.264538 | 114.14768 | 139.290484 | 235.646157 | 218.047095 | 270.707389 | -0.8757913 | 6.45E-06 | 1.66E-05 |
| gene-BPHYT_RS02060 | 63.8483787 | 32.0040225 | 55.9195373 | 148.881045 | 142.042107 | 99.5913471 | -1.35054307 | 6.60E-06 | 1.70E-05 |
| gene-BPHYT_RS16565 | 7.1829426 | 3.20040225 | 2.03343772 | 39.4386873 | 29.9036016 | 24.4451488 | -2.86495905 | 6.68E-06 | 1.72E-05 |
| gene-BPHYT_RS17555 | 119.71571 | 132.283293 | 127.089857 | 64.0878669 | 39.8714688 | 61.56556 | 1.18201761 | 6.73E-06 | 1.73E-05 |
| gene-BPHYT_RS24370 | 8552.49032 | 8537.6064 | 7289.87422 | 5639.73228 | 5993.18015 | 5804.36479 | 0.48366615 | 6.88E-06 | 1.77E-05 |
| gene-BPHYT_RS24685 | 430.178451 | 543.001582 | 557.161935 | 293.81822 | 302.773966 | 333.178325 | 0.71588136 | 6.91E-06 | 1.77E-05 |
| gene-BPHYT_RS21410 | 272.951819 | 278.434996 | 271.463935 | 404.246545 | 446.062057 | 447.255686 | -0.65691911 | 6.94E-06 | 1.78E-05 |
| gene-BPHYT_RS12040 | 855.568274 | 936.651059 | 917.080411 | 1486.83851 | 1181.19226 | 1365.30683 | -0.57564886 | 6.99E-06 | 1.79E-05 |
| gene-BPHYT_RS12345 | 3602.64477 | 3569.51531 | 3402.95802 | 2547.7392 | 2511.90253 | 1840.62917 | 0.61660444 | 6.99E-06 | 1.79E-05 |
| gene-BPHYT_RS14500 | 2431.82512 | 2130.4011 | 2027.33741 | 3151.15111 | 3065.11916 | 3115.39841 | -0.50141285 | 7.02E-06 | 1.80E-05 |
| gene-BPHYT_RS03955 | 75.0218449 | 52.2732368 | 79.304071 | 29.5790155 | 13.7058174 | 18.1075177 | 1.74009768 | 7.12E-06 | 1.82E-05 |
| gene-BPHYT_RS17560 | 926.599595 | 841.705792 | 782.873522 | 1172.31498 | 1297.06872 | 1266.62086 | -0.54895 | 7.49E-06 | 1.92E-05 |
| gene-BPHYT_RS26030 | 30.3279799 | 23.4696165 | 28.4681281 | 1.97193437 | 0 | 5.4322553 | 3.3733347 | 7.54E-06 | 1.93E-05 |
| gene-BPHYT_RS01990 | 150.04369 | 113.08088 | 160.64158 | 289.874352 | 269.132414 | 230.87085 | -0.89465771 | 7.65E-06 | 1.95E-05 |
| gene-BPHYT_RS27010 | 1065.46982 | 923.84945 | 809.308212 | 1610.08441 | 1275.887 | 1479.38419 | -0.64103353 | 7.74E-06 | 1.98E-05 |
| gene-BPHYT_RS13560 | 36.7128177 | 58.6740413 | 19.3176583 | 127.189767 | 97.1867051 | 107.73973 | -1.54005685 | 7.78E-06 | 1.99E-05 |
| gene-BPHYT_RS22115 | 202.718602 | 203.758943 | 192.159864 | 80.8493089 | 132.07424 | 72.4300706 | 1.08239412 | 7.79E-06 | 1.99E-05 |
| gene-BPHYT_RS13680 | 976.880193 | 991.057897 | 927.2476 | 632.004964 | 529.542944 | 712.53082 | 0.62483316 | 7.87E-06 | 2.01E-05 |
| gene-BPHYT_RS17625 | 8100.76304 | 7653.22858 | 5624.48873 | 10328.0062 | 11299.8234 | 11324.4415 | -0.62407763 | 7.96E-06 | 2.03E-05 |
| gene-BPHYT_RS15275 | 21.5488278 | 24.5364173 | 28.4681281 | 0.98596718 | 1.2459834 | 0 | 5.16500837 | 8.14E-06 | 2.07E-05 |
| gene-BPHYT_RS08905 | 1901.08547 | 2246.68238 | 2243.89852 | 3020.01748 | 3182.2416 | 2901.72971 | -0.51066124 | 8.26E-06 | 2.10E-05 |
| gene-BPHYT_RS23485 | 292.904437 | 227.22856 | 176.909082 | 86.765112 | 114.630473 | 130.374127 | 1.07359269 | 8.29E-06 | 2.11E-05 |
| gene-BPHYT_RS14515 | 95.772568 | 162.153714 | 132.173452 | 261.281303 | 250.442663 | 240.829985 | -0.95537465 | 8.38E-06 | 2.13E-05 |
| gene-BPHYT_RS11535 | 465.29506 | 491.795146 | 453.456611 | 273.11291 | 322.7097 | 301.490169 | 0.65416402 | 8.39E-06 | 2.13E-05 |
| gene-BPHYT_RS10030 | 60.6559597 | 30.9372218 | 48.8025053 | 5.91580309 | 12.459834 | 12.6752624 | 2.18957984 | 8.40E-06 | 2.14E-05 |
| gene-BPHYT_RS25155 | 218.680697 | 195.224537 | 212.494242 | 333.256908 | 335.169534 | 354.00197 | -0.70577358 | 8.42E-06 | 2.14E-05 |
| gene-BPHYT_RS16850 | 561.067627 | 521.665567 | 403.637387 | 714.826207 | 885.894196 | 822.986678 | -0.70322794 | 8.76E-06 | 2.23E-05 |
| gene-BPHYT_RS06875 | 268.961295 | 277.368195 | 343.650974 | 540.310016 | 465.997791 | 454.498693 | -0.71670309 | 8.77E-06 | 2.23E-05 |
| gene-BPHYT_RS12245 | 1808.50533 | 2236.01437 | 2470.62683 | 1565.71589 | 1350.646 | 1265.71548 | 0.63899183 | 8.83E-06 | 2.24E-05 |
| gene-BPHYT_RS02340 | 573.837303 | 649.681657 | 667.984291 | 959.346068 | 939.471482 | 886.362989 | -0.55933179 | 8.94E-06 | 2.27E-05 |
| gene-BPHYT_RS08490 | 17.5583041 | 62.9412443 | 26.4346903 | 3.94386873 | 0 | 6.33763118 | 3.29326768 | 8.96E-06 | 2.27E-05 |
| gene-BPHYT_RS25095 | 315.25137 | 325.374229 | 323.316597 | 167.614421 | 216.801111 | 188.318184 | 0.75475 | 9.01E-06 | 2.28E-05 |
| gene-BPHYT_RS33740 | 248.210572 | 250.698176 | 258.24659 | 153.81088 | 139.550141 | 146.670893 | 0.78101622 | 9.10E-06 | 2.31E-05 |
| gene-BPHYT_RS17665 | 1211.52299 | 1430.57981 | 1195.66138 | 1940.38342 | 1941.24214 | 1721.11955 | -0.54607188 | 9.12E-06 | 2.31E-05 |
| gene-BPHYT_RS19680 | 2518.02043 | 2726.74272 | 2224.58086 | 1718.5408 | 1785.49421 | 1728.36256 | 0.51365734 | 9.19E-06 | 2.33E-05 |
| gene-BPHYT_RS13165 | 67.8389023 | 54.4068383 | 40.6687544 | 21.691278 | 6.22991699 | 9.05375883 | 2.12423898 | 9.30E-06 | 2.35E-05 |
| gene-BPHYT_RS10020 | 165.20768 | 114.14768 | 87.4378219 | 55.2141622 | 44.8554023 | 46.17417 | 1.32993027 | 9.38E-06 | 2.37E-05 |
| gene-BPHYT_RS04220 | 2331.26393 | 2372.56487 | 2341.50353 | 3532.72041 | 3038.95351 | 3197.78762 | -0.47195022 | 9.66E-06 | 2.44E-05 |
| gene-BPHYT_RS16050 | 307.270322 | 268.833789 | 235.878775 | 132.119602 | 174.437676 | 142.144014 | 0.86288976 | 9.69E-06 | 2.45E-05 |
| gene-BPHYT_RS05550 | 51.8768077 | 38.404827 | 41.6854732 | 111.414292 | 80.9889209 | 133.995631 | -1.30563435 | 9.88E-06 | 2.49E-05 |
| gene-BPHYT_RS14240 | 199.526183 | 168.554519 | 180.975957 | 98.5967182 | 95.9407217 | 98.6859712 | 0.90642207 | 1.00E-05 | 2.53E-05 |
| gene-BPHYT_RS17225 | 1380.72119 | 1345.23575 | 1580.99783 | 2155.32426 | 2134.36956 | 1907.62699 | -0.52480834 | 1.00E-05 | 2.53E-05 |
| gene-BPHYT_RS13890 | 244.220048 | 174.955323 | 176.909082 | 345.088514 | 387.500837 | 325.029942 | -0.82011067 | 1.02E-05 | 2.56E-05 |
| gene-BPHYT_RS32645 | 238.633315 | 201.625342 | 207.410647 | 344.102547 | 356.351252 | 357.623474 | -0.70489825 | 1.04E-05 | 2.63E-05 |
| gene-BPHYT_RS08005 | 1368.74962 | 1433.78021 | 1278.01561 | 1036.25151 | 857.236578 | 888.173741 | 0.55205905 | 1.07E-05 | 2.68E-05 |
| gene-BPHYT_RS01565 | 520.364286 | 508.863958 | 549.028184 | 799.619385 | 838.546827 | 712.53082 | -0.57351857 | 1.12E-05 | 2.82E-05 |
| gene-BPHYT_RS33580 | 2656.89066 | 2822.75479 | 2973.90266 | 2028.13449 | 1981.1136 | 2123.10645 | 0.46252016 | 1.13E-05 | 2.84E-05 |
| gene-BPHYT_RS12570 | 3176.45684 | 3498.03966 | 3557.49929 | 4646.86333 | 4578.98899 | 4673.55031 | -0.44230213 | 1.15E-05 | 2.88E-05 |
| gene-BPHYT_RS02795 | 1225.09077 | 1142.5436 | 1192.61122 | 730.601682 | 757.557906 | 911.713514 | 0.56810623 | 1.17E-05 | 2.94E-05 |
| gene-BPHYT_RS17960 | 1351.19131 | 1457.24983 | 1615.56627 | 1014.56023 | 1051.60999 | 1019.45324 | 0.5195003 | 1.19E-05 | 2.99E-05 |
| gene-BPHYT_RS08770 | 652.849672 | 650.748458 | 428.03864 | 327.341105 | 370.057069 | 284.288027 | 0.82204014 | 1.26E-05 | 3.18E-05 |
| gene-BPHYT_RS12955 | 2498.86592 | 2296.82202 | 2342.52025 | 1576.56152 | 1665.8798 | 1839.72379 | 0.4900567 | 1.27E-05 | 3.20E-05 |
| gene-BPHYT_RS13910 | 739.044983 | 601.675623 | 727.970703 | 450.587002 | 383.762887 | 478.943842 | 0.6539342 | 1.28E-05 | 3.22E-05 |
| gene-BPHYT_RS34255 | 410.225833 | 444.855913 | 387.369885 | 282.972581 | 194.37341 | 253.505247 | 0.76053509 | 1.29E-05 | 3.25E-05 |
| gene-BPHYT_RS24770 | 228.257954 | 150.418906 | 107.772199 | 359.878022 | 301.527982 | 338.61058 | -1.0340174 | 1.33E-05 | 3.33E-05 |
| gene-BPHYT_RS10900 | 287.317704 | 357.378251 | 271.463935 | 179.446027 | 181.913576 | 171.116042 | 0.78236222 | 1.34E-05 | 3.36E-05 |
| gene-BPHYT_RS12615 | 6464.64834 | 6820.0572 | 6391.09475 | 8695.24458 | 9157.97798 | 8466.16988 | -0.41964345 | 1.35E-05 | 3.38E-05 |
| gene-BPHYT_RS17545 | 17960.5489 | 24152.369 | 23244.2266 | 15882.9453 | 13551.3154 | 14469.7174 | 0.57386784 | 1.35E-05 | 3.38E-05 |
| gene-BPHYT_RS03920 | 353.560397 | 257.098981 | 246.045964 | 519.604705 | 454.78394 | 477.13309 | -0.75704118 | 1.35E-05 | 3.39E-05 |
| gene-BPHYT_RS22705 | 350.367978 | 288.036203 | 264.346903 | 470.306346 | 474.719675 | 510.631998 | -0.68650063 | 1.39E-05 | 3.48E-05 |
| gene-BPHYT_RS18315 | 52.6749124 | 61.8744435 | 42.7021921 | 132.119602 | 109.646539 | 107.73973 | -1.15423641 | 1.40E-05 | 3.51E-05 |
| gene-BPHYT_RS13425 | 27.9336657 | 32.0040225 | 19.3176583 | 0 | 0 | 5.4322553 | 3.74220563 | 1.41E-05 | 3.52E-05 |
| gene-BPHYT_RS12765 | 563.461942 | 625.14524 | 593.763814 | 1092.45164 | 814.873143 | 858.296337 | -0.63535904 | 1.42E-05 | 3.55E-05 |
| gene-BPHYT_RS37260 | 4885.19907 | 5266.7953 | 6190.80113 | 7990.27805 | 7730.081 | 7486.55318 | -0.50607297 | 1.42E-05 | 3.55E-05 |
| gene-BPHYT_RS28095 | 79.0123686 | 124.815688 | 86.421103 | 191.277633 | 195.619394 | 183.791304 | -0.98172362 | 1.45E-05 | 3.61E-05 |
| gene-BPHYT_RS34470 | 61.4540645 | 82.1436578 | 75.2371956 | 131.133635 | 145.780058 | 168.399914 | -1.03258926 | 1.47E-05 | 3.68E-05 |
| gene-BPHYT_RS08765 | 541.913114 | 528.066371 | 554.111778 | 308.607728 | 356.351252 | 384.78475 | 0.62936473 | 1.49E-05 | 3.71E-05 |
| gene-BPHYT_RS10685 | 210.69965 | 236.829767 | 235.878775 | 119.302029 | 117.122439 | 144.860141 | 0.83618648 | 1.49E-05 | 3.72E-05 |
| gene-BPHYT_RS06535 | 1409.45296 | 1616.20314 | 1902.28099 | 1085.54987 | 980.588935 | 1165.21876 | 0.6072837 | 1.50E-05 | 3.73E-05 |
| gene-BPHYT_RS14410 | 53.4730171 | 101.346071 | 66.0867259 | 176.488126 | 183.15956 | 133.995631 | -1.16678329 | 1.54E-05 | 3.83E-05 |
| gene-BPHYT_RS16080 | 1022.37216 | 1136.1428 | 1150.92575 | 1583.4633 | 1530.06761 | 1516.5046 | -0.48530223 | 1.54E-05 | 3.83E-05 |
| gene-BPHYT_RS03500 | 1685.5972 | 1538.32668 | 1278.01561 | 2246.03324 | 2108.20391 | 2255.29133 | -0.55354829 | 1.55E-05 | 3.86E-05 |
| gene-BPHYT_RS17065 | 453.323489 | 571.805202 | 478.874583 | 302.691925 | 336.415518 | 309.638552 | 0.66460017 | 1.57E-05 | 3.89E-05 |
| gene-BPHYT_RS30560 | 386.282691 | 393.649477 | 368.052227 | 252.407599 | 235.490862 | 243.546113 | 0.64937001 | 1.63E-05 | 4.04E-05 |
| gene-BPHYT_RS19940 | 188.352717 | 222.961357 | 178.942519 | 103.526554 | 98.4326885 | 115.888113 | 0.88810754 | 1.64E-05 | 4.08E-05 |
| gene-BPHYT_RS24995 | 43.0976556 | 68.275248 | 59.9864127 | 17.7474093 | 18.689751 | 16.2967659 | 1.69873041 | 1.65E-05 | 4.08E-05 |
| gene-BPHYT_RS32630 | 69.4351118 | 57.6072405 | 70.1536013 | 130.147668 | 133.320224 | 135.806382 | -1.01474606 | 1.65E-05 | 4.08E-05 |
| gene-BPHYT_RS15905 | 12501.5125 | 13278.4689 | 16110.927 | 10486.747 | 9388.48491 | 7996.2798 | 0.58784707 | 1.67E-05 | 4.14E-05 |
| gene-BPHYT_RS07275 | 47.0881793 | 59.740842 | 53.8860995 | 129.161701 | 104.662606 | 112.26661 | -1.11427609 | 1.69E-05 | 4.18E-05 |
| gene-BPHYT_RS29420 | 142.062643 | 164.287316 | 328.400192 | 68.0317356 | 103.416622 | 84.1999571 | 1.31422558 | 1.69E-05 | 4.19E-05 |
| gene-BPHYT_RS04390 | 134.081595 | 142.951301 | 151.49111 | 256.351467 | 250.442663 | 227.249347 | -0.77732299 | 1.73E-05 | 4.29E-05 |
| gene-BPHYT_RS23260 | 2094.22682 | 2295.75521 | 2364.88807 | 3082.13341 | 3350.44936 | 2946.09312 | -0.47344244 | 1.77E-05 | 4.38E-05 |
| gene-BPHYT_RS11290 | 577.029722 | 545.135183 | 649.683351 | 841.030007 | 928.257632 | 843.810323 | -0.55970221 | 1.77E-05 | 4.39E-05 |
| gene-BPHYT_RS07230 | 216.286383 | 211.226549 | 248.079402 | 414.106217 | 328.939617 | 364.866481 | -0.7160124 | 1.86E-05 | 4.61E-05 |
| gene-BPHYT_RS13990 | 175.583041 | 214.426951 | 184.026114 | 310.579662 | 302.773966 | 330.462197 | -0.72130041 | 1.88E-05 | 4.65E-05 |
| gene-BPHYT_RS07180 | 522.7586 | 534.467176 | 536.827558 | 313.537564 | 393.730754 | 325.029942 | 0.6301577 | 1.90E-05 | 4.70E-05 |
| gene-BPHYT_RS13900 | 579.424036 | 381.914669 | 419.904889 | 220.856649 | 289.068148 | 273.423517 | 0.82245004 | 1.93E-05 | 4.76E-05 |
| gene-BPHYT_RS24520 | 1763.01336 | 1778.35685 | 1745.70628 | 2364.3493 | 2450.84935 | 2312.33001 | -0.43065793 | 1.93E-05 | 4.77E-05 |
| gene-BPHYT_RS07765 | 1050.30583 | 965.454679 | 987.234012 | 630.03303 | 671.585052 | 757.799614 | 0.54387085 | 1.95E-05 | 4.81E-05 |
| gene-BPHYT_RS17005 | 2769.42342 | 3295.34752 | 3383.64036 | 2311.10708 | 2251.492 | 1865.07432 | 0.55584448 | 1.95E-05 | 4.82E-05 |
| gene-BPHYT_RS17500 | 731.86204 | 712.622901 | 740.17133 | 984.981215 | 1046.62606 | 1041.18227 | -0.49178485 | 1.97E-05 | 4.85E-05 |
| gene-BPHYT_RS20665 | 141.264538 | 119.481684 | 200.293615 | 314.523531 | 281.592248 | 264.369758 | -0.90084062 | 1.97E-05 | 4.87E-05 |
| gene-BPHYT_RS12885 | 54.2711219 | 37.3380263 | 38.6353167 | 80.8493089 | 118.368423 | 116.793489 | -1.26701914 | 1.98E-05 | 4.89E-05 |
| gene-BPHYT_RS15950 | 375.907329 | 347.777045 | 268.413779 | 193.249568 | 205.587261 | 178.359049 | 0.78544157 | 2.01E-05 | 4.95E-05 |
| gene-BPHYT_RS15165 | 408.629623 | 475.793135 | 457.523487 | 597.496112 | 794.937408 | 702.571685 | -0.64255958 | 2.02E-05 | 4.97E-05 |
| gene-BPHYT_RS24070 | 60.6559597 | 56.5404398 | 34.5684412 | 11.8316062 | 9.96786719 | 18.1075177 | 1.91139711 | 2.05E-05 | 5.04E-05 |
| gene-BPHYT_RS35690 | 153.236109 | 119.481684 | 155.557986 | 78.8773746 | 69.7750703 | 40.7419147 | 1.1858501 | 2.09E-05 | 5.14E-05 |
| gene-BPHYT_RS29560 | 107.744139 | 71.4756503 | 102.688605 | 22.6772452 | 46.1013857 | 38.0257871 | 1.4137815 | 2.11E-05 | 5.20E-05 |
| gene-BPHYT_RS04525 | 1094.20159 | 896.11263 | 371.102384 | 68.0317356 | 139.550141 | 192.845063 | 2.55989431 | 2.12E-05 | 5.21E-05 |
| gene-BPHYT_RS02360 | 169.198203 | 144.018101 | 154.541267 | 245.505828 | 286.576182 | 262.559006 | -0.75985396 | 2.13E-05 | 5.23E-05 |
| gene-BPHYT_RS28320 | 572.241094 | 525.93277 | 623.248661 | 389.457037 | 371.303053 | 382.068623 | 0.59062965 | 2.18E-05 | 5.36E-05 |
| gene-BPHYT_RS35605 | 124.504338 | 118.414883 | 111.839075 | 186.347797 | 213.063161 | 235.39773 | -0.83852215 | 2.21E-05 | 5.43E-05 |
| gene-BPHYT_RS02890 | 2871.58083 | 3153.46302 | 2815.29452 | 3833.44041 | 4164.07652 | 4010.81516 | -0.44189286 | 2.26E-05 | 5.53E-05 |
| gene-BPHYT_RS06570 | 5704.85263 | 6603.49664 | 6757.11354 | 8532.56 | 8807.85664 | 8703.37836 | -0.45017985 | 2.29E-05 | 5.60E-05 |
| gene-BPHYT_RS16775 | 1130.1163 | 1186.28243 | 1234.2967 | 1661.3547 | 1657.15792 | 1569.01641 | -0.46121914 | 2.29E-05 | 5.62E-05 |
| gene-BPHYT_RS06055 | 4244.32097 | 4607.51244 | 3958.08652 | 3009.17184 | 3281.92027 | 3035.72534 | 0.45803087 | 2.31E-05 | 5.66E-05 |
| gene-BPHYT_RS14560 | 551.490371 | 397.91668 | 479.891302 | 325.36917 | 272.870364 | 267.085886 | 0.72553982 | 2.32E-05 | 5.67E-05 |
| gene-BPHYT_RS02425 | 134.8797 | 114.14768 | 150.474391 | 233.674222 | 218.047095 | 250.78912 | -0.8149582 | 2.34E-05 | 5.72E-05 |
| gene-BPHYT_RS24805 | 200.324288 | 246.430973 | 228.761743 | 143.951209 | 118.368423 | 113.171985 | 0.84418894 | 2.34E-05 | 5.73E-05 |
| gene-BPHYT_RS21450 | 47.886284 | 73.6092518 | 68.1201636 | 14.7895077 | 28.6576182 | 17.2021418 | 1.66106271 | 2.37E-05 | 5.79E-05 |
| gene-BPHYT_RS12580 | 5021.67498 | 5818.33129 | 5650.92342 | 7475.60318 | 7399.8954 | 7394.20484 | -0.43366499 | 2.43E-05 | 5.93E-05 |
| gene-BPHYT_RS16515 | 395.061843 | 368.046259 | 414.821295 | 256.351467 | 230.506929 | 262.559006 | 0.65020925 | 2.46E-05 | 6.01E-05 |
| gene-BPHYT_RS33830 | 441.351918 | 515.264762 | 558.178654 | 748.349091 | 756.311923 | 751.461983 | -0.57665948 | 2.50E-05 | 6.09E-05 |
| gene-BPHYT_RS08795 | 161.217156 | 138.684098 | 451.423174 | 1183.16062 | 1121.38506 | 1358.9692 | -2.28627888 | 2.52E-05 | 6.16E-05 |
| gene-BPHYT_RS19450 | 74.2237402 | 102.412872 | 71.1703202 | 201.137305 | 139.550141 | 162.062283 | -1.02785477 | 2.53E-05 | 6.16E-05 |
| gene-BPHYT_RS16165 | 5684.90002 | 6418.94011 | 5445.54621 | 8033.6606 | 7688.96355 | 8206.327 | -0.44753435 | 2.56E-05 | 6.25E-05 |
| gene-BPHYT_RS15135 | 239.43142 | 355.24465 | 303.998939 | 482.137952 | 482.195575 | 502.483615 | -0.71120116 | 2.57E-05 | 6.26E-05 |
| gene-BPHYT_RS06770 | 2503.65455 | 2559.255 | 2095.45757 | 3165.94062 | 3394.05878 | 3563.55948 | -0.49991507 | 2.62E-05 | 6.38E-05 |
| gene-BPHYT_RS28300 | 598.57855 | 624.078439 | 698.485856 | 458.47474 | 411.174522 | 414.662154 | 0.57963204 | 2.63E-05 | 6.41E-05 |
| gene-BPHYT_RS03505 | 1990.47321 | 1757.02084 | 1850.42832 | 2562.52871 | 2422.19173 | 2784.93622 | -0.47293411 | 2.66E-05 | 6.49E-05 |
| gene-BPHYT_RS25960 | 82.2047875 | 72.542451 | 77.2706333 | 18.7333765 | 7.47590039 | 38.931163 | 1.80051072 | 2.67E-05 | 6.49E-05 |
| gene-BPHYT_RS20655 | 54.2711219 | 97.0788683 | 46.7690675 | 16.7614421 | 14.9518008 | 26.2559006 | 1.75406391 | 2.69E-05 | 6.55E-05 |
| gene-BPHYT_RS16160 | 191.545136 | 157.886511 | 248.079402 | 86.765112 | 94.6947383 | 118.604241 | 0.99056971 | 2.79E-05 | 6.78E-05 |
| gene-BPHYT_RS04880 | 106.946034 | 119.481684 | 159.624861 | 195.221502 | 251.688647 | 294.247162 | -0.94494263 | 2.82E-05 | 6.84E-05 |
| gene-BPHYT_RS20730 | 55.0692266 | 65.0748458 | 62.0198504 | 142.965241 | 127.090307 | 106.834354 | -1.0514358 | 2.86E-05 | 6.93E-05 |
| gene-BPHYT_RS21895 | 1538.74593 | 1614.06954 | 1413.23921 | 1173.30095 | 1003.01664 | 1034.84463 | 0.50727494 | 2.89E-05 | 7.03E-05 |
| gene-BPHYT_RS18750 | 43.0976556 | 37.3380263 | 32.5350035 | 77.8914074 | 119.614406 | 81.4838295 | -1.29173473 | 2.92E-05 | 7.09E-05 |
| gene-BPHYT_RS33920 | 11192.6208 | 12223.403 | 10667.4143 | 14831.9043 | 15561.0867 | 15138.7901 | -0.4178146 | 2.93E-05 | 7.10E-05 |
| gene-BPHYT_RS01445 | 1191.57037 | 984.657093 | 1117.37403 | 764.124566 | 755.065939 | 777.717884 | 0.52066331 | 2.94E-05 | 7.13E-05 |
| gene-BPHYT_RS16905 | 896.271615 | 841.705792 | 654.766945 | 1297.53281 | 1193.6521 | 1139.86824 | -0.60070851 | 2.95E-05 | 7.14E-05 |
| gene-BPHYT_RS23085 | 75.0218449 | 89.611263 | 88.4545408 | 147.895077 | 180.667593 | 152.103148 | -0.92628984 | 3.03E-05 | 7.35E-05 |
| gene-BPHYT_RS33455 | 944.157899 | 875.843416 | 906.913223 | 616.229489 | 661.617185 | 652.776012 | 0.49916652 | 3.10E-05 | 7.50E-05 |
| gene-BPHYT_RS15270 | 22.3469325 | 25.603218 | 24.4012526 | 0.98596718 | 0 | 5.4322553 | 3.38265023 | 3.10E-05 | 7.51E-05 |
| gene-BPHYT_RS11395 | 540.316904 | 547.268785 | 446.339579 | 335.228842 | 269.132414 | 352.191219 | 0.67811204 | 3.15E-05 | 7.62E-05 |
| gene-BPHYT_RS12325 | 582.616455 | 508.863958 | 555.128497 | 391.428971 | 322.7097 | 366.677233 | 0.60605675 | 3.20E-05 | 7.74E-05 |
| gene-BPHYT_RS18935 | 771.767277 | 897.179431 | 700.519294 | 1041.18134 | 1360.61387 | 1209.58218 | -0.60757469 | 3.22E-05 | 7.79E-05 |
| gene-BPHYT_RS25990 | 20.7507231 | 14.9352105 | 25.4179715 | 0 | 1.2459834 | 1.81075177 | 4.31493993 | 3.26E-05 | 7.86E-05 |
| gene-BPHYT_RS30200 | 125.302443 | 134.416895 | 129.123295 | 72.9615715 | 66.0371201 | 54.322553 | 1.01041373 | 3.27E-05 | 7.89E-05 |
| gene-BPHYT_RS14835 | 2059.11021 | 2484.57895 | 2458.4262 | 1736.28821 | 1571.18507 | 1652.31099 | 0.49656905 | 3.28E-05 | 7.91E-05 |
| gene-BPHYT_RS12280 | 7929.96863 | 16413.7963 | 20813.2518 | 5262.10685 | 5624.36906 | 4457.16547 | 1.55730765 | 3.30E-05 | 7.96E-05 |
| gene-BPHYT_RS09240 | 260.980248 | 361.645454 | 335.517224 | 212.968911 | 170.699726 | 163.873035 | 0.80378357 | 3.32E-05 | 8.00E-05 |
| gene-BPHYT_RS09990 | 130.091072 | 168.554519 | 177.9258 | 304.663859 | 250.442663 | 267.991261 | -0.79454525 | 3.49E-05 | 8.41E-05 |
| gene-BPHYT_RS13285 | 329.617255 | 292.303406 | 325.350035 | 443.685232 | 560.692529 | 473.511587 | -0.63827466 | 3.50E-05 | 8.42E-05 |
| gene-BPHYT_RS14770 | 247.412467 | 246.430973 | 196.22674 | 136.063471 | 110.892523 | 140.333262 | 0.82924023 | 3.54E-05 | 8.53E-05 |
| gene-BPHYT_RS20575 | 15.1639899 | 17.068812 | 11.1839075 | 44.3685232 | 47.3473691 | 50.7010495 | -1.71382286 | 3.58E-05 | 8.62E-05 |
| gene-BPHYT_RS07945 | 472.478002 | 522.732368 | 517.509899 | 359.878022 | 257.918564 | 331.367573 | 0.66764508 | 3.65E-05 | 8.77E-05 |
| gene-BPHYT_RS00155 | 197.131869 | 386.181872 | 259.263309 | 138.035406 | 160.731858 | 115.888113 | 1.02359853 | 3.65E-05 | 8.78E-05 |
| gene-BPHYT_RS23280 | 98.1668822 | 88.5444623 | 88.4545408 | 40.4246545 | 44.8554023 | 38.0257871 | 1.16512993 | 3.65E-05 | 8.78E-05 |
| gene-BPHYT_RS07195 | 254.59541 | 347.777045 | 444.306142 | 693.134929 | 586.858181 | 545.941657 | -0.80578382 | 3.67E-05 | 8.81E-05 |
| gene-BPHYT_RS31180 | 421.399299 | 353.111048 | 326.366754 | 189.305699 | 186.89751 | 261.65363 | 0.78503654 | 3.67E-05 | 8.81E-05 |
| gene-BPHYT_RS19880 | 166.803889 | 119.481684 | 108.788918 | 67.0457684 | 61.0531865 | 58.8494324 | 1.0845787 | 3.72E-05 | 8.94E-05 |
| gene-BPHYT_RS19555 | 2646.5153 | 2776.88235 | 2669.90372 | 3429.19386 | 3620.82776 | 3702.08199 | -0.40995943 | 3.75E-05 | 8.99E-05 |
| gene-BPHYT_RS03130 | 1788.55271 | 1935.17656 | 1910.41474 | 1309.36442 | 1475.24434 | 1301.93052 | 0.46392843 | 3.75E-05 | 9.00E-05 |
| gene-BPHYT_RS13410 | 148.44748 | 89.611263 | 77.2706333 | 39.4386873 | 31.149585 | 50.7010495 | 1.37413523 | 3.80E-05 | 9.10E-05 |
| gene-BPHYT_RS24745 | 121.311919 | 150.418906 | 135.223608 | 69.0177028 | 47.3473691 | 76.9569501 | 1.05981553 | 3.80E-05 | 9.10E-05 |
| gene-BPHYT_RS15380 | 913.82992 | 1048.66514 | 928.264319 | 649.752373 | 727.654305 | 619.277104 | 0.53489898 | 3.87E-05 | 9.27E-05 |
| gene-BPHYT_RS26685 | 133.28349 | 134.416895 | 142.34064 | 74.9335059 | 72.2670371 | 66.0924395 | 0.94408242 | 3.91E-05 | 9.36E-05 |
| gene-BPHYT_RS19000 | 10443.9985 | 9478.52467 | 8693.96297 | 6540.90629 | 7558.13529 | 6406.43975 | 0.48117552 | 3.98E-05 | 9.52E-05 |
| gene-BPHYT_RS29775 | 983.265031 | 355.24465 | 695.4357 | 318.4674 | 342.645435 | 310.543928 | 1.06812174 | 4.01E-05 | 9.58E-05 |
| gene-BPHYT_RS18770 | 1185.18553 | 1027.32912 | 967.916354 | 1514.44559 | 1765.55848 | 1403.33262 | -0.55639134 | 4.01E-05 | 9.58E-05 |
| gene-BPHYT_RS32620 | 118.119501 | 114.14768 | 195.210021 | 279.028713 | 250.442663 | 275.234268 | -0.91619185 | 4.02E-05 | 9.61E-05 |
| gene-BPHYT_RS05520 | 1000.82334 | 788.365754 | 902.846347 | 1226.54318 | 1293.33077 | 1384.31973 | -0.53517813 | 4.12E-05 | 9.85E-05 |
| gene-BPHYT_RS28185 | 114.927082 | 180.289327 | 220.627993 | 89.7230136 | 49.8393359 | 83.2945812 | 1.19801784 | 4.15E-05 | 9.90E-05 |
| gene-BPHYT_RS10875 | 46.2900745 | 36.2712255 | 35.5851601 | 8.87370464 | 6.22991699 | 13.5806382 | 2.01865957 | 4.16E-05 | 9.93E-05 |
| gene-BPHYT_RS08090 | 2819.70402 | 2550.72059 | 2524.51293 | 1902.91666 | 2052.13466 | 1814.37327 | 0.45347544 | 4.17E-05 | 9.94E-05 |
| gene-BPHYT_RS08285 | 147.649376 | 163.220515 | 124.039701 | 67.0457684 | 72.2670371 | 82.3892054 | 0.96966077 | 4.22E-05 | 0.00010058 |
| gene-BPHYT_RS18325 | 2784.58741 | 2773.68195 | 2409.6237 | 3478.49222 | 3564.7585 | 3957.39799 | -0.46534811 | 4.22E-05 | 0.00010058 |
| gene-BPHYT_RS33545 | 2131.73774 | 2232.81397 | 1903.29771 | 3073.25971 | 2901.89534 | 2696.20938 | -0.4681482 | 4.31E-05 | 0.00010259 |
| gene-BPHYT_RS02380 | 1514.80278 | 1322.83293 | 1396.97171 | 981.037346 | 1046.62606 | 1028.507 | 0.47150038 | 4.34E-05 | 0.00010327 |
| gene-BPHYT_RS02465 | 25.5393515 | 28.8036203 | 21.351096 | 74.9335059 | 48.5933525 | 84.1999571 | -1.46508753 | 4.38E-05 | 0.00010426 |
| gene-BPHYT_RS14275 | 1040.72857 | 1298.29651 | 1120.42418 | 655.668176 | 740.114139 | 878.214607 | 0.60381188 | 4.43E-05 | 0.00010532 |
| gene-BPHYT_RS02045 | 173.986832 | 167.487718 | 170.808768 | 264.239205 | 276.608314 | 283.382651 | -0.68550259 | 4.45E-05 | 0.00010583 |
| gene-BPHYT_RS33860 | 4183.66501 | 4409.0875 | 4268.18577 | 5601.27956 | 5557.08596 | 5613.33047 | -0.38317648 | 4.45E-05 | 0.00010584 |
| gene-BPHYT_RS26160 | 30.3279799 | 30.9372218 | 36.6018789 | 80.8493089 | 87.2188379 | 67.9031912 | -1.26842047 | 4.46E-05 | 0.00010604 |
| gene-BPHYT_RS09890 | 81.4066828 | 67.2084473 | 82.3542276 | 28.5930483 | 39.8714688 | 23.539773 | 1.34645959 | 4.49E-05 | 0.0001065 |
| gene-BPHYT_RS01500 | 2713.55609 | 2843.024 | 3365.33942 | 2184.90328 | 2158.04325 | 2025.32585 | 0.48635735 | 4.59E-05 | 0.00010887 |
| gene-BPHYT_RS22835 | 10.3753615 | 2.1336015 | 3.05015658 | 33.5228842 | 33.6415518 | 27.1612765 | -2.53394842 | 4.61E-05 | 0.00010946 |
| gene-BPHYT_RS14985 | 655.243986 | 435.254706 | 584.613344 | 253.393566 | 383.762887 | 332.272949 | 0.79306609 | 4.65E-05 | 0.00011026 |
| gene-BPHYT_RS09795 | 707.120794 | 717.956905 | 487.008334 | 948.500429 | 1067.80777 | 990.481216 | -0.65188582 | 4.66E-05 | 0.00011047 |
| gene-BPHYT_RS09555 | 1923.43241 | 1823.16248 | 1350.20265 | 1199.92206 | 1082.75957 | 1082.82956 | 0.59906337 | 4.71E-05 | 0.00011165 |
| gene-BPHYT_RS29515 | 122.908129 | 114.14768 | 104.722043 | 185.36183 | 232.998896 | 190.128935 | -0.82616957 | 4.75E-05 | 0.00011249 |
| gene-BPHYT_RS01790 | 683.975756 | 899.313032 | 641.5496 | 1080.62003 | 1065.31581 | 1311.88965 | -0.63789609 | 4.81E-05 | 0.00011388 |
| gene-BPHYT_RS17580 | 2037.56138 | 2161.33832 | 1898.21411 | 1395.14356 | 1543.77343 | 1508.35622 | 0.45546304 | 4.84E-05 | 0.00011448 |
| gene-BPHYT_RS08315 | 473.276107 | 477.926736 | 452.439892 | 327.341105 | 312.741833 | 305.111673 | 0.57068841 | 4.89E-05 | 0.00011568 |
| gene-BPHYT_RS11305 | 63.8483787 | 44.8056315 | 59.9864127 | 24.6491796 | 13.7058174 | 16.2967659 | 1.61921903 | 5.01E-05 | 0.00011833 |
| gene-BPHYT_RS17700 | 1790.94702 | 1832.76369 | 1667.41893 | 2320.96675 | 2541.80613 | 2306.89775 | -0.43782043 | 5.04E-05 | 0.00011915 |
| gene-BPHYT_RS22130 | 114.128977 | 129.082891 | 170.808768 | 71.9756043 | 71.0210537 | 58.8494324 | 1.03698782 | 5.09E-05 | 0.00012016 |
| gene-BPHYT_RS28020 | 368.724387 | 408.584687 | 380.252853 | 229.730354 | 256.67258 | 263.464382 | 0.62564701 | 5.11E-05 | 0.00012075 |
| gene-BPHYT_RS13465 | 237.835211 | 224.028158 | 204.360491 | 342.130612 | 336.415518 | 362.150353 | -0.64258777 | 5.13E-05 | 0.00012112 |
| gene-BPHYT_RS06470 | 173.986832 | 161.086913 | 227.745025 | 107.470423 | 105.908589 | 87.8214607 | 0.90426005 | 5.30E-05 | 0.00012499 |
| gene-BPHYT_RS04870 | 28.7317704 | 27.7368195 | 29.4848469 | 65.073834 | 79.7429375 | 67.9031912 | -1.30327756 | 5.31E-05 | 0.00012518 |
| gene-BPHYT_RS15105 | 158.822842 | 178.155725 | 225.711587 | 316.495466 | 312.741833 | 305.111673 | -0.7347495 | 5.34E-05 | 0.00012586 |
| gene-BPHYT_RS22360 | 4976.98112 | 5108.90879 | 4249.88483 | 3194.53367 | 3735.45823 | 3363.47141 | 0.47828702 | 5.35E-05 | 0.00012615 |
| gene-BPHYT_RS15420 | 494.824935 | 587.807213 | 702.552732 | 391.428971 | 398.714688 | 325.935318 | 0.677902 | 5.39E-05 | 0.00012699 |
| gene-BPHYT_RS20100 | 85.3972065 | 67.2084473 | 70.1536013 | 203.10924 | 125.844323 | 133.090255 | -1.05019532 | 5.42E-05 | 0.00012765 |
| gene-BPHYT_RS01930 | 703.928375 | 640.08045 | 560.212092 | 943.570593 | 979.342951 | 861.917841 | -0.54625657 | 5.51E-05 | 0.00012975 |
| gene-BPHYT_RS15500 | 1109.36558 | 1291.89571 | 1734.52237 | 1000.75669 | 788.707491 | 724.300706 | 0.71759374 | 5.52E-05 | 0.00012977 |
| gene-BPHYT_RS15040 | 51.8768077 | 35.2044248 | 38.6353167 | 85.7791449 | 107.154572 | 93.253716 | -1.17332805 | 5.55E-05 | 0.00013048 |
| gene-BPHYT_RS13065 | 268.961295 | 273.100992 | 212.494242 | 351.004317 | 492.163442 | 409.229899 | -0.72770509 | 5.59E-05 | 0.00013139 |
| gene-BPHYT_RS21495 | 136.475909 | 162.153714 | 129.123295 | 88.7370464 | 61.0531865 | 63.3763118 | 0.99984725 | 5.77E-05 | 0.00013545 |
| gene-BPHYT_RS10635 | 31.9241893 | 22.4028158 | 44.7356298 | 64.0878669 | 89.7108047 | 104.118227 | -1.37975882 | 5.80E-05 | 0.0001361 |
| gene-BPHYT_RS09590 | 716.69805 | 685.952882 | 684.251792 | 1008.64443 | 951.931316 | 935.253287 | -0.47215627 | 5.81E-05 | 0.00013639 |
| gene-BPHYT_RS12110 | 2215.53874 | 2660.60107 | 2571.282 | 3535.67832 | 3222.11307 | 3481.17027 | -0.46005341 | 5.83E-05 | 0.00013682 |
| gene-BPHYT_RS11265 | 4843.69763 | 5149.44722 | 4276.31952 | 3132.41774 | 3329.26764 | 3752.78304 | 0.48212586 | 5.85E-05 | 0.00013706 |
| gene-BPHYT_RS05095 | 300.08738 | 272.034191 | 272.480654 | 486.081821 | 363.827152 | 496.145984 | -0.67414814 | 5.85E-05 | 0.0001371 |
| gene-BPHYT_RS35275 | 177.977356 | 153.619308 | 157.591423 | 233.674222 | 309.003883 | 280.666524 | -0.74624557 | 5.92E-05 | 0.0001386 |
| gene-BPHYT_RS10255 | 59.857855 | 55.473639 | 73.2037579 | 110.428324 | 125.844323 | 143.04939 | -1.01049386 | 5.93E-05 | 0.00013877 |
| gene-BPHYT_RS16750 | 158.822842 | 113.08088 | 196.22674 | 86.765112 | 73.5130205 | 66.0924395 | 1.04988708 | 5.98E-05 | 0.00013988 |
| gene-BPHYT_RS06585 | 1293.72777 | 1442.31461 | 1259.71467 | 1701.77936 | 1891.4028 | 1940.22052 | -0.47001385 | 5.98E-05 | 0.00013988 |
| gene-BPHYT_RS24765 | 270.557505 | 267.766988 | 242.995807 | 372.695595 | 418.650422 | 401.081516 | -0.60816164 | 6.14E-05 | 0.00014354 |
| gene-BPHYT_RS09210 | 631.300844 | 615.544033 | 555.128497 | 452.558937 | 370.057069 | 382.068623 | 0.58025634 | 6.27E-05 | 0.00014657 |
| gene-BPHYT_RS19625 | 313.65516 | 364.845857 | 427.021921 | 604.397883 | 508.361227 | 609.317969 | -0.64323566 | 6.32E-05 | 0.00014761 |
| gene-BPHYT_RS20365 | 189.948927 | 153.619308 | 181.992676 | 309.593695 | 281.592248 | 267.991261 | -0.70610685 | 6.33E-05 | 0.00014782 |
| gene-BPHYT_RS06610 | 3433.44656 | 3530.04368 | 3962.1534 | 5304.50344 | 4841.89149 | 4684.41482 | -0.44105684 | 6.36E-05 | 0.00014851 |
| gene-BPHYT_RS05750 | 3.99052367 | 6.4008045 | 6.10031316 | 31.5509498 | 28.6576182 | 23.539773 | -2.36217129 | 6.40E-05 | 0.00014936 |
| gene-BPHYT_RS16710 | 492.43062 | 512.06436 | 481.924739 | 345.088514 | 307.757899 | 350.380467 | 0.56467183 | 6.46E-05 | 0.00015077 |
| gene-BPHYT_RS12920 | 482.055259 | 529.133172 | 554.111778 | 344.102547 | 330.185601 | 375.730991 | 0.57306443 | 6.49E-05 | 0.00015137 |
| gene-BPHYT_RS13965 | 1568.2758 | 1477.51904 | 1087.88918 | 903.145939 | 940.717466 | 896.322124 | 0.59404791 | 6.56E-05 | 0.00015277 |
| gene-BPHYT_RS19790 | 1737.474 | 1869.03491 | 1811.79301 | 1399.08743 | 1335.6942 | 1287.44451 | 0.42961681 | 6.56E-05 | 0.00015289 |
| gene-BPHYT_RS10945 | 162.015261 | 112.014079 | 149.457672 | 70.0036699 | 59.8072031 | 80.5784536 | 1.00598533 | 6.57E-05 | 0.00015294 |
| gene-BPHYT_RS15390 | 4617.83399 | 4958.48989 | 5083.5943 | 6285.54079 | 6400.61672 | 6502.40959 | -0.38856678 | 6.58E-05 | 0.00015306 |
| gene-BPHYT_RS29185 | 340.790721 | 324.307428 | 356.86832 | 518.618738 | 525.804994 | 468.079332 | -0.56421191 | 6.58E-05 | 0.0001531 |
| gene-BPHYT_RS32680 | 190.747031 | 203.758943 | 208.427366 | 92.6809151 | 142.042107 | 95.0644677 | 0.87959684 | 6.60E-05 | 0.00015343 |
| gene-BPHYT_RS30545 | 243.421944 | 202.692143 | 224.694868 | 70.0036699 | 127.090307 | 141.238638 | 0.98970454 | 6.61E-05 | 0.00015358 |
| gene-BPHYT_RS01795 | 1193.16658 | 1301.49692 | 1411.20578 | 1977.85017 | 1618.53243 | 1971.90867 | -0.51299088 | 6.65E-05 | 0.00015443 |
| gene-BPHYT_RS01525 | 141.264538 | 169.621319 | 184.026114 | 283.958549 | 276.608314 | 255.315999 | -0.72442433 | 6.68E-05 | 0.00015504 |
| gene-BPHYT_RS34605 | 102.955511 | 129.082891 | 89.4712596 | 182.403929 | 208.079228 | 188.318184 | -0.84886801 | 6.71E-05 | 0.0001558 |
| gene-BPHYT_RS03160 | 803.691466 | 816.102574 | 716.786796 | 1201.894 | 1074.03769 | 1032.12851 | -0.50139155 | 6.86E-05 | 0.00015927 |
| gene-BPHYT_RS20855 | 35.914713 | 28.8036203 | 36.6018789 | 94.6528495 | 78.4969541 | 67.9031912 | -1.24588693 | 6.90E-05 | 0.00015997 |
| gene-BPHYT_RS37185 | 1570.67012 | 1349.50295 | 1180.4106 | 2016.30289 | 1877.69698 | 1977.34093 | -0.5171321 | 6.90E-05 | 0.00016002 |
| gene-BPHYT_RS28125 | 8581.22209 | 11320.8896 | 11665.8322 | 7721.109 | 7225.45773 | 7031.14911 | 0.52225437 | 6.99E-05 | 0.00016208 |
| gene-BPHYT_RS10510 | 110.138453 | 149.352105 | 58.9696938 | 46.3404576 | 26.1656514 | 43.4580424 | 1.44602266 | 7.10E-05 | 0.00016447 |
| gene-BPHYT_RS15780 | 1351.98942 | 1221.48686 | 1263.78154 | 867.65112 | 903.337964 | 994.10272 | 0.47272311 | 7.18E-05 | 0.00016635 |
| gene-BPHYT_RS02480 | 262.576457 | 265.633387 | 305.015658 | 429.881692 | 421.142389 | 406.513772 | -0.59460304 | 7.27E-05 | 0.00016815 |
| gene-BPHYT_RS15835 | 394.263738 | 305.105015 | 296.881907 | 523.548574 | 478.457625 | 550.468537 | -0.63779542 | 7.38E-05 | 0.00017079 |
| gene-BPHYT_RS14635 | 153.236109 | 178.155725 | 153.524548 | 234.660189 | 279.100281 | 283.382651 | -0.71851927 | 7.63E-05 | 0.00017646 |
| gene-BPHYT_RS33875 | 857.962588 | 884.377822 | 698.485856 | 1214.71157 | 1167.48644 | 1119.04459 | -0.52001836 | 7.64E-05 | 0.00017668 |
| gene-BPHYT_RS09535 | 596.98234 | 601.675623 | 715.770077 | 398.330742 | 477.211642 | 403.797644 | 0.58337553 | 7.68E-05 | 0.00017745 |
| gene-BPHYT_RS07690 | 444.544336 | 460.857924 | 436.172391 | 291.846286 | 287.822165 | 322.313814 | 0.57083782 | 7.83E-05 | 0.00018079 |
| gene-BPHYT_RS05205 | 249.806782 | 178.155725 | 201.310334 | 137.049438 | 79.7429375 | 110.455858 | 0.93929161 | 7.85E-05 | 0.00018122 |
| gene-BPHYT_RS34355 | 136.475909 | 123.748887 | 119.972825 | 74.9335059 | 62.2991699 | 57.9440565 | 0.96357796 | 7.96E-05 | 0.00018379 |
| gene-BPHYT_RS24725 | 35.1166083 | 58.6740413 | 43.718911 | 5.91580309 | 0 | 18.1075177 | 2.45337965 | 8.02E-05 | 0.00018492 |
| gene-BPHYT_RS08300 | 730.265831 | 681.685679 | 597.830689 | 437.769429 | 413.666488 | 498.862112 | 0.57266872 | 8.06E-05 | 0.00018573 |
| gene-BPHYT_RS34615 | 42.2995509 | 74.6760525 | 35.5851601 | 18.7333765 | 12.459834 | 12.6752624 | 1.78882289 | 8.20E-05 | 0.00018888 |
| gene-BPHYT_RS13460 | 31.1260846 | 18.1356128 | 43.718911 | 76.9054402 | 80.9889209 | 80.5784536 | -1.3551568 | 8.23E-05 | 0.00018954 |
| gene-BPHYT_RS28890 | 280.134761 | 234.696165 | 294.848469 | 168.600388 | 138.304157 | 176.548297 | 0.740255 | 8.33E-05 | 0.00019174 |
| gene-BPHYT_RS17255 | 1399.0776 | 1479.65264 | 1588.11486 | 2010.38709 | 1982.35959 | 1998.16457 | -0.42407299 | 8.39E-05 | 0.00019309 |
| gene-BPHYT_RS07115 | 2418.25734 | 2925.16766 | 2952.55157 | 3762.45077 | 3913.63385 | 3693.02823 | -0.45504257 | 8.56E-05 | 0.00019704 |
| gene-BPHYT_RS11480 | 3041.57714 | 3652.72577 | 4206.16592 | 5565.78474 | 4993.90146 | 5065.57807 | -0.51992828 | 8.63E-05 | 0.00019853 |
| gene-BPHYT_RS16910 | 1558.69854 | 1367.63856 | 1014.68542 | 879.482727 | 778.739624 | 890.889869 | 0.62858629 | 8.82E-05 | 0.00020276 |
| gene-BPHYT_RS16820 | 348.771768 | 361.645454 | 309.082533 | 201.137305 | 221.785045 | 230.87085 | 0.64110532 | 8.83E-05 | 0.00020287 |
| gene-BPHYT_RS12360 | 334.405883 | 283.769 | 269.430498 | 519.604705 | 393.730754 | 483.470722 | -0.65394792 | 8.84E-05 | 0.00020314 |
| gene-BPHYT_RS13270 | 131.687281 | 140.817699 | 105.738761 | 59.1580309 | 76.0049873 | 53.4171771 | 1.0139958 | 8.92E-05 | 0.00020485 |
| gene-BPHYT_RS19570 | 972.88967 | 1031.59633 | 959.782603 | 1349.78907 | 1333.20224 | 1310.98428 | -0.4303898 | 8.98E-05 | 0.00020608 |
| gene-BPHYT_RS23900 | 90.9839396 | 61.8744435 | 77.2706333 | 164.656519 | 128.33629 | 151.197773 | -0.94454821 | 9.17E-05 | 0.00021043 |
| gene-BPHYT_RS17530 | 909.041291 | 983.590292 | 818.458682 | 642.850603 | 629.221616 | 648.249132 | 0.49704321 | 9.27E-05 | 0.00021246 |
| gene-BPHYT_RS15935 | 343.98314 | 260.299383 | 333.483786 | 518.618738 | 426.126322 | 526.928764 | -0.64986239 | 9.35E-05 | 0.00021433 |
| gene-BPHYT_RS19535 | 3374.38681 | 3644.19136 | 3264.68426 | 4357.97495 | 4605.15464 | 4562.18907 | -0.39542228 | 9.47E-05 | 0.00021682 |
| gene-BPHYT_RS13045 | 181.169774 | 197.358139 | 194.193302 | 102.540587 | 129.582273 | 95.9698436 | 0.81011826 | 9.47E-05 | 0.00021682 |
| gene-BPHYT_RS14870 | 854.770169 | 1037.99713 | 1022.81917 | 1429.65241 | 1265.91913 | 1417.81863 | -0.49847072 | 9.50E-05 | 0.00021755 |
| gene-BPHYT_RS03000 | 54.2711219 | 33.0708233 | 44.7356298 | 128.175734 | 78.4969541 | 97.7805954 | -1.19953466 | 9.68E-05 | 0.00022154 |
| gene-BPHYT_RS03195 | 2186.00886 | 2575.25701 | 2917.98313 | 3877.80893 | 3665.68316 | 3364.37678 | -0.50673931 | 9.90E-05 | 0.00022642 |
| gene-BPHYT_RS08275 | 331.213464 | 375.513864 | 339.584099 | 235.646157 | 209.325211 | 233.586978 | 0.62121876 | 9.92E-05 | 0.00022671 |
| gene-BPHYT_RS14065 | 391.869424 | 422.453097 | 397.537074 | 270.155008 | 250.442663 | 286.098779 | 0.58405893 | 9.94E-05 | 0.00022709 |
| gene-BPHYT_RS06485 | 293.702542 | 347.777045 | 358.901757 | 235.646157 | 119.614406 | 189.22356 | 0.87004937 | 9.94E-05 | 0.00022709 |
| gene-BPHYT_RS18745 | 921.810967 | 428.853902 | 413.804576 | 315.509498 | 313.987816 | 252.599871 | 1.00359163 | 9.95E-05 | 0.00022709 |
| gene-BPHYT_RS13190 | 396.658052 | 322.173827 | 491.075209 | 284.944516 | 191.881443 | 221.817091 | 0.78978736 | 9.95E-05 | 0.00022709 |
| gene-BPHYT_RS18385 | 39.9052367 | 48.0060338 | 32.5350035 | 13.8035406 | 12.459834 | 7.24300706 | 1.85777799 | 0.00010043 | 0.0002291 |
| gene-BPHYT_RS04465 | 207.507231 | 248.564575 | 195.210021 | 342.130612 | 309.003883 | 389.31163 | -0.67995628 | 0.00010075 | 0.00022973 |
| gene-BPHYT_RS01865 | 865.943636 | 733.958916 | 985.200575 | 644.822537 | 513.34516 | 553.184665 | 0.59423621 | 0.00010177 | 0.00023197 |
| gene-BPHYT_RS15970 | 2836.46422 | 3038.24854 | 3047.10642 | 3746.67529 | 3879.9923 | 4095.9205 | -0.3942207 | 0.00010231 | 0.00023308 |
| gene-BPHYT_RS16790 | 111.734663 | 158.953312 | 130.140014 | 245.505828 | 203.095294 | 240.829985 | -0.79018828 | 0.00010427 | 0.00023746 |
| gene-BPHYT_RS02695 | 769.372963 | 1056.13274 | 1019.76902 | 637.920767 | 667.847102 | 595.737331 | 0.58077633 | 0.00010652 | 0.00024247 |
| gene-BPHYT_RS02860 | 1068.66224 | 1282.2945 | 1115.34059 | 734.545551 | 867.204445 | 835.66194 | 0.5079935 | 0.00010674 | 0.00024288 |
| gene-BPHYT_RS18420 | 60.6559597 | 81.076857 | 100.655167 | 20.7053108 | 18.689751 | 45.2687942 | 1.49507684 | 0.00010748 | 0.00024446 |
| gene-BPHYT_RS21835 | 43.8957603 | 34.137624 | 27.4514092 | 87.7510792 | 72.2670371 | 85.105333 | -1.20943787 | 0.00011016 | 0.00025044 |
| gene-BPHYT_RS19895 | 51.8768077 | 38.404827 | 35.5851601 | 15.7754749 | 12.459834 | 3.62150353 | 2.00624164 | 0.00011028 | 0.00025062 |
| gene-BPHYT_RS15685 | 742.237402 | 744.626924 | 815.408525 | 1085.54987 | 984.326885 | 1113.61234 | -0.46886588 | 0.00011247 | 0.00025547 |
| gene-BPHYT_RS29135 | 55.8673313 | 39.4716278 | 57.952975 | 6.90177028 | 23.6736846 | 15.39139 | 1.76451003 | 0.00011251 | 0.00025547 |
| gene-BPHYT_RS01665 | 2629.7551 | 2942.23647 | 2807.16077 | 4087.81994 | 3523.64105 | 3648.66481 | -0.42684703 | 0.00011275 | 0.00025591 |
| gene-BPHYT_RS15870 | 589.001293 | 653.94886 | 780.840084 | 997.798788 | 928.257632 | 1007.68336 | -0.53757418 | 0.00011306 | 0.00025644 |
| gene-BPHYT_RS33655 | 243.421944 | 220.827755 | 289.764875 | 454.530871 | 382.516903 | 359.434226 | -0.6663642 | 0.00011308 | 0.00025644 |
| gene-BPHYT_RS35585 | 423.793613 | 338.175838 | 226.728306 | 594.538211 | 560.692529 | 534.171771 | -0.76973395 | 0.00011367 | 0.00025768 |
| gene-BPHYT_RS02070 | 39.1071319 | 30.9372218 | 27.4514092 | 8.87370464 | 8.72188379 | 6.33763118 | 2.0407623 | 0.00011651 | 0.000264 |
| gene-BPHYT_RS38630 | 39.1071319 | 38.404827 | 31.5182846 | 5.91580309 | 11.2138506 | 11.7698865 | 1.92157773 | 0.000117 | 0.000265 |
| gene-BPHYT_RS01820 | 347.175559 | 336.042236 | 314.166128 | 450.587002 | 477.211642 | 581.251317 | -0.59812109 | 0.00011727 | 0.00026549 |
| gene-BPHYT_RS12275 | 1070.25845 | 822.503378 | 617.148348 | 530.450344 | 528.296961 | 480.754594 | 0.70715107 | 0.00011787 | 0.00026674 |
| gene-BPHYT_RS05940 | 629.704635 | 540.86798 | 565.295686 | 818.352761 | 801.167325 | 814.838295 | -0.48645434 | 0.00011799 | 0.00026691 |
| gene-BPHYT_RS30420 | 28.7317704 | 25.603218 | 63.0365693 | 81.8352761 | 114.630473 | 102.307475 | -1.35138032 | 0.00011916 | 0.00026944 |
| gene-BPHYT_RS19155 | 144.456957 | 112.014079 | 83.3709465 | 61.1299653 | 52.3313027 | 40.7419147 | 1.14747803 | 0.00011934 | 0.00026972 |
| gene-BPHYT_RS26045 | 8.77915207 | 24.5364173 | 13.2173452 | 0 | 0 | 0.90537588 | 5.37608716 | 0.0001212 | 0.00027381 |
| gene-BPHYT_RS08620 | 98.1668822 | 130.149692 | 98.6217294 | 195.221502 | 232.998896 | 163.873035 | -0.85621722 | 0.00012301 | 0.0002778 |
| gene-BPHYT_RS06120 | 808.480095 | 836.371788 | 857.093998 | 1194.99223 | 1049.11802 | 1181.51553 | -0.45469922 | 0.00012751 | 0.00028782 |
| gene-BPHYT_RS32750 | 59.0597503 | 70.4088495 | 76.2539145 | 121.273963 | 158.239892 | 118.604241 | -0.95226265 | 0.00012944 | 0.00029206 |
| gene-BPHYT_RS02965 | 524.35481 | 650.748458 | 563.262248 | 871.594989 | 818.611093 | 792.203898 | -0.51567192 | 0.00013012 | 0.00029348 |
| gene-BPHYT_RS09935 | 55.8673313 | 44.8056315 | 48.8025053 | 87.7510792 | 107.154572 | 113.171985 | -1.03777522 | 0.00013057 | 0.00029437 |
| gene-BPHYT_RS28350 | 11.971571 | 13.8684098 | 32.5350035 | 1.97193437 | 1.2459834 | 1.81075177 | 3.51035473 | 0.0001346 | 0.00030333 |
| gene-BPHYT_RS11330 | 1231.4756 | 1042.26433 | 882.51197 | 713.84024 | 654.141284 | 761.421118 | 0.56778144 | 0.00013545 | 0.00030512 |
| gene-BPHYT_RS13925 | 566.654361 | 474.726334 | 456.506768 | 327.341105 | 322.7097 | 354.00197 | 0.57787702 | 0.00013791 | 0.00031053 |
| gene-BPHYT_RS13355 | 106.14793 | 147.218504 | 169.79205 | 70.0036699 | 67.2831035 | 76.9569501 | 0.97465311 | 0.00013889 | 0.00031261 |
| gene-BPHYT_RS21685 | 142.860747 | 134.416895 | 127.089857 | 66.0598012 | 73.5130205 | 79.6730777 | 0.88351061 | 0.00013986 | 0.00031465 |
| gene-BPHYT_RS03580 | 1277.76568 | 1515.92387 | 1163.12638 | 1018.5041 | 874.680346 | 821.175926 | 0.54333158 | 0.00014022 | 0.00031534 |
| gene-BPHYT_RS21380 | 47.0881793 | 26.6700188 | 45.7523487 | 11.8316062 | 8.72188379 | 12.6752624 | 1.83810622 | 0.00014168 | 0.00031849 |
| gene-BPHYT_RS15910 | 809.2782 | 905.713837 | 1065.52137 | 677.359454 | 650.403334 | 507.010495 | 0.60026554 | 0.0001422 | 0.00031949 |
| gene-BPHYT_RS10910 | 36.7128177 | 39.4716278 | 53.8860995 | 9.85967182 | 19.9357344 | 8.14838295 | 1.80976847 | 0.00014225 | 0.00031949 |
| gene-BPHYT_RS32500 | 31.9241893 | 39.4716278 | 36.6018789 | 107.470423 | 64.7911367 | 76.9569501 | -1.21570294 | 0.00014592 | 0.00032749 |
| gene-BPHYT_RS22460 | 23.943142 | 21.336015 | 22.3678149 | 1.97193437 | 6.22991699 | 2.71612765 | 2.68797744 | 0.00014593 | 0.00032749 |
| gene-BPHYT_RS37155 | 201.122393 | 94.9452668 | 138.273765 | 382.555267 | 264.14848 | 230.87085 | -1.00846284 | 0.00014852 | 0.00033316 |
| gene-BPHYT_RS13040 | 52.6749124 | 51.206436 | 49.8192241 | 20.7053108 | 16.1977842 | 19.0128935 | 1.45251718 | 0.00015083 | 0.00033822 |
| gene-BPHYT_RS12485 | 5562.78999 | 6032.75824 | 5644.82311 | 7944.92356 | 7296.47878 | 7202.26515 | -0.38065674 | 0.00015135 | 0.00033923 |
| gene-BPHYT_RS14375 | 1446.96388 | 2230.68037 | 2302.86822 | 3048.61053 | 3141.12415 | 2940.66087 | -0.61116136 | 0.00015239 | 0.00034142 |
| gene-BPHYT_RS12430 | 3446.21624 | 4065.57766 | 5174.08228 | 6414.70249 | 6156.40397 | 5932.92816 | -0.54489669 | 0.00015347 | 0.0003437 |
| gene-BPHYT_RS11565 | 368.724387 | 300.837812 | 265.363622 | 447.629101 | 578.136297 | 459.025573 | -0.66223734 | 0.00015509 | 0.00034718 |
| gene-BPHYT_RS20630 | 272.951819 | 250.698176 | 256.213153 | 153.81088 | 183.15956 | 154.819276 | 0.67048094 | 0.00015589 | 0.00034882 |
| gene-BPHYT_RS09820 | 99.7630917 | 86.4108608 | 91.5046973 | 32.536917 | 58.5612197 | 34.4042836 | 1.16508399 | 0.00015824 | 0.00035394 |
| gene-BPHYT_RS10610 | 39.1071319 | 46.939233 | 55.9195373 | 98.5967182 | 102.170639 | 91.4429642 | -1.04638269 | 0.0001588 | 0.00035506 |
| gene-BPHYT_RS02520 | 987.255555 | 1134.0092 | 1060.43777 | 725.671846 | 763.787823 | 814.838295 | 0.46431642 | 0.00016142 | 0.00036075 |
| gene-BPHYT_RS04740 | 2456.56637 | 3472.43644 | 6102.34659 | 10775.6353 | 11397.0101 | 11242.0523 | -1.47376768 | 0.00016607 | 0.000371 |
| gene-BPHYT_RS24800 | 63.8483787 | 97.0788683 | 62.0198504 | 31.5509498 | 21.1817178 | 35.3096594 | 1.32263694 | 0.00016677 | 0.00037233 |
| gene-BPHYT_RS02385 | 1208.33057 | 1026.26232 | 944.53182 | 694.120896 | 738.868155 | 793.109274 | 0.51490556 | 0.00016681 | 0.00037233 |
| gene-BPHYT_RS28120 | 2636.93804 | 3830.88149 | 5878.66844 | 1754.03562 | 1761.82053 | 1257.5671 | 1.37105284 | 0.00016729 | 0.00037326 |
| gene-BPHYT_RS24655 | 477.26663 | 571.805202 | 454.47333 | 298.748056 | 320.217733 | 370.298736 | 0.60165253 | 0.0001677 | 0.00037401 |
| gene-BPHYT_RS34170 | 303.279799 | 237.896567 | 209.444085 | 149.867012 | 149.518008 | 148.481645 | 0.74862005 | 0.00016846 | 0.00037556 |
| gene-BPHYT_RS09200 | 577.029722 | 522.732368 | 584.613344 | 918.921414 | 751.327989 | 748.745855 | -0.52212346 | 0.00017256 | 0.00038453 |
| gene-BPHYT_RS32820 | 70.2332165 | 106.680075 | 79.304071 | 38.4527201 | 44.8554023 | 28.0666524 | 1.20895948 | 0.00017403 | 0.00038766 |
| gene-BPHYT_RS13080 | 141.264538 | 129.082891 | 114.889231 | 197.193437 | 254.180613 | 201.898822 | -0.75538323 | 0.00017888 | 0.00039829 |
| gene-BPHYT_RS28995 | 93.3782538 | 124.815688 | 110.822356 | 53.2422278 | 66.0371201 | 39.8365389 | 1.05670226 | 0.00018177 | 0.00040457 |
| gene-BPHYT_RS06180 | 1852.40109 | 2080.26146 | 1942.94974 | 2633.51834 | 2559.2499 | 2515.1342 | -0.39204419 | 0.00018814 | 0.00041857 |
| gene-BPHYT_RS06740 | 1575.45874 | 1734.61802 | 1534.22876 | 1189.07642 | 1197.39005 | 1241.27034 | 0.41671095 | 0.00018873 | 0.0004197 |
| gene-BPHYT_RS29885 | 50.2805982 | 58.6740413 | 66.0867259 | 28.5930483 | 6.22991699 | 19.9182694 | 1.647602 | 0.00019292 | 0.00042884 |
| gene-BPHYT_RS22370 | 181.967879 | 128.01609 | 116.922669 | 73.9475387 | 76.0049873 | 73.3354465 | 0.94116919 | 0.00019597 | 0.00043543 |
| gene-BPHYT_RS28845 | 49.4824935 | 42.67203 | 68.1201636 | 17.7474093 | 22.4277012 | 18.1075177 | 1.46716768 | 0.00020011 | 0.00044447 |
| gene-BPHYT_RS16240 | 656.840195 | 672.084473 | 697.469138 | 974.135576 | 865.958462 | 925.294152 | -0.44975238 | 0.00020033 | 0.00044474 |
| gene-BPHYT_RS23250 | 235.440896 | 241.09697 | 143.357359 | 365.793825 | 377.53297 | 315.070807 | -0.76889144 | 0.0002004 | 0.00044474 |
| gene-BPHYT_RS35525 | 27.1355609 | 64.008045 | 47.7857864 | 119.302029 | 90.9567881 | 104.118227 | -1.19503331 | 0.00020107 | 0.00044595 |
| gene-BPHYT_RS13935 | 160.419051 | 144.018101 | 188.092989 | 95.6388167 | 85.9728545 | 97.7805954 | 0.81476474 | 0.00020111 | 0.00044595 |
| gene-BPHYT_RS09460 | 64238.6519 | 64031.5146 | 55709.0765 | 78299.5978 | 84997.2495 | 77718.3712 | -0.38955984 | 0.000202 | 0.00044774 |
| gene-BPHYT_RS10425 | 472.478002 | 666.750469 | 590.713657 | 924.837217 | 784.969541 | 853.769458 | -0.57044374 | 0.00020451 | 0.0004531 |
| gene-BPHYT_RS28945 | 681.581442 | 787.298954 | 813.375087 | 541.295983 | 549.478679 | 535.077147 | 0.48829384 | 0.00020647 | 0.00045727 |
| gene-BPHYT_RS08410 | 63.8483787 | 61.8744435 | 62.0198504 | 30.5649827 | 23.6736846 | 23.539773 | 1.26930934 | 0.00020668 | 0.00045756 |
| gene-BPHYT_RS33595 | 1814.89016 | 1910.64014 | 1553.54642 | 2313.07901 | 2287.62552 | 2717.9384 | -0.47165996 | 0.00020986 | 0.00046428 |
| gene-BPHYT_RS29130 | 62.2521692 | 84.2772593 | 80.3207899 | 35.4948186 | 29.9036016 | 34.4042836 | 1.17513026 | 0.0002099 | 0.00046428 |
| gene-BPHYT_RS01950 | 1245.84149 | 1298.29651 | 1477.2925 | 1834.88493 | 1760.57454 | 1807.13026 | -0.42663545 | 0.00021048 | 0.00046539 |
| gene-BPHYT_RS02515 | 1228.28318 | 1779.42365 | 1642.00096 | 1130.90436 | 930.749599 | 1065.62741 | 0.57047418 | 0.00021301 | 0.00047079 |
| gene-BPHYT_RS06035 | 231.450373 | 260.299383 | 196.22674 | 395.37284 | 320.217733 | 356.718098 | -0.64239033 | 0.00021591 | 0.00047699 |
| gene-BPHYT_RS32490 | 61.4540645 | 75.7428533 | 96.5882916 | 139.021373 | 152.009975 | 142.144014 | -0.89495699 | 0.00021632 | 0.00047772 |
| gene-BPHYT_RS23990 | 454.919698 | 429.920702 | 394.486917 | 228.744386 | 306.511916 | 300.584793 | 0.61649119 | 0.00021755 | 0.00048022 |
| gene-BPHYT_RS29175 | 700.735956 | 650.748458 | 718.820234 | 490.02569 | 502.13131 | 504.294367 | 0.46874413 | 0.00022125 | 0.00048821 |
| gene-BPHYT_RS26635 | 106.946034 | 117.348083 | 116.922669 | 172.544257 | 183.15956 | 215.47946 | -0.74715527 | 0.00022649 | 0.00049956 |
| gene-BPHYT_RS04370 | 85.3972065 | 128.01609 | 119.972825 | 208.039076 | 198.11136 | 175.642921 | -0.80941374 | 0.00022698 | 0.00050043 |
| gene-BPHYT_RS16070 | 290.510123 | 230.428962 | 228.761743 | 183.389896 | 102.170639 | 131.279503 | 0.84351479 | 0.00022771 | 0.00050183 |
| gene-BPHYT_RS12065 | 312.857055 | 279.501797 | 396.520355 | 548.197753 | 482.195575 | 479.849218 | -0.61153129 | 0.00023674 | 0.00052153 |
| gene-BPHYT_RS14125 | 1008.80438 | 1028.39592 | 951.648852 | 1354.71891 | 1380.54961 | 1255.75635 | -0.41654452 | 0.00023943 | 0.00052723 |
| gene-BPHYT_RS12795 | 45.4919698 | 73.6092518 | 65.070007 | 32.536917 | 18.689751 | 12.6752624 | 1.52289856 | 0.00024094 | 0.00053034 |
| gene-BPHYT_RS33890 | 471.679897 | 496.062349 | 585.630063 | 767.082468 | 689.028819 | 752.367359 | -0.50968331 | 0.00024314 | 0.00053498 |
| gene-BPHYT_RS30225 | 344.781245 | 413.918691 | 373.135821 | 514.674869 | 575.64433 | 536.887899 | -0.52474664 | 0.0002459 | 0.00054083 |
| gene-BPHYT_RS14705 | 3521.23808 | 3466.03564 | 2990.17017 | 4234.72905 | 4313.59453 | 4746.88575 | -0.41414518 | 0.00025044 | 0.00055058 |
| gene-BPHYT_RS21855 | 113.330872 | 78.9432555 | 92.5214162 | 49.2983591 | 51.0853193 | 34.4042836 | 1.09198484 | 0.00025208 | 0.00055395 |
| gene-BPHYT_RS21260 | 85.3972065 | 81.076857 | 83.3709465 | 46.3404576 | 41.1174522 | 28.0666524 | 1.12016335 | 0.00026141 | 0.00057422 |
| gene-BPHYT_RS07845 | 190.747031 | 256.03218 | 201.310334 | 151.838946 | 112.138506 | 71.5246948 | 0.95077245 | 0.00026472 | 0.00058127 |
| gene-BPHYT_RS14370 | 1525.17815 | 1752.75363 | 1896.18067 | 1216.6835 | 1309.52855 | 1271.14774 | 0.44592492 | 0.00026873 | 0.00058983 |
| gene-BPHYT_RS23860 | 758.199497 | 764.896138 | 733.054298 | 963.289937 | 989.310818 | 1110.89621 | -0.44194111 | 0.00027202 | 0.00059681 |
| gene-BPHYT_RS24975 | 51.0787029 | 68.275248 | 69.1368824 | 24.6491796 | 31.149585 | 21.7290212 | 1.28892061 | 0.00027298 | 0.00059868 |
| gene-BPHYT_RS08575 | 1215.51351 | 1241.75607 | 1196.6781 | 905.117873 | 912.059848 | 947.92855 | 0.40176379 | 0.00027315 | 0.0005988 |
| gene-BPHYT_RS14440 | 5399.97663 | 6639.76787 | 7042.81154 | 9555.99393 | 8640.89487 | 8109.45178 | -0.46337297 | 0.00027747 | 0.00060803 |
| gene-BPHYT_RS09790 | 146.053166 | 106.680075 | 142.34064 | 58.1720638 | 72.2670371 | 78.7677018 | 0.91980314 | 0.00027825 | 0.00060948 |
| gene-BPHYT_RS09485 | 661.628824 | 649.681657 | 447.356298 | 391.428971 | 392.484771 | 356.718098 | 0.62632253 | 0.00027932 | 0.00061158 |
| gene-BPHYT_RS20335 | 17.5583041 | 7.46760525 | 6.10031316 | 40.4246545 | 31.149585 | 43.4580424 | -1.85576942 | 0.00028892 | 0.00063235 |
| gene-BPHYT_RS07330 | 90.1858349 | 93.878466 | 94.5548539 | 55.2141622 | 32.3955684 | 45.2687942 | 1.05668114 | 0.00028949 | 0.00063333 |
| gene-BPHYT_RS19660 | 526.749124 | 584.606811 | 454.47333 | 413.120249 | 297.790032 | 291.531034 | 0.64218498 | 0.00029727 | 0.00065008 |
| gene-BPHYT_RS10480 | 13.5677805 | 13.8684098 | 38.6353167 | 0 | 1.2459834 | 5.4322553 | 3.24206629 | 0.00030845 | 0.00067426 |
| gene-BPHYT_RS18735 | 616.934959 | 304.038214 | 531.743963 | 258.323402 | 325.201667 | 229.965474 | 0.84140511 | 0.0003128 | 0.00068349 |
| gene-BPHYT_RS30470 | 45.4919698 | 20.2692143 | 30.5015658 | 74.9335059 | 63.5451533 | 98.6859712 | -1.28951835 | 0.00031324 | 0.00068418 |
| gene-BPHYT_RS32510 | 47.0881793 | 23.4696165 | 37.6185978 | 102.540587 | 80.9889209 | 68.8085671 | -1.20873751 | 0.00031478 | 0.00068726 |
| gene-BPHYT_RS22355 | 795.710419 | 689.153285 | 677.13476 | 461.432641 | 544.494745 | 525.118012 | 0.50005108 | 0.00032309 | 0.00070512 |
| gene-BPHYT_RS04505 | 134.8797 | 80.0100563 | 113.872512 | 49.2983591 | 46.1013857 | 63.3763118 | 1.04846528 | 0.00032701 | 0.00071314 |
| gene-BPHYT_RS27005 | 731.86204 | 457.657522 | 300.948782 | 1520.3614 | 1102.69531 | 1295.59289 | -1.39377309 | 0.00032703 | 0.00071314 |
| gene-BPHYT_RS03715 | 697.543537 | 742.493322 | 769.656177 | 1023.43394 | 999.278686 | 947.023174 | -0.42696905 | 0.00032739 | 0.00071365 |
| gene-BPHYT_RS21555 | 252.201096 | 332.841834 | 238.928932 | 390.443004 | 447.30804 | 422.810537 | -0.61482466 | 0.00032882 | 0.00071647 |
| gene-BPHYT_RS01860 | 86.9934159 | 121.615286 | 177.9258 | 40.4246545 | 67.2831035 | 67.9031912 | 1.13692509 | 0.00033191 | 0.00072292 |
| gene-BPHYT_RS21735 | 470.083688 | 392.582676 | 362.968633 | 575.804834 | 585.612197 | 607.507218 | -0.52694965 | 0.00033582 | 0.00073114 |
| gene-BPHYT_RS14320 | 2580.2726 | 2708.60711 | 2543.83059 | 1990.66774 | 1900.12468 | 2132.1602 | 0.3783814 | 0.00033993 | 0.00073979 |
| gene-BPHYT_RS03400 | 1018.38164 | 1093.47077 | 1058.40433 | 1330.06973 | 1415.43714 | 1424.15626 | -0.39572299 | 0.0003437 | 0.00074769 |
| gene-BPHYT_RS08605 | 98.9649869 | 156.81971 | 83.3709465 | 60.1439981 | 29.9036016 | 57.0386806 | 1.18998098 | 0.00034465 | 0.00074944 |
| gene-BPHYT_RS00160 | 411.023938 | 427.787101 | 322.299878 | 512.702935 | 608.039898 | 598.453459 | -0.56507339 | 0.00034508 | 0.00075008 |
| Novel00758 | 32.7222941 | 39.4716278 | 47.7857864 | 68.0317356 | 87.2188379 | 102.307475 | -1.10968281 | 0.0003453 | 0.00075026 |
| gene-BPHYT_RS30445 | 57.4635408 | 38.404827 | 41.6854732 | 100.568653 | 88.4648213 | 92.3483401 | -1.02319797 | 0.00034826 | 0.00075638 |
| gene-BPHYT_RS26315 | 478.86284 | 521.665567 | 430.072078 | 329.313039 | 320.217733 | 343.13746 | 0.52598156 | 0.00035294 | 0.00076623 |
| gene-BPHYT_RS01970 | 62.2521692 | 32.0040225 | 65.070007 | 123.245898 | 92.2027715 | 122.225744 | -1.07994193 | 0.00035403 | 0.00076814 |
| gene-BPHYT_RS09340 | 243.421944 | 247.497774 | 191.143146 | 412.134282 | 388.74682 | 290.625658 | -0.67563565 | 0.0003541 | 0.00076814 |
| gene-BPHYT_RS29645 | 78.2142639 | 85.34406 | 74.2204767 | 32.536917 | 41.1174522 | 39.8365389 | 1.0692985 | 0.00035697 | 0.00077406 |
| gene-BPHYT_RS16060 | 33.5203988 | 37.3380263 | 47.7857864 | 18.7333765 | 3.7379502 | 9.05375883 | 1.88233874 | 0.00035932 | 0.00077883 |
| gene-BPHYT_RS14675 | 16.7601994 | 14.9352105 | 22.3678149 | 0 | 0 | 4.52687942 | 3.45616693 | 0.0003606 | 0.0007813 |
| gene-BPHYT_RS33390 | 16.7601994 | 14.9352105 | 12.2006263 | 0.98596718 | 0 | 0.90537588 | 4.43869744 | 0.00036308 | 0.00078635 |
| gene-BPHYT_RS26140 | 55.8673313 | 77.8764548 | 51.8526618 | 25.6351467 | 24.919668 | 26.2559006 | 1.26781124 | 0.0003635 | 0.00078696 |
| gene-BPHYT_RS22735 | 479.660945 | 449.123116 | 606.981159 | 341.144645 | 356.351252 | 339.515956 | 0.56679591 | 0.00036508 | 0.00079006 |
| gene-BPHYT_RS17010 | 1928.22104 | 2444.04052 | 2694.30498 | 3300.03216 | 3372.87706 | 3170.62634 | -0.47870667 | 0.00036636 | 0.00079251 |
| gene-BPHYT_RS15880 | 888.290568 | 997.458702 | 996.384482 | 1213.7256 | 1305.7906 | 1330.90255 | -0.41874797 | 0.00036654 | 0.00079258 |
| gene-BPHYT_RS13430 | 186.756508 | 137.617297 | 79.304071 | 0 | 14.9518008 | 9.05375883 | 4.08479666 | 0.00037158 | 0.00080316 |
| gene-BPHYT_RS07175 | 398.254262 | 388.315473 | 341.617537 | 266.211139 | 259.164547 | 244.451488 | 0.55295535 | 0.00037262 | 0.00080507 |
| gene-BPHYT_RS16425 | 83.800997 | 60.8076428 | 72.187039 | 40.4246545 | 23.6736846 | 29.8774041 | 1.20062401 | 0.00037556 | 0.00081101 |
| gene-BPHYT_RS23730 | 118.917605 | 109.880477 | 137.257046 | 82.8212433 | 53.5772861 | 50.7010495 | 0.96561029 | 0.00037567 | 0.00081101 |
| gene-BPHYT_RS26810 | 98.9649869 | 70.4088495 | 76.2539145 | 157.754749 | 156.993908 | 128.563375 | -0.84229058 | 0.00038883 | 0.00083909 |
| gene-BPHYT_RS15260 | 862.751217 | 613.410431 | 706.619607 | 439.741363 | 474.719675 | 550.468537 | 0.57617119 | 0.00038916 | 0.00083947 |
| gene-BPHYT_RS06275 | 215.488278 | 275.234594 | 299.932064 | 412.134282 | 403.698621 | 379.352495 | -0.59938407 | 0.0003922 | 0.00084569 |
| gene-BPHYT_RS27085 | 93.3782538 | 82.1436578 | 120.989544 | 58.1720638 | 43.6094189 | 43.4580424 | 1.02691723 | 0.0003976 | 0.00085698 |
| gene-BPHYT_RS12320 | 196.333764 | 187.756932 | 250.112839 | 123.245898 | 139.550141 | 123.13112 | 0.71873088 | 0.00040142 | 0.00086488 |
| gene-BPHYT_RS23120 | 223.469325 | 222.961357 | 238.928932 | 147.895077 | 107.154572 | 160.251531 | 0.7146488 | 0.00040481 | 0.00087184 |
| gene-BPHYT_RS17175 | 83.800997 | 144.018101 | 109.805637 | 216.91278 | 170.699726 | 207.331077 | -0.82721701 | 0.0004142 | 0.00089161 |
| gene-BPHYT_RS06450 | 189.948927 | 86.4108608 | 122.006263 | 245.505828 | 223.031028 | 277.950396 | -0.89917989 | 0.00041432 | 0.00089161 |
| gene-BPHYT_RS05990 | 23.943142 | 27.7368195 | 28.4681281 | 82.8212433 | 41.1174522 | 72.4300706 | -1.30550964 | 0.00041917 | 0.00090168 |
| gene-BPHYT_RS23925 | 192.343241 | 195.224537 | 189.109708 | 71.9756043 | 115.876456 | 133.995631 | 0.84127092 | 0.00042713 | 0.00091835 |
| gene-BPHYT_RS03460 | 1055.09446 | 1122.27439 | 1124.49106 | 1418.80678 | 1530.06761 | 1388.8466 | -0.39346651 | 0.00042726 | 0.00091835 |
| gene-BPHYT_RS21885 | 188.352717 | 211.226549 | 168.775331 | 127.189767 | 80.9889209 | 117.698865 | 0.79393047 | 0.00042789 | 0.00091934 |
| gene-BPHYT_RS04430 | 217.084487 | 232.562564 | 315.182846 | 487.067788 | 362.581169 | 372.109488 | -0.67867419 | 0.00043188 | 0.00092754 |
| gene-BPHYT_RS07235 | 22.3469325 | 32.0040225 | 35.5851601 | 78.8773746 | 82.2349043 | 50.7010495 | -1.2403309 | 0.00043685 | 0.00093785 |
| gene-BPHYT_RS30720 | 39.1071319 | 28.8036203 | 34.5684412 | 8.87370464 | 7.47590039 | 13.5806382 | 1.75952148 | 0.00044737 | 0.00096004 |
| gene-BPHYT_RS18275 | 417.408776 | 575.005604 | 531.743963 | 341.144645 | 331.431584 | 354.907346 | 0.56568003 | 0.00044946 | 0.00096411 |
| gene-BPHYT_RS03405 | 1327.24817 | 1404.97659 | 1473.22563 | 1010.61636 | 986.818852 | 1145.30049 | 0.41884612 | 0.00044962 | 0.00096411 |
| gene-BPHYT_RS26905 | 42.2995509 | 32.0040225 | 20.3343772 | 5.91580309 | 7.47590039 | 10.8645106 | 1.96009422 | 0.00045031 | 0.00096521 |
| gene-BPHYT_RS06505 | 3676.0704 | 4134.91971 | 3898.10011 | 5040.26424 | 4982.68761 | 4907.13729 | -0.35084256 | 0.00045088 | 0.00096605 |
| gene-BPHYT_RS32815 | 114.927082 | 99.2124698 | 94.5548539 | 46.3404576 | 59.8072031 | 56.1333047 | 0.93413845 | 0.00045573 | 0.00097605 |
| gene-BPHYT_RS03885 | 569.84678 | 601.675623 | 536.827558 | 726.657813 | 765.033807 | 863.728592 | -0.46427809 | 0.00046141 | 0.00098783 |
| gene-BPHYT_RS24275 | 70.2332165 | 88.5444623 | 37.6185978 | 23.6632124 | 26.1656514 | 26.2559006 | 1.36875576 | 0.00046511 | 0.00099536 |
| gene-BPHYT_RS33685 | 40.7033414 | 59.740842 | 47.7857864 | 109.442357 | 107.154572 | 79.6730777 | -1.00336322 | 0.00047722 | 0.00102086 |
| gene-BPHYT_RS26750 | 252.201096 | 266.700188 | 298.915345 | 360.863989 | 394.976737 | 452.687942 | -0.56580692 | 0.00048462 | 0.00103627 |
| gene-BPHYT_RS18335 | 716.69805 | 623.011638 | 513.443024 | 796.661483 | 1016.72245 | 900.849004 | -0.54807749 | 0.00048548 | 0.0010377 |
| gene-BPHYT_RS02985 | 3.19241893 | 4.267203 | 12.2006263 | 20.7053108 | 23.6736846 | 45.2687942 | -2.21731374 | 0.00048592 | 0.00103822 |
| gene-BPHYT_RS13885 | 494.02683 | 336.042236 | 388.386604 | 641.864636 | 571.90638 | 602.074962 | -0.57278941 | 0.0004864 | 0.00103886 |
| gene-BPHYT_RS01475 | 687.168175 | 777.697747 | 901.829628 | 1179.21675 | 1110.17121 | 1016.73712 | -0.48321459 | 0.00049985 | 0.00106716 |
| gene-BPHYT_RS01660 | 1334.43111 | 1474.31864 | 1566.76376 | 1929.53778 | 1905.10862 | 1892.2356 | -0.38899251 | 0.00051021 | 0.00108884 |
| gene-BPHYT_RS14365 | 2287.36817 | 2477.11134 | 2781.7428 | 1924.60794 | 1964.91582 | 1815.27865 | 0.40351474 | 0.00051515 | 0.00109894 |
| gene-BPHYT_RS04685 | 221.873116 | 237.896567 | 178.942519 | 146.90911 | 112.138506 | 128.563375 | 0.71710693 | 0.00051967 | 0.00110815 |
| gene-BPHYT_RS01785 | 821.249771 | 812.902172 | 951.648852 | 632.004964 | 635.451533 | 637.384622 | 0.44055798 | 0.00052041 | 0.00110925 |
| gene-BPHYT_RS02130 | 3901.93404 | 4236.26578 | 3483.27881 | 2770.56778 | 2930.55295 | 3106.34466 | 0.39986652 | 0.0005206 | 0.00110925 |
| gene-BPHYT_RS30985 | 0 | 2.1336015 | 0 | 15.7754749 | 11.2138506 | 13.5806382 | -4.38838259 | 0.0005249 | 0.00111798 |
| gene-BPHYT_RS30370 | 28.7317704 | 55.473639 | 43.718911 | 4.92983591 | 4.98393359 | 21.7290212 | 1.97433981 | 0.00052533 | 0.00111845 |
| gene-BPHYT_RS04730 | 714.303736 | 775.564145 | 1766.04066 | 3007.19991 | 2824.64436 | 3243.05641 | -1.47905391 | 0.00053259 | 0.00113346 |
| gene-BPHYT_RS30755 | 13.5677805 | 12.801609 | 32.5350035 | 0 | 3.7379502 | 3.62150353 | 3.01963626 | 0.00053311 | 0.00113412 |
| gene-BPHYT_RS09170 | 4046.391 | 4770.73296 | 5413.01121 | 3740.75949 | 3472.55573 | 3182.39623 | 0.4527733 | 0.00054511 | 0.00115918 |
| gene-BPHYT_RS29670 | 137.274014 | 153.619308 | 117.939388 | 197.193437 | 266.640447 | 207.331077 | -0.71162183 | 0.00054588 | 0.00116036 |
| gene-BPHYT_RS32635 | 231.450373 | 249.631376 | 303.998939 | 393.400906 | 378.778953 | 384.78475 | -0.56194612 | 0.00054639 | 0.00116099 |
| gene-BPHYT_RS03865 | 182.765984 | 172.821722 | 191.143146 | 323.397236 | 241.720779 | 276.139644 | -0.6239112 | 0.00056577 | 0.00120171 |
| gene-BPHYT_RS32745 | 67.8389023 | 40.5384285 | 56.9362561 | 100.568653 | 154.501941 | 89.6322124 | -1.04500669 | 0.0005666 | 0.001203 |
| gene-BPHYT_RS29490 | 429.380347 | 389.382274 | 360.935195 | 512.702935 | 551.970646 | 605.696466 | -0.50064832 | 0.000567 | 0.00120336 |
| gene-BPHYT_RS12145 | 5530.0677 | 6305.85924 | 5868.50126 | 4720.81087 | 4686.14356 | 4460.78698 | 0.35227829 | 0.00056786 | 0.00120473 |
| gene-BPHYT_RS28100 | 143.658852 | 140.817699 | 113.872512 | 212.968911 | 232.998896 | 193.750439 | -0.67885798 | 0.00056927 | 0.00120724 |
| gene-BPHYT_RS21405 | 2241.8762 | 2052.52464 | 1741.63941 | 2558.58484 | 2798.47871 | 2758.68032 | -0.42648396 | 0.00057203 | 0.00121261 |
| gene-BPHYT_RS24170 | 100.561196 | 115.214481 | 116.922669 | 63.1018997 | 39.8714688 | 67.9031912 | 0.94644506 | 0.00057271 | 0.00121358 |
| gene-BPHYT_RS02885 | 5540.44306 | 6270.65481 | 5390.64339 | 4575.87369 | 4368.4178 | 4430.0042 | 0.36290162 | 0.00057474 | 0.0012174 |
| gene-BPHYT_RS33340 | 255.393515 | 182.422928 | 102.688605 | 95.6388167 | 82.2349043 | 90.5375883 | 1.01220913 | 0.00058285 | 0.0012341 |
| gene-BPHYT_RS13765 | 389.47511 | 501.396353 | 854.043842 | 307.621761 | 338.907484 | 351.285843 | 0.80491788 | 0.00058373 | 0.00123548 |
| gene-BPHYT_RS20595 | 22.3469325 | 18.1356128 | 11.1839075 | 67.0457684 | 39.8714688 | 39.8365389 | -1.49697912 | 0.00058641 | 0.00124066 |
| gene-BPHYT_RS10220 | 19.1545136 | 24.5364173 | 19.3176583 | 6.90177028 | 0 | 2.71612765 | 2.65260647 | 0.00058723 | 0.00124189 |
| gene-BPHYT_RS15160 | 83.800997 | 117.348083 | 113.872512 | 161.698618 | 186.89751 | 179.264425 | -0.75006325 | 0.00058745 | 0.00124189 |
| gene-BPHYT_RS23215 | 141.264538 | 124.815688 | 86.421103 | 65.073834 | 52.3313027 | 65.1870636 | 0.94695667 | 0.00058929 | 0.00124528 |
| gene-BPHYT_RS13180 | 223.469325 | 126.949289 | 105.738761 | 302.691925 | 246.704713 | 284.288027 | -0.86364457 | 0.0005929 | 0.00125242 |
| gene-BPHYT_RS36775 | 537.92259 | 601.675623 | 463.6238 | 390.443004 | 325.201667 | 385.690126 | 0.53909615 | 0.00059406 | 0.00125439 |
| gene-BPHYT_RS19695 | 58.2616455 | 51.206436 | 57.952975 | 28.5930483 | 21.1817178 | 20.8236453 | 1.24449873 | 0.00059613 | 0.00125825 |
| gene-BPHYT_RS34860 | 133.28349 | 104.546474 | 87.4378219 | 56.2001294 | 64.7911367 | 47.9849218 | 0.95644103 | 0.0006012 | 0.00126847 |
| gene-BPHYT_RS05530 | 419.004985 | 363.779056 | 323.316597 | 550.169688 | 510.853193 | 520.591133 | -0.51386442 | 0.00060249 | 0.00127069 |
| gene-BPHYT_RS03165 | 2667.26602 | 2444.04052 | 2246.94868 | 3160.02482 | 3485.01557 | 3041.15759 | -0.39577925 | 0.00060596 | 0.00127752 |
| gene-BPHYT_RS35675 | 438.957603 | 446.989514 | 418.88817 | 326.355137 | 287.822165 | 309.638552 | 0.49656187 | 0.00060884 | 0.00128309 |
| gene-BPHYT_RS02155 | 114.128977 | 142.951301 | 119.972825 | 178.46006 | 194.37341 | 248.978368 | -0.72722009 | 0.00061176 | 0.00128872 |
| gene-BPHYT_RS10775 | 0.79810473 | 1.06680075 | 0 | 9.85967182 | 12.459834 | 16.2967659 | -4.3597876 | 0.00061363 | 0.00129217 |
| gene-BPHYT_RS30150 | 75.0218449 | 115.214481 | 64.0532881 | 39.4386873 | 36.1335186 | 41.6472906 | 1.11008429 | 0.00061866 | 0.00130224 |
| gene-BPHYT_RS30465 | 1174.01206 | 1171.34722 | 776.773209 | 637.920767 | 666.601118 | 776.812508 | 0.5848653 | 0.00062041 | 0.00130542 |
| gene-BPHYT_RS02235 | 793.316105 | 696.62089 | 826.592433 | 1017.51813 | 1019.21442 | 1064.72204 | -0.4206286 | 0.00062907 | 0.00132313 |
| gene-BPHYT_RS20455 | 22.3469325 | 13.8684098 | 12.2006263 | 51.2702935 | 37.379502 | 46.17417 | -1.46331426 | 0.00063379 | 0.00133255 |
| gene-BPHYT_RS33590 | 4166.10671 | 4209.59576 | 4611.83675 | 6020.31562 | 5469.86712 | 5243.93711 | -0.3657588 | 0.00063767 | 0.00134018 |
| gene-BPHYT_RS33430 | 727.073412 | 710.4893 | 649.683351 | 495.941493 | 524.559011 | 521.496509 | 0.43764499 | 0.0006386 | 0.00134162 |
| gene-BPHYT_RS24785 | 4.7886284 | 8.534406 | 10.1671886 | 0 | 0 | 0 | 5.35382585 | 0.00064228 | 0.00134881 |
| gene-BPHYT_RS26730 | 549.894161 | 498.19595 | 752.371956 | 390.443004 | 398.714688 | 411.946027 | 0.58342224 | 0.00064382 | 0.00135152 |
| gene-BPHYT_RS34375 | 954.533261 | 1034.79673 | 994.351044 | 1246.26252 | 1380.54961 | 1280.2015 | -0.38886109 | 0.00065013 | 0.00136424 |
| gene-BPHYT_RS36910 | 1153.26134 | 1721.81641 | 1870.7627 | 1073.71826 | 1090.23547 | 1037.56076 | 0.56710112 | 0.00065221 | 0.00136807 |
| gene-BPHYT_RS15855 | 312.857055 | 275.234594 | 261.296747 | 206.067141 | 174.437676 | 181.075177 | 0.59733375 | 0.00065822 | 0.00138013 |
| gene-BPHYT_RS29105 | 604.963388 | 612.343631 | 500.225679 | 782.857943 | 758.80389 | 818.459798 | -0.45853136 | 0.00065875 | 0.00138071 |
| gene-BPHYT_RS09300 | 674.3985 | 681.685679 | 680.184917 | 821.310663 | 1029.18229 | 922.578025 | -0.44439307 | 0.00066228 | 0.00138757 |
| gene-BPHYT_RS02245 | 487.641992 | 573.938804 | 604.947721 | 764.124566 | 786.215524 | 738.786721 | -0.45931497 | 0.0006712 | 0.00140572 |
| gene-BPHYT_RS23145 | 168.400099 | 157.886511 | 114.889231 | 102.540587 | 73.5130205 | 54.322553 | 0.94064952 | 0.00067315 | 0.00140926 |
| gene-BPHYT_RS13345 | 257.787829 | 331.775033 | 321.28316 | 202.123272 | 200.603327 | 200.08807 | 0.59258922 | 0.00067343 | 0.0014093 |
| gene-BPHYT_RS19760 | 112.532767 | 152.552507 | 170.808768 | 188.319732 | 262.902497 | 291.531034 | -0.7733869 | 0.00067667 | 0.00141552 |
| gene-BPHYT_RS06500 | 1084.62433 | 1414.5778 | 1270.89857 | 1587.40716 | 1783.00224 | 1756.42921 | -0.44438309 | 0.00068573 | 0.00143391 |
| gene-BPHYT_RS17050 | 1913.05705 | 1897.83854 | 2283.55056 | 2657.18156 | 2632.76292 | 2691.6825 | -0.3894926 | 0.00071979 | 0.00150456 |
| gene-BPHYT_RS05125 | 108.542244 | 126.949289 | 113.872512 | 45.3544904 | 59.8072031 | 78.7677018 | 0.91982145 | 0.00072049 | 0.00150543 |
| gene-BPHYT_RS32845 | 7.98104733 | 6.4008045 | 1.01671886 | 21.691278 | 26.1656514 | 25.3505247 | -2.22018904 | 0.0007263 | 0.00151653 |
| gene-BPHYT_RS38595 | 7.1829426 | 2.1336015 | 3.05015658 | 35.4948186 | 7.47590039 | 30.78278 | -2.54539764 | 0.00072636 | 0.00151653 |
| gene-BPHYT_RS03550 | 1267.39032 | 1505.25586 | 1376.63734 | 1078.6481 | 1020.4604 | 1042.99302 | 0.40005657 | 0.00073282 | 0.00152942 |
| gene-BPHYT_RS14610 | 907.445082 | 989.991096 | 771.689614 | 617.215456 | 614.269815 | 693.517926 | 0.47053753 | 0.00073461 | 0.00153258 |
| gene-BPHYT_RS03710 | 2100.61166 | 2521.91697 | 2649.56935 | 1838.8288 | 1885.17288 | 1733.79482 | 0.41379437 | 0.00074883 | 0.00156146 |
| gene-BPHYT_RS35805 | 43.0976556 | 42.67203 | 46.7690675 | 5.91580309 | 4.98393359 | 25.3505247 | 1.83251987 | 0.00074904 | 0.00156146 |
| gene-BPHYT_RS07250 | 19.1545136 | 14.9352105 | 11.1839075 | 40.4246545 | 36.1335186 | 48.8902977 | -1.46252768 | 0.00076229 | 0.00158848 |
| gene-BPHYT_RS10890 | 13.5677805 | 1.06680075 | 11.1839075 | 0 | 0 | 0 | 5.50642416 | 0.00077672 | 0.00161791 |
| gene-BPHYT_RS35500 | 34.3185035 | 54.4068383 | 69.1368824 | 103.526554 | 137.058174 | 86.0107089 | -1.05305415 | 0.0007785 | 0.00162101 |
| gene-BPHYT_RS09155 | 34329.677 | 35680.2179 | 35368.599 | 27406.9298 | 29695.5223 | 27758.8246 | 0.31242878 | 0.00079105 | 0.00164649 |
| gene-BPHYT_RS02740 | 1135.70304 | 1472.18504 | 1245.4806 | 1010.61636 | 892.124113 | 908.997387 | 0.45333142 | 0.00079224 | 0.00164833 |
| gene-BPHYT_RS26365 | 19.9526183 | 18.1356128 | 30.5015658 | 4.92983591 | 4.98393359 | 5.4322553 | 2.15544812 | 0.00079651 | 0.00165658 |
| gene-BPHYT_RS09305 | 166.803889 | 190.957334 | 196.22674 | 116.344128 | 130.828257 | 84.1999571 | 0.74727554 | 0.00080791 | 0.00167964 |
| gene-BPHYT_RS19610 | 458.910222 | 352.044248 | 590.713657 | 745.39119 | 675.323002 | 696.234054 | -0.59477649 | 0.00080936 | 0.001682 |
| gene-BPHYT_RS23835 | 2028.78223 | 1448.71542 | 1409.17234 | 1169.35708 | 1117.64711 | 1155.25963 | 0.50638473 | 0.00081039 | 0.00168351 |
| gene-BPHYT_RS15075 | 213.093964 | 226.161759 | 223.678149 | 319.453367 | 407.436571 | 283.382651 | -0.60445356 | 0.0008201 | 0.00170302 |
| gene-BPHYT_RS21560 | 407.033414 | 362.712255 | 345.684412 | 493.969558 | 589.350147 | 497.05136 | -0.49886744 | 0.00082293 | 0.00170825 |
| gene-BPHYT_RS17145 | 13.5677805 | 8.534406 | 7.11703202 | 25.6351467 | 33.6415518 | 38.0257871 | -1.71521155 | 0.00083082 | 0.00172395 |
| gene-BPHYT_RS10155 | 143.658852 | 150.418906 | 139.290484 | 98.5967182 | 68.5290869 | 86.0107089 | 0.76868421 | 0.00084183 | 0.00174613 |
| gene-BPHYT_RS26725 | 396.658052 | 361.645454 | 405.670825 | 226.772452 | 302.773966 | 267.991261 | 0.5490717 | 0.00084835 | 0.00175897 |
| gene-BPHYT_RS03155 | 1278.56378 | 1160.67922 | 1211.92888 | 1500.64205 | 1662.14185 | 1569.92178 | -0.37323798 | 0.00084916 | 0.00175998 |
| gene-BPHYT_RS35590 | 2262.62692 | 2871.82762 | 2739.04061 | 3387.78324 | 3588.43219 | 3414.17246 | -0.4006465 | 0.00085951 | 0.00178075 |
| gene-BPHYT_RS35815 | 6.38483787 | 9.60120675 | 6.10031316 | 0 | 0 | 0 | 5.26828499 | 0.00086084 | 0.00178281 |
| gene-BPHYT_RS11325 | 538.720695 | 563.270796 | 461.590362 | 341.144645 | 389.992804 | 380.257871 | 0.49358366 | 0.0008629 | 0.0017864 |
| gene-BPHYT_RS24690 | 645.666729 | 541.934781 | 700.519294 | 453.544904 | 411.174522 | 475.322339 | 0.49360621 | 0.00088983 | 0.00184144 |
| gene-BPHYT_RS13500 | 959.321889 | 963.321078 | 1072.6384 | 671.443651 | 786.215524 | 776.812508 | 0.4230463 | 0.00090832 | 0.00187899 |
| gene-BPHYT_RS28940 | 1525.17815 | 1602.33473 | 1951.08349 | 1221.61334 | 1240.99947 | 1315.51116 | 0.42606445 | 0.00092268 | 0.00190795 |
| gene-BPHYT_RS08590 | 726.275307 | 571.805202 | 553.095059 | 408.190414 | 421.142389 | 468.984707 | 0.51290556 | 0.00092401 | 0.00190998 |
| gene-BPHYT_RS29380 | 5357.67707 | 4376.01668 | 3531.0646 | 5687.05871 | 6150.17405 | 6931.55776 | -0.50048955 | 0.00092442 | 0.0019101 |
| gene-BPHYT_RS29390 | 86.9934159 | 90.6780638 | 93.5381351 | 134.091537 | 180.667593 | 141.238638 | -0.74590488 | 0.0009322 | 0.00192542 |
| gene-BPHYT_RS12370 | 731.063936 | 682.75248 | 820.492119 | 564.959195 | 560.692529 | 521.496509 | 0.44050013 | 0.00093683 | 0.00193425 |
| gene-BPHYT_RS19650 | 148.44748 | 162.153714 | 140.307203 | 225.786485 | 223.031028 | 231.776226 | -0.59539653 | 0.00094756 | 0.00195565 |
| gene-BPHYT_RS29650 | 617.733064 | 630.479243 | 737.121173 | 403.260578 | 498.393359 | 507.91587 | 0.49410148 | 0.00096074 | 0.00198209 |
| gene-BPHYT_RS03495 | 737.448774 | 715.823303 | 733.054298 | 962.30397 | 995.540735 | 904.470507 | -0.38765403 | 0.00096979 | 0.00200001 |
| gene-BPHYT_RS32690 | 177.977356 | 174.955323 | 213.51096 | 121.273963 | 124.59834 | 112.26661 | 0.66263474 | 0.00098087 | 0.00202208 |
| gene-BPHYT_RS35265 | 75.8199497 | 65.0748458 | 75.2371956 | 106.484456 | 135.81219 | 133.995631 | -0.79601864 | 0.00099275 | 0.0020458 |
| gene-BPHYT_RS34435 | 142.860747 | 138.684098 | 164.708455 | 271.140975 | 210.571194 | 213.668708 | -0.64189414 | 0.00099387 | 0.00204732 |
| gene-BPHYT_RS02350 | 881.90573 | 815.035773 | 886.578845 | 1115.12888 | 1091.48146 | 1147.11124 | -0.37633584 | 0.00099511 | 0.0020491 |
| gene-BPHYT_RS11530 | 422.995509 | 480.060338 | 523.610213 | 301.705958 | 327.693634 | 367.582609 | 0.51454398 | 0.00099997 | 0.00205832 |
| gene-BPHYT_RS34720 | 59.0597503 | 74.6760525 | 43.718911 | 25.6351467 | 26.1656514 | 25.3505247 | 1.20137937 | 0.00100562 | 0.00206878 |
| gene-BPHYT_RS30790 | 13.5677805 | 8.534406 | 12.2006263 | 47.3264248 | 27.4116348 | 30.78278 | -1.61403786 | 0.00100582 | 0.00206878 |
| gene-BPHYT_RS09105 | 1646.49007 | 1737.81842 | 1776.20785 | 1404.01727 | 1358.1219 | 1234.9327 | 0.36869265 | 0.00100859 | 0.00207367 |
| gene-BPHYT_RS02455 | 63.8483787 | 69.3420488 | 78.2873522 | 121.273963 | 140.796124 | 107.73973 | -0.80486224 | 0.00101509 | 0.00208626 |
| gene-BPHYT_RS34845 | 944.956004 | 970.788683 | 1007.56839 | 791.731647 | 645.4194 | 746.029728 | 0.4191875 | 0.00103022 | 0.00211654 |
| gene-BPHYT_RS09000 | 1333.63301 | 1218.28646 | 861.160874 | 726.657813 | 811.135192 | 816.649046 | 0.53661635 | 0.00104321 | 0.00214241 |
| gene-BPHYT_RS20965 | 19.1545136 | 16.0020113 | 7.11703202 | 2.95790155 | 0 | 0 | 3.79590624 | 0.00105329 | 0.00216228 |
| gene-BPHYT_RS07710 | 632.098949 | 664.616867 | 704.58617 | 502.843263 | 508.361227 | 484.376097 | 0.42010113 | 0.00105838 | 0.00217191 |
| gene-BPHYT_RS04860 | 47.886284 | 28.8036203 | 63.0365693 | 74.9335059 | 117.122439 | 101.402099 | -1.06416166 | 0.00106776 | 0.00218972 |
| gene-BPHYT_RS02990 | 16.7601994 | 28.8036203 | 26.4346903 | 52.2562607 | 59.8072031 | 53.4171771 | -1.21271902 | 0.00106787 | 0.00218972 |
| gene-BPHYT_RS24620 | 310.462741 | 234.696165 | 261.296747 | 171.55829 | 173.191692 | 188.318184 | 0.59920112 | 0.00107697 | 0.00220753 |
| gene-BPHYT_RS02125 | 642.47431 | 643.280852 | 561.22881 | 463.404576 | 444.816073 | 455.404069 | 0.43774308 | 0.00108463 | 0.00222239 |
| gene-BPHYT_RS02150 | 426.986032 | 468.325529 | 481.924739 | 621.159325 | 608.039898 | 621.087856 | -0.42762492 | 0.00108681 | 0.00222602 |
| gene-BPHYT_RS07185 | 123.706234 | 94.9452668 | 106.75548 | 65.073834 | 56.0692529 | 60.6601842 | 0.84081468 | 0.00108911 | 0.00222988 |
| gene-BPHYT_RS03170 | 9862.18019 | 9541.46591 | 8318.79371 | 7218.26574 | 7169.38847 | 7428.60912 | 0.34569055 | 0.00109658 | 0.00224433 |
| gene-BPHYT_RS29660 | 3274.62372 | 2944.37007 | 2993.22032 | 2397.87219 | 2389.79616 | 2501.55357 | 0.33795338 | 0.00110329 | 0.0022572 |
| gene-BPHYT_RS29035 | 119.71571 | 166.420917 | 187.07627 | 223.81455 | 261.656514 | 267.991261 | -0.67613412 | 0.0011135 | 0.00227724 |
| gene-BPHYT_RS05130 | 19.1545136 | 10.6680075 | 21.351096 | 1.97193437 | 3.7379502 | 2.71612765 | 2.62944609 | 0.00111585 | 0.00228117 |
| gene-BPHYT_RS20960 | 50.2805982 | 40.5384285 | 36.6018789 | 94.6528495 | 68.5290869 | 87.8214607 | -0.97583121 | 0.00113776 | 0.00232508 |
| gene-BPHYT_RS29250 | 103.753615 | 100.279271 | 104.722043 | 134.091537 | 164.469809 | 234.492354 | -0.79049923 | 0.00114019 | 0.00232915 |
| gene-BPHYT_RS28005 | 89.3877301 | 92.8116653 | 52.8693807 | 41.4106217 | 32.3955684 | 37.1204112 | 1.08078476 | 0.00114244 | 0.00233204 |
| gene-BPHYT_RS32675 | 273.749924 | 233.629364 | 247.062683 | 148.881045 | 189.389477 | 162.062283 | 0.59854889 | 0.00114246 | 0.00233204 |
| gene-BPHYT_RS33670 | 118.917605 | 98.145669 | 150.474391 | 176.488126 | 226.768979 | 200.08807 | -0.7123913 | 0.00114408 | 0.00233445 |
| gene-BPHYT_RS13690 | 1853.19919 | 1473.25184 | 1206.84529 | 2042.924 | 2206.6366 | 2131.25483 | -0.49207268 | 0.00115136 | 0.00234843 |
| gene-BPHYT_RS35720 | 44.6938651 | 72.542451 | 43.718911 | 21.691278 | 22.4277012 | 22.6343971 | 1.26466095 | 0.00115305 | 0.00235098 |
| gene-BPHYT_RS22125 | 359.14713 | 293.370206 | 300.948782 | 507.773099 | 385.00887 | 496.145984 | -0.54288685 | 0.0011684 | 0.00238137 |
| gene-BPHYT_RS08070 | 28811.5809 | 35063.6071 | 42602.5536 | 49844.5849 | 48779.0041 | 46682.9913 | -0.44858478 | 0.00116994 | 0.00238362 |
| gene-BPHYT_RS03825 | 169.198203 | 163.220515 | 131.156733 | 74.9335059 | 105.908589 | 95.0644677 | 0.75462069 | 0.00118644 | 0.00241633 |
| gene-BPHYT_RS08935 | 457.314012 | 530.199973 | 497.175522 | 677.359454 | 672.831035 | 641.006125 | -0.42467467 | 0.0012011 | 0.00244525 |
| gene-BPHYT_RS29730 | 67.0407976 | 67.2084473 | 114.889231 | 48.3123919 | 27.4116348 | 38.0257871 | 1.11931792 | 0.00120318 | 0.00244857 |
| gene-BPHYT_RS18915 | 15.9620947 | 16.0020113 | 40.6687544 | 8.87370464 | 2.4919668 | 2.71612765 | 2.34611363 | 0.00120661 | 0.00245463 |
| gene-BPHYT_RS00140 | 917.820443 | 979.323089 | 1214.97904 | 1325.13989 | 1667.12579 | 1345.38856 | -0.47871647 | 0.00121955 | 0.00247998 |
| gene-BPHYT_RS04325 | 993.640393 | 1206.55165 | 1399.00515 | 1870.37975 | 1526.32966 | 1612.47445 | -0.47822085 | 0.00122 | 0.00247998 |
| gene-BPHYT_RS32835 | 101.359301 | 110.947278 | 79.304071 | 49.2983591 | 28.6576182 | 63.3763118 | 1.0289861 | 0.00123208 | 0.00250359 |
| gene-BPHYT_RS33240 | 701.534061 | 1090.27037 | 1081.78887 | 612.28562 | 715.194471 | 616.560976 | 0.56329493 | 0.00124915 | 0.00253734 |
| gene-BPHYT_RS30350 | 353.560397 | 328.574631 | 328.400192 | 247.477763 | 241.720779 | 224.533219 | 0.50367373 | 0.00125001 | 0.00253813 |
| gene-BPHYT_RS36735 | 57.4635408 | 70.4088495 | 54.9028184 | 26.6211139 | 21.1817178 | 34.4042836 | 1.13732187 | 0.00128586 | 0.00260994 |
| gene-BPHYT_RS18930 | 600.174759 | 586.740413 | 634.432568 | 816.380827 | 1131.35293 | 714.341572 | -0.54527482 | 0.00128747 | 0.00261223 |
| gene-BPHYT_RS35060 | 296.096856 | 210.159748 | 301.965501 | 153.81088 | 161.977842 | 197.371943 | 0.65501138 | 0.00129958 | 0.00263581 |
| gene-BPHYT_RS15740 | 5008.90531 | 7709.76902 | 6964.52419 | 5171.39787 | 4101.77735 | 4580.29659 | 0.50630309 | 0.00130019 | 0.00263606 |
| gene-BPHYT_RS17070 | 118.119501 | 103.479673 | 115.90595 | 56.2001294 | 79.7429375 | 54.322553 | 0.83933527 | 0.00130171 | 0.00263813 |
| gene-BPHYT_RS09050 | 10552.5408 | 9705.75323 | 9902.84169 | 7292.21328 | 8680.76634 | 7755.44981 | 0.34634122 | 0.00130428 | 0.00264236 |
| gene-BPHYT_RS18210 | 697.543537 | 539.80118 | 659.85054 | 888.356431 | 827.332977 | 863.728592 | -0.44194402 | 0.00131453 | 0.00266212 |
| gene-BPHYT_RS10670 | 168.400099 | 201.625342 | 339.584099 | 120.287996 | 152.009975 | 127.658 | 0.82753807 | 0.00132315 | 0.00267856 |
| gene-BPHYT_RS34225 | 1546.72697 | 1372.97257 | 1175.327 | 923.85125 | 1024.19835 | 1074.68117 | 0.43874363 | 0.00134627 | 0.00272436 |
| gene-BPHYT_RS33630 | 31.1260846 | 48.0060338 | 34.5684412 | 92.6809151 | 63.5451533 | 74.2408224 | -1.03146147 | 0.00134995 | 0.00273078 |
| gene-BPHYT_RS16370 | 410.225833 | 322.173827 | 388.386604 | 242.547927 | 229.260945 | 291.531034 | 0.55272298 | 0.00135646 | 0.00274291 |
| gene-BPHYT_RS16195 | 366.330073 | 280.568597 | 276.54753 | 208.039076 | 191.881443 | 218.195588 | 0.58023164 | 0.00136952 | 0.00276828 |
| gene-BPHYT_RS04875 | 77.4161591 | 60.8076428 | 64.0532881 | 117.330095 | 98.4326885 | 142.144014 | -0.82326671 | 0.00140674 | 0.00284245 |
| gene-BPHYT_RS10505 | 43.8957603 | 45.8724323 | 57.952975 | 24.6491796 | 22.4277012 | 14.4860141 | 1.26946341 | 0.00141654 | 0.0028612 |
| gene-BPHYT_RS24470 | 67.8389023 | 68.275248 | 111.839075 | 126.203799 | 169.453742 | 145.765517 | -0.83295061 | 0.00142255 | 0.00287224 |
| gene-BPHYT_RS35680 | 403.04289 | 344.576642 | 305.015658 | 434.811527 | 565.676463 | 522.401885 | -0.52920971 | 0.00144113 | 0.00290868 |
| gene-BPHYT_RS10930 | 64.6464834 | 90.6780638 | 68.1201636 | 39.4386873 | 33.6415518 | 38.0257871 | 0.99978801 | 0.00145098 | 0.00292747 |
| gene-BPHYT_RS07530 | 154.832318 | 152.552507 | 115.90595 | 75.919473 | 93.4487549 | 83.2945812 | 0.74984418 | 0.00146718 | 0.00295903 |
| gene-BPHYT_RS19990 | 27.1355609 | 41.6052293 | 46.7690675 | 79.8633418 | 68.5290869 | 82.3892054 | -1.01291684 | 0.00147674 | 0.00297721 |
| gene-BPHYT_RS23170 | 285.721495 | 406.451086 | 274.514092 | 237.618091 | 208.079228 | 161.156907 | 0.67160451 | 0.00147837 | 0.00297938 |
| gene-BPHYT_RS08130 | 181.169774 | 195.224537 | 220.627993 | 110.428324 | 158.239892 | 94.1590918 | 0.72698496 | 0.00148479 | 0.0029912 |
| gene-BPHYT_RS23125 | 89.3877301 | 167.487718 | 87.4378219 | 60.1439981 | 68.5290869 | 27.1612765 | 1.1515547 | 0.00148886 | 0.00299829 |
| gene-BPHYT_RS12730 | 564.260046 | 736.092518 | 639.516163 | 907.089808 | 791.199458 | 969.657571 | -0.46272968 | 0.00149975 | 0.0030191 |
| gene-BPHYT_RS04705 | 201.920498 | 201.625342 | 220.627993 | 105.498489 | 145.780058 | 147.576269 | 0.64738665 | 0.00150278 | 0.00302406 |
| gene-BPHYT_RS15635 | 1497.24448 | 1693.01279 | 2168.66133 | 2761.69408 | 2386.05821 | 2290.60098 | -0.47363455 | 0.00151433 | 0.00304616 |
| gene-BPHYT_RS23090 | 34.3185035 | 43.7388308 | 31.5182846 | 93.6668823 | 87.2188379 | 50.7010495 | -1.0780249 | 0.00156895 | 0.00315485 |
| gene-BPHYT_RS34420 | 586.606979 | 564.337597 | 653.750227 | 487.067788 | 439.83214 | 360.339601 | 0.48860758 | 0.00157368 | 0.00316318 |
| gene-BPHYT_RS12975 | 32.7222941 | 38.404827 | 34.5684412 | 15.7754749 | 12.459834 | 9.95913471 | 1.46918222 | 0.00157636 | 0.00316739 |
| gene-BPHYT_RS28060 | 10.3753615 | 10.6680075 | 7.11703202 | 0 | 1.2459834 | 0 | 4.66174332 | 0.00158306 | 0.00317968 |
| gene-BPHYT_RS09620 | 971.29346 | 960.120675 | 949.615415 | 811.450991 | 713.948487 | 631.04699 | 0.41846136 | 0.00160651 | 0.00322558 |
| gene-BPHYT_RS34105 | 558.673313 | 354.177849 | 386.353167 | 660.598012 | 604.301948 | 651.870636 | -0.55803947 | 0.00161157 | 0.00323452 |
| gene-BPHYT_RS21225 | 466.891269 | 454.45712 | 585.630063 | 731.587649 | 621.745716 | 738.786721 | -0.4753284 | 0.00163017 | 0.00327064 |
| gene-BPHYT_RS15065 | 427.784137 | 588.874014 | 428.03864 | 355.934153 | 331.431584 | 306.017048 | 0.53904393 | 0.00163257 | 0.00327424 |
| gene-BPHYT_RS03040 | 284.92339 | 193.090936 | 294.848469 | 171.55829 | 181.913576 | 106.834354 | 0.75459938 | 0.00164896 | 0.00330589 |
| gene-BPHYT_RS29360 | 27.1355609 | 20.2692143 | 16.2675018 | 4.92983591 | 1.2459834 | 7.24300706 | 2.19909001 | 0.00165423 | 0.00331521 |
| gene-BPHYT_RS23105 | 788.527476 | 727.558112 | 648.666632 | 459.460707 | 575.64433 | 544.130906 | 0.45711464 | 0.00165929 | 0.00332412 |
| gene-BPHYT_RS37230 | 79.8104733 | 85.34406 | 87.4378219 | 122.259931 | 145.780058 | 143.954765 | -0.70650437 | 0.00166549 | 0.00333531 |
| gene-BPHYT_RS29465 | 7.1829426 | 12.801609 | 17.2842206 | 48.3123919 | 24.919668 | 36.2150353 | -1.57938643 | 0.00168782 | 0.00337878 |
| gene-BPHYT_RS33250 | 290.510123 | 326.44103 | 343.650974 | 410.162348 | 550.724662 | 421.905162 | -0.52414363 | 0.0016986 | 0.00339909 |
| gene-BPHYT_RS03785 | 63.8483787 | 112.014079 | 105.738761 | 194.235535 | 134.566207 | 162.967659 | -0.8154103 | 0.00170101 | 0.00340265 |
| gene-BPHYT_RS07665 | 798.104733 | 737.159318 | 680.184917 | 562.001294 | 551.970646 | 561.333047 | 0.40393834 | 0.00171199 | 0.00342334 |
| gene-BPHYT_RS04935 | 1086.22054 | 1196.95044 | 965.882916 | 1565.71589 | 1313.2665 | 1428.68314 | -0.40764943 | 0.00171461 | 0.00342732 |
| gene-BPHYT_RS22275 | 90.9839396 | 101.346071 | 77.2706333 | 156.768782 | 147.026041 | 134.901007 | -0.70210901 | 0.00171674 | 0.00343031 |
| gene-BPHYT_RS32650 | 2160.46951 | 2106.93148 | 2342.52025 | 2740.98877 | 2834.61223 | 2705.26314 | -0.32501384 | 0.00172134 | 0.00343822 |
| gene-BPHYT_RS11205 | 1090.21107 | 1155.34521 | 1367.48687 | 910.047709 | 968.129101 | 818.459798 | 0.42256406 | 0.00172677 | 0.00344778 |
| gene-BPHYT_RS13865 | 236.239001 | 350.977447 | 234.862057 | 201.137305 | 160.731858 | 153.9139 | 0.66940811 | 0.0017285 | 0.00344996 |
| gene-BPHYT_RS24825 | 209.901545 | 176.022124 | 211.477523 | 312.551597 | 245.45873 | 331.367573 | -0.57603892 | 0.00174889 | 0.00348866 |
| gene-BPHYT_RS15895 | 1237.86044 | 1221.48686 | 1180.4106 | 1460.2174 | 1611.05653 | 1543.66588 | -0.34184466 | 0.00174918 | 0.00348866 |
| gene-BPHYT_RS28105 | 770.171068 | 873.709814 | 831.676027 | 1068.78843 | 1044.13409 | 1076.49193 | -0.3668278 | 0.00175074 | 0.00349049 |
| gene-BPHYT_RS16880 | 11191.0246 | 13138.718 | 13093.3055 | 15025.1539 | 16043.2822 | 16124.7445 | -0.33475725 | 0.00176625 | 0.0035201 |
| gene-BPHYT_RS18405 | 177.179251 | 116.281282 | 197.243459 | 235.646157 | 272.870364 | 270.707389 | -0.6637872 | 0.00181352 | 0.00361297 |
| gene-BPHYT_RS14335 | 414.216357 | 322.173827 | 218.594555 | 656.654143 | 418.650422 | 484.376097 | -0.706186 | 0.00181829 | 0.00362113 |
| gene-BPHYT_RS07860 | 22.3469325 | 29.870421 | 43.718911 | 61.1299653 | 61.0531865 | 81.4838295 | -1.09982175 | 0.00184312 | 0.00366923 |
| gene-BPHYT_RS09965 | 210.69965 | 163.220515 | 144.374078 | 260.295336 | 267.886431 | 258.937503 | -0.59709971 | 0.00184464 | 0.00367091 |
| gene-BPHYT_RS04735 | 808.480095 | 918.515446 | 2339.4701 | 3714.13838 | 3780.31363 | 4276.0903 | -1.53349208 | 0.00186262 | 0.00370533 |
| gene-BPHYT_RS13740 | 890.684882 | 741.426521 | 685.268511 | 1053.99892 | 968.129101 | 1099.12632 | -0.42878872 | 0.00188345 | 0.00374539 |
| gene-BPHYT_RS21010 | 36.7128177 | 54.4068383 | 36.6018789 | 16.7614421 | 14.9518008 | 19.0128935 | 1.32027276 | 0.00189068 | 0.00375838 |
| gene-BPHYT_RS18830 | 59.0597503 | 56.5404398 | 74.2204767 | 95.6388167 | 135.81219 | 105.023602 | -0.82196614 | 0.00191482 | 0.00380496 |
| gene-BPHYT_RS34795 | 471.679897 | 403.250684 | 442.272704 | 310.579662 | 330.185601 | 322.313814 | 0.45390038 | 0.00192495 | 0.00382368 |
| gene-BPHYT_RS26870 | 35.914713 | 46.939233 | 77.2706333 | 27.6070811 | 22.4277012 | 9.95913471 | 1.42233637 | 0.00194205 | 0.00385623 |
| gene-BPHYT_RS28935 | 1927.42293 | 1961.84658 | 2089.35726 | 1622.90198 | 1558.72523 | 1592.55618 | 0.32415809 | 0.00194539 | 0.00386143 |
| gene-BPHYT_RS22230 | 100.561196 | 107.746876 | 123.022982 | 70.0036699 | 71.0210537 | 43.4580424 | 0.85171148 | 0.00196462 | 0.00389818 |
| gene-BPHYT_RS11570 | 283.32718 | 322.173827 | 344.667693 | 498.899394 | 412.420505 | 419.189034 | -0.48856484 | 0.00197082 | 0.00390903 |
| gene-BPHYT_RS17575 | 145.255061 | 152.552507 | 128.106576 | 212.968911 | 232.998896 | 195.561191 | -0.58807012 | 0.00197553 | 0.00391694 |
| gene-BPHYT_RS34670 | 27.1355609 | 27.7368195 | 31.5182846 | 71.9756043 | 52.3313027 | 55.2279289 | -1.05998665 | 0.00201004 | 0.00398389 |
| gene-BPHYT_RS22760 | 38.3090272 | 30.9372218 | 65.070007 | 76.9054402 | 87.2188379 | 105.023602 | -1.00667987 | 0.00203607 | 0.00403401 |
| gene-BPHYT_RS03320 | 1.59620947 | 2.1336015 | 0 | 7.88773746 | 14.9518008 | 13.5806382 | -3.27194493 | 0.0020525 | 0.00406506 |
| gene-BPHYT_RS17260 | 2422.24787 | 2518.71657 | 2914.93297 | 3426.23596 | 3462.58786 | 3154.32958 | -0.35443922 | 0.00205715 | 0.00407279 |
| gene-BPHYT_RS02825 | 1114.15421 | 1096.67117 | 1022.81917 | 1428.66645 | 1298.3147 | 1387.94123 | -0.34795215 | 0.00206851 | 0.00409376 |
| gene-BPHYT_RS12970 | 3892.35678 | 4620.31405 | 4881.26724 | 3670.75582 | 3386.58288 | 3329.06712 | 0.3665334 | 0.00208223 | 0.00411941 |
| gene-BPHYT_RS20950 | 61.4540645 | 58.6740413 | 54.9028184 | 33.5228842 | 22.4277012 | 27.1612765 | 1.06774167 | 0.00210429 | 0.00416152 |
| gene-BPHYT_RS15755 | 482.853364 | 534.467176 | 390.420042 | 650.73834 | 622.991699 | 661.829771 | -0.45990596 | 0.00210752 | 0.00416638 |
| gene-BPHYT_RS17970 | 375.907329 | 457.657522 | 328.400192 | 263.253238 | 272.870364 | 273.423517 | 0.52056459 | 0.00211778 | 0.00418514 |
| gene-BPHYT_RS34550 | 37.5109225 | 35.2044248 | 31.5182846 | 71.9756043 | 62.2991699 | 68.8085671 | -0.96146137 | 0.00212114 | 0.00419024 |
| gene-BPHYT_RS08990 | 4903.55548 | 10057.7975 | 5050.04257 | 14061.864 | 15526.1991 | 14319.425 | -1.13370126 | 0.0021242 | 0.00419474 |
| gene-BPHYT_RS12330 | 27.9336657 | 32.0040225 | 25.4179715 | 12.8175734 | 2.4919668 | 9.05375883 | 1.77207463 | 0.0021431 | 0.00423052 |
| gene-BPHYT_RS14620 | 418.20688 | 487.527943 | 370.085665 | 636.9348 | 519.575077 | 618.371728 | -0.47862761 | 0.00215613 | 0.00425468 |
| gene-BPHYT_RS05510 | 312.857055 | 349.910646 | 259.263309 | 436.783462 | 436.094189 | 419.189034 | -0.48668652 | 0.0022042 | 0.00434795 |
| gene-BPHYT_RS34920 | 941.763585 | 951.586269 | 881.495251 | 1143.72193 | 1181.19226 | 1196.00154 | -0.34339191 | 0.00225675 | 0.00444999 |
| gene-BPHYT_RS08675 | 169.996308 | 112.014079 | 112.855793 | 59.1580309 | 82.2349043 | 83.2945812 | 0.82057302 | 0.00226513 | 0.00446488 |
| gene-BPHYT_RS19820 | 21.5488278 | 25.603218 | 13.2173452 | 5.91580309 | 3.7379502 | 4.52687942 | 2.07894385 | 0.00228333 | 0.0044991 |
| gene-BPHYT_RS25840 | 28.7317704 | 17.068812 | 32.5350035 | 4.92983591 | 0 | 11.7698865 | 2.16876779 | 0.00229096 | 0.0045125 |
| gene-BPHYT_RS18940 | 242.623839 | 217.627353 | 236.895494 | 311.56563 | 413.666488 | 296.96329 | -0.54693345 | 0.00229998 | 0.00452861 |
| gene-BPHYT_RS38690 | 781.344534 | 699.821292 | 797.107586 | 543.267917 | 527.050978 | 635.57387 | 0.41616133 | 0.00232102 | 0.00456837 |
| gene-BPHYT_RS30820 | 259.384038 | 290.169804 | 291.798313 | 363.82189 | 396.222721 | 395.649261 | -0.45948008 | 0.00233255 | 0.00458939 |
| gene-BPHYT_RS08650 | 46.2900745 | 42.67203 | 64.0532881 | 22.6772452 | 21.1817178 | 24.4451488 | 1.15810526 | 0.00233962 | 0.00460162 |
| gene-BPHYT_RS12780 | 10653.9001 | 11589.7234 | 11634.3139 | 9115.2666 | 9642.66552 | 8651.77194 | 0.30571362 | 0.00234888 | 0.00461816 |
| gene-BPHYT_RS02430 | 918.618548 | 768.09654 | 914.030255 | 1073.71826 | 1105.18727 | 1241.27034 | -0.39482474 | 0.0023818 | 0.00468117 |
| gene-BPHYT_RS04895 | 99.7630917 | 97.0788683 | 106.75548 | 151.838946 | 149.518008 | 169.30529 | -0.63459816 | 0.00238336 | 0.00468253 |
| gene-BPHYT_RS09760 | 27.9336657 | 23.4696165 | 25.4179715 | 4.92983591 | 13.7058174 | 2.71612765 | 1.90785086 | 0.0024226 | 0.00475789 |
| gene-BPHYT_RS23255 | 177.179251 | 182.422928 | 185.042832 | 226.772452 | 301.527982 | 268.896637 | -0.54759803 | 0.00243737 | 0.00478504 |
| gene-BPHYT_RS33735 | 375.109225 | 406.451086 | 386.353167 | 236.632124 | 284.084215 | 310.543928 | 0.48953377 | 0.0024382 | 0.00478504 |
| gene-BPHYT_RS35070 | 290.510123 | 294.437007 | 306.032377 | 185.36183 | 237.982829 | 200.993446 | 0.51684973 | 0.00244221 | 0.00479116 |
| gene-BPHYT_RS11275 | 199.526183 | 204.825744 | 197.243459 | 128.175734 | 132.07424 | 144.860141 | 0.56871876 | 0.00244442 | 0.00479375 |
| gene-BPHYT_RS22025 | 47.886284 | 40.5384285 | 50.835943 | 16.7614421 | 9.96786719 | 28.0666524 | 1.32057042 | 0.00247508 | 0.00485133 |
| gene-BPHYT_RS17245 | 1633.72039 | 1718.61601 | 1861.61223 | 2135.60492 | 2163.02718 | 2237.18381 | -0.32651354 | 0.00247557 | 0.00485133 |
| gene-BPHYT_RS09785 | 103.753615 | 65.0748458 | 128.106576 | 72.9615715 | 26.1656514 | 33.4989077 | 1.15617368 | 0.00248378 | 0.00486565 |
| gene-BPHYT_RS17805 | 30.3279799 | 23.4696165 | 27.4514092 | 9.85967182 | 7.47590039 | 9.05375883 | 1.61739295 | 0.00248561 | 0.00486746 |
| gene-BPHYT_RS12665 | 244.220048 | 196.291338 | 252.146277 | 173.530224 | 135.81219 | 149.387021 | 0.59325785 | 0.00249943 | 0.00489275 |
| gene-BPHYT_RS35620 | 861.953112 | 646.481255 | 506.325992 | 408.190414 | 408.682555 | 525.118012 | 0.58653323 | 0.00254334 | 0.0049769 |
| gene-BPHYT_RS09140 | 391.071319 | 395.783078 | 443.289423 | 225.786485 | 245.45873 | 354.907346 | 0.57056021 | 0.00255904 | 0.00500581 |
| gene-BPHYT_RS18815 | 425.389823 | 375.513864 | 421.938327 | 505.801165 | 721.424388 | 516.064253 | -0.50788102 | 0.00258788 | 0.00506038 |
| gene-BPHYT_RS08565 | 445.342441 | 492.861947 | 523.610213 | 767.082468 | 634.20555 | 593.021203 | -0.44945573 | 0.00260035 | 0.00508292 |
| gene-BPHYT_RS07915 | 681.581442 | 564.337597 | 629.348974 | 437.769429 | 459.767874 | 501.578239 | 0.42325484 | 0.00260758 | 0.00509522 |
| gene-BPHYT_RS11505 | 369.522492 | 330.708233 | 334.500505 | 443.685232 | 490.917459 | 461.7417 | -0.42968434 | 0.00260949 | 0.0050971 |
| gene-BPHYT_RS17550 | 1606.58483 | 1721.81641 | 1518.97798 | 2249.97711 | 2037.18286 | 1902.19473 | -0.35264573 | 0.00261062 | 0.00509747 |
| gene-BPHYT_RS20620 | 68.6370071 | 67.2084473 | 70.1536013 | 41.4106217 | 31.149585 | 34.4042836 | 0.94077226 | 0.0026399 | 0.00515278 |
| gene-BPHYT_RS33750 | 395.859948 | 361.645454 | 347.71785 | 456.502805 | 556.954579 | 493.429856 | -0.44399493 | 0.00264684 | 0.00516445 |
| gene-BPHYT_RS04855 | 62.2521692 | 80.0100563 | 102.688605 | 146.90911 | 128.33629 | 133.995631 | -0.74822611 | 0.00265366 | 0.00517589 |
| gene-BPHYT_RS26865 | 7.1829426 | 10.6680075 | 20.3343772 | 0.98596718 | 0 | 2.71612765 | 3.25895951 | 0.00265529 | 0.00517695 |
| gene-BPHYT_RS33315 | 196.333764 | 211.226549 | 132.173452 | 72.9615715 | 135.81219 | 103.212851 | 0.79824104 | 0.00265612 | 0.00517695 |
| gene-BPHYT_RS04205 | 1444.56957 | 1613.00273 | 1638.9508 | 1784.6006 | 2126.89366 | 2148.45697 | -0.3680484 | 0.00266708 | 0.00519642 |
| gene-BPHYT_RS21780 | 58.2616455 | 86.4108608 | 54.9028184 | 11.8316062 | 17.4437676 | 47.0795459 | 1.36665 | 0.00266954 | 0.00519934 |
| gene-BPHYT_RS07850 | 152.438004 | 190.957334 | 183.009395 | 105.498489 | 130.828257 | 101.402099 | 0.6433734 | 0.00267352 | 0.0052052 |
| gene-BPHYT_RS21660 | 59.0597503 | 56.5404398 | 46.7690675 | 21.691278 | 19.9357344 | 32.5935318 | 1.11833502 | 0.00269058 | 0.00523654 |
| gene-BPHYT_RS06930 | 1850.80488 | 859.841405 | 2157.47742 | 3305.94796 | 3749.16405 | 3881.34641 | -1.167559 | 0.00271925 | 0.00529044 |
| gene-BPHYT_RS04900 | 142.062643 | 98.145669 | 82.3542276 | 170.572323 | 159.485875 | 207.331077 | -0.73145292 | 0.00272044 | 0.00529083 |
| gene-BPHYT_RS07720 | 79.0123686 | 107.746876 | 90.4879785 | 52.2562607 | 42.3634355 | 57.9440565 | 0.84981586 | 0.00272966 | 0.00530685 |
| gene-BPHYT_RS29870 | 127.696757 | 160.020113 | 217.577836 | 96.6247839 | 110.892523 | 96.8752195 | 0.7304371 | 0.00274372 | 0.00533226 |
| gene-BPHYT_RS17640 | 1880.33475 | 2173.07313 | 2380.13885 | 2834.65565 | 2728.70364 | 2677.19649 | -0.35772425 | 0.00275023 | 0.00534116 |
| gene-BPHYT_RS30405 | 88.5896254 | 51.206436 | 73.2037579 | 15.7754749 | 39.8714688 | 39.8365389 | 1.16749661 | 0.00275028 | 0.00534116 |
| gene-BPHYT_RS14075 | 257.787829 | 244.297372 | 221.644711 | 361.849956 | 336.415518 | 313.260056 | -0.48105381 | 0.00276456 | 0.00536697 |
| gene-BPHYT_RS16010 | 517.171867 | 490.728345 | 373.135821 | 676.373487 | 741.360122 | 549.563161 | -0.50759582 | 0.00279486 | 0.00542383 |
| gene-BPHYT_RS19425 | 606.559597 | 682.75248 | 605.96444 | 739.475387 | 882.156246 | 881.83611 | -0.40181293 | 0.00280407 | 0.00543838 |
| gene-BPHYT_RS31175 | 256.191619 | 279.501797 | 196.22674 | 151.838946 | 176.929643 | 158.44078 | 0.58992969 | 0.00280437 | 0.00543838 |
| gene-BPHYT_RS07610 | 2591.44607 | 2065.32625 | 2258.13259 | 2950.01381 | 3020.26376 | 2860.08241 | -0.35194056 | 0.00282398 | 0.00547445 |
| gene-BPHYT_RS19635 | 486.843887 | 595.274819 | 552.078341 | 717.784109 | 745.098072 | 692.612551 | -0.40068076 | 0.00282952 | 0.0054832 |
| gene-BPHYT_RS13015 | 39.9052367 | 19.2024135 | 21.351096 | 8.87370464 | 6.22991699 | 9.05375883 | 1.73334673 | 0.0028502 | 0.00552131 |
| gene-BPHYT_RS29690 | 293.702542 | 198.42494 | 258.24659 | 154.796848 | 184.405543 | 157.535404 | 0.6015784 | 0.00286303 | 0.00554416 |
| gene-BPHYT_RS16930 | 577.029722 | 650.748458 | 650.70007 | 769.054402 | 816.119126 | 854.674834 | -0.37866055 | 0.00287518 | 0.0055657 |
| gene-BPHYT_RS04695 | 648.061043 | 627.278841 | 573.429437 | 492.983591 | 458.521891 | 453.593317 | 0.39634417 | 0.00287923 | 0.00557154 |
| gene-BPHYT_RS19500 | 447.736755 | 339.242639 | 260.280028 | 187.333765 | 234.244879 | 252.599871 | 0.63817866 | 0.0028838 | 0.00557838 |
| gene-BPHYT_RS27065 | 61.4540645 | 37.3380263 | 30.5015658 | 20.7053108 | 7.47590039 | 19.0128935 | 1.43831595 | 0.00288947 | 0.00558571 |
| gene-BPHYT_RS08875 | 1368.74962 | 1498.85505 | 1578.96439 | 1854.60427 | 1891.4028 | 1836.10229 | -0.32860194 | 0.00288966 | 0.00558571 |
| gene-BPHYT_RS08065 | 10987.5079 | 13947.353 | 17796.6469 | 20247.8221 | 19753.8208 | 18710.498 | -0.45844696 | 0.00289801 | 0.00559984 |
| gene-BPHYT_RS03860 | 21.5488278 | 36.2712255 | 35.5851601 | 0.98596718 | 17.4437676 | 6.33763118 | 1.9605425 | 0.0029017 | 0.00560497 |
| gene-BPHYT_RS14295 | 111.734663 | 133.350094 | 134.206889 | 73.9475387 | 82.2349043 | 77.8623259 | 0.695519 | 0.00290639 | 0.00561202 |
| gene-BPHYT_RS22490 | 1558.69854 | 860.908205 | 519.543337 | 344.102547 | 399.960671 | 406.513772 | 1.35354005 | 0.00293873 | 0.00567078 |
| gene-BPHYT_RS27120 | 94.9744633 | 108.813677 | 76.2539145 | 62.1159325 | 47.3473691 | 45.2687942 | 0.85403752 | 0.00293893 | 0.00567078 |
| gene-BPHYT_RS25075 | 27.1355609 | 22.4028158 | 23.3845338 | 5.91580309 | 7.47590039 | 9.05375883 | 1.69891313 | 0.00294377 | 0.0056781 |
| gene-BPHYT_RS08110 | 56592.8085 | 58275.0578 | 54011.156 | 64761.2824 | 71662.7352 | 69565.4613 | -0.28657061 | 0.00299108 | 0.00576729 |
| gene-BPHYT_RS08445 | 268.961295 | 211.226549 | 308.065814 | 196.207469 | 180.667593 | 132.184879 | 0.63457534 | 0.00300483 | 0.00579173 |
| gene-BPHYT_RS35285 | 421.399299 | 487.527943 | 394.486917 | 541.295983 | 590.596131 | 621.087856 | -0.42856859 | 0.00300635 | 0.00579259 |
| gene-BPHYT_RS17520 | 203.516707 | 291.236605 | 296.881907 | 205.081174 | 135.81219 | 162.062283 | 0.64768289 | 0.00301522 | 0.00580761 |
| gene-BPHYT_RS04690 | 73.4256355 | 66.1416465 | 113.872512 | 28.5930483 | 46.1013857 | 51.6064253 | 1.0039844 | 0.00305056 | 0.00587358 |
| gene-BPHYT_RS03410 | 1201.94573 | 1294.02931 | 1294.28311 | 1626.84585 | 1527.57565 | 1578.07016 | -0.32113306 | 0.00305758 | 0.005885 |
| gene-BPHYT_RS03280 | 399.052367 | 487.527943 | 554.111778 | 589.608375 | 680.306936 | 725.206082 | -0.47184272 | 0.00306355 | 0.00589439 |
| gene-BPHYT_RS05535 | 975.283984 | 985.723893 | 967.916354 | 1317.25216 | 1143.81276 | 1249.41872 | -0.34209198 | 0.00307075 | 0.00590614 |
| gene-BPHYT_RS06095 | 5852.50201 | 6644.03507 | 6582.2379 | 5073.78712 | 5265.52584 | 5138.00814 | 0.30168334 | 0.00307328 | 0.00590889 |
| gene-BPHYT_RS09175 | 1038.33426 | 1662.07557 | 2064.956 | 1088.50777 | 1021.70639 | 1076.49193 | 0.57947851 | 0.00307741 | 0.00591471 |
| gene-BPHYT_RS28855 | 14.3658852 | 16.0020113 | 20.3343772 | 35.4948186 | 42.3634355 | 41.6472906 | -1.24201417 | 0.0031285 | 0.00601077 |
| gene-BPHYT_RS03655 | 172.390622 | 109.880477 | 94.5548539 | 77.8914074 | 64.7911367 | 69.713943 | 0.83121072 | 0.00312991 | 0.00601134 |
| gene-BPHYT_RS26265 | 35.914713 | 20.2692143 | 31.5182846 | 6.90177028 | 6.22991699 | 14.4860141 | 1.64788384 | 0.00313336 | 0.00601582 |
| gene-BPHYT_RS02670 | 632.098949 | 671.017672 | 464.640519 | 493.969558 | 363.827152 | 351.285843 | 0.54763472 | 0.00313737 | 0.00602046 |
| gene-BPHYT_RS04680 | 169.996308 | 209.092947 | 196.22674 | 140.993307 | 110.892523 | 125.847248 | 0.60072347 | 0.003138 | 0.00602046 |
| gene-BPHYT_RS10250 | 86.9934159 | 86.4108608 | 131.156733 | 53.2422278 | 64.7911367 | 52.5118012 | 0.84000821 | 0.00315598 | 0.00605279 |
| gene-BPHYT_RS20930 | 211.497754 | 210.159748 | 156.574704 | 123.245898 | 134.566207 | 121.320368 | 0.6126967 | 0.00317962 | 0.00609596 |
| gene-BPHYT_RS21370 | 224.26743 | 218.694154 | 200.293615 | 273.11291 | 306.511916 | 325.029942 | -0.49114647 | 0.00318476 | 0.00610365 |
| gene-BPHYT_RS16485 | 357.550921 | 325.374229 | 310.099252 | 508.759066 | 418.650422 | 430.95892 | -0.45137206 | 0.00319901 | 0.00612879 |
| gene-BPHYT_RS03525 | 597.780445 | 663.550067 | 625.282099 | 803.563254 | 779.985607 | 838.378068 | -0.3619621 | 0.00322768 | 0.00618151 |
| gene-BPHYT_RS34185 | 18255.8477 | 20020.6497 | 17008.6898 | 30586.6739 | 20132.5998 | 24028.6759 | -0.4352094 | 0.0032335 | 0.00619047 |
| gene-BPHYT_RS26670 | 665.619348 | 533.400375 | 683.235073 | 459.460707 | 505.86926 | 417.378282 | 0.44794902 | 0.00323619 | 0.00619342 |
| gene-BPHYT_RS18695 | 134.8797 | 151.485707 | 94.5548539 | 184.375863 | 196.865377 | 213.668708 | -0.64329449 | 0.00324297 | 0.00620419 |
| gene-BPHYT_RS01735 | 287.317704 | 308.305417 | 312.13269 | 211.982944 | 218.047095 | 224.533219 | 0.47037218 | 0.00325015 | 0.00621573 |
| gene-BPHYT_RS04905 | 52.6749124 | 44.8056315 | 71.1703202 | 32.536917 | 26.1656514 | 7.24300706 | 1.36799725 | 0.00325226 | 0.00621755 |
| gene-BPHYT_RS16830 | 615.338749 | 560.070394 | 616.131629 | 786.801811 | 782.477574 | 739.692096 | -0.36492272 | 0.00328235 | 0.00627286 |
| gene-BPHYT_RS11295 | 542.711219 | 549.402386 | 623.248661 | 469.320379 | 393.730754 | 417.378282 | 0.42000467 | 0.00328571 | 0.00627706 |
| gene-BPHYT_RS19630 | 104.55172 | 147.218504 | 164.708455 | 253.393566 | 204.341277 | 193.750439 | -0.65142727 | 0.00329733 | 0.00629702 |
| gene-BPHYT_RS05115 | 69.4351118 | 54.4068383 | 92.5214162 | 32.536917 | 44.8554023 | 32.5935318 | 0.98615129 | 0.00332854 | 0.00635438 |
| gene-BPHYT_RS19045 | 502.007877 | 445.922714 | 453.456611 | 521.576639 | 740.114139 | 678.126536 | -0.46664722 | 0.00333256 | 0.00635981 |
| gene-BPHYT_RS09270 | 2325.67719 | 2186.94154 | 2148.32695 | 1677.13018 | 1901.37067 | 1762.76684 | 0.31940499 | 0.00334559 | 0.0063824 |
| gene-BPHYT_RS15990 | 761.391916 | 582.47321 | 569.362561 | 458.47474 | 493.409426 | 448.161062 | 0.45368226 | 0.00336118 | 0.00640988 |
| Novel00761 | 322.434312 | 305.105015 | 312.13269 | 410.162348 | 402.452638 | 447.255686 | -0.42350316 | 0.00339053 | 0.00646357 |
| gene-BPHYT_RS21305 | 247.412467 | 256.03218 | 218.594555 | 168.600388 | 150.763991 | 179.264425 | 0.53090799 | 0.00340396 | 0.00648688 |
| gene-BPHYT_RS24585 | 166.803889 | 176.022124 | 185.042832 | 209.025043 | 279.100281 | 287.909531 | -0.55634719 | 0.00342952 | 0.00653328 |
| gene-BPHYT_RS18865 | 245.816258 | 262.432985 | 273.497373 | 343.116579 | 404.944605 | 334.083701 | -0.46762874 | 0.00343098 | 0.00653376 |
| gene-BPHYT_RS07715 | 808.480095 | 920.649047 | 780.840084 | 576.790802 | 660.371201 | 674.505033 | 0.3925333 | 0.00343401 | 0.00653722 |
| gene-BPHYT_RS17295 | 5420.72735 | 3613.25414 | 2911.88281 | 2873.10837 | 2787.26486 | 2578.51052 | 0.53647181 | 0.00344103 | 0.00654828 |
| gene-BPHYT_RS07020 | 233.046582 | 209.092947 | 205.37721 | 140.993307 | 149.518008 | 155.724652 | 0.5386285 | 0.00344291 | 0.00654955 |
| gene-BPHYT_RS09055 | 3711.18701 | 4146.65452 | 4223.45014 | 5211.82253 | 4780.8383 | 4954.21683 | -0.3074707 | 0.00345352 | 0.00656597 |
| gene-BPHYT_RS13685 | 1481.28239 | 1572.46431 | 656.800383 | 516.646804 | 625.483666 | 545.941657 | 1.13660583 | 0.00345398 | 0.00656597 |
| gene-BPHYT_RS20725 | 50.2805982 | 65.0748458 | 36.6018789 | 94.6528495 | 98.4326885 | 82.3892054 | -0.85803866 | 0.00345654 | 0.00656853 |
| gene-BPHYT_RS27490 | 358.349025 | 288.036203 | 198.260178 | 396.358807 | 429.864272 | 446.35031 | -0.5879613 | 0.00346991 | 0.00659161 |
| gene-BPHYT_RS13830 | 989.649869 | 980.38989 | 887.595564 | 717.784109 | 760.049873 | 763.231869 | 0.35115253 | 0.00347713 | 0.006603 |
| gene-BPHYT_RS05810 | 8.77915207 | 5.33400375 | 10.1671886 | 22.6772452 | 17.4437676 | 38.931163 | -1.70667433 | 0.00348616 | 0.00661781 |
| gene-BPHYT_RS10595 | 249.806782 | 259.232582 | 222.66143 | 170.572323 | 130.828257 | 189.22356 | 0.57033831 | 0.00351407 | 0.00666846 |
| gene-BPHYT_RS09920 | 88.5896254 | 49.0728345 | 86.421103 | 130.147668 | 122.106373 | 132.184879 | -0.7728275 | 0.00356156 | 0.00675619 |
| gene-BPHYT_RS06050 | 863.549321 | 975.055886 | 945.548539 | 745.39119 | 662.863168 | 750.556607 | 0.36479746 | 0.00361075 | 0.0068471 |
| gene-BPHYT_RS26755 | 270.557505 | 343.509842 | 268.413779 | 375.653496 | 386.254854 | 491.619105 | -0.51003303 | 0.00363678 | 0.00689404 |
| gene-BPHYT_RS26610 | 206.709126 | 226.161759 | 266.380341 | 159.726684 | 178.175626 | 131.279503 | 0.57889402 | 0.00368204 | 0.00697738 |
| gene-BPHYT_RS18620 | 226.661744 | 217.627353 | 144.374078 | 386.499135 | 250.442663 | 283.382651 | -0.64457742 | 0.00376347 | 0.00712919 |
| gene-BPHYT_RS12050 | 264.172667 | 291.236605 | 258.24659 | 392.414939 | 322.7097 | 409.229899 | -0.47041798 | 0.00377277 | 0.0071443 |
| gene-BPHYT_RS26035 | 53.4730171 | 50.1396353 | 22.3678149 | 19.7193437 | 11.2138506 | 17.2021418 | 1.37918167 | 0.00380858 | 0.00720958 |
| gene-BPHYT_RS29685 | 270.557505 | 250.698176 | 177.9258 | 157.754749 | 144.534074 | 159.346155 | 0.59936866 | 0.00382071 | 0.00722748 |
| gene-BPHYT_RS33555 | 780.546429 | 678.485277 | 933.347913 | 599.468047 | 642.927434 | 499.767487 | 0.45940609 | 0.00382072 | 0.00722748 |
| gene-BPHYT_RS17600 | 409.427728 | 510.997559 | 631.382412 | 334.242875 | 350.121335 | 398.365389 | 0.51647748 | 0.00383099 | 0.00724437 |
| gene-BPHYT_RS14765 | 7044.07238 | 7683.099 | 9402.61601 | 7190.65866 | 5549.61006 | 4946.97383 | 0.4480092 | 0.003877 | 0.0073288 |
| gene-BPHYT_RS10225 | 52.6749124 | 38.404827 | 40.6687544 | 16.7614421 | 24.919668 | 17.2021418 | 1.18114594 | 0.00389169 | 0.007354 |
| gene-BPHYT_RS18575 | 441.351918 | 508.863958 | 568.345842 | 702.008634 | 723.916354 | 612.034097 | -0.42499748 | 0.00390646 | 0.00737932 |
| gene-BPHYT_RS18825 | 92.5801491 | 118.414883 | 148.440954 | 201.137305 | 194.37341 | 166.589163 | -0.64910733 | 0.003921 | 0.0074036 |
| gene-BPHYT_RS15445 | 20.7507231 | 20.2692143 | 11.1839075 | 40.4246545 | 53.5772861 | 32.5935318 | -1.26342788 | 0.00392206 | 0.0074036 |
| gene-BPHYT_RS07245 | 19.1545136 | 4.267203 | 13.2173452 | 31.5509498 | 38.6254854 | 35.3096594 | -1.49558559 | 0.00396392 | 0.00748 |
| gene-BPHYT_RS16025 | 187.554612 | 170.68812 | 219.611274 | 117.330095 | 118.368423 | 145.765517 | 0.59567768 | 0.0039724 | 0.00749339 |
| gene-BPHYT_RS06565 | 1431.00179 | 1648.20716 | 1701.98737 | 2002.49935 | 1982.35959 | 2010.83984 | -0.32740241 | 0.00401218 | 0.00756579 |
| gene-BPHYT_RS09710 | 67.0407976 | 81.076857 | 53.8860995 | 27.6070811 | 28.6576182 | 44.3634183 | 0.9947643 | 0.00401458 | 0.00756719 |
| gene-BPHYT_RS16410 | 29.5298751 | 12.801609 | 27.4514092 | 9.85967182 | 3.7379502 | 5.4322553 | 1.8635594 | 0.00401573 | 0.00756719 |
| gene-BPHYT_RS16675 | 1268.18842 | 1305.76412 | 1317.66764 | 1636.70552 | 1521.34573 | 1664.08087 | -0.31012885 | 0.00404065 | 0.00761149 |
| gene-BPHYT_RS14215 | 1336.02732 | 1427.3794 | 1455.94141 | 1060.90069 | 1224.80168 | 1041.18227 | 0.34377562 | 0.00408509 | 0.00769251 |
| gene-BPHYT_RS01515 | 731.063936 | 700.888093 | 573.429437 | 772.012304 | 950.685333 | 977.805954 | -0.42829053 | 0.00408689 | 0.00769322 |
| gene-BPHYT_RS09780 | 25.5393515 | 32.0040225 | 16.2675018 | 4.92983591 | 7.47590039 | 9.95913471 | 1.71604045 | 0.00409339 | 0.00770277 |
| gene-BPHYT_RS17440 | 70.2332165 | 74.6760525 | 89.4712596 | 119.302029 | 133.320224 | 121.320368 | -0.67500144 | 0.00411988 | 0.00774992 |
| gene-BPHYT_RS29880 | 44.6938651 | 45.8724323 | 35.5851601 | 21.691278 | 9.96786719 | 20.8236453 | 1.24474488 | 0.00412692 | 0.00776044 |
| gene-BPHYT_RS21960 | 185.958403 | 236.829767 | 188.092989 | 275.084844 | 267.886431 | 333.178325 | -0.52517125 | 0.00414782 | 0.00779704 |
| gene-BPHYT_RS37050 | 121.311919 | 82.1436578 | 96.5882916 | 54.228195 | 47.3473691 | 68.8085671 | 0.81337156 | 0.00416639 | 0.00782921 |
| gene-BPHYT_RS38755 | 6.38483787 | 6.4008045 | 0 | 21.691278 | 22.4277012 | 14.4860141 | -2.17196286 | 0.00421799 | 0.00792342 |
| Novel01097 | 205.112916 | 210.159748 | 279.597686 | 153.81088 | 115.876456 | 178.359049 | 0.62527136 | 0.00422592 | 0.00793555 |
| gene-BPHYT_RS34815 | 1338.42164 | 1189.48284 | 1206.84529 | 1723.47064 | 1569.93908 | 1452.22292 | -0.34476319 | 0.00424325 | 0.00796532 |
| gene-BPHYT_RS07675 | 62.2521692 | 43.7388308 | 53.8860995 | 107.470423 | 145.780058 | 59.7548083 | -0.9560499 | 0.00429589 | 0.00806132 |
| gene-BPHYT_RS21440 | 3.99052367 | 13.8684098 | 1.01671886 | 0 | 0 | 0 | 5.03682635 | 0.00430604 | 0.00807757 |
| gene-BPHYT_RS14695 | 22.3469325 | 27.7368195 | 17.2842206 | 5.91580309 | 6.22991699 | 8.14838295 | 1.72144994 | 0.00430848 | 0.00807934 |
| gene-BPHYT_RS28915 | 181.967879 | 228.295361 | 189.109708 | 131.133635 | 140.796124 | 134.901007 | 0.55777515 | 0.00434006 | 0.00813574 |
| gene-BPHYT_RS17975 | 165.20768 | 169.621319 | 162.675018 | 94.6528495 | 115.876456 | 119.509617 | 0.59267202 | 0.00434386 | 0.00814003 |
| gene-BPHYT_RS25325 | 14.3658852 | 14.9352105 | 20.3343772 | 5.91580309 | 0 | 3.62150353 | 2.31797595 | 0.00436959 | 0.0081854 |
| gene-BPHYT_RS20940 | 212.295859 | 273.100992 | 232.828619 | 177.474093 | 142.042107 | 170.210666 | 0.54605499 | 0.00438021 | 0.00820244 |
| gene-BPHYT_RS11165 | 11.971571 | 17.068812 | 17.2842206 | 0.98596718 | 2.4919668 | 5.4322553 | 2.34520231 | 0.0044008 | 0.00823815 |
| gene-BPHYT_RS01960 | 190.747031 | 222.961357 | 235.878775 | 303.677892 | 286.576182 | 311.449304 | -0.47733856 | 0.00443562 | 0.00830043 |
| gene-BPHYT_RS21240 | 1976.10732 | 1968.24738 | 1550.49626 | 1130.90436 | 1383.04157 | 1567.20565 | 0.42915069 | 0.00445726 | 0.00833804 |
| gene-BPHYT_RS29605 | 848.385331 | 885.444623 | 727.970703 | 660.598012 | 620.499732 | 618.371728 | 0.37409928 | 0.00448553 | 0.00838802 |
| gene-BPHYT_RS21365 | 255.393515 | 588.874014 | 259.263309 | 740.461354 | 841.038794 | 896.322124 | -1.16799128 | 0.004525 | 0.0084589 |
| gene-BPHYT_RS02115 | 456.515907 | 506.730356 | 460.573643 | 339.172711 | 371.303053 | 367.582609 | 0.40106253 | 0.00453306 | 0.00847103 |
| gene-BPHYT_RS17230 | 2656.09255 | 2697.9391 | 3262.65082 | 3732.87175 | 3548.56072 | 3562.6541 | -0.33198109 | 0.00453722 | 0.00847586 |
| gene-BPHYT_RS10905 | 75.8199497 | 55.473639 | 74.2204767 | 24.6491796 | 46.1013857 | 35.3096594 | 0.9687184 | 0.00455819 | 0.00851209 |
| gene-BPHYT_RS35810 | 39.9052367 | 14.9352105 | 24.4012526 | 11.8316062 | 7.47590039 | 4.52687942 | 1.74821249 | 0.00462295 | 0.00863005 |
| gene-BPHYT_RS07705 | 537.92259 | 597.40842 | 495.142085 | 420.02202 | 422.388372 | 393.838509 | 0.39966457 | 0.00463375 | 0.0086472 |
| gene-BPHYT_RS32670 | 75.8199497 | 82.1436578 | 73.2037579 | 24.6491796 | 51.0853193 | 46.17417 | 0.92994946 | 0.00464529 | 0.00866323 |
| gene-BPHYT_RS35785 | 11.1734663 | 18.1356128 | 8.13375087 | 37.4667529 | 27.4116348 | 32.5935318 | -1.3930373 | 0.00464555 | 0.00866323 |
| gene-BPHYT_RS34415 | 78.2142639 | 75.7428533 | 107.772199 | 44.3685232 | 58.5612197 | 44.3634183 | 0.83594704 | 0.00464876 | 0.00866623 |
| gene-BPHYT_RS20065 | 68.6370071 | 51.206436 | 52.8693807 | 28.5930483 | 36.1335186 | 21.7290212 | 1.01692954 | 0.00470029 | 0.00875925 |
| gene-BPHYT_RS13070 | 171.592518 | 201.625342 | 225.711587 | 290.860319 | 254.180613 | 304.206297 | -0.5093887 | 0.00473994 | 0.00883011 |
| gene-BPHYT_RS09940 | 486.045783 | 546.201984 | 573.429437 | 711.868306 | 631.713583 | 759.610366 | -0.39236644 | 0.00476451 | 0.00887281 |
| gene-BPHYT_RS26430 | 15.9620947 | 8.534406 | 19.3176583 | 0.98596718 | 0 | 5.4322553 | 2.683 | 0.00478628 | 0.00891027 |
| gene-BPHYT_RS04460 | 22.3469325 | 8.534406 | 11.1839075 | 34.5088514 | 43.6094189 | 32.5935318 | -1.36404739 | 0.00481303 | 0.00895699 |
| gene-BPHYT_RS12565 | 3917.89614 | 4175.45814 | 4977.85554 | 5545.07943 | 5339.03886 | 5564.44018 | -0.33185974 | 0.00482195 | 0.00897048 |
| gene-BPHYT_RS26275 | 30.3279799 | 24.5364173 | 28.4681281 | 9.85967182 | 8.72188379 | 11.7698865 | 1.44995351 | 0.00482622 | 0.00897533 |
| gene-BPHYT_RS07025 | 149.245585 | 147.218504 | 126.073139 | 195.221502 | 196.865377 | 220.911715 | -0.5368762 | 0.00486707 | 0.00904818 |
| gene-BPHYT_RS06130 | 55.0692266 | 52.2732368 | 61.0031316 | 26.6211139 | 34.8875352 | 24.4451488 | 0.98127291 | 0.00488387 | 0.00907628 |
| gene-BPHYT_RS35430 | 145.255061 | 135.483695 | 142.34064 | 241.56196 | 219.293078 | 169.30529 | -0.57166275 | 0.00490896 | 0.00911978 |
| gene-BPHYT_RS17240 | 939.369271 | 918.515446 | 1208.87872 | 1367.53648 | 1309.52855 | 1318.22729 | -0.38213067 | 0.00492233 | 0.00914147 |
| gene-BPHYT_RS33490 | 364.733863 | 333.908635 | 300.948782 | 444.671199 | 483.441559 | 417.378282 | -0.42522391 | 0.0049375 | 0.00916649 |
| gene-BPHYT_RS24625 | 1788.55271 | 1728.21722 | 1137.7084 | 1074.70423 | 1179.94628 | 1125.38222 | 0.46229567 | 0.00494112 | 0.00917006 |
| gene-BPHYT_RS04670 | 1193.96468 | 1442.31461 | 1310.55061 | 1621.91602 | 1622.27039 | 1715.6873 | -0.3307554 | 0.00499318 | 0.00926349 |
| gene-BPHYT_RS07910 | 85.3972065 | 54.4068383 | 87.4378219 | 23.6632124 | 52.3313027 | 38.931163 | 0.99831534 | 0.00502514 | 0.00931957 |
| gene-BPHYT_RS04015 | 43.8957603 | 41.6052293 | 18.3009395 | 8.87370464 | 7.47590039 | 19.0128935 | 1.53398331 | 0.00503017 | 0.00932569 |
| gene-BPHYT_RS31085 | 0 | 0 | 0 | 3.94386873 | 4.98393359 | 6.33763118 | -4.86119542 | 0.00503548 | 0.00933233 |
| gene-BPHYT_RS34640 | 104.55172 | 71.4756503 | 91.5046973 | 124.231865 | 164.469809 | 140.333262 | -0.67202465 | 0.00505896 | 0.00937263 |
| gene-BPHYT_RS34335 | 135.677805 | 132.283293 | 132.173452 | 183.389896 | 189.389477 | 204.61495 | -0.52943072 | 0.00506948 | 0.0093889 |
| gene-BPHYT_RS16220 | 330.41536 | 398.983481 | 343.650974 | 490.02569 | 459.767874 | 471.700835 | -0.40813316 | 0.005105 | 0.00945144 |
| gene-BPHYT_RS29325 | 173.188727 | 188.823733 | 222.66143 | 236.632124 | 337.661501 | 277.950396 | -0.54251215 | 0.00513754 | 0.00950841 |
| gene-BPHYT_RS10545 | 2292.15679 | 2285.08721 | 1855.51192 | 2635.49028 | 2901.89534 | 2622.87393 | -0.34260175 | 0.00517892 | 0.00958171 |
| gene-BPHYT_RS33975 | 5174.11299 | 5567.63312 | 5597.03732 | 4601.50884 | 4587.71087 | 4295.10319 | 0.27700114 | 0.00520796 | 0.00963213 |
[truncated: 152,222 more chars]
